# Carltonine-derived compounds for targeted butyrylcholinesterase inhibition†

**DOI:** 10.1039/d4md00060a

**Published:** 2024-03-22

**Authors:** Filip Pidany, Jana Kroustkova, Jaroslav Jenco, Katerina Hradiska Breiterova, Lubica Muckova, Lucie Novakova, Jiri Kunes, Jakub Fibigar, Tomas Kucera, Martin Novak, Ales Sorf, Martina Hrabinova, Lenka Pulkrabkova, Jiri Janousek, Ondrej Soukup, Daniel Jun, Jan Korabecny, Lucie Cahlikova

**Affiliations:** a Faculty of Pharmacy in Hradec Kralove, Department of Pharmacognosy and Pharmaceutical Botany, Charles University Akademika Heyrovskeho 1203 500 05 Hradec Kralove Czech Republic cahlikova@faf.cuni.cz; b Biomedical Research Center, University Hospital Hradec Kralove Sokolska 581 500 05 Hradec Kralove Czech Republic jan.korabecny@fnhk.cz; c Military Faculty of Medicine, Department of Toxicology and Military Pharmacy, University of Defence Trebesska 1575 500 01 Hradec Kralove Czech Republic; d Faculty of Pharmacy in Hradec Kralove, Department of Analytical Chemistry, Charles University Akademika Heyrovskeho 1203 500 05 Hradec Kralove Czech Republic; e Faculty of Pharmacy in Hradec Kralove, Department of Bioorganic and Organic Chemistry, Charles University Akademika Heyrovskeho 1203 500 05 Hradec Kralove Czech Republic

## Abstract

The investigation into human butyrylcholinesterase (*h*BChE) inhibitors as therapeutic agents for Alzheimer's disease (AD) holds significant promise, addressing both symptomatic relief and disease progression. In the pursuit of novel drug candidates with a selective BChE inhibition pattern, we focused on naturally occurring template structures, specifically Amaryllidaceae alkaloids of the carltonine-type. Herein, we explored a series of compounds implementing an innovative chemical scaffold built on the 3- and 4-benzyloxy-benzylamino chemotype. Notably, compounds 28 (*h*BChE IC_50_ = 0.171 ± 0.063 μM) and 33 (*h*BChE IC_50_ = 0.167 ± 0.018 μM) emerged as top-ranked *h*BChE inhibitors. *In silico* simulations elucidated the binding modes of these compounds within *h*BChE. CNS availability was predicted using the BBB score algorithm, corroborated by *in vitro* permeability assessments with the most potent derivatives. Compound 33 was also inspected for aqueous solubility, microsomal and plasma stability. Chemoinformatics analysis validated these *h*BChE inhibitors for oral administration, indicating favorable gastrointestinal absorption in compliance with Lipinski's and Veber's rules. Safety assessments, crucial for the chronic administration typical in AD treatment, were conducted through cytotoxicity testing on human neuroblastoma (SH-SY5Y) and hepatocellular carcinoma (HepG2) cell lines.

## Abstract 2

Innovative chemotype design provided 41 novel *h*BChE inhibitors. CNS availability was predicted using the BBB score. Potent derivatives showed favorable drug-like properties and acceptable safety profiles in SH-SY5Y and HepG2 cells.

## Graphical abstract

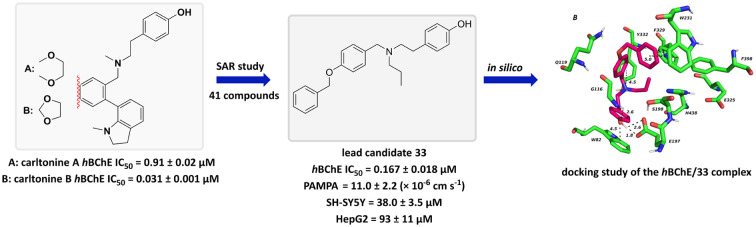

## 1. Introduction

Among the wide variety of enzymes present in the human body, cholinesterases (ChEs) stand out as the most effective ones.^1^ These serine hydrolases can be divided into two main groups based on their substrate and inhibitor specificities: acetylcholinesterase (AChE, E.C. 3.1.1.7) and the related butyrylcholinesterase (BChE, E.C. 3.1.1.8).^2,3^ Both enzymes belong to the α/β-hydrolase family, sharing catalytic mechanisms but differing in substrate specificity.^4^ AChE primarily serves as a catalyst, facilitating the hydrolytic transformation of the cationic neurotransmitter acetylcholine (ACh) into choline and acetic acid. This role is pivotal in transmitting nerve impulses across neuromuscular synapses.^5–8^ In a healthy human brain, AChE dominates, constituting approximately 90% of ChE activity, with BChE contributing the remainder.^9^ The human BChE gene, located on the third chromosome (3q26), exhibits poly-allelism and encodes a monomeric subunit comprising 574 amino acid residues with a molecular weight of approximately 65.1 kDa.^10^ BChE, synthesized in the human liver, is widespread in human plasma, brain tissue, and leg muscles, among other locations.^10–12^ From a structural point of view, AChE and BChE share almost the same backbone structure due to their approximately 65% homologous amino acid sequences.^13,14^ Both enzymes feature active sites composed of catalytic triad residues (Ser-His-Glu), a choline binding pocket, and an acyl-binding pocket, lodged at the bottom of a ∼20 Å deep gorge. The main difference at the molecular level lies in the acyl-binding pocket, a subunit domain within the gorge, responsible for accommodating the acyl moiety of substrates during hydrolysis.^15–17^ BChE's active site gorge is bulkier than AChE's in size (500 Å *vs.* 300 Å), forming a bowl-like shape instead of a narrow, deep gorge. Notably, BChE's gorge comprises approximately 40% fewer aromatic residues, which are replaced by smaller aliphatic residues.^18,19^ This substitution lowers substrate specificity, enabling BChE to adapt to a broader array of ligands and substrates. Besides its exclusive hydrolysis of choline esters, BChE is implicated in metabolizing esters such as butyrylcholine, succinylcholine, cocaine, or aspirin.^20–24^

Since Broomfield *et al.* first demonstrated the BChE neuroprotective function against neurotoxic agents in 1991, it has garnered substantial attention within the scientific community.^25^ Its pivotal role in neurodegenerative disorders, especially Alzheimer's disease (AD), has emerged as a focal point of research. AD is an insidious, multifactorial, age-related disease that accounts for the majority of dementia cases worldwide. AD has been listed as a global public health threat by the World Health Organization.^26–28^ Projections from the World Alzheimer Report indicate a staggering rise in dementia cases, increasing from 57.4 million in 2019 to an alarming 152.8 million cases globally by 2050, with significant regional disparities, particularly in lower- and middle-income countries.^29^ Despite numerous proposed mechanisms elucidating AD's pathogenesis, the underlying causes and optimal therapeutic interventions remain elusive. Several factors have been closely associated with AD progression, including i) neuro-cholinergic disturbances;^30–32^ ii) amyloid-β (Aβ) protein deposits;^33–35^ iii) neurofibrillary tangles (NFTs) formed by hyperphosphorylated tau protein,^36–38^ and iv) brain inflammation, immune responses, and oxidative stress.^39–42^ The U.S. Food and Drug Administration has approved seven drugs to treat AD.^43^ Three of these are classified as ChE inhibitors, falling into the categories of short-acting, reversible agents, such as donepezil and galantamine, and intermediate-acting, pseudo-irreversible agents, exemplified by rivastigmine.^44^ BChE is an enzyme closely related to AChE, serving as a co-regulator of cholinergic neurotransmission. In advanced AD stages, the depletion of cholinergic neurons leads to a 90% reduction in AChE levels, while BChE levels and activity elevate to 165% of normal levels, overtaking the main responsibility for the termination of cholinergic neurotransmission.^45–47^ These pathological changes are observed primarily in the hippocampus, a region intricately related to cognitive and memory functions. The direct connection between the etiopathogenesis of the disease and the procognitive effects of selective BChE inhibitors has been substantiated by their application in animal models.^48^ Additionally, BChE expression aligns with Aβ aggregation, in parallel with senile plaque formation and maturation.^49,50^ Studies in BChE knockout mice have shown reduced Aβ plaque deposition. Notably, these mice did not experience cognitive impairments after exposure to neurotoxic Aβ_25–35_, contrasting to impaired cognitive abilities observed in wild-type animals.^51^

Selective BChE inhibitors are endowed with other advantageous properties compared to AChE-selective inhibitors regarding the side effects. Unlike the peripheral side effects associated with general ChE inhibitors, such as cramps, gastrointestinal issues, and the risk of bradycardia, selective BChE inhibition circumvents these peripheral parasympathomimetic effects.^52,53^ The safety factor of enzyme inhibition is demonstrated by the fact that both animal and human BChE nullizygotes are viable and lead normal lifespans.^54,55^ Despite the existence of numerous cholinesterase inhibitors documented in the literature, the ongoing quest for novel scaffolds targeting AD persists as an essential scientific grail.

In our recent studies, we reported the isolation of Amaryllidaceae alkaloids (AAs) and the synthesis of first-in-class compounds possessing promising BChE inhibitory properties.^56–59^ A newly isolated and unique structural framework of AAs referred to as the carltonine-type expanded the chemical space by a new structural scaffold, designated as carltonines A–E. Building upon these findings, our subsequent investigations probe the synthesis of a first series of highly selective BChE inhibitors, shedding light on key aspects of the structure–activity relationship (SAR) pertinent to BChE inhibition. Here, we report the design, synthesis, and biological evaluation of a compound library as a follow-up to carltonine drug discovery, building upon the previously observed SAR. Beyond elucidating the compounds' ChE inhibitory role, we have predicted their blood–brain barrier (BBB) permeation, determined their cytotoxic profiles, and complemented our investigation with comprehensive docking studies. For the most promising compound 33 in the study, we have also determined aqueous solubility, microsomal and plasma stabilities. These analyses provide a comprehensive understanding of the potential of these compounds, paving the way toward the discovery of novel BChE inhibitors.

## 2. Design

Natural medicine has gained significant attention in recent years, underpinning its potential value built-in in active ingredients and newly discovered molecules. Natural products (NPs) exhibit distinctive properties compared to their synthetic counterparts, imposing both advantages and challenges in drug discovery.

NPs are typically endowed with by enormous skeletal diversity and structural complexity, a testament to their evolution over time, optimizing them to serve specific biological functions. Traditional medicine exploits the knowledge gained from NPs by providing valuable insights into their efficacy and safety.^60^ Compelling evidence of the practical application of NPs or the compounds derived from them in the therapy of a neurodegenerative diseases, such as AD, can be illustrated in the isoquinoline alkaloid galanthamine and the semi-synthetic drug rivastigmine, the latter derived from the carbamate physostigmine.^61^

As part of our work devoted to the isolation of new alkaloids from *Narcissus pseudonarcissus* cv. Carlton, we identified new species designated as carltonines A–D, all showing selective BChE inhibition patterns.^56,58^ Notably, carltonines A and B displayed remarkably potent inhibitory activity. However, the quantity of these isolated compounds was insufficient to conduct extensive follow-up biological assays. Based on the promising *h*BChE inhibition results demonstrated by carltonine A/B and the initial series of synthetic analogs, we opted to retain a pharmacophore likely encompassing crucial design requirements, particularly the 4-[2-(benzylamino)ethyl]phenol moiety.

**Fig. 1:**
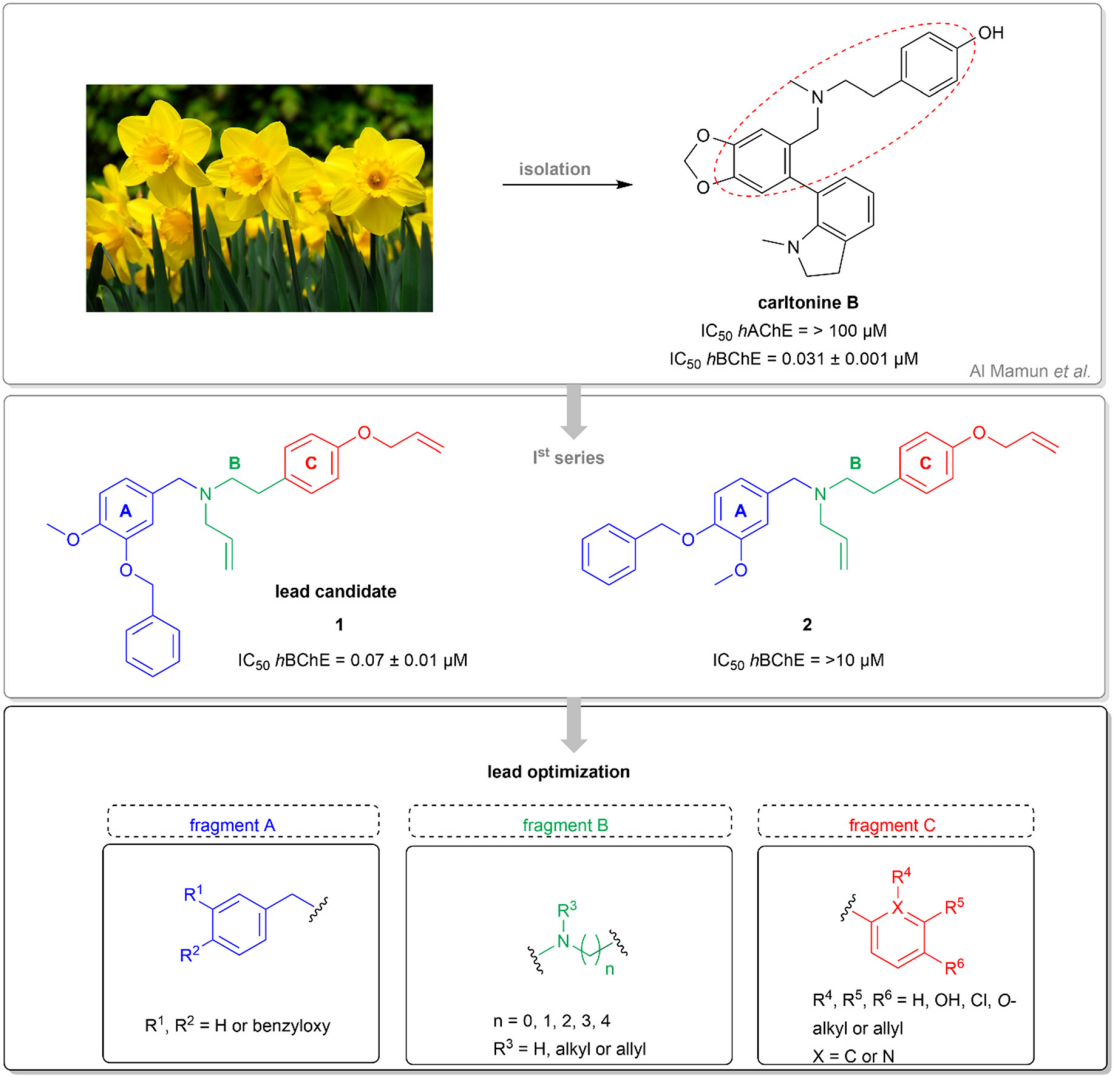
Chemical structures of template structures, namely carltonine-type Amaryllidaceae alkaloids isolated from *Narcissus pseudonarcissus* cv. Carlton carltonine B, template compounds 1 and 2, and the design of novel *h*BChE inhibitors relevant for this study.

Within this study, the fundamental disparity of the newly developed compounds compared to previously reported entities was concentrated on the absence of a methoxy group in the *meta* or *para* positions on fragment A. From the chemistry perspective, we followed essential SAR principles targeted to structural modifications in four regions: i) positional isomerism of the benzyloxy group regarding the first series to positions 3 and 4 (Fig. 1, fragment A), ii) expanding the chemical space between aromatic rings A and C using methylene spacer of various lengths (in the range of 0 to 4 carbons), iii) exploring the role of the basic center of the molecule (secondary/tertiary amine), in the case of tertiary amines, substitution with short alkyl chains (Fig. 1, fragment B), and iv) the substitution of various electron-withdrawing or electron-donating groups on the aromatic moiety (Fig. 1, fragment C). In this pursuit, a total of 41 novel potential BChE inhibitors were designed and synthesized, starting from the commercially available 3-benzyloxybenzaldehyde and 4-benzyloxybenzaldehyde.

## 3. Results

### 3.1 Chemistry

**Scheme 1:**
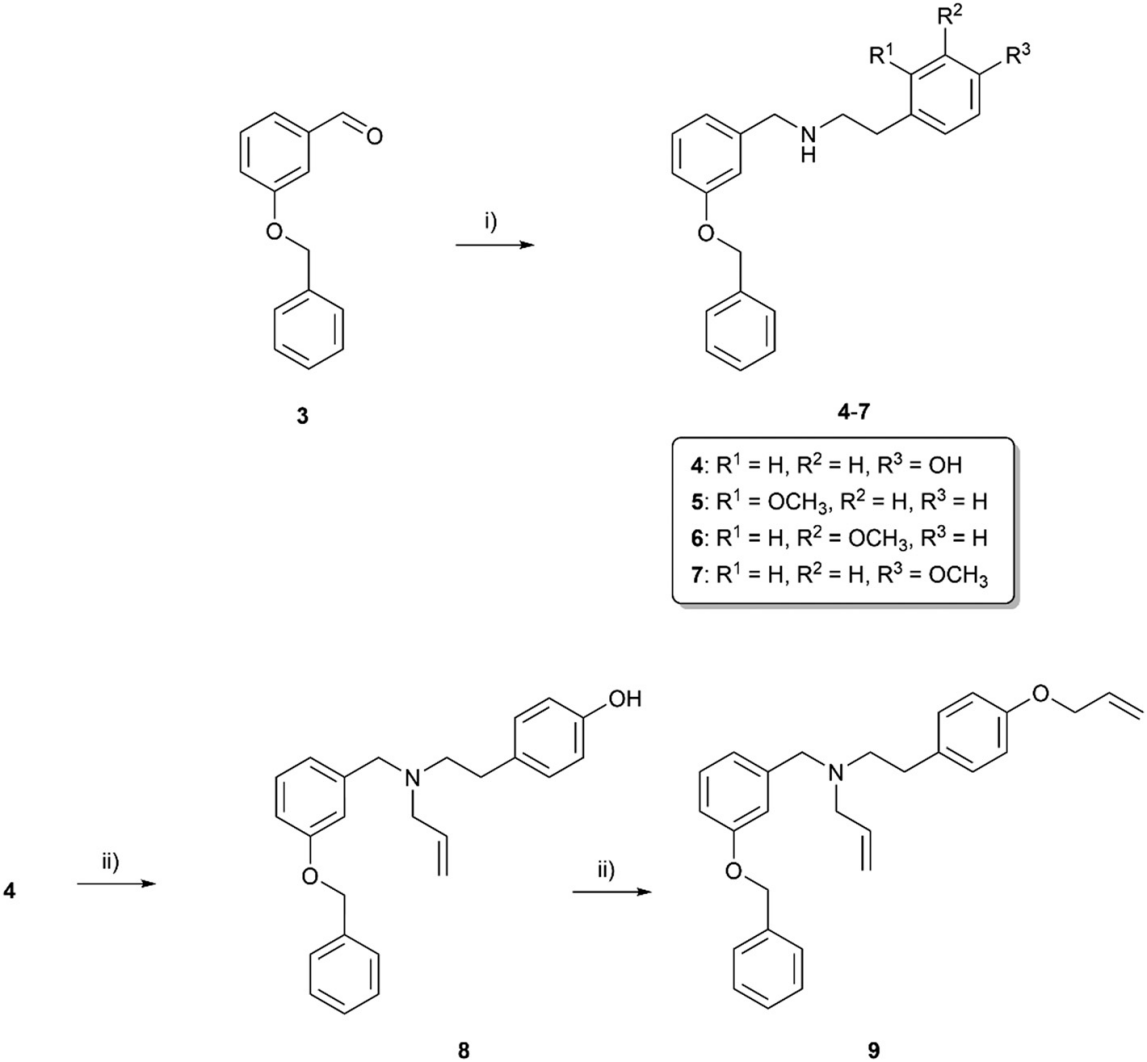
Synthesis of lead compound 4 and *o*-, *m*- and *p*-methoxy-substituted secondary amines 5–7, *N*-substituted derivative 8 and respective ether 9. Reagents and conditions: i) selected primary amine, MeOH, RT, 24 h, afterwards NaBH_4_, 0 °C → RT, 3 h; ii) allyl bromide, NaH, THF, RT, 24 h.

**Scheme 2:**
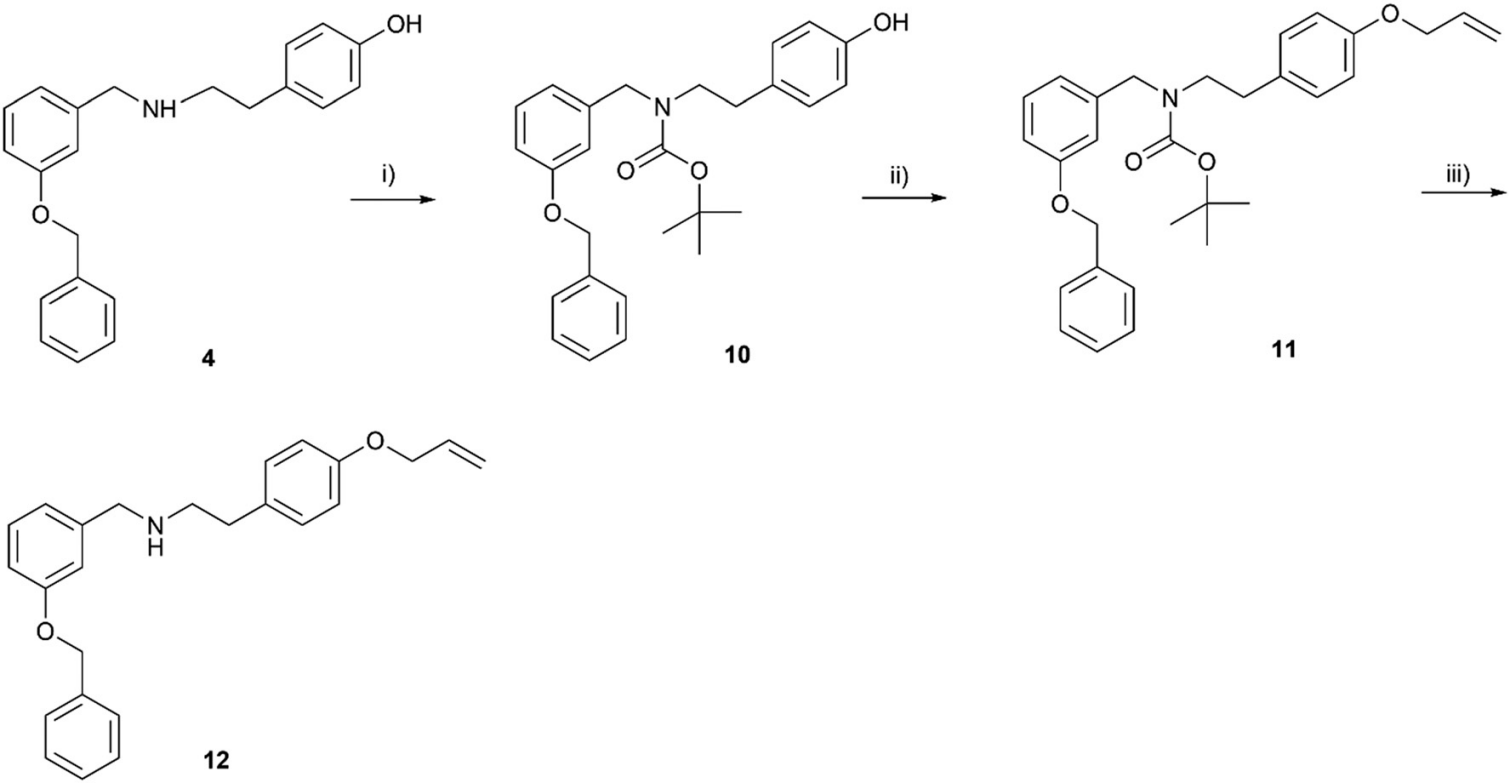
Synthesis of Boc-protected intermediates 10 and 11, and ether 12. Reagents and conditions: i) Boc_2_O, TEA, THF, RT, overnight; ii) allyl bromide, NaH, THF, RT, 24 h; iii) TFA, DCM, 0 °C → RT, 3 h.

**Scheme 3:**
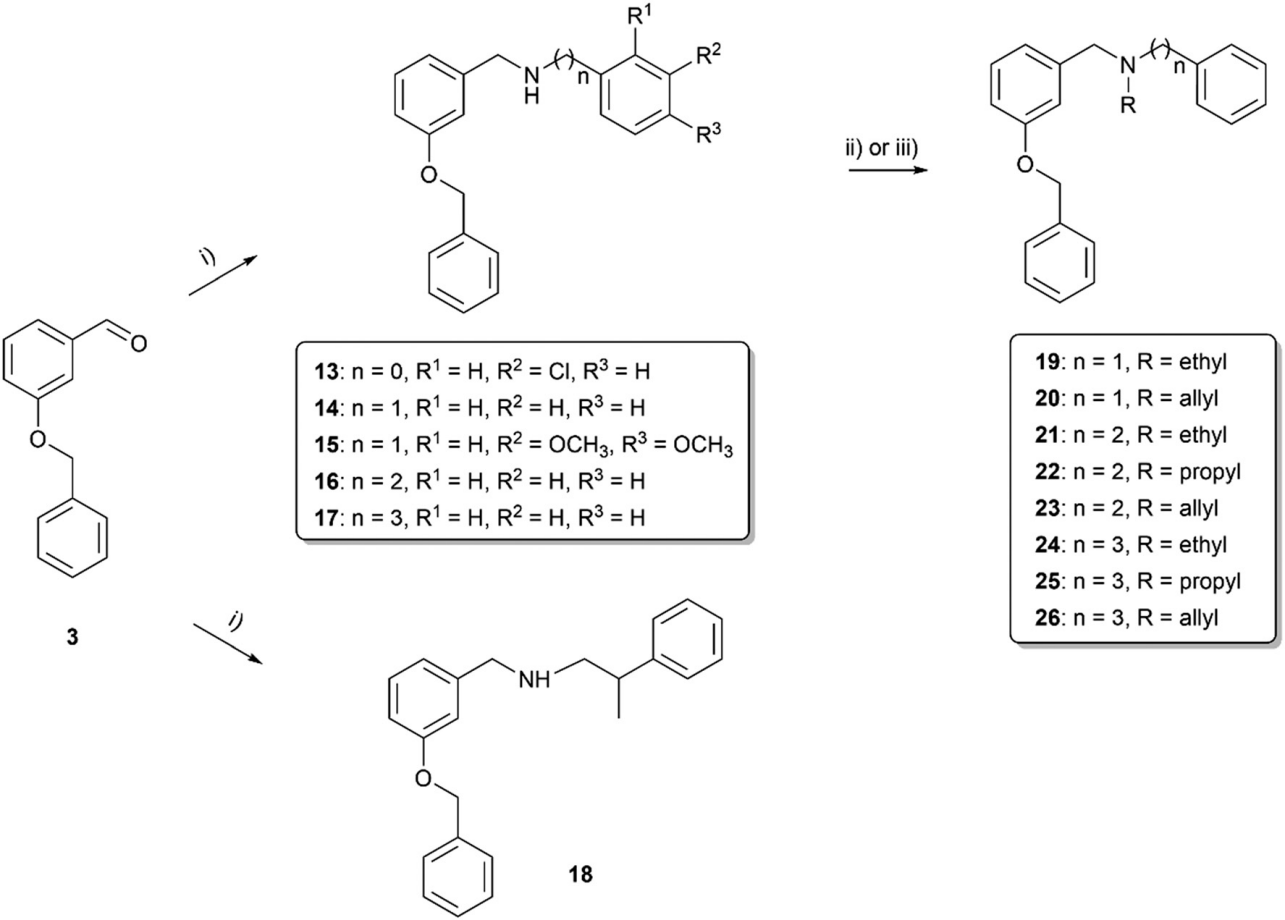
Synthesis of *m*-benzyloxy derivatives with various linker lengths 13–26 and branched derivative 18. Reagents and conditions: i) selected primary amine, MeOH, RT, 24 h, then NaBH_4_, 0 °C → RT, 3 h; ii) for 19, 21, 22, 24 and 25: selected alkyl halides, K_2_CO_3_, KI cat., CH_3_CN, reflux, 24 h; iii) for 20, 23 and 26: allyl bromide NaH, THF, RT, 24 h.

The synthetic route started from commercially available 3-benzyloxybenzaldehyde (3), which was reacted with tyramine to give the lead compound 4 under reductive amination conditions in an excellent yield of 95% (Scheme 1). To reveal the influence of the phenolic group present on fragment C in carltonines and template compounds from the first series, derivatives with a methoxy group present in the *o*-, *m*- and *p*- positions were synthesized (compounds 5–7; Scheme 1). Compound 4 was further structurally modified by allyl substitution to inspect the influence of tertiary amine and etherification on the activity, resulting in compounds 8 and 9, respectively. Nitrogen represents a more nucleophilic center than phenolic oxygen; thus, the substitution to tertiary amine was preferential. For comparative purposes, a secondary amine with an ether functional group 12 was prepared as a positional isomer of 8 (Scheme 2). In the first step, secondary nitrogen was protected in the reaction of 4 with di-*tert*-butyl dicarbonate (Boc_2_O) in the presence of triethylamine (TEA) to form carbamate 10. In the next step, the alkylation of the phenolic group took place, affording intermediate 11, followed by *N*-deprotection under acidic conditions using trifluoroacetic acid (TFA) to form 12. Afterward, the influence of the chain was investigated in terms of the length (compounds 13–26) and branching (compound 18), also considering aromatic substitution in fragment C (Scheme 3). Introducing ethyl, propyl, or allyl appendages, the secondary amine group was converted to tertiary amine (19–26; Scheme 3).

**Scheme 4:**
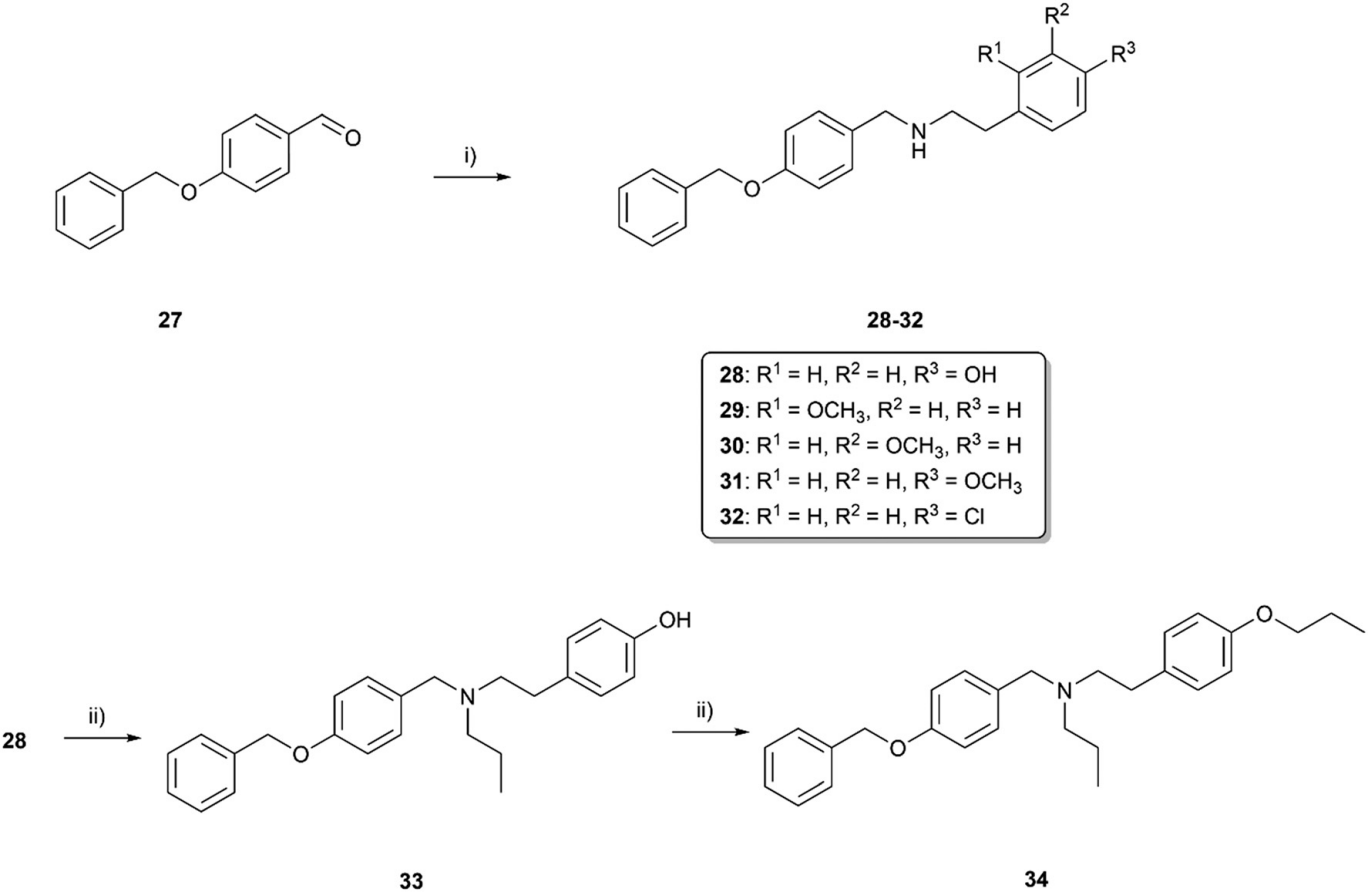
Synthesis of compound 28, *o*-, *m*- and *p*-methoxy and *p*-chloro-substituted secondary amines 29–32, nitrogen-substituted derivative 33, and respective ether 34. Reagents and conditions: i) selected primary amine, MeOH, RT, 24 h, then NaBH_4_, 0 °C → RT, 3 h; ii) propyl bromide, K_2_CO_3_, KI cat., CH_3_CN, reflux, 24 h.

**Scheme 5:**
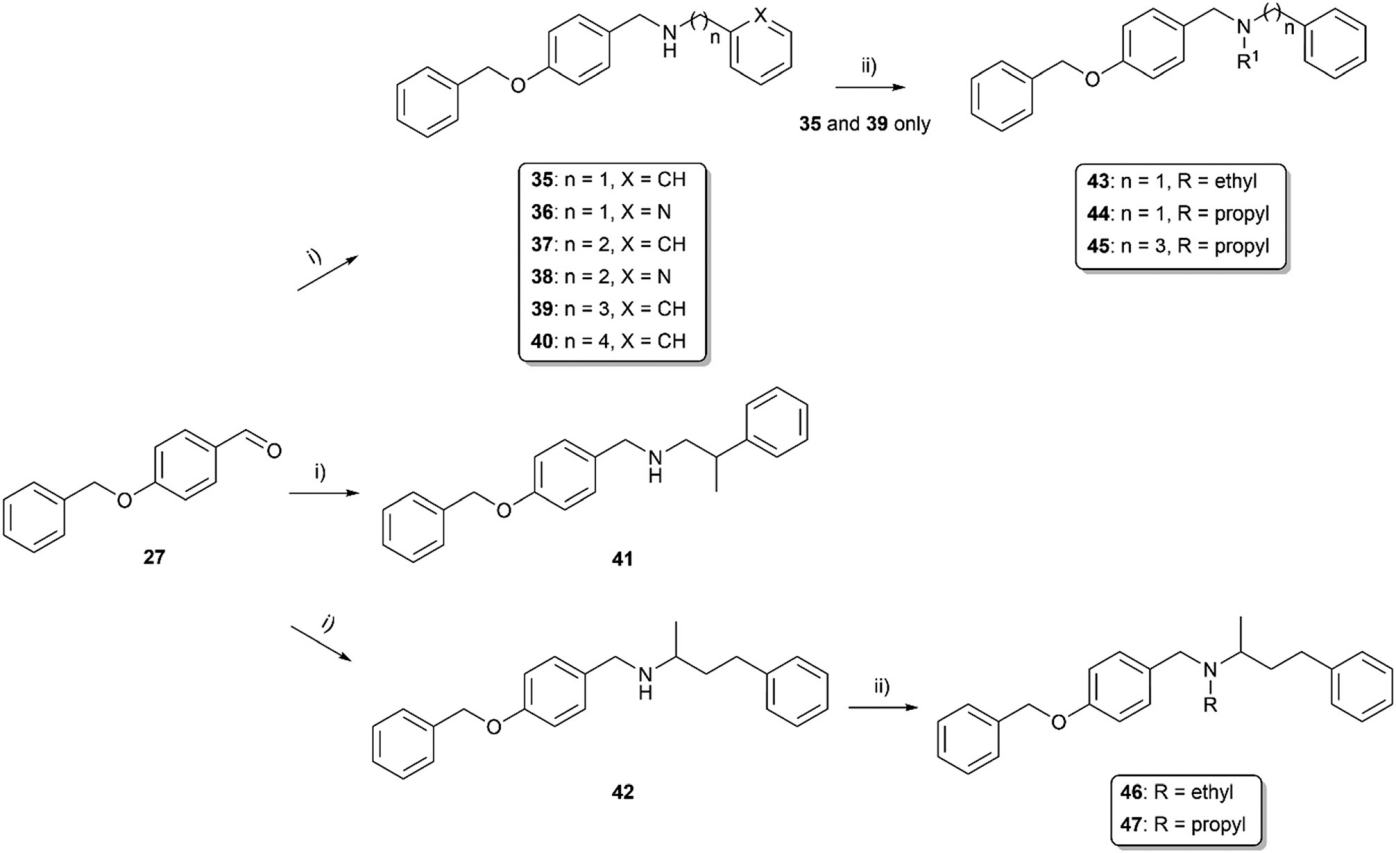
Synthesis of *p*-benzyloxy derivatives with various linker lengths 35–40, branched derivatives 41, 42 and respective tertiary amines 43–47. Reagents and conditions: i) selected primary amine, MeOH, RT, 24 h, then NaBH_4_, 0 °C → RT, 3 h; ii) alkyl halides, K_2_CO_3_, KI cat., CH_3_CN, reflux, 24 h.

As the endeavors with *m*-substituted compounds did not improve the desired activity, we turned our attention to positional isomers bearing a benzyloxy group in the *p*-position. The synthetic route started with reductive amination of aldehyde (27) and the appropriate hydroxy-, methoxy-, or chloro-substituted 2-phenylethan-1-amine (compounds 28–32; Scheme 4). Compound 28 was firstly alkylated at the secondary nitrogen with the propyl group to form 33. The next step involved etherification to generate compound 34. Reductive amination was also employed in the synthesis of compounds 35–42, probing the effect of the linker (elongation and branching) tethering the basic center to aromatic fragment C formed either by phenyl or pyridin-2-yl. Further, the secondary amine group in compounds 35, 39 and 42 was alkylated with ethyl or propyl substituents, enabling the formation of compounds 43–47 (Scheme 5). All the compounds were characterized by ^1^H and ^13^C NMR experiments and HRMS analysis, ensuring the structural confirmation of the newly synthesized molecules.

### 3.2 *In vitro h*AChE/*h*BChE inhibitory potencies

To determine the inhibitory potential of the synthesized compounds (4–9, 12–26, 28–47) against ChEs (*h*AChE and *h*BChE), we employed a modified Ellman's assay.^56,62^ Initial screening of the compounds at a concentration of 10 μM allowed us to identify candidates demonstrating >50% inhibitory potency against at least one cholinesterase. Subsequently, these selected compounds were subjected to detailed evaluation in a concentration-dependent manner to determine their IC_50_ values (Tables 1 and 2). The dose–response curves are attached in the ESI.† Galantamine and eserine were used as references with known *h*AChE/*h*BChE inhibition properties. Based on our assumptions supported by *in vitro* data, all the compounds showed very weak or no inhibitory capacity for *h*AChE when employing 10 μM concentration of the compound (data not shown) and were highly selective for *h*BChE. Almost all of the new compounds exhibited inhibitory potency for *h*BChE in the micromolar to nanomolar range (Tables 1 and 2).

**Table 1:** *In vitro h*AChE/*h*BChE inhibition of 4–9, 12–26 and their predicted CNS availability, estimated using a BBB score algorithm. Galantamine and eserine were used as positive controls

Compound	R^1^	R^2^	*n*	% inhibition of *h*BChE ± SEM^a^	IC_50_, *h*BChE ± SEM^b^ (μM)	BBB score^c^
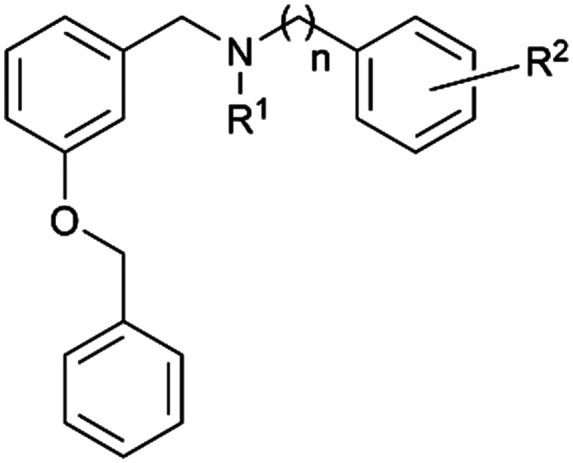						
4	H	4-OH	2	96.0 ± 0.8	**0.193 ± 0.055**	4.9
5	H	2-OMe	2	94.0 ± 0.4	0.4 ± 0.1	5.2
6	H	3-OMe	2	78.0 ± 0.4	2.7 ± 0.2	5.2
7	H	4-OMe	2	73.0 ± 0.6	4.8 ± 0.2	5.1
8	Allyl	4-OH	2	92.0 ± 0.5	0.6 ± 0.1	5.1
9	Allyl	4-Allyloxy	2	46.0 ± 1.7	>10	5.0
12	H	4-Allyloxy	2	75.5 ± 0.5	4.0 ± 1.0	5.1
13	H	3-Cl	0	7.5 ± 0.4	>10	4.8
14	H	H	1	92.0 ± 0.3	0.5 ± 0.1	5.3
15	H	3,4-DiOMe	1	49.0 ± 1.4	>10	4.9
16	H	H	2	91.0 ± 0.9	0.6 ± 0.1	5.3
17	H	H	3	89.0 ± 1.8	0.6 ± 0.1	5.3
18	H	H	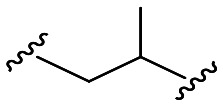	96.0 ± 0.3	**0.188 ± 0.023**	5.3
19	Ethyl	H	1	88.0 ± 1.1	0.5 ± 0.1	5.1
20	Allyl	H	1	36.0 ± 0.8	>10	5.1
21	Ethyl	H	2	91.0 ± 0.4	0.4 ± 0.1	5.1
22	Propyl	H	2	81.0 ± 0.8	0.7 ± 0.1	5.0
23	Allyl	H	2	60.0 ± 0.4	7.7 ± 1.7	5.0
24	Ethyl	H	3	94.0 ± 0.6	0.6 ± 0.1	5.0
25	Propyl	H	3	89.0 ± 0.8	0.54 ± 0.03	4.9
26	Allyl	H	3	76.0 ± 1.7	1.2 ± 0.3	5.0
**Galantamine** d				29.0 ± 3.5	34.0 ± 2.7	5.0
**Eserine** d				96.0 ± 0.3	0.30 ± 0.01	5.0

a Tested at 10 μM compound concentration.

b Compound concentration required to decrease enzyme activity by 50%; the values are the mean ± SEM of three independent measurements, each performed in triplicate.

c Calculated BBB score values.^63^

d Reference compound.

**Table 2:** *In vitro h*AChE/*h*BChE inhibition of 28–47 and their predicted CNS availability estimated by a BBB score algorithm. Galantamine and eserine were used as positive controls

Compound	R^1^	R^2^	*n*	X	% inhibition of *h*BChE ± SEM^a^	IC_50_, *h*BChE ± SEM^b^ (μM)	BBB score^c^
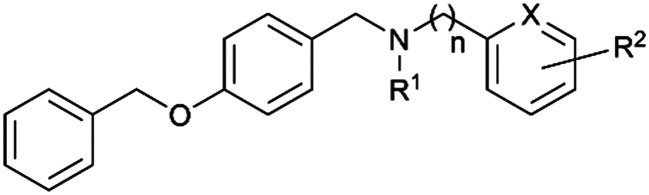							
28	H	4-OH	2	CH	97.0 ± 0.3	**0.171 ± 0.063**	4.9
29	H	2-OMe	2	CH	89.0 ± 0.9	0.8 ± 0.2	5.2
30	H	3-OMe	2	CH	66.0 ± 0.1	4.9 ± 1.0	5.1
31	H	4-OMe	2	CH	93.0 ± 0.2	3.0 ± 0.6	5.1
32	H	4-Cl	2	CH	85.0 ± 1.0	0.7 ± 0.2	5.2
33	Propyl	4-OH	2	CH	95.0 ± 1.1	**0.167 ± 0.018**	5.1
34	Propyl	4-Propyloxy	2	CH	30.0 ± 0.9	>10	5.0
35	H	H	1	CH	60.0 ± 0.1	2.8 ± 0.6	5.3
36	H	H	1	N	18.0 ± 0.5	>10	5.1
37	H	H	2	CH	81.0 ± 1.4	2.0 ± 0.3	5.3
38	H	H	2	N	23.0 ± 1.0	>10	5.2
39	H	H	3	CH	90.0 ± 0.3	0.7 ± 0.1	5.3
40	H	H	4	CH	85.0 ± 0.6	0.8 ± 0.1	5.2
41	H	H	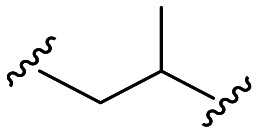	CH	88.0 ± 1.0	1.6 ± 0.1	5.2
42	H	H	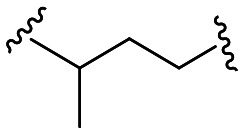	CH	92.0 ± 0.5	0.5 ± 0.1	5.2
43	Ethyl	H	1	CH	50.0 ± 1.1	>10	5.1
44	Propyl	H	1	CH	18.0 ± 0.3	>10	5.1
45	Propyl	H	3	CH	93.0 ± 0.8	0.29 ± 0.04	4.9
46	Ethyl	H	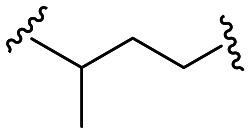	CH	87.0 ± 1.5	0.5 ± 0.3	4.9
47	Propyl	H	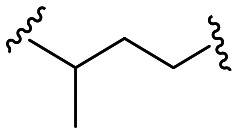	CH	93.0 ± 0.2	1.0 ± 0.3	4.9
**Galantamine** d					29.0 ± 3.5	34.0 ± 2.7	5.0
**Eserine** d					96.0 ± 0.3	0.30 ± 0.01	5.0

a Tested at 10 μM compound concentration.

b Compound concentration required to decrease enzyme activity by 50%; the values are the mean ± SEM of three independent measurements, each performed in triplicate.

c Calculated BBB score values.^63^

d Reference compound.

Initially, two pilot compounds revealed completely different *h*BChE inhibition patterns, with derivative 1 combining *O*-benzylisovanillin and tyramine with *N*- and *O*-allyl substitutions as the most active inhibitor of *h*BChE, IC_50_ = 72 ± 5 nM (Fig. 1). On the contrary, its positional derivative 2 derived from *O*-benzylvanillin was completely inactive in *h*BChE inhibition, IC_50_ > 10 μM (Fig. 1). Based on these findings, we decided to investigate the SAR in more detail, putting emphasis on the structure simplification. Indeed, newly developed compounds lack the methoxy group on fragment A.

Our initial attempts were devoted to the modification of compound 1, generating hydroxy-substituted positional isomers 4 and 28, both of them endowed with a better inhibitory profile (4: *h*BChE IC_50_ = 0.193 ± 0.055 μM; 28: *h*BChE IC_50_ = 0.171 ± 0.063 μM). In contrast, 3- and 4-methoxy derivatives discovered in a recent study were less active (data not shown; *h*BChE IC_50_ = 0.29 ± 0.02 μM and *h*BChE IC_50_ = 0.36 ± 0.03 μM).^57^

Subsequently, we aimed to discern the influence of the basic center on activity, specifically focusing on nitrogen substitution by allyl (8) and propyl (33). We also inspected the effect of etherification, namely substitution of hydroxyl by allyl and propyl in compounds 9 and 34, respectively. Allyl monosubstitution of compound 8 resulted in a slight deterioration of activity, whereas propyl monosubstitution in the case of 33 exhibited almost no shift in activity. However, disubstitution in the case of 9 and 34 led to a complete loss of *h*BChE inhibitory activity. Etherification of phenol with an allyl group in compound 12 resulted in activity decline, revealing that the substitution to tertiary amine is more favorable to improve inhibitory potency.

Our attention then shifted to fragment C, where we investigated the replacement of the phenolic hydroxyl group with a methoxy group attached at different positions (compounds 5–7 and 29–31) or an electron-accepting chlorine atom (compound 32). The activity increased in the order of *p*- > *m*- > *o*-positions of the methoxy group. However, neither methoxy nor chlorine substitution led to a fundamental improvement in inhibitory activity.

Further efforts were directed to the length of the connecting linker between the two aromatic rings (fragment B). From the family of compounds related to 3-benzyloxybenzaldehyde, compounds having linkers ranging from 0 to 4 methylene units were prepared. Compound 13, originating from 3-chloroaniline and compound 15 (3,4-dimethoxy substituted) were completely inactive. Other compounds (14, 16 and 17) with linkers comprising 1 to 3 methylene units exhibited nearly identical results, with compounds 14 and 17 displaying *h*BChE IC_50_ values of 0.5 ± 0.1 μM and compound 16 exhibiting a *h*BChE IC_50_ value of 0.6 ± 0.1 μM. In contrast, for compounds derived from 4-benzyloxybenzaldehyde (35–40), a more evident improvement in inhibitory activity was observed with increasing linker length, although these compounds were generally less active than the compound from the previous group. The introduction of a pyridine heterocycle into fragment C (36 and 38) negatively impacted inhibition ability. Branching the connecting linker of the first group of compounds (Table 1) derived from the chain isomer 17 increased the activity, highlighting compound 18 (*h*BChE IC_50_ = 0.188 ± 0.023 μM).

In the case of the second group of compounds derived from 27 (Table 2), a deterioration in activity was observed with the same linker, as evidenced by compound 41 (*h*BChE IC_50_ = 1.6 ± 0.1 μM), while a slight improvement was achieved with an extended branched chain, represented by compound 42 (*h*BChE IC_50_ = 0.5 ± 0.1 μM).

Following the moderate success of nitrogen alkylation, wherein compound 33 demonstrated *h*BChE inhibition properties, attempts were made to modify the tertiary amine by introducing short-to-medium length substituents (*N*-allyl, *N*-ethyl, *N*-propyl) in combination with achiral and chiral compounds (19–26 and 43–47). However, these efforts resulted in negligible changes in *h*BChE inhibition. In all cases, the pattern of selective *h*BChE inhibition was maintained, with IC_50_ values ranging from micromolar to submicromolar levels.

### 3.3 Docking studies

To distinguish between the determinants responsible for the high inhibitory activity of compound 33 and BChE-inactive compound 9, we performed molecular modeling in tandem with molecular dynamics simulation within the *h*BChE active site (PDB ID: 6QAA).^64^ The choice of the enzyme was dictated by the high resolution of the ligand–enzyme complex solved at 1.9 Å and also by the nature of the ligand within the co-crystal structure, being a highly selective and reversible BChE inhibitor.

**Fig. 2:**
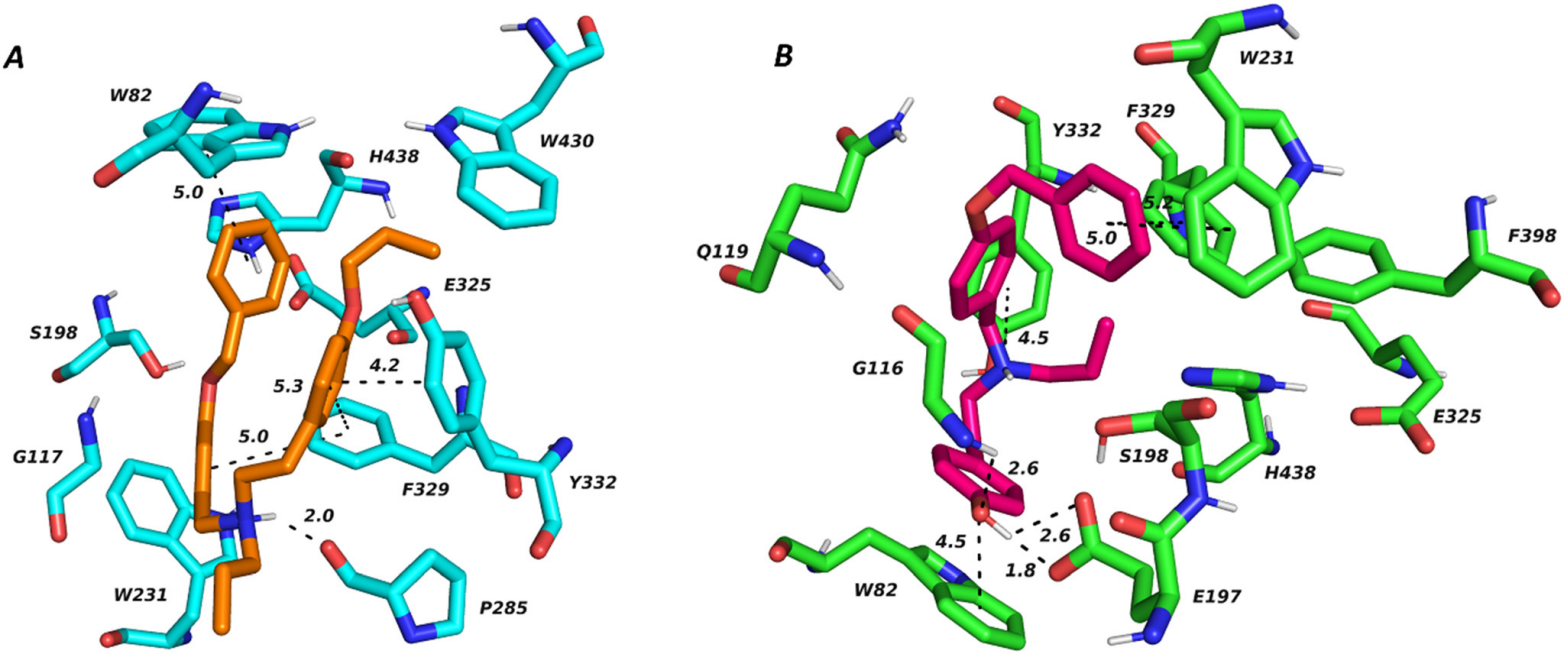
Top-scored docking pose of compounds 9 (A) and 33 (B) colored as orange and purple sticks, respectively. Important amino acid residues in close ligand vicinity are rendered in light-blue and green, respectively. Crucial interactions are displayed in dashed lines, and the distance is given in Å. The figure was created in the PyMOL Molecular Graphics System, version 2.5.2, Schrödinger, LLC.

The top-scored docking pose of compound 9 (Fig. 2A) revealed U-shaped characteristics of the ligand. Indeed, the compound seems to be distorted within the *h*BChE active site, providing mainly several hydrophobic interactions with aromatic residues like F329, Y332 and W82. The protonated ammonium moiety of the ligand can presumably be in contact with the backbone amide of P285. F329 is the main driver for the compound distortion. In line with the inappropriate geometry of the molecule given by the 3-benzyloxy substitution, that is, a “hard-to-fit” enzyme gorge, the overall topology can be conceived as improbable. On the other hand, *h*BChE active ligand 33 revealed a completely different binding pose, spanning BChE active in an extended conformation (Fig. 2B). Accordingly, compound 9 comes into contact with both tryptophan residues, W82 by parallel π–π stackings and W231 *via* distorted π–π interaction. The ligand is anchored in its center at the protonated ammonium moiety to Y332 *via* cation–π interaction. The free phenolic hydroxyl group of 9 can be considered pivotal to maintaining high ligand affinity as it is implicated in several hydrogen contacts, specifically to E197 and G116. The latter, along with the extended geometry of the molecule, contributes to a better orientation of compound 33 into the *h*BChE active site.

### 3.4 Enzyme kinetic analysis of compound 33

**Chart 1:**
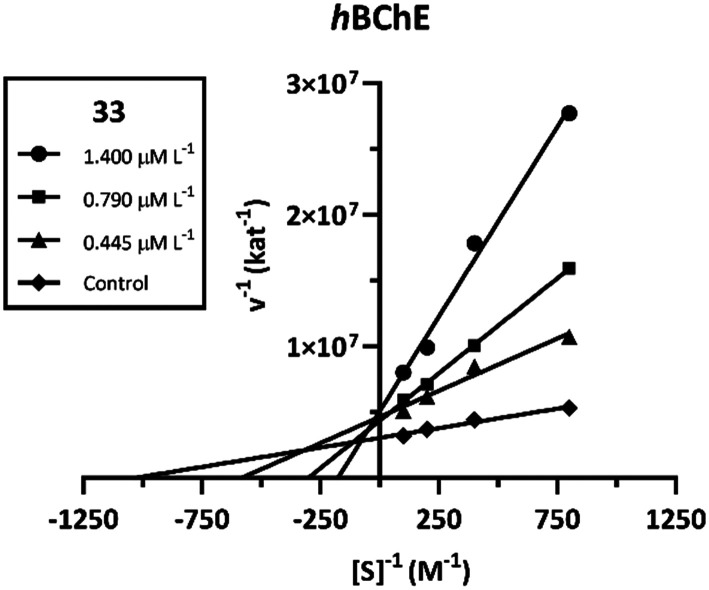
Steady state inhibition of *h*BChE substrate hydrolysis by compound 33 at different concentrations. Lineweaver–Burk plots of initial velocity at increasing substrate concentrations are presented. Lines were derived from a linear regression of the data points, measured in triplicate.

A kinetic study was performed to estimate the interaction mode of derivative 33 with *h*BChE. The inhibition kinetics was calculated from rate curves that were determined at several concentrations of the tested compound and substrate. The type of enzyme inhibition and appropriate kinetic parameters (*K*_i_ and *K*_i′_) were determined using nonlinear regression analysis. Results for each type of inhibition model (competitive, non-competitive, uncompetitive and mixed) were compared by the sum-of-squares *F*-test. Statistical analysis showed a mixed inhibition pattern of *h*BChE (*p* < 0.0002), consistent with the Lineweaver–Burk plot, used for visualization of the obtained data (Chart 1).

The intersection of lines is located above the *x*-axis, which means reversible binding mode to both the free enzyme and enzyme–substrate complex, with higher affinity to the free enzyme (*K*_i_ < *K*_i′_). Both *K*_m_ and *V*_max_ were decreased at increasing concentrations of the inhibitor. A *K*_i_ value of 181.6 ± 28 nM and *K*_i′_ of 1619 ± 315 nM, respectively, were determined for 33 towards *h*BChE.

### 3.5 Bioavailability prediction

The blood–brain barrier (BBB), a highly selective membrane, restricts the passage of substances from the bloodstream into the brain, including potential therapeutic drugs. For AD, it is essential that drugs must effectively cross this barrier to reach the brain and exert their intended effects. According to these prerequisites, we have applied an *in silico* calculation model of the so-called “BBB score”.^63^ The algorithm employed in this study functions as a predictive tool for CNS penetration, enabling us to distinguish between drugs specifically targeted for the CNS and those with non-CNS targets. To assess the physicochemical properties relevant to CNS penetration, various descriptors such as molecular weight, topological polar surface area, p*K*_a_, number of heavy atoms, aromatic rings, and the count of hydrogen bond donors and acceptors were computed using MarvinSketch software (ChemAxon Ltd., v.21.14.0). Compound BBB score values exceeding 4.0 are hypothesized to possess the capability to permeate the CNS. Notably, all novel compounds investigated in this study (compounds 4–9, 12–26, 28–47) exhibit BBB score values ranging from 4.8 to 5.3, as detailed in Tables 1 and 2, presuming their potential to cross the blood–brain barrier and enter the CNS.

Furthermore, we experimentally determined the ability of 12 highlighted *h*BChE inhibitors to pass through the BBB using a parallel artificial membrane permeation assay (PAMPA) as described by Di *et al.*^65^ The measurement (Table 3) shows that all selected compounds are expected to pass through the BBB by passive diffusion. The methodology was validated on a set of compounds (positive and negative controls) recognized for their activity in the CNS (Table 3).

**Table 3:** Prediction of BBB penetration of the selected compounds and reference drugs, expressed as Pe ± SEM (*n* = 3)

Compound	BBB penetration estimation^a^	
	Pe ± SEM (×10^−6^ cm s^−1^)	CNS (+/−)
4	14.0 ± 0.4	CNS +
5	6.2 ± 0.7	CNS +
14	9.8 ± 2.9	CNS +
18	8.1 ± 0.6	CNS +
19	36 ± 14	CNS +
21	21.0 ± 0.6	CNS +
25	33.0 ± 6.8	CNS +
28	10.0 ± 0.5	CNS +
33	11.0 ± 2.2	CNS +
42	22.0 ± 4.2	CNS +
45	25.0 ± 2.9	CNS +
46	36.0 ± 2.0	CNS +
Furosemide	0.20 ± 0.07	CNS −
Chlorothiazide	1.1 ± 0.5	CNS −
Galantamine	6.7 ± 0.7	CNS +
Donepezil	22.0 ± 2.1	CNS +
Rivastigmine	20.0 ± 2.1	CNS +
Tacrine	6.0 ± 0.6	CNS +

a CNS (+) (high BBB permeation predicted): Pe (×10^−6^ cm s^−1^) > 4.0; CNS (−) (low BBB permeation predicted): Pe (×10^−6^ cm s^−1^) < 2.0; CNS (+/−) (BBB permeation uncertain): Pe (×10^−6^ cm s^−1^) from 4.0 to 2.0.

### 3.6 Cytotoxicity profile determination

In light of the CNS being the proposed target site for the action of the investigated compounds, the neurotoxicity profile of the most potent *h*BChE inhibitors (compounds 4, 5, 18, 28, and 33) was assessed using the human neuroblastoma cell line (SH-SY5Y) through a colorimetric MTT (3-[4,5-dimethylthiazol-2-yl]-2,5-diphenyltetrazolium bromide) assay (Table 4). Considering the chronic nature of AD necessitating prolonged medication usage, the hepatotoxicity of the synthesized compounds was also evaluated on the human hepatocyte carcinoma cell line (HepG2). An elevated sensitivity of the SH-SY5Y cell line was observed for the derivatives under investigation, suggesting an augmented vulnerability of the neuronal cell line. Among these derivatives, compounds 28 (IC_50_ = 13 μM) and 5 (IC_50_ = 16 μM) exhibited the highest cytotoxicity towards SH-SY5Y. Galantamine and the lead compound 1 from the first series were employed as reference compounds for comparative analysis. The data indicated that despite their elevated neurotoxicity, the newly synthesized compounds can be deemed safe (as predicted by safety index values in Table 4) due to their significantly superior inhibitory efficiency against *h*BChE.

**Table 4:** Cytotoxicity data received from two cell lines. Galantamine was used as a positive control

Compound	SH-SY5Y cell IC_50_ (μM) ± SEM^a^	HepG2 cell IC_50_ (μM) ± SEM^a^	Safety index^c^
4	21.0 ± 2.4	60.0 ± 4.0	310
5	16.0 ± 2.8	52 ± 10	130
18	36.0 ± 1.4	55.0 ± 6.9	290
28	13.0 ± 1.2	85 ± 12	500
33	38.0 ± 3.5	93 ± 11	560
1	>125	n.t.^b^	—
**Galantamine**	4700 ± 210	4200 ± 200	120

a The results are expressed as the mean of a minimum of three experiments.

b n.t. = not tested.

c Safety index is calculated as cytotoxicity for HepG2 cells IC_50_/*h*BChE IC_50_.

### 3.7 Physicochemical properties and druglikeness of selected compounds predicted by SwissADME

The evaluation of *in silico* physicochemical and pharmacokinetic properties of synthesized compounds is crucial in the early stages of drug discovery. The easy-to-use and freely available SwissADME software (SwissADME) has emerged recently as an indispensable tool for medicinal chemists.^66^ To shed light on the physicochemical properties of developed small molecules, Table 5 provides a comprehensive overview of essential ADME-related parameters such as molecular weight, rotatable bonds, hydrogen bond donor and acceptor counts, partition coefficient octanol–water, topological polar surface area, and water solubility. Chemoinformatics analysis of the most potent *h*BChE inhibitors in our study revealed that, with the exception of compound 45, all other derivatives adhered to Lipinski's rule of five.^67^ In addition, these compounds also complied with Veber's rules, displaying a number of rotatable bonds less than 10 and a topological polar surface area (TPSA) below 140 Å^2^. This finding indicates high potential for gastrointestinal absorption, suggesting their suitability for oral administration.^68^ A crucial determinant of a drug's ADME profile is its aqueous solubility (ESOL, Table 5). Our results indicate that the synthesized compounds exhibit a balanced hydrophilic–lipophilic profile, demonstrating moderate solubility and lipophilicity (log *P* below 5). This balanced profile is indicative of their potential as drug candidates, as it aligns with the optimal physicochemical characteristics necessary for successful drug development. Furthermore, we also asssesed the susceptibility of the newly discovered drugs to P-glycoprotein (P-gp) efflux. Understanding the physiological role of the P-gp efflux transporter is imperative, as it elucidates whether the compounds are prone to externalization or excretion processes, ultimately impacting their bioavailability. However, only three of these compounds were found to be resistant to active efflux mediated by P-gp.

**Table 5:** Predicted drug-like and physicochemical properties for the selected compounds using various predictive models

Code	MW	Lipinski's rule of five				Drug-likeness	GI absorption		Solubility ESOL		P-gp
		Rot. bonds	HBA	HBD	clog *P*_o/W_	LP violation	Class	TPSA	Class	mg L^−1^	
4	333.4	8	3	2	4.10	Yes; 0	High	41.49	Moderately	7.31	No
5	347.5	9	3	1	4.46	Yes; 0	High	30.49	Moderately	4.79	No
18	331.5	8	2	1	4.84	Yes; 1	High	21.26	Moderately	2.79	Yes
						MLOGP >4.15					
21	345.5	9	2	0	5.06	Yes; 1	High	12.47	Moderately	1.39	Yes
						MLOGP >4.15					
28	333.4	8	3	2	4.10	Yes; 0	High	41.49	Moderately	7.31	No
33	375.5	10	3	1	4.99	Yes; 1	High	32.70	Moderately	9.71	Yes
						MLOGP >4.15					
45	387.6	11	2	0	5.99	Yes; 1	Low	12.47	Poorly	0.195	Yes
						MLOGP >4.15					
**Required**	≤500	≤10	≤10	≤5	≤5	—	—	≤140	—	—	—

### 3.8 Aqueous solubility, microsomal and plasma stability evaluation

The top-ranked compound 33 was investigated for aqueous solubility, yielding a concentation of 267 μM. To further investigate the drug-likeness of this compound, human liver microsomal (HLM) and plasma stabilities were investigated. Herein, we used standards diazepam and verapamil with low and high metabolic clearance, respectively, and compared their HLM stability after 45 min of incubation with compound 33. Consistent with the existing literature, verapamil exhibited high intrinsic clearance in microsomal stability assessments (CL_int_ = 90.1 μL min^−1^ mg^−1^) with a short half-life (*T*½ = 15.40 min), while diazepam demonstrated low metabolic clearance (CL_int_ = 4.6 μL min^−1^ mg^−1^) and an extended half-life (*T*½ = 301.30 min).^69,70^ In comparison to the reference compounds, compound 33 demonstrated a shorter half-life (*T*½ = 8.62 min) similar to the rapid clearance observed with verapamil. However, compound 33 displayed a slightly elevated intrinsic clearance (CL_int_ = 161.0 μL min^−1^ mg^−1^) relative to verapamil, implying a higher metabolic turnover rate. After the incubation of compound 33 with human plasma, 94.5% of the compounds remained unchanged after 120 min, proposing a plasma-stable profile (Table 6).

**Table 6:** Aqueous solubility, microsomal stability determined as HLM half-life (HLM *T*½), intrinsic clearance (CL_int_) and plasma stability (%) for compound 33. Diazepam and verapamil were selected for comparative purposes, displaying low and high CL_int_, respectively

Compound	Aqueous solubility [μM]	HLM *T*½ (min)	CL_int_ (μL min^−1^ mg^−1^)	Plasma stability^a^ (%)
33	267	8.62	161.0	94.5
Diazepam	n.d.^b^	301.30	4.6	n.d.
Verapamil	n.d.	15.40	90.1	n.d.

a Percentage of the remaining compound after 120 min incubation.

b n.d. = not determined.

## Conclusions

The symptoms of AD are primarily attributed to the dysfunction of cholinergic neurotransmission. Both AChE and BChE enzymes control cholinergic activity. Notably, BChE exhibits a more pronounced function during the disease's advanced stages. In this study, we systematically designed, synthesized, and subjected 41 selective *h*BChE inhibitors, inspired by carltonine-type alkaloids, to rigorous *in vitro* evaluations. This study is a direct continuation to our preliminary study,^57^ delving into the distinctive cholinesterase activity of methoxy group-containing positional isomers 1 and 2. Newly introduced compounds exhibited negligible inhibitory activity against *h*AChE while displaying remarkable selectivity for *h*BChE, displaying their inhibitory efficacy within the micromolar to sub-micromolar range. In particular, both *m*- and *p*-benzyloxy isomers (4: *h*BChE IC_50_ = 0.193 ± 0.055 μM; 28: *h*BChE IC_50_ = 0.171 ± 0.063 μM) demonstrated substantial activity. Compound 33 emerged as the most potent inhibitor (IC_50_ = 0.167 ± 0.018 μM) followed by its precursor secondary amine 28, with formation of the tertiary amine leading to a slight increase in activity. To support the obtained *in vitro* outcomes, we conducted an *in silico* study, comparing the binding interactions of the highly active molecule 33 with the inactive compound 9. Molecular modeling and dynamics simulations predicted that disubstitution in 3-benzyloxy derivatives induces a U-shaped conformation, hindering proper enzyme gorge accommodation. Conversely, the active ligand 33 exhibited a distinctive binding orientation, adjusting the *h*BChE active site properly. Utilizing the BBB score algorithm, we predicted the CNS accessibility for selected compounds. These theoretical observations were further validated through an *in vitro* permeability assessment (PAMPA-assay) for 12 of the most potent *h*BChE inhibitors. Safety assessments, crucial for chronic AD treatment, indicated minimal cytotoxic effects on SH-SY5Y and HepG2 cell lines for the five most active compounds. Additionally, our chemoinformatics analysis confirmed the adherence of the potent *h*BChE inhibitors to both Lipinski's and Veber's rules, highlighting their potential for gastrointestinal absorption and oral administration. These theoretical predictions were substantiated through empirical verification, demonstrating high plasma and satisfactory HLM stabilities of compound 33. The presented series of *h*BChE inhibitors, centered on the *N*-benzyl-2-phenylethan-1-amine moiety, stands as a promising scaffold for the continual advancement of anti-AD therapeutics.

## 4. Materials and methods

### 4.1 General chemistry

All solvents were treated using standard techniques before use. All reagents and catalysts were purchased from Sigma-Aldrich Co. LLC or Fluorochem Ltd. and were used without additional purification. Analytical thin-layer chromatography (TLC) was carried out using plates coated with silica gel 60 with a fluorescent indicator F254 (Merck, Prague Czech Republic). TLC plates were visualized by exposure to ultraviolet light (254 and 366 nm). The NMR spectra were obtained in CDCl_3_ at ambient temperature on a VNMR S500 (Varian) spectrometer operating at 500 MHz for ^1^H and 126 MHz for ^13^C and on a JNM-ECZ600R (Jeol) instrument operating at 600 MHz for ^1^H and 151 MHz for ^13^C. Chemical shifts were recorded as *δ* values in parts per million (ppm) and were indirectly referenced to tetramethylsilane (TMS) *via* the residual solvent signal of chloroform-d_1_ (CDCl_3_ – 7.26 (H), 77.0 (C) ppm). Coupling constants (*J*) are given in Hz. ESI-HRMS data were obtained with a Waters Synapt G2-Si hybrid mass analyzer of quadrupole-time-of-flight (Q-TOF) type, coupled to a Waters Acquity I-Class UHPLC system. Chromatographic analysis data were obtained with a Waters HPLC-PDA-MS system (Waters Corporation, Milford, Massachusetts, USA). Analysis confirmed the purity of all the compounds >95% (PDA detection, UV-vis, uncalibrated).

#### 4.1.1. PAINS analysis

Pan assay interference compounds (PAINS) are recognized for causing false positives in bioassays due to their nonspecific interactions. Using computational screening, we have confirmed that our newly designed molecules possess characteristics unrelated to PAINS.^66,71–73^

### 4.2 Chemical synthesis

#### 4.2.1. General procedure A: reductive amination

To a stirred solution of 3 (3-benzyloxybenzaldehyde; 1.0 eq) in methanol, selected primary amine (1.0 eq) was added. The mixture was stirred at room temperature (RT) for 24 h; then, the reaction was cooled to 0 °C and sodium borohydride (NaBH_4_; 0.9 eq.) was added. The reaction mixture was stirred for a further 3 h until the reaction was completed, as monitored by analytical TLC. The reaction mixture was evaporated to dryness and separated by preparative TLC to obtain 4–7, 13–18, 28–32, 35–42.

##### 4-[2-({[3-(Benzyloxy)phenyl]methyl}amino)ethyl]phenol (4)

Aldehyde 3 (80 mg; 0.377 mmol); tyramine (52 mg; 0.377 mmol); NaBH_4_ (13 mg; 0.339 mmol) in MeOH (5 mL). After evaporation, the residue was purified by preparative TLC using mobile phase toluene : cyclohexane : diethylamine, To/cHx/DEA (8 : 2 : 3) to get pure product 4 as a yellow amorphous solid. Yield 95%. ^1^H NMR (500 MHz, CDCl_3_) *δ*: 7.43–7.36 (m, 3H), 7.35–7.29 (m, 1H), 7.24–7.19 (m, 1H), 7.03–6.97 (m, 2H), 6.96–6.92 (m, 1H), 6.90–6.84 (m, 2H), 6.72–6.66 (m, 2H), 4.98 (s, 2H), 4.70 (bs, 1H), 3.80 (s, 2H), 2.89 (t, *J* = 6.9 Hz, 2H), 2.77 (t, *J* = 6.9 Hz, 2H). ^13^C NMR (126 MHz, CDCl_3_) *δ*: 159.0, 155.1, 140.3, 136.9, 130.3, 129.7, 129.5, 128.5, 127.9, 127.5, 120.9, 115.7, 114.6, 114.0, 69.8, 53.4, 50.0, 34.6. ESI-HRMS *m/z* calcd for C_22_H_24_NO_2_^+^ [M + H]^+^ 334.1802, found 334.1809; 99.82% purity.

##### {[3-(Benzyloxy)phenyl]methyl}[2-(2-methoxyphenyl)ethyl]amine (5)

Aldehyde 3 (80 mg; 0.377 mmol); 2-(2-methoxyphenyl)ethan-1-amine (55 μl; 0.377 mmol); NaBH_4_ (13 mg; 0.339 mmol) in MeOH (5 mL). After evaporation, the residue was purified by preparative TLC using mobile phase cHx/DEA (9 : 1.5) to get pure product 5 as a yellow oil. Yield 95%. ^1^H NMR (500 MHz, CDCl_3_) *δ*: 7.48–7.44 (m, 2H), 7.44–7.39 (m, 2H), 7.38–7.33 (m, 1H), 7.30–7.15 (m, 3H), 7.03–6.97 (m, 1H), 6.95–6.90 (m, 2H), 6.90–6.85 (m, 2H), 5.07 (s, 2H), 3.84 (s, 2H), 3.82 (s, 3H), 2.96–2.84 (m, 4H). ^13^C NMR (126 MHz, CDCl_3_) *δ*: 158.9, 157.6, 141.8, 137.1, 130.3, 129.3, 128.5, 128.2, 127.9, 127.5, 127.4, 120.7, 120.4, 114.4, 113.4, 110.3, 69.9, 55.2, 53.5, 48.9, 30.6. ESI-HRMS *m*/*z* calcd for C_23_H_26_NO_2_^+^ [M + H]^+^ 348.1958, found 348.1966; 98.24% purity.

##### {[3-(Benzyloxy)phenyl]methyl}[2-(3-methoxyphenyl)ethyl]amine (6)

Aldehyde 3 (80 mg; 0.377 mmol); 2-(3-methoxyphenyl)ethan-1-amine (55 μl; 0.377 mmol); NaBH_4_ (13 mg; 0.339 mmol) in MeOH (5 mL). After evaporation, the residue was purified by preparative TLC using mobile phase cHx/DEA (9 : 0.7) to get pure product 6 as a yellow oil. Yield 96%. ^1^H NMR (600 MHz, CDCl_3_) *δ*: 7.46–7.42 (m, 2H), 7.40–7.36 (m, 2H), 7.34–7.30 (m, 1H), 7.23–7.18 (m, 2H), 6.96–6.92 (m, 1H), 6.91–6.84 (m, 2H), 6.81–6.78 (m, 1H), 6.77–6.73 (m, 2H), 5.04 (s, 2H), 3.79 (s, 2H), 3.77 (s, 3H), 2.91–2.88 (m, 2H), 2.83–2.79 (m, 2H). ^13^C NMR (151 MHz, CDCl_3_) *δ*: 159.7, 159.0, 141.6, 141.5, 137.1, 129.4, 129.4, 128.5, 127.9, 127.5, 121.1, 120.7, 114.4, 113.5, 111.5, 69.9, 55.1, 53.6, 50.2, 36.2. ESI-HRMS *m*/*z* calcd for C_23_H_26_NO_2_^+^ [M + H]^+^ 348.1958, found 348.1964; 98.34% purity.

##### {[3-(Benzyloxy)phenyl]methyl}[2-(4-methoxyphenyl)ethyl]amine (7)

Aldehyde 3 (80 mg; 0.377 mmol); 2-(4-methoxyphenyl)ethan-1-amine (55 μl; 0.377 mmol); NaBH_4_ (13 mg; 0.339 mmol) in MeOH (5 mL). After evaporation, the residue was purified by preparative TLC using mobile phase cHx/DEA (9 : 0.7) to get pure product 7 as a yellow oil. Yield 100%. ^1^H NMR (600 MHz, CDCl_3_) *δ*: 7.45–7.41 (m, 2H), 7.40–7.36 (m, 2H), 7.34–7.30 (m, 1H), 7.24–7.20 (m, 1H), 7.13–7.09 (m, 2H), 6.96–6.92 (m, 1H), 6.90–6.80 (m, 4H), 5.04 (s, 2H), 3.78 (s, 2H), 3.77 (s, 3H), 2.86 (t, *J* = 7.4 Hz, 2H), 2.77 (t, *J* = 7.4 Hz, 2H). ^13^C NMR (151 MHz, CDCl_3_) *δ*: 159.0, 158.0, 141.9, 137.1, 132.0, 129.6, 129.4, 128.5, 127.9, 127.5, 120.7, 114.4, 113.9, 113.4, 69.9, 55.2, 53.7, 50.6, 35.3. ESI-HRMS *m*/*z* calcd for C_23_H_26_NO_2_^+^ [M + H]^+^ 348.1958, found 348.1963; 99.52% purity.

##### *N*-{[3-(Benzyloxy)phenyl]methyl}-3-chloroaniline (13)

Aldehyde 3 (80 mg; 0.377 mmol); 3-chloroaniline (40 μl; 0.377 mmol); NaBH_4_ (13 mg; 0.339 mmol) in MeOH (5 mL). After evaporation, the residue was purified by preparative TLC using mobile phase cHx/DEA (9 : 0.7) to get pure product 13 as a yellow oil. Yield 87%. ^1^H NMR (500 MHz, CDCl_3_) *δ*: 7.47–7.38 (m, 4H), 7.38–7.33 (m, 1H), 7.32–7.27 (m, 1H), 7.12–7.05 (m, 1H), 7.03–6.99 (m, 1H), 6.99–6.95 (m, 1H), 6.94–6.91 (m, 1H), 6.73–6.67 (m, 1H), 6.64–6.60 (m, 1H), 6.53–6.47 (m, 1H), 5.08 (s, 2H), 4.30 (s, 2H), 4.14 (bs, 1H). ^13^C NMR (126 MHz, CDCl_3_) *δ*: 159.1, 149.1, 140.5, 136.8, 135.0, 130.2, 129.8, 128.6, 128.0, 127.5, 119.9, 117.4, 113.9, 113.7, 112.5, 111.1, 70.0, 48.0. ESI-HRMS *m*/*z* calcd for C_20_H_19_ClNO^+^ [M + H]^+^ 324.1150, found 324.1153; 99.64% purity.

##### Benzyl({[3-(benzyloxy)phenyl]methyl})amine (14)

Aldehyde 3 (80 mg; 0.377 mmol); benzylamine (40 mg; 0.377 mmol); NaBH_4_ (13 mg; 0.339 mmol) in MeOH (5 mL). After evaporation, the residue was purified by preparative TLC using mobile phase cHx/DEA (9 : 0.7) to get pure product 14 as a yellow oil. Yield 96%. ^1^H NMR (500 MHz, CDCl_3_) *δ*: 7.49–7.45 (m, 2H), 7.44–7.39 (m, 2H), 7.38–7.33 (m, 5H), 7.31–7.25 (m, 2H), 7.06–7.02 (m, 1H), 7.01–6.94 (m, 1H), 6.93–6.87 (m, 1H), 5.10 (s, 2H), 3.83 (s, 2H), 3.82 (s, 2H), 2.02 (bs, 1H). ^13^C NMR (126 MHz, CDCl_3_) *δ*: 159.0, 141.9, 140.1, 137.1, 129.4, 128.5, 128.4, 128.2, 127.9, 127.5, 127.0, 120.7, 114.6, 113.4, 69.9, 53.0, 53.0. ESI-HRMS *m*/*z* calcd for C_21_H_22_NO^+^ [M + H]^+^ 304.1696, found 304.1700; 99.99% purity.

##### {[3-(Benzyloxy)phenyl]methyl}[(3,4-dimethoxyphenyl)methyl]amine (15)

Aldehyde 3 (80 mg; 0.377 mmol); 1-(3,4-dimethoxyphenyl)methanamine (57 μl; 0.377 mmol); NaBH_4_ (13 mg; 0.339 mmol) in MeOH (5 mL). After evaporation, the residue was purified by preparative TLC using mobile phase cHx/DEA (9 : 1.5) to get pure product 15 as a yellow oil. Yield 79%. ^1^H NMR (500 MHz, CDCl_3_) *δ*: 7.48–7.43 (m, 2H), 7.43–7.38 (m, 2H), 7.37–7.32 (m, 1H), 7.29–7.24 (m, 1H), 7.05–7.00 (m, 1H), 6.97–6.92 (m, 2H), 6.91–6.82 (m, 3H), 5.09 (s, 2H), 3.90 (s, 3H), 3.89 (s, 3H), 3.80 (s, 2H), 3.76 (s, 2H), 2.01 (bs, 1H). ^13^C NMR (126 MHz, CDCl_3_) *δ*: 159.0, 148.9, 148.0, 141.8, 137.0, 132.6, 129.4, 128.5, 127.9, 127.5, 120.7, 120.3, 114.6, 113.3, 111.4, 111.0, 69.9, 55.9, 55.8, 52.9, 52.7. ESI-HRMS *m*/*z* calcd for C_23_H_26_NO_3_^+^ [M + H]^+^ 364.1907, found 364.1910; 99.95% purity.

##### {[3-(Benzyloxy)phenyl]methyl}(2-phenylethyl)amine (16)

Aldehyde 3 (80 mg; 0.377 mmol); 2-phenylethan-1-amine (48 μl; 0.377 mmol); NaBH_4_ (13 mg; 0.339 mmol) in MeOH (5 mL). After evaporation, the residue was purified by preparative TLC using mobile phase cHx/DEA (9 : 0.7) to get pure product 16 as a yellow oil. Yield 93%. ^1^H NMR (500 MHz, CDCl_3_) *δ*: 7.49–7.44 (m, 2H), 7.44–7.38 (m, 2H), 7.37–7.30 (m, 3H), 7.28–7.21 (m, 4H), 7.00–6.96 (m, 1H), 6.94–6.86 (m, 2H), 5.07 (s, 2H), 3.82 (s, 2H), 2.97–2.83 (m, 4H), 2.27 (bs, 1H). ^13^C NMR (126 MHz, CDCl_3_) *δ*: 158.9, 141.4, 139.8, 137.0, 129.4, 128.7, 128.5, 128.4, 127.9, 127.5, 126.2, 120.7, 114.4, 113.5, 69.9, 53.6, 50.3, 36.1. ESI-HRMS *m*/*z* calcd for C_22_H_24_NO^+^ [M + H]^+^ 318.1852, found 318.1859; 99.47% purity.

##### {[3-(Benzyloxy)phenyl]methyl}(3-phenylpropyl)amine (17)

Aldehyde 3 (80 mg; 0.377 mmol); 3-phenylpropan-1-amine (54 μl; 0.377 mmol); NaBH_4_ (13 mg; 0.339 mmol) in MeOH (5 mL). After evaporation, the residue was purified by preparative TLC using mobile phase cHx/DEA (9 : 0.7) to get pure product 17 as a yellow oil. Yield 99%. ^1^H NMR (500 MHz, CDCl_3_) *δ*: 7.49–7.45 (m, 2H), 7.44–7.39 (m, 2H), 7.38–7.33 (m, 1H), 7.32–7.26 (m, 3H), 7.22–7.19 (m, 3H), 7.03–6.99 (m, 1H), 6.96–6.93 (m, 1H), 6.92–6.89 (m, 1H), 5.09 (s, 2H), 3.80 (s, 2H), 2.72–2.66 (m, 4H), 2.10 (bs, 1H), 1.92–1.83 (m, 2H). ^13^C NMR (126 MHz, CDCl_3_) *δ*: 158.9, 142.0, 141.7, 137.0, 129.4, 128.5, 128.4, 128.3, 127.9, 127.5, 125.8, 120.7, 114.5, 113.4, 69.9, 53.7, 48.7, 33.6, 31.5. ESI-HRMS *m*/*z* calcd for C_23_H_26_NO^+^ [M + H]^+^ 332.2009, found 332.2021; 99.44% purity.

##### {[3-(Benzyloxy)phenyl]methyl}(2-phenylpropyl)amine (18)

Aldehyde 3 (80 mg; 0.377 mmol); 2-phenylpropan-1-amine (55 μl; 0.377 mmol); NaBH_4_ (13 mg; 0.339 mmol) in MeOH (5 mL). After evaporation, the residue was purified by preparative TLC using mobile phase cHx/DEA (9 : 0.7) to get pure product 18 as a yellow oil. Yield 92%. [*α*]^24^_D_ = −38.3° (*c* = 0.105; MeOH); ^1^H NMR (500 MHz, CDCl_3_) *δ*: 7.48–7.44 (m, 2H), 7.44–7.38 (m, 2H), 7.38–7.31 (m, 3H), 7.25–7.21 (m, 4H), 6.94–6.90 (m, 1H), 6.89–6.84 (m, 2H), 5.06 (s, 2H), 3.79 (d, *J* = 13.4 Hz, 1H), 3.74 (d, *J* = 13.4 Hz, 1H), 3.04–2.94 (m, 1H), 2.86–2.76 (m, 2H), 1.67 (bs, 1H), 1.28 (d, *J* = 7.0 Hz, 3H). ^13^C NMR (126 MHz, CDCl_3_) *δ*: 158.9, 145.3, 142.0, 137.1, 129.3, 128.5, 127.9, 127.5, 127.2, 126.4, 120.6, 114.3, 113.3, 69.9, 56.2, 53.6, 40.0, 20.1. ESI-HRMS *m*/*z* calcd for C_23_H_26_NO^+^ [M + H]^+^ 332.2009, found 332.2014; 100% purity.

##### 4-[2-({[4-(Benzyloxy)phenyl]methyl}amino)ethyl]phenol (28)

Aldehyde 27 (100 mg; 0.471 mmol); tyramine (65 mg; 0.471 mmol); NaBH_4_ (16 mg; 0.424 mmol) in MeOH (5 mL). After evaporation, the residue was purified by preparative TLC using mobile phase To/cHx/DEA (8 : 2 : 4) to get pure product 28 as a yellow amorphous solid. Yield 34%. ^1^H NMR (500 MHz, CDCl_3_) *δ*: 7.46–7.37 (m, 4H), 7.37–7.31 (m, 1H), 7.22–7.17 (m, 2H), 7.02–6.97 (m, 2H), 6.95–6.89 (m, 2H), 6.70–6.65 (m, 2H), 5.04 (s, 2H), 4.71 (bs, 1H), 3.76 (s, 2H), 2.89 (t, *J* = 6.9 Hz, 2H), 2.77 (t, *J* = 6.9 Hz, 2H). ^13^C NMR (126 MHz, CDCl_3_) *δ*: 158.0, 155.1, 136.9, 131.3, 130.3, 129.7, 129.6, 128.6, 127.9, 127.4, 115.7, 114.8, 70.0, 52.9, 50.0, 34.6. ESI-HRMS *m*/*z* calcd for C_22_H_24_NO_2_^+^ [M + H]^+^ 334.1802, found 334.1798; 99.90% purity.

##### {[4-(Benzyloxy)phenyl]methyl}[2-(2-methoxyphenyl)ethyl]amine (29)

Aldehyde 27 (100 mg; 0.471 mmol); 2-(2-methoxyphenyl)ethan-1-amine (69 μl; 0.471 mmol); NaBH_4_ (16 mg; 0.424 mmol) in MeOH (5 mL). After evaporation, the residue was purified by preparative TLC using mobile phase cHx/DEA (9 : 1.5) to get pure product 29 as a yellow oil. Yield 99%. ^1^H NMR (600 MHz, CDCl_3_) *δ*: 7.45–7.41 (m, 2H), 7.40–7.36 (m, 2H), 7.34–7.29 (m, 1H), 7.23–7.17 (m, 3H), 7.16–7.13 (m, 1H), 6.95–6.81 (m, 4H), 5.05 (s, 2H), 3.79 (s, 3H), 3.75 (s, 2H), 2.90–2.81 (m, 4H) 1.44 (bs, 1H). ^13^C NMR (151 MHz, CDCl_3_) *δ*: 157.7, 157.6, 137.1, 133.0, 130.3, 129.3, 128.5, 128.5, 127.9, 127.4, 127.3, 120.4, 114.7, 110.3, 70.0, 55.2, 53.1, 49.1, 30.8. ESI-HRMS *m*/*z* calcd for C_23_H_26_NO_2_^+^ [M + H]^+^ 348.1958, found 348.1960; 99.87% purity.

##### {[4-(Benzyloxy)phenyl]methyl}[2-(3-methoxyphenyl)ethyl]amine (30)

Aldehyde 27 (100 mg; 0.471 mmol); 2-(3-methoxyphenyl)ethan-1-amine (69 μl; 0.471 mmol); NaBH_4_ (16 mg; 0.424 mmol) in MeOH (5 mL). After evaporation, the residue was purified by preparative TLC using mobile phase cHx/DEA (9 : 1.5) to get pure product 30 as a yellow oil. Yield 93%. ^1^H NMR (600 MHz, CDCl_3_) *δ*: 7.44–7.41 (m, 2H), 7.39–7.36 (m, 2H), 7.33–7.30 (m, 1H), 7.22–7.18 (m, 3H), 6.94–6.88 (m, 2H), 6.81–6.77 (m, 1H), 6.76–6.73 (m, 2H), 5.05 (s, 2H), 3.78 (s, 3H), 3.74 (s, 2H), 2.92–2.86 (m, 2H), 2.82–2.77 (m, 2H) 1.47 (bs, 1H). ^13^C NMR (151 MHz, CDCl_3_) *δ*: 159.7, 157.8, 141.7, 137.1, 132.7, 129.4, 129.3, 128.5, 127.9, 127.4, 121.1, 114.7, 114.4, 111.4, 70.0, 55.1, 53.2, 50.3, 36.4. ESI-HRMS *m*/*z* calcd for C_23_H_26_NO_2_^+^ [M + H]^+^ 348.1958, found 348.1960; 98.03% purity.

##### {[4-(Benzyloxy)phenyl]methyl}[2-(4-methoxyphenyl)ethyl]amine (31)

Aldehyde 27 (100 mg; 0.471 mmol); 2-(4-methoxyphenyl)ethan-1-amine (69 μl; 0.471 mmol); NaBH_4_ (16 mg; 0.424 mmol) in MeOH (5 mL). After evaporation, the residue was purified by preparative TLC using mobile phase cHx/DEA (9 : 1.5) to get pure product 31 as a yellow oil. Yield 97%. ^1^H NMR (600 MHz, CDCl_3_) *δ*: 7.44–7.41 (m, 2H), 7.40–7.34 (m, 2H), 7.34–7.29 (m, 1H), 7.22–7.18 (m, 2H), 7.14–7.08 (m, 2H), 6.96–6.89 (m, 2H), 6.85–6.80 (m, 2H), 5.04 (s, 2H), 3.78 (s, 3H), 3.73 (s, 2H), 2.85 (t, *J* = 7.2 Hz, 2H), 2.76 (t, *J* = 7.2 Hz, 2H), 1.73 (bs, 1H). ^13^C NMR (151 MHz, CDCl_3_) *δ*: 158.0, 157.8, 137.1, 132.6, 132.0, 129.6, 129.3, 128.5, 127.9, 127.4, 114.7, 113.9, 70.0, 55.2, 53.2, 50.6, 35.3. ESI-HRMS *m*/*z* calcd for C_23_H_26_NO_2_^+^ [M + H]^+^ 348.1958, found 348.1960; 98.66% purity.

##### {[4-(Benzyloxy)phenyl]methyl}[2-(4-chlorophenyl)ethyl]amine (32)

Aldehyde 27 (100 mg; 0.471 mmol); 2-(4-chlorophenyl)ethan-1-amine (66 μl; 0.471 mmol); NaBH_4_ (16 mg; 0.424 mmol) in MeOH (5 mL). After evaporation, the residue was purified by preparative TLC using mobile phase cHx/DEA (9 : 1.5) to get pure product 32 as a white amorphous solid. Yield 86%. ^1^H NMR (600 MHz, CDCl_3_) *δ*: 7.45–7.41 (m, 2H), 7.41–7.35 (m, 2H), 7.34–7.29 (m, 1H), 7.26–7.22 (m, 2H), 7.22–7.17 (m, 2H), 7.14–7.09 (m, 2H), 6.95–6.89 (m, 2H), 5.04 (s, 2H), 3.73 (s, 2H), 2.86 (t, *J* = 7.2 Hz, 2H), 2.78 (t, *J* = 7.2 Hz, 2H), 1.77 (bs, 1H). ^13^C NMR (151 MHz, CDCl_3_) *δ*: 157.9, 138.4, 137.1, 132.3, 131.9, 130.1, 129.3, 128.6, 128.5, 127.9, 127.4, 114.8, 70.0, 53.2, 50.2, 35.6. ESI-HRMS *m*/*z* calcd for C_22_H_23_ClNO^+^ [M + H]^+^ 352.1463, found 352.1465; 97.50% purity.

##### Benzyl({[4-(benzyloxy)phenyl]methyl})amine (35)

Aldehyde 27 (100 mg; 0.471 mmol); benzylamine (51 mg; 0.471 mmol); NaBH_4_ (16 mg; 0.424 mmol) in MeOH (5 mL). After evaporation, the residue was purified by preparative TLC using mobile phase cHx/DEA (9 : 1.5) to get pure product 35 as a yellow oil. Yield 88%. ^1^H NMR (600 MHz, CDCl_3_) *δ*: 7.46–7.41 (m, 2H), 7.41–7.37 (m, 2H), 7.37–7.30 (m, 5H), 7.29–7.23 (m, 3H), 6.97–6.92 (m, 2H), 5.06 (s, 2H), 3.80 (s, 2H), 3.75 (s, 2H) 1.75 (bs, 1H). ^13^C NMR (151 MHz, CDCl_3_) *δ*: 157.9, 140.3, 137.1, 132.7, 129.3, 128.5, 128.4, 128.1, 127.9, 127.4, 126.9, 114.8, 70.0, 53.0, 52.5. ESI-HRMS *m*/*z* calcd for C_21_H_22_NO^+^ [M + H]^+^ 304.1696, found 304.1701; 99.53% purity.

##### {[4-(Benzyloxy)phenyl]methyl}[(pyridin-2-yl)methyl]amine (36)

Aldehyde 27 (100 mg; 0.471 mmol); 1-(pyridin-2-yl)methanamine (46 μl; 0.471 mmol); NaBH_4_ (16 mg; 0.424 mmol) in MeOH (5 mL). After evaporation, the residue was purified by preparative TLC using mobile phase cHx/DEA (9 : 2.5) to get pure product 36 as a yellow oil. Yield 87%. ^1^H NMR (600 MHz, CDCl_3_) *δ*: 8.58–8.53 (m, 1H), 7.62 (td, *J* = 7.6 Hz, *J* = 1.4 Hz, 1H), 7.44–7.41 (m, 2H), 7.39–7.35 (m, 2H), 7.33–7.29 (m, 2H), 7.28–7.24 (m, 2H), 7.15 (ddd, *J* = 7.6 Hz, *J* = 4.9 Hz, *J* = 1.4 Hz, 1H), 6.98–6.90 (m, 2H), 5.05 (s, 2H), 3.91 (s, 2H), 3.78 (s, 2H), 2.37 (bs, 1H). ^13^C NMR (151 MHz, CDCl_3_) *δ*: 159.6, 157.9, 149.3, 137.1, 136.4, 132.4, 129.5, 128.5, 127.9, 127.4, 122.3, 121.9, 114.8, 70.0, 54.3, 52.8. ESI-HRMS *m*/*z* calcd for C_20_H_21_N_2_O^+^ [M + H]^+^ 305.1648, found 305.1654; 99.94% purity.

##### {[4-(Benzyloxy)phenyl]methyl}(2-phenylethyl)amine (37)

Aldehyde 27 (100 mg; 0.471 mmol); 2-phenylethan-1-amine (59 μl; 0.471 mmol); NaBH_4_ (16 mg; 0.424 mmol) in MeOH (5 mL). After evaporation, the residue was purified by preparative TLC using mobile phase cHx/DEA (9 : 1.5) to get pure product 37 as a yellow oil. Yield 83%. ^1^H NMR (600 MHz, CDCl_3_) *δ*: 7.44–7.41 (m, 2H), 7.40–7.36 (m, 2H), 7.35–7.27 (m, 3H), 7.23–7.18 (m, 5H), 6.95–6.89 (m, 2H), 5.04 (s, 2H), 3.74 (s, 2H), 2.90 (t, *J* = 7.3 Hz, 2H), 2.83 (t, *J* = 7.3 Hz, 2H), 1.80 (bs, 1H). ^13^C NMR (151 MHz, CDCl_3_) *δ*: 157.9, 140.0, 137.1, 132.5, 129.3, 128.7, 128.5, 128.4, 127.9, 127.4, 126.1, 114.7, 70.0, 53.2, 50.4, 36.2. ESI-HRMS *m*/*z* calcd for C_22_H_24_NO^+^ [M + H]^+^ 318.1852, found 318.1856; 99.85% purity.

##### {[4-(Benzyloxy)phenyl]methyl}[2-(pyridin-2-yl)ethyl]amine (38)

Aldehyde 27 (100 mg; 0.471 mmol); 2-(pyridin-2-yl)ethan-1-amine (56 μl; 0.471 mmol); NaBH_4_ (16 mg; 0.424 mmol) in MeOH (5 mL). After evaporation, the residue was purified by preparative TLC using mobile phase cHx/DEA (9 : 2.5) to get pure product 38 as a yellow oil. Yield 100%. ^1^H NMR (600 MHz, CDCl_3_) *δ*: 8.53–8.49 (m, 1H), 7.57 (td, *J* = 7.6 Hz, *J* = 1.9 Hz, 1H), 7.44–7.40 (m, 2H), 7.40–7.35 (m, 2H), 7.33–7.26 (m, 1H), 7.24–7.18 (m, 2H), 7.15 (dt, *J* = 7.6 Hz, *J* = 1.2 Hz, 1H), 7.10 (ddd, *J* = 7.6 Hz, *J* = 4.9 Hz, *J* = 1.2 Hz, 1H), 6.93–6.89 (m, 2H), 5.04 (s, 2H), 3.76 (s, 2H), 3.06–2.96 (m, 4H), 2.03 (bs, 1H). ^13^C NMR (151 MHz, CDCl_3_) *δ*: 160.3, 157.8, 149.3, 137.1, 136.3, 132.6, 129.3, 128.5, 127.9, 127.4, 123.3, 121.2, 114.7, 70.0, 53.2, 48.7, 38.3. ESI-HRMS *m*/*z* calcd for C_21_H_23_N_2_O^+^ [M + H]^+^ 319.1805, found 319.1812; 99.98% purity.

##### {[4-(Benzyloxy)phenyl]methyl}(3-phenylpropyl)amine (39)

Aldehyde 27 (100 mg; 0.471 mmol); 3-phenylpropan-1-amine (67 μl; 0.471 mmol); NaBH_4_ (16 mg; 0.424 mmol) in MeOH (5 mL). After evaporation, the residue was purified by preparative TLC using mobile phase ethyl acetate : cyclohexane : ammonium hydroxide 28% EtOAc/cHx/NH_4_OH (50 : 24 : 0.6) to get pure product 39 as a yellow oil. Yield 100%. ^1^H NMR (600 MHz, CDCl_3_) *δ*: 7.45–7.41 (m, 2H), 7.41–7.36 (m, 2H), 7.34–7.29 (m, 1H), 7.29–7.22 (m, 4H), 7.20–7.15 (m, 3H), 6.96–6.91 (m, 2H), 5.05 (s, 2H), 3.72 (s, 2H), 2.67 (t, overlap, *J* = 6.7 Hz, 2H), 2.66 (t, overlap, *J* = 6.7 Hz, 2H), 2.08 (bs, 1H), 1.89–1.81 (m, 2H). ^13^C NMR (151 MHz, CDCl_3_) *δ*: 157.9, 142.1, 137.1, 132.4, 129.4, 128.5, 128.4, 128.3, 127.9, 127.4, 125.8, 114.8, 70.0, 53.2, 48.6, 33.6, 31.5. ESI-HRMS *m*/*z* calcd for C_23_H_26_NO^+^ [M + H]^+^ 332.2009, found 332.2017; 99.58% purity.

##### {[4-(Benzyloxy)phenyl]methyl}(4-phenylbutyl)amine (40)

Aldehyde 27 (100 mg; 0.471 mmol); 4-phenylbutan-1-amine (75 μl; 0.471 mmol); NaBH_4_ (16 mg; 0.424 mmol) in MeOH (5 mL). After evaporation, the residue was purified by preparative TLC using mobile phase To/cHx/DEA (9 : 3 : 1) to get pure product 40 as a white amorphous solid. Yield 92%. ^1^H NMR (600 MHz, CDCl_3_) *δ*: 7.45–7.41 (m, 2H), 7.41–7.35 (m, 2H), 7.35–7.30 (m, 1H), 7.30–7.21 (m, 4H), 7.19–7.15 (m, 3H), 6.96–6.91 (m, 2H), 5.05 (s, 2H), 3.71 (s, 2H), 2.67–2.59 (m, 4H), 1.76 (bs, 1H), 1.70–1.62 (m, 2H), 1.60–1.52 (m, 2H). ^13^C NMR (151 MHz, CDCl_3_) *δ*: 157.8, 142.4, 137.1, 132.7, 129.3, 128.5, 128.4, 128.2, 127.9, 127.4, 125.7, 114.7, 70.0, 53.4, 49.1, 35.8, 29.6, 29.2. ESI-HRMS *m*/*z* calcd for C_24_H_28_NO^+^ [M + H]^+^ 346.2165, found 346.2166; 99.94% purity.

##### {[4-(Benzyloxy)phenyl]methyl}(2-phenylpropyl)amine (41)

Aldehyde 27 (100 mg; 0.471 mmol); 2-phenylpropan-1-amine (69 μl; 0.471 mmol); NaBH_4_ (16 mg; 0.424 mmol) in MeOH (5 mL). After evaporation, the residue was purified by preparative TLC using mobile phase cHx/DEA (9 : 0.7) to get pure product 41 as a yellow amorphous solid. Yield 83%. [*α*]^24^_D_ = −34.1° (*c* = 0.129; MeOH); ^1^H NMR (600 MHz, CDCl_3_) *δ*: 7.44–7.41 (m, 2H), 7.40–7.35 (m, 2H), 7.35–7.27 (m, 3H), 7.23–7.19 (m, 3H), 7.18–7.14 (m, 2H), 6.94–6.88 (m, 2H), 5.04 (s, 2H), 3.72 (d, *J* = 13.1 Hz, 1H), 3.68 (d, *J* = 13.1 Hz, 1H), 3.02–2.94 (m, 1H), 2.79 (d, overlap, *J* = 9.0 Hz, 1H), 2.78 (d, overlap, *J* = 5.7 Hz, 1H), 2.03 (bs, 1H), 1.26 (d, *J* = 6.9 Hz, 3H). ^13^C NMR (151 MHz, CDCl_3_) *δ*: 157.8, 145.2, 137.1, 132.3, 129.3, 128.5, 127.9, 127.4, 127.2, 126.4, 114.7, 70.0, 56.1, 53.0, 39.8, 20.1. ESI-HRMS *m*/*z* calcd for C_23_H_26_NO^+^ [M + H]^+^ 332.2009, found 332.2012; 99.98% purity.

##### {[4-(Benzyloxy)phenyl]methyl}(4-phenylbutan-2-yl)amine (42)

Aldehyde 27 (100 mg; 0.471 mmol); 4-phenylbutan-2-amine (76 μl; 0.471 mmol); NaBH_4_ (16 mg; 0.424 mmol) in MeOH (5 mL). After evaporation, the residue was purified by preparative TLC using mobile phase EtOAc/cHx/NH_4_OH (40 : 24 : 0.6) to get pure product 42 as a white amorphous solid. Yield 92%. [*α*]^24^_D_ = 30.5° (*c* = 0.131; MeOH); ^1^H NMR (600 MHz, CDCl_3_) *δ*: 7.45–7.41 (m, 2H), 7.40–7.36 (m, 2H), 7.34–7.30 (m, 1H), 7.28–7.21 (m, 4H), 7.19–7.15 (m, 3H), 6.96–6.90 (m, 2H), 5.05 (s, 2H), 3.77 (d, *J* = 12.8 Hz, 1H), 3.68 (d, *J* = 12.8 Hz, 1H), 2.78–2.71 (m, 1H), 2.71–2.60 (m, 2H), 1.87–1.80 (m, 1H), 1.72–1.65 (m, 1H), 1.15 (d, *J* = 6.3 Hz, 3H). ^13^C NMR (151 MHz, CDCl_3_) *δ*: 157.9, 142.3, 137.1, 132.4, 129.5, 128.6, 128.3, 127.9, 127.4, 125.7, 114.8, 70.0, 51.9, 50.4, 38.3, 32.2, 20.0. ESI-HRMS *m*/*z* calcd for C_24_H_28_NO^+^ [M + H]^+^ 346.2165, found 346.2171; 99.63% purity.

#### 4.2.2. Amine group protection

##### *tert*-Butyl *N*-{[3-(benzyloxy)phenyl]methyl}-*N*-[2-(4-hydroxyphenyl)ethyl]carbamate (10)

The corresponding secondary amine 4 (40 mg; 0.120 mmol) was dissolved in dry THF (3 mL). Triethylamine (34 μl, 0.240 mmol) was added followed by di-*tert*-butyl dicarbonate (39 mg, 0.180 mmol) and the mixture was stirred at room temperature overnight. After reaction completion as monitored by TLC, the reaction mixture was evaporated to dryness and separated by preparative TLC using mobile phase To/cHx/DEA (8 : 2 : 3) to get pure product 10 as a yellow oil. Yield 96%. The crude product was used directly for the next step.

#### 4.2.3. General procedure B: alkylation of compounds using NaH base

The corresponding secondary amine was dissolved in dry THF (2 mL). Sodium hydride (1.2 eq, 60% suspension in mineral oil) was added to the reaction mixture at 0 °C, stirred under argon, and after 30 min allyl bromide (1.3 eq.) was added. The reaction was stirred under argon for 24 h at RT. After the completion of the reaction as monitored by TLC, the mixture was evaporated to dryness and separated by preparative TLC to obtain 8, 9, 11, 20, 23, 26.

##### 4-[2-({[3-(Benzyloxy)phenyl]methyl}(prop-2-en-1-yl)amino)ethyl]phenol (8)

Compound 4 (36 mg; 0.234 mmol); NaH (5 mg; 0.130 mmol); allyl bromide (12 μl; 0.140 mmol) in dry THF (2 mL). After evaporation, the residue was purified by preparative TLC using mobile phase To/cHx/DEA (8 : 2 : 3) to get pure product 8 as a yellow oil. Yield 45%. ^1^H NMR (600 MHz, CDCl_3_) *δ*: 7.45–7.40 (m, 2H), 7.39–7.34 (m, 2H), 7.34–7.28 (m, 1H), 7.23–7.17 (m, 1H), 7.00–6.94 (m, 3H), 6.92–6.88 (m, 1H), 6.87–6.84 (m, 1H), 6.73–6.67 (m, 2H), 5.87 (ddt, *J* = 17.1 Hz, *J* = 10.2 Hz, *J* = 6.4 Hz, 1H), 5.19 (d, *J* = 17.1 Hz, 1H), 5.15 (d, *J* = 10.2 Hz, 1H), 5.01 (s, 2H), 3.64 (s, 2H), 3.48 (s, 1H), 3.16 (d, *J* = 6.4 Hz, 2H), 2.74–2.65 (m, 4H). ^13^C NMR (151 MHz, CDCl_3_) *δ*: 158.9, 154.0, 140.6, 137.1, 135.3, 132.2, 129.8, 129.2, 128.5, 127.9, 127.5, 121.6, 117.8, 115.2, 115.1, 113.6, 69.9, 57.9, 56.6, 55.2, 32.2. ESI-HRMS *m*/*z* calcd for C_25_H_28_NO_2_^+^ [M + H]^+^ 374.2115, found 374.2119; 99.91% purity.

##### {[3-(Benzyloxy)phenyl]methyl}(prop-2-en-1-yl){2-[4-(prop-2-en-1-yloxy)phenyl]ethyl}amine (9)

Compound 8 (15 mg; 0.040 mmol); NaH (2 mg; 0.048 mmol); allyl bromide (5 μl; 0.052 mmol) in dry THF (2 mL). After evaporation, the residue was purified by preparative TLC using mobile phase To : cHx : DEA (8 : 2 : 3) to get pure product 9 as a yellow oil. Yield 51%. ^1^H NMR (500 MHz, CDCl_3_) *δ*: 7.49–7.43 (m, 2H), 7.42–7.38 (m, 2H), 7.36–7.32 (m, 1H), 7.25–7.20 (m, 1H), 7.12–7.05 (m, 2H), 7.03–7.00 (m, 1H), 6.95–6.90 (m, 1H), 6.90–6.86 (m, 1H), 6.86–6.81 (m, 2H), 6.05 (ddt, *J* = 17.5 Hz, *J* = 10.6 Hz, *J* = 5.3 Hz, 1H), 5.95–5.84 (m, 1H), 5.41 (d, *J* = 17.5 Hz, 1H), 5.28 (d, *J* = 10.6 Hz, 1H), 5.21 (d, *J* = 16.8 Hz, 1H), 5.17 (d, *J* = 10.8 Hz, 1H), 5.05 (s, 2H), 4.52–4.49 (m, 2H), 3.66 (s, 2H), 3.18 (d, *J* = 6.4 Hz, 2H), 2.79–2.68 (m, 4H). ^13^C NMR (126 MHz, CDCl_3_) *δ*: 158.9, 156.8, 141.1, 137.1, 135.6, 133.4, 132.7, 129.6, 129.1, 128.7, 128.5, 127.9, 127.5, 121.4, 117.5, 115.0, 114.5, 113.5, 69.9, 68.8, 57.9, 56.7, 55.3, 32.5. ESI-HRMS *m*/*z* calcd for C_25_H_28_NO_2_^+^ [M + H]^+^ 414.2428, found 414.2432; 99.93% purity.

##### *tert*-Butyl *N*-{[3-(benzyloxy)phenyl]methyl}-*N*-{2-[4-(prop-2-en-1-yloxy)phenyl]ethyl}carbamate (11)

Compound 10 (25 mg; 0.058 mmol); NaH (3 mg; 0.070 mmol); allyl bromide (7 μl; 0.074 mmol) in dry THF (2 mL). After evaporation, the residue was purified by preparative TLC using mobile phase To/cHx/DEA (8 : 2 : 1) to get pure product 11 as a yellow oil. Yield 66%. ^1^H NMR (500 MHz, CDCl_3_) *δ*: 7.45–7.42 (m, 2H), 7.42–7.36 (m, 2H), 7.36–7.30 (m, 1H), 7.23 (t, *J* = 8.0 Hz, 1H), 7.10–6.99 (m, 2H), 6.90–6.76 (m, 5H), 6.06 (ddt, *J* = 17.3 Hz, *J* = 10.6 Hz, *J* = 5.3 Hz, 1H), 5.41 (dq, *J* = 17.3 Hz, *J* = 1.6 Hz, 1H), 5.29 (dq, *J* = 10.6 Hz, *J* = 1.6 Hz, 1H), 5.05 (s, 2H), 4.52 (dt, *J* = 5.3 Hz, *J* = 1.6 Hz, 2H), 4.40–4.27 (m, 2H), 3.41–3.25 (m, 2H), 2.81–2.65 (m, 2H), 1.50 (s, 9H).^13^C NMR (126 MHz, CDCl_3_) *δ*: 159.0, 157.1, 155.9, 140.1, 137.0, 133.4, 131.5, 129.7, 129.5, 128.6, 127.9, 127.5, 120.3, 117.5, 114.7, 114.1, 113.5, 79.6, 69.9, 68.8, 50.1, 48.6, 33.9, 28.4.

##### Benzyl({[3-(benzyloxy)phenyl]methyl})(prop-2-en-1-yl)amine (20)

Compound 14 (21 mg; 0.069 mmol); NaH (3 mg; 0.083 mmol); allyl bromide (8 μl; 0.090 mmol) in dry THF (2 mL). After evaporation, the residue was purified by preparative TLC using mobile phase cHx/DEA (9 : 0.5) to get pure product 20 as a yellow oil. Yield 66%. ^1^H NMR (500 MHz, CDCl_3_) *δ*: 7.49–7.45 (m, 2H), 7.44–7.30 (m, 7H), 7.27–7.23 (m, 2H), 7.10–7.05 (m, 1H), 7.01–6.95 (m, 1H), 6.90–6.84 (m, 1H), 5.92 (ddt, *J* = 16.9 Hz, *J* = 10.0 Hz, *J* = 6.2 Hz, 1H), 5.23 (d, *J* = 16.9 Hz, 1H), 5.17 (d, *J* = 10.0 Hz, 1H), 5.09 (s, 2H), 3.60 (s, 2H), 3.57 (s, 2H), 3.08 (d, *J* = 6.2 Hz, 2H). ^13^C NMR (126 MHz, CDCl_3_) *δ*: 158.9, 141.4, 139.6, 137.2, 135.9, 129.1, 128.7, 128.6, 128.2, 127.9, 127.5, 126.8, 121.3, 117.3, 115.1, 113.2, 69.9, 57.7, 57.7, 56.3. ESI-HRMS *m*/*z* calcd for C_24_H_26_NO^+^ [M + H]^+^ 344.2009, found 344.2013; 99.64% purity.

##### {[3-(Benzyloxy)phenyl]methyl}(2-phenylethyl)(prop-2-en-1-yl)amine (23)

Compound 16 (25 mg; 0.079 mmol); NaH (4 mg; 0.095 mmol); allyl bromide (9 μl; 0.103 mmol) in dry THF (2 mL). After evaporation, the residue was purified by preparative TLC using mobile phase cHx/DEA (9 : 0.5) to get pure product 23 as a yellow oil. Yield 67%. ^1^H NMR (600 MHz, CDCl_3_) *δ*: 7.46–7.42 (m, 2H), 7.40–7.36 (m, 2H), 7.33–7.30 (m, 1H), 7.28–7.23 (m, 2H), 7.22–7.12 (m, 4H), 7.01–6.97 (m, 1H), 6.92–6.88 (m, 1H), 6.87–6.84 (m, 1H), 5.87 (ddt, *J* = 16.9 Hz, *J* = 10.2 Hz, *J* = 6.2 Hz, 1H), 5.19 (d, *J* = 16.9 Hz, 1H), 5.14 (d, *J* = 10.2 Hz, 1H), 5.03 (s, 2H), 3.64 (s, 2H), 3.16 (d, *J* = 6.2 Hz, 2H), 2.82–2.70 (m, 4H). ^13^C NMR (151 MHz, CDCl_3_) *δ*: 158.9, 141.2, 140.6, 137.2, 135.7, 129.1, 128.8, 128.5, 128.2, 127.9, 127.5, 125.8, 121.4, 117.3, 115.0, 113.5, 69.9, 58.0, 56.7, 55.1, 33.4. ESI-HRMS *m*/*z* calcd for C_25_H_28_NO^+^ [M + H]^+^ 358.2165, found 358.2169; 99.97% purity.

##### {[3-(Benzyloxy)phenyl]methyl}(3-phenylpropyl)(prop-2-en-1-yl)amine (26)

Compound 17 (27 mg; 0.082 mmol); NaH (4 mg; 0.098 mmol); allyl bromide (9 μl; 0.107 mmol) in dry THF (2 mL). After evaporation, the residue was purified by preparative TLC using mobile phase cHx/DEA (9 : 0.5) to get pure product 26 as a yellow oil. Yield 69%. ^1^H NMR (500 MHz, CDCl_3_) *δ*: 7.51–7.44 (m, 2H), 7.42–7.38 (m, 2H), 7.37–7.32 (m, 1H), 7.29–7.22 (m, 3H), 7.21–7.15 (m, 3H), 7.08–7.03 (m, 1H), 6.98–6.92 (m, 1H), 6.92–6.86 (m, 1H), 5.89 (ddt, *J* = 17.3 Hz, *J* = 10.0 Hz, *J* = 6.4 Hz, 1H), 5.19 (d, *J* = 17.3 Hz, 1H), 5.14 (d, *J* = 10.0 Hz, 1H), 5.09 (s, 2H), 3.59 (s, 2H), 3.11 (d, *J* = 6.4 Hz, 2H), 2.67–2.60 (m, 2H), 2.56–2.49 (m, 2H), 1.88–1.79 (m, 2H). ^13^C NMR (126 MHz, CDCl_3_) *δ*: 158.9, 142.5, 137.2, 135.8, 129.1, 128.5, 128.4, 128.2, 127.9, 127.5, 125.6, 121.5, 117.3, 117.2, 115.2, 113.3, 69.9, 58.0, 56.7, 53.0, 33.6, 28.9. ESI-HRMS *m*/*z* calcd for C_26_H_30_NO^+^ [M + H]^+^ 372.2322, found 372.2328; 99.89% purity.

#### 4.2.4. Deprotection of the amino group

##### {[3-(Benzyloxy)phenyl]methyl}({2-[4-(prop-2-en-1-yloxy)phenyl]ethyl})amine (12)

The corresponding Boc-protected amino intermediate 11 (17 mg; 0.036 mmol) was dissolved in DCM (1 mL). The reaction mixture was cooled to 0 °C and treated with TFA (100 μl). The reaction mixture was allowed to warm to RT and then stirred for 3 h. After reaction completion as monitored by TLC, the reaction mixture was evaporated to dryness and separated by preparative TLC using mobile phase To/cHx/DEA (8 : 2 : 1) to get pure product 12 as a yellow oil. Yield 88%. ^1^H NMR (500 MHz, CDCl_3_) *δ*: 7.46–7.42 (m, 2H), 7.42–7.36 (m, 2H), 7.36–7.31 (m, 1H), 7.24 (t, *J* = 8.0 Hz, 1H), 7.13–7.09 (m, 2H), 6.99 (bs, 1H), 6.92 (d, *J* = 8.0 Hz, 1H), 6.88 (dd, *J* = 8.0 Hz, *J* = 2.5 Hz, 1H), 6.86–6.83 (m, 2H), 6.05 (ddt, *J* = 17.3 Hz, *J* = 10.6 Hz, *J* = 5.3 Hz, 1H), 5.41 (dq, *J* = 17.3 Hz, *J* = 1.6 Hz, 1H), 5.29 (dq, *J* = 10.6 Hz, *J* = 1.6 Hz, 1H), 5.06 (s, 2H), 4.51 (dt, *J* = 5.3 Hz, *J* = 1.6 Hz, 2H), 3.83 (s, 2H), 2.92–2.86 (m, 2H), 2.85–2.78 (m, 2H). ^13^C NMR (126 MHz, CDCl_3_) *δ*: 159.0, 157.1, 137.0, 133.4, 131.6, 129.6, 129.5, 128.5, 128.3, 127.9, 127.5, 120.9, 117.6, 114.7, 114.6, 113.9, 69.9, 68.8, 53.2, 50.1, 34.7. ESI-HRMS *m*/*z* calcd for C_25_H_28_NO_2_^+^ [M + H]^+^ 374.2115, found 374.2115; 99.65% purity.

#### 4.2.5. General procedure C: alkylation of compounds using K_2_CO_3_ base

The corresponding secondary amine was dissolved in dry CH_3_CN (2 mL). Potassium carbonate (2.0 eq.) was added followed by potassium iodide if required (catalytic amount), and the corresponding alkylating agent (2.0 eq.). The reaction was left stirring under reflux for 24 h. After the completion of the reaction as monitored by TLC, a precipitate was formed during the reaction which was removed by filtration and the filtrate was evaporated to dryness and separated by preparative TLC to obtain 19, 21, 22, 24, 25, 33, 34, 43–47.

##### Benzyl({[3-(benzyloxy)phenyl]methyl})ethylamine (19)

Compound 14 (25 mg; 0.082 mmol); K_2_CO_3_ (23 mg; 0.164 mmol); ethyl iodide (13 μl; 0.164 mmol) in dry CH_3_CN (2 mL). After filtration and evaporation, the residue was purified by preparative TLC using mobile phase cHx/DEA (9 : 0.5) to get pure product 19 as a yellow oil. Yield 80%. ^1^H NMR (500 MHz, CDCl_3_) *δ*: 7.50–7.45 (m, 2H), 7.44–7.30 (m, 7H), 7.29–7.21 (m, 2H), 7.11–7.07 (m, 1H), 7.02–6.97 (m, 1H), 6.91–6.85 (m, 1H), 5.09 (s, 2H), 3.60 (s, 2H), 3.58 (s, 2H), 2.58–2.49 (m, 2H), 1.09 (t, *J* = 7.1 Hz, 3H). ^13^C NMR (126 MHz, CDCl_3_) *δ*: 158.8, 141.6, 139.7, 137.1, 129.1, 128.7, 128.5, 128.1, 127.9, 127.5, 126.8, 121.3, 115.1, 113.2, 69.9, 57.7, 57.6, 47.1, 11.8. ESI-HRMS *m*/*z* calcd for C_23_H_26_NO^+^ [M + H]^+^ 332.2009, found 332.2014; 99.55% purity.

##### {[3-(Benzyloxy)phenyl]methyl}(ethyl)(2-phenylethyl)amine (21)

Compound 16 (31 mg; 0.098 mmol); K_2_CO_3_ (27 mg; 0.196 mmol); ethyl iodide (16 μl; 0.196 mmol) in dry CH_3_CN (2 mL). After filtration and evaporation, the residue was purified by preparative TLC using mobile phase cHx/DEA (9 : 0.5) to get pure product 21 as a yellow oil. Yield 73%. ^1^H NMR (600 MHz, CDCl_3_) *δ*: 7.48–7.43 (m, 2H), 7.41–7.36 (m, 2H), 7.34–7.30 (m, 1H), 7.29–7.25 (m, 2H), 7.23–7.14 (m, 4H), 7.04–7.00 (m, 1H), 6.95–6.90 (m, 1H), 6.88–6.87 (m, 1H), 5.04 (s, 2H), 3.66 (s, 2H), 2.83–2.72 (m, 4H), 2.65–2.59 (m, 2H), 1.07 (t, *J* = 7.1 Hz, 3H). ^13^C NMR (151 MHz, CDCl_3_) *δ*: 158.9, 140.6, 137.2, 129.1, 128.8, 128.5, 128.3, 127.9, 127.5, 125.9, 121.4, 115.1, 113.5, 69.9, 57.9, 54.9, 47.3, 33.4, 11.7. ESI-HRMS *m*/*z* calcd for C_24_H_28_NO^+^ [M + H]^+^ 346.2165, found 346.2171; 99.89% purity.

##### {[3-(Benzyloxy)phenyl]methyl}(2-phenylethyl)propylamine (22)

Compound 16 (29 mg; 0.081 mmol); K_2_CO_3_ (22 mg; 0.162 mmol); KI cat.; *n*-propyl bromide (15 μl; 0.162 mmol) in dry CH_3_CN (2 mL). After filtration and evaporation, the residue was purified by preparative TLC using mobile phase cHx/DEA (9 : 0.5) to get pure product 22 as a yellow oil. Yield 25%. ^1^H NMR (500 MHz, CDCl_3_) *δ*: 7.48–7.44 (m, 2H), 7.43–7.39 (m, 2H), 7.36–7.32 (m, 1H), 7.30–7.26 (m, 2H), 7.25–7.15 (m, 4H), 7.05–7.00 (m, 1H), 6.96–6.90 (m, 1H), 6.90–6.86 (m, 1H), 5.05 (s, 2H), 3.65 (s, 2H), 2.84–2.70 (m, 4H), 2.52–2.46 (m, 2H), 1.57–1.46 (m, 2H), 0.88 (t, *J* = 7.4 Hz, 3H). ^13^C NMR (126 MHz, CDCl_3_) *δ*: 158.9, 137.2, 129.0, 128.8, 128.5, 128.2, 127.9, 127.5, 125.8, 121.3, 114.9, 113.4, 69.9, 58.5, 55.8, 55.6, 33.5, 20.2, 11.9. ESI-HRMS *m*/*z* calcd for C_25_H_30_NO^+^ [M + H]^+^ 360.2322, found 360.2326; 99.27% purity.

##### {[3-(Benzyloxy)phenyl]methyl}(ethyl)(3-phenylpropyl)amine (24)

Compound 17 (30 mg; 0.091 mmol); K_2_CO_3_ (25 mg; 0.182 mmol); ethyl iodide (15 μl; 0.182 mmol) in dry CH_3_CN (2 mL). After filtration and evaporation, the residue was purified by preparative TLC using mobile phase cHx/DEA (9 : 0.5) to get pure product 24 as a yellow oil. Yield 63%. ^1^H NMR (600 MHz, CDCl_3_) *δ*: 7.47–7.43 (m, 2H), 7.40–7.36 (m, 2H), 7.34–7.29 (m, 1H), 7.28–7.24 (m, 2H), 7.23–7.20 (m, 1H), 7.18–7.15 (m, 3H), 7.05–7.02 (m, 1H), 6.96–6.91 (m, 1H), 6.89–6.84 (m, 1H), 5.07 (s, 2H), 3.56 (s, 2H), 2.65–2.59 (m, 2H), 2.56–2.47 (m, 4H), 1.85–1.77 (m, 2H), 1.02 (t, *J* = 7.1 Hz, 3H). ^13^C NMR (151 MHz, CDCl_3_) *δ*: 158.9, 142.5, 137.2, 129.1, 128.5, 128.4, 128.3, 127.9, 127.5, 125.6, 121.5, 115.2, 113.2, 69.9, 58.0, 52.7, 47.3, 33.6, 28.8, 11.7. ESI-HRMS *m*/*z* calcd for C_25_H_30_NO^+^ [M + H]^+^ 360.2322, found 360.2321; 99.92% purity.

##### {[3-(Benzyloxy)phenyl]methyl}(3-phenylpropyl)propylamine (25)

Compound 17 (24 mg; 0.072 mmol); K_2_CO_3_ (20 mg; 0.144 mmol); KI cat.; *n*-propyl bromide (13 μl; 0.144 mmol) in dry CH_3_CN (2 mL). After filtration and evaporation, the residue was purified by preparative TLC using mobile phase cHx/DEA (9 : 0.5) to get pure product 25 as a yellow oil. Yield 95%. ^1^H NMR (600 MHz, CDCl_3_) *δ*: 7.46–7.43 (m, 2H), 7.40–7.36 (m, 2H), 7.33–7.30 (m, 1H), 7.28–7.24 (m, 2H), 7.24–7.20 (m, 1H), 7.19–7.14 (m, 3H), 7.06–7.02 (m, 1H), 6.96–6.91 (m, 1H), 6.89–6.84 (m, 1H), 5.07 (s, 2H), 3.56 (s, 2H), 2.64–2.58 (m, 2H), 2.51–2.46 (m, 2H), 2.42–2.37 (m, 2H), 1.83–1.75 (m, 2H), 1.52–1.43 (m, 2H), 0.87 (t, *J* = 7.3 Hz, 3H). ^13^C NMR (151 MHz, CDCl_3_) *δ*: 158.8, 142.5, 137.2, 129.1, 128.5, 128.4, 128.2, 127.9, 127.5, 125.6, 121.4, 115.1, 113.3, 69.9, 58.6, 55.8, 53.4, 33.6, 28.9, 20.1, 11.9. ESI-HRMS *m*/*z* calcd for C_26_H_32_NO^+^ [M + H]^+^ 374.2478, found 374.2483; 99.90% purity.

##### 4-[2-({[4-(Benzyloxy)phenyl]methyl}(propyl)amino)ethyl]phenol (33)

Compound 28 (28 mg; 0.084 mmol); K_2_CO_3_ (23 mg; 0.168 mmol); KI cat.; *n*-propyl bromide (15 μl; 0.168 mmol) in dry CH_3_CN (2 mL). After filtration and evaporation, the residue was purified by preparative TLC using mobile phase To/cHx/DEA (8 : 2 : 1) to get pure product 33 as a yellow oil. Yield 82%. ^1^H NMR (600 MHz, CDCl_3_) *δ*: 7.45–7.41 (m, 2H), 7.39–7.36 (m, 2H), 7.34–7.29 (m, 1H), 7.26–7.22 (m, 2H), 6.95–6.88 (m, 4H), 6.73–6.69 (m, 2H), 5.04 (s, 2H), 3.69 (s, 2H), 2.72 (s, 4H), 2.56–2.50 (m, 2H), 1.60–1.50 (m, 2H), 0.86 (t, *J* = 7.3 Hz, 3H). ^13^C NMR (151 MHz, CDCl_3_) *δ*: 158.2, 154.6, 137.0, 131.3, 130.5, 129.7, 128.6, 127.9, 127.5, 115.4, 114.7, 70.0, 57.3, 55.1, 54.9, 31.7, 19.4, 11.7. ESI-HRMS *m*/*z* calcd for C_25_H_30_NO_2_^+^ [M + H]^+^ 376.2271, found 376.2270; 98.03% purity.

##### {[4-(Benzyloxy)phenyl]methyl}[2-(4-propoxyphenyl)ethyl]propylamine (34)

Compound 33 (30 mg; 0.080 mmol); K_2_CO_3_ (22 mg; 0.160 mmol); KI cat.; *n*-propyl bromide (15 μl; 0.160 mmol) in dry CH_3_CN (2 mL). After filtration and evaporation, the residue was purified by preparative TLC using mobile phase To/cHx/DEA (8 : 2 : 1) to get pure product 34 as a yellow oil. Yield 57%. ^1^H NMR (600 MHz, CDCl_3_) *δ*: 7.45–7.41 (m, 2H), 7.40–7.36 (m, 2H), 7.34–7.28 (m, 1H), 7.24–7.18 (m, 2H), 7.05–7.00 (m, 2H), 6.93–6.87 (m, 2H), 6.81–6.75 (m, 2H), 5.05 (s, 2H), 3.90–3.84 (m, 2H), 3.59–3.56 (m, 2H), 2.71–2.61 (m, 4H), 2.47–2.41 (m, 2H), 1.82–1.73 (m, 2H), 1.54–1.44 (m, 2H), 1.01 (t, *J* = 7.4 Hz, 3H), 0.85 (t, *J* = 7.4 Hz, 3H). ^13^C NMR (151 MHz, CDCl_3_) *δ*: 157.7, 157.4, 137.2, 132.7, 129.9, 129.6, 128.5, 127.9, 127.5, 114.5, 114.3, 70.0, 69.5, 57.8, 55.7, 55.6, 32.5, 22.6, 20.2, 11.9, 10.5. ESI-HRMS *m*/*z* calcd for C_28_H_36_NO_2_^+^ [M + H]^+^ 418.2741, found 418.2740; 99.99% purity.

##### Benzyl({[4-(benzyloxy)phenyl]methyl})ethylamine (43)

Compound 35 (30 mg; 0.099 mmol); K_2_CO_3_ (27 mg; 0.198 mmol); ethyl iodide (16 μl; 0.198 mmol) in dry CH_3_CN (2 mL). After filtration and evaporation, the residue was purified by preparative TLC cHx/DEA (9 : 1.5) to get pure product 43 as a yellow oil. Yield 96%. ^1^H NMR (600 MHz, CDCl_3_) *δ*: 7.45–7.42 (m, 2H), 7.40–7.35 (m, 4H), 7.34–7.26 (m, 6H), 7.25–7.21 (m, 1H), 6.96–6.89 (m, 2H), 5.05 (s, 2H), 3.56 (s, 2H), 3.52 (s, 2H), 2.50 (q, *J* = 7.1 Hz, 2H), 1.06 (t, *J* = 7.1 Hz, 3H). ^13^C NMR (151 MHz, CDCl_3_) *δ*: 157.8, 140.0, 137.2, 132.2, 129.9, 128.7, 128.6, 128.1, 127.9, 127.5, 126.7, 114.5, 70.0, 57.5, 57.0, 46.9, 11.8. ESI-HRMS *m*/*z* calcd for C_23_H_26_NO^+^ [M + H]^+^ 332.2009, found 332.2018; 98.67% purity.

##### Benzyl({[4-(benzyloxy)phenyl]methyl})propylamine (44)

Compound 35 (55 mg; 0.181 mmol); K_2_CO_3_ (50 mg; 0.362 mmol); KI cat.; *n*-propyl bromide (33 μl; 0.362 mmol) in dry CH_3_CN (2 mL). After filtration and evaporation, the residue was purified by preparative TLC using mobile phase cHx/DEA (9 : 1.5) to get pure product 44 as a yellow oil. Yield 61%. ^1^H NMR (600 MHz, CDCl_3_) *δ*: 7.45–7.41 (m, 2H), 7.41–7.34 (m, 4H), 7.34–7.26 (m, 5H), 7.24–7.20 (m, 1H), 6.94–6.90 (m, 2H), 5.05 (s, 2H), 3.55 (s, 2H), 3.51 (s, 2H), 2.40–2.35 (m, 2H), 1.57–1.48 (m, 2H), 0.85 (t, *J* = 7.4 Hz, 3H). ^13^C NMR (151 MHz, CDCl_3_) *δ*: 157.8, 139.9, 137.2, 132.0, 129.9, 128.8, 128.5, 128.1, 127.9, 127.5, 126.7, 114.5, 70.0, 58.1, 57.5, 55.2, 20.1, 11.8. ESI-HRMS *m*/*z* calcd for C_24_H_28_NO^+^ [M + H]^+^ 346.2165, found 346.2170; 99.98% purity.

##### {[4-(Benzyloxy)phenyl]methyl}(3-phenylpropyl)propylamine (45)

Compound 39 (32 mg; 0.097 mmol); K_2_CO_3_ (27 mg; 0.194 mmol); KI cat.; *n*-propyl bromide (18 μl; 0.194 mmol) in dry CH_3_CN (2 mL). After filtration and evaporation, the residue was purified by preparative TLC using mobile phase EtOAc/cHx/NH_4_OH (50 : 24 : 0.6) to get pure product 45 as a yellow oil. Yield 69%. ^1^H NMR (600 MHz, CDCl_3_) *δ*: 7.46–7.43 (m, 2H), 7.41–7.36 (m, 2H), 7.35–7.29 (m, 1H), 7.27–7.21 (m, 4H), 7.18–7.12 (m, 3H), 6.94–6.89 (m, 2H), 5.06 (s, 2H), 3.50 (s, 2H), 2.59 (t, *J* = 7.5 Hz, 2H), 2.45 (t, *J* = 7.5 Hz, 2H), 2.37 (t, *J* = 7.5 Hz, 2H), 1.82–1.74 (m, 2H), 1.52–1.42 (m, 2H), 0.86 (t, *J* = 7.4 Hz, 3H). ^13^C NMR (151 MHz, CDCl_3_) *δ*: 157.7, 142.6, 137.2, 132.3, 130.0, 128.6, 128.4, 128.2, 127.9, 127.5, 125.6, 114.5, 70.0, 57.9, 55.7, 53.1, 33.7, 28.9, 20.1, 11.9. ESI-HRMS *m*/*z* calcd for C_26_H_32_NO^+^ [M + H]^+^ 374.2478, found 374.2484; 99.99% purity.

##### {[4-(Benzyloxy)phenyl]methyl}(ethyl)(4-phenylbutan-2-yl)amine (46)

Compound 42 (32 mg; 0.093 mmol); K_2_CO_3_ (25 mg; 0.186 mmol); ethyl iodide (15 μl; 0.186 mmol) in dry CH_3_CN (2 mL). After filtration and evaporation, the residue was purified by preparative TLC using mobile phase EtOAc/cHx/NH_4_OH (40 : 24 : 0.6) to get pure product 46 as a yellow oil. Yield 55%. [*α*]^24^_D_ = 29.1° (*c* = 0.110; MeOH); ^1^H NMR (600 MHz, CDCl_3_) *δ*: 7.48–7.42 (m, 2H), 7.40–7.36 (m, 2H), 7.35–7.29 (m, 1H), 7.29–7.22 (m, 4H), 7.18–7.12 (m, 3H), 6.95–6.89 (m, 2H), 5.06 (s, 2H), 3.66 (d, *J* = 13.9 Hz, 1H), 3.35 (d, *J* = 13.9 Hz, 1H), 2.84–2.72 (m, 2H), 2.61–2.48 (m, 2H), 2.42–2.34 (m, 1H), 1.88–1.78 (m, 1H), 1.59–1.47 (m, 1H), 1.04–0.95 (m, 6H). ^13^C NMR (151 MHz, CDCl_3_) *δ*: 157.6, 143.1, 137.3, 133.7, 129.6, 128.5, 128.4, 128.2, 127.9, 127.5, 125.5, 114.4, 70.1, 53.2, 52.8, 43.1, 36.2, 33.3, 14.3, 13.7. ESI-HRMS *m*/*z* calcd for C_26_H_32_NO^+^ [M + H]^+^ 374.2478, found 374.2477; 99.99% purity.

##### {[4-(Benzyloxy)phenyl]methyl}(4-phenylbutan-2-yl)propylamine (47)

Compound 42 (30 mg; 0.087 mmol); K_2_CO_3_ (24 mg; 0.174 mmol); KI cat.; *n*-propyl bromide (18 μl; 0.174 mmol) in dry CH_3_CN (2 mL). After filtration and evaporation, the residue was purified by preparative TLC using mobile phase EtOAc/cHx/NH_4_OH (24 : 80 : 0.6) to get pure product 47 as a yellow oil. Yield 47%. [*α*]^24^_D_ = 26.5° (*c* = 0.106; MeOH); ^1^H NMR (600 MHz, CDCl_3_) *δ*: 7.47–7.43 (m, 2H), 7.41–7.36 (m, 2H), 7.34–7.30 (m, 1H), 7.29–7.22 (m, 4H), 7.18–7.11 (m, 3H), 6.95–6.88 (m, 2H), 5.06 (s, 2H), 3.65 (d, *J* = 13.8 Hz, 1H), 3.36 (d, *J* = 13.8 Hz, 1H), 2.80–2.72 (m, 2H), 2.58–2.50 (m, 1H), 2.44–2.29 (m, 2H), 1.88–1.78 (m, 1H), 1.57–1.48 (m, 1H), 1.47–1.37 (m, 2H), 0.98 (d, *J* = 6.6 Hz, 3H), 0.86 (t, *J* = 7.4 Hz, 3H). ^13^C NMR (151 MHz, CDCl_3_) *δ*: 157.6, 143.1, 137.3, 133.6, 129.7, 128.5, 128.4, 128.2, 127.9, 127.5, 125.5, 114.4, 70.1, 53.5, 53.4, 51.1, 36.1, 33.4, 21.8, 13.6, 12.0. ESI-HRMS *m*/*z* calcd for C_27_H_34_NO^+^ [M + H]^+^ 388.2635, found 388.2638; 99.99% purity.

### 4.3 *In vitro h*AChE and *h*BChE inhibition assay

The activities of *h*AChE and *h*BChE were determined using a modified Ellman's method, as described in ref. 56 and 62 against recombinant AChE (*h*AChE, E.C. 3.1.1.7) and recombinant BChE (*h*BChE, E.C. 3.1.1.8), both of which were purchased from Sigma-Aldrich, Prague, Czech Republic. The results are expressed as IC_50_ (the concentration of the compound required to reduce 50% of cholinesterase activity). The other compounds used, including the phosphate buffer solution (PBS, pH = 7.4), 5,5′-dithio-bis(2-nitrobenzoic) acid (Ellman's reagent, DTNB), acetylthiocholine (ATChI), and butyrylthiocholine (BTChI), were commercially available and purchased from Sigma-Aldrich in Prague, Czech Republic. In brief, the corresponding enzyme (8.3 μL), 5 mM DTNB (283 μL), and the sample dilution in dimethyl sulfoxide (DMSO) at various concentrations (8.3 μL) were added to 96-well microplates (BRAND GMBH + CO KG, Wertheim, Germany) and pre-incubated for 5 minutes in the assay medium. The reaction was initiated by adding 10 mM substrate (ATChI or BTChI, 33.3 μL). The increase in absorbance (Δ*A*) was measured for 1 minute at 412 nm using a spectrophotometer (Synergy™ HT Multi-Detection Microplate Reader). Each measurement was repeated in triplicate. The percentage of inhibition was calculated using the following formula:
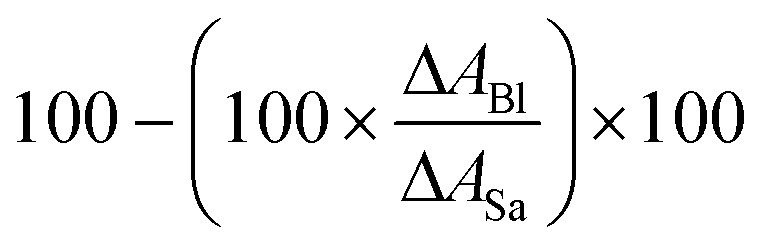
where Δ*A*_Bl_ represents the increase in absorbance of the blank sample, and Δ*A*_Sa_ represents the increase in absorbance of the measured sample. The *I*_inhibition_ potency of the tested compounds was expressed as an IC_50_ value (the concentration of the inhibitor that causes 50% cholinesterase inhibition). Microsoft Excel (Redmond, WA, USA) and GraphPad Prism version 7 for Windows (GraphPad Software, San Diego, CA, USA) were used for the statistical data evaluation.

### 4.4 Kinetic study of cholinesterase inhibition

The kinetic study of *h*BChE was conducted using the modified Ellman's method mentioned earlier,^62,74,75^ with slight variations applied at the collaborating laboratory. Solutions of the corresponding ChE in phosphate buffer (PB) were prepared to achieve a final activity of 0.002 U μL^−1^. The solutions of the tested compounds (8.3 μL at varying concentrations) were pre-incubated for 5 minutes in the assay medium. Subsequently, a solution of the substrate (33.3 μL of 0.01, 0.005, 0.0025, and 0.00125 M BTChI iodide solution) was added to initiate the reaction. The increase in absorbance was measured for 1 minute at 412 nm using the Multimode microplate reader Synergy 2 (BioTek Inc., Winooski, VT, USA). Nunc flat-bottomed polystyrene 96-well microplates (ThermoFisher Scientific, USA) were used for these measurements. To calculate the resulting measured activity (expressed as the percentage of inhibition I), the following formula was applied:
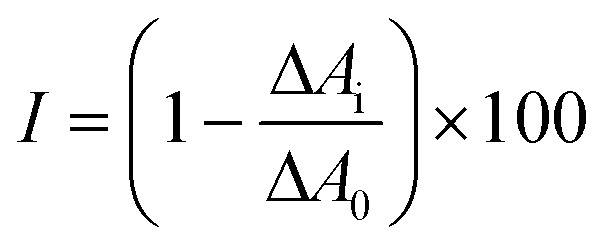
where Δ*A*_i_ indicates the absorbance change provided by adequate enzyme exposed to the corresponding inhibitor and Δ*A*_0_ indicates the absorbance change when a solution of PB was added instead of a solution of the inhibitor. Microsoft Excel (Redmond, WA, USA) and GraphPad Prism version 8.30 for Windows (GraphPad Software, San Diego, CA, USA) were employed for the statistical data analysis. The values of *V*_max_ and *K*_m_ for the Michaelis–Menten kinetics, as well as the values of *K*_i_ (enzyme–inhibitor dissociation constant) and *K*_i′_ (enzyme–substrate–inhibitor dissociation constant), were calculated through nonlinear regression from the substrate velocity curves. Linear regression was used for the calculation of Lineweaver–Burk plots. All calculations were carried out using GraphPad Prism software version 10.1.0 for Windows (San Diego, CA, USA).

### 4.5 PAMPA assay

PAMPA (the parallel artificial membrane permeability assay) is a high-throughput screening tool applicable for prediction of the passive transport of potential drugs across the BBB.^65,76,77^ In this study, it has been used as a non-cell-based *in vitro* assay carried out in a coated 96-well membrane filter. The filter membrane of the donor plate was coated with PBL (polar brain lipid, Avanti, USA) in dodecane (4 μl of 20 mg ml^−1^ PBL in dodecane) and the acceptor well was filled with 300 μl of PBS (pH 7.4; *V*_A_). The tested compounds were dissolved first in DMSO and subsequently diluted with PBS (pH 7.4) to final concentrations of 50–100 μM in the donor wells. The concentration of DMSO did not exceed 0.5% (v/v) in the donor solution. About 300 μL of the donor solution was added to the donor wells (*V*_D_) and the donor filter plate was carefully put on the acceptor plate so that the coated membrane was “in touch” with both the donor solution and acceptor buffer. In principle, the test compound diffused from the donor well through the lipid membrane (area = 0.28 cm^2^) to the acceptor well. The concentration of the drug in both the donor and acceptor wells was assessed after 3, 4, 5, and 6 h of incubation in quadruplicate using the UV plate reader Spark (Tecan Group Ltd, Switzerland) at the maximum absorption wavelength of each compound. Besides that, a solution of theoretical compound concentration, simulating the equilibrium state established if the membrane were ideally permeable, was prepared and assessed as well. The concentration of the compounds in the donor and acceptor wells and equilibrium concentration were calculated from the standard curve and expressed as the permeability (Pe) according the equation:
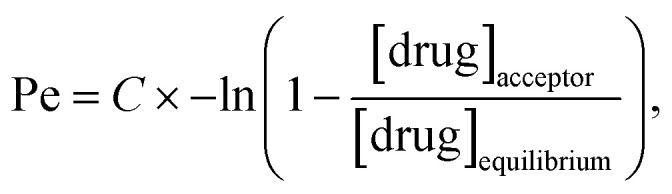
where
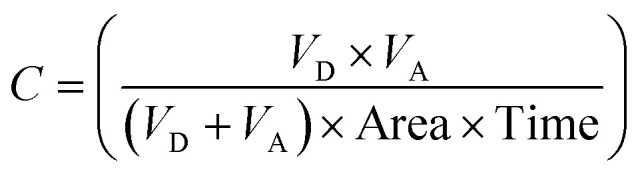


### 4.6 Cytotoxicity assay

#### 4.6.1. Human neuroblastoma cell line

The cytotoxicity of the tested compounds was assessed using SH-SY5Y cells (ECACC, 94030304) and the MTT assay (3-(4,5-dimethylthiazol-2-yl)-2,5-diphenyl-tetrazolium bromide, Sigma-Aldrich, St. Louis, MO).^78^ The cells were cultured following ECACC recommended conditions and seeded into 96-well plates at a density of 8000 cells per well, with 100 μL of medium per well. The tested compounds were dissolved in either DMSO (Sigma Aldrich) or phosphate-buffered saline (PBS, Sigma Aldrich) and subsequently in the growth medium – high glucose Dulbecco's modified Eagle's medium (DMEM; Sigma-Aldrich, D6429). The final concentration of DMSO did not exceed 1% (v/v). The cells were exposed to the tested compounds for 24 hours. Following exposure, the medium was replaced with a solution containing 10 μM MTT, and the cells were allowed to produce formazan for approximately 3 hours while being monitored.^79^ Afterward, the medium was aspirated, and the purple crystals of MTT formazan were dissolved in 100 μL of DMSO with shaking. Cell viability was determined spectrophotometrically by measuring the amount of formazan produced, with the absorbance measured at 570 nm with a 650 nm reference wavelength using a Spark spectrophotometer (Tecan Group Ltd, Switzerland). The IC_50_ value was then calculated from control-subtracted triplicates using non-linear regression (four parameters) with GraphPad Prism 9 software. The final IC_50_ and SEM values were obtained as the means of three independent measurements.

#### 4.6.2. Human hepatocellular carcinoma cell line

Human hepatocellular carcinoma HepG2 cells (ATCC HB-8065; passage 20–25), purchased from Health Protection Agency Culture Collections (ECACC, Salisbury, UK), were cultured in minimum essential Eagle medium supplemented with 10% v/v fetal bovine serum and 1% v/v l-glutamine solution (Sigma-Aldrich, St. Louis, MO, USA) at 37 °C in a humidified atmosphere containing 5% CO_2_. For passaging, the cells were treated with trypsin/EDTA (Sigma-Aldrich, St. Louis, USA) at 37 °C and then harvested. For cytotoxicity evaluation, the cells treated with the studied substances were used, while untreated HepG2 cells served as the control group. The cells were seeded in a 96-well plate at a density of 5 × 10^4^ cells per well and incubated for 24 hours. All tested compounds were dissolved in DMSO to prepare 10 mM stock solutions and subsequently diluted to the desired concentration just before the cell treatment (the highest DMSO concentration used was 0.5% v/v). Positive and negative controls were included, as well, and the activity of all the samples was determined in triplicate. Plates were incubated for 24 h in a humidified atmosphere containing 5% CO_2_ at 37 °C. After the incubation, a solution of thiazolyl blue tetrazolium bromide (Sigma-Aldrich, St. Louis, MO, USA) in RPMI 1640 without phenol red (BioTech) was added and incubated for 30 minutes in a humidified atmosphere containing 5% CO_2_ at 37 °C. Afterwards, the formazan crystals were dissolved in DMSO, and the absorbance of samples was recorded at 570 nm (BioTek, Synergy Neo2 Multi-Mode Reader NEO2SMALPHAB; BioTek, Winooski, VT, USA). IC_50_ values were calculated by nonlinear regression from a semi-logarithmic plot of incubation concentration *versus* percentage of absorbance relative to untreated controls using GraphPad Prism software (version 9; GraphPad Software, Inc., La Jolla, CA, USA).

### 4.7 *In silico* studies

Molecular docking was used for binding pose calculations. The 3D structure of ligands was built by OpenBabel, v.3.1.0 (ref. 80) and optimized by Avogadro, v.1.2.0 using the general Amber force-field.^81^ The ligands were converted into pdbqt-format by OpenBabel, v.3.1.0. The human BChE was gained from the RCSB database (PDB ID: 6QAA, resolution 1.90 Å) and prepared for docking by the function DockPrep of the software Chimera, v.1.16 (ref. 82) and by MGLTools, v.1.5.7.^83^ The docking calculation was made by Vina, v.1.2.3 as semi-flexible with flexible ligands and a rigid receptor.^84^

The docking pose of ligands was then improved by molecular dynamics (MD) simulation. The receptor structure was prepared using the software Chimera. The best-scored docking poses were taken as the starting point for MD. The force-field parameters for ligands were assessed by Antechamber^85^ v.20.0 using general Amber force-field 2.^86^ MD simulation was carried out by Gromacs, v.2018.1.^87^ The receptor–ligand complex was solvated in the periodic water box using the TIP3P model. The system was neutralized by adding Na^+^ and Cl^−^ ions to the concentration of 10 nM. The system energy was minimalized and equilibrated in a 100-ps isothermal–isochoric NVT and then a 100-ps isothermal–isobaric NPT phase. Then, a 10-ns MD simulation was run at a temperature of 300 K. The molecular docking and MD results were 3D visualized by the PyMOL Molecular Graphics System, version 2.5.2, Schrödinger, LLC.

### 4.8 Aqueous solubility determination

The water solubility of the tested compound was determined in ultrapure distilled water containing 5% DMSO. Briefly, 200 μl of the aqueous solution of compound 33 at the concentration of 50 μM was analyzed for the UV/vis absorption spectrum using a multimode microplate reader (TECAN Spark®, Tecan Group Ltd., Switzerland) and the wavelength of maximum absorbance was used for further testing of the corresponding compound. Subsequently, six consecutive concentrations were prepared for the compound by serial dilution of 50 μM standard solution and the absorbance of these solutions (200 μl) was measured by a microplate reader. The data were used to calculate the calibration curve by the linear regression method. Finally, the over-saturated solutions of the tested compound were prepared and incubated in an ultrasound water bath at 37 °C for 10 min. Then the solutions were centrifuged at 8000 RPM for 10 min and the UV/vis absorption of the supernatant was measured by a microplate reader. The obtained absorption value was used to calculate the maximum water solubility of the tested compound. In case the absorption value of the supernatant was higher than the calibration curve range, the supernatant was diluted with a 5% DMSO/water solution to match the calibration curve.

### 4.9 HLM and plasma stability determination

#### 4.9.1. HLM incubation

The tested compound 33 was incubated with human liver microsomes according to the Cyprotex assay protocol.^88^ Briefly, compounds were dissolved in DMSO to produce stock sample solutions. 5 μL of stock solution was mixed with 12.5 μL of pooled human liver microsomes (concentration 0.5 mg mL^−1^, H2620, LOT no. 1310528, SekiSui, XenoTech, Canada) and 458 μL of 0.1 M potassium phosphate buffer solution (pH = 7.4, adjusted by addition of KOH) and preincubated for 5 min (300 rpm, 37 °C). The final concentration of DMSO in the incubation mixture did not exceed 0.5% (v/v) and the concentration of tested compounds was set at 3 μM. The biotransformation reaction was started by addition of 25 μL of RapidStart NADPH Regenerating System (K5000, LOT. 1910008, SekiSui, XenoTech, Canada) and then incubated (400 rpm, 37 °C) for 5 different time points (0, 5, 15, 30, 45 min). The reaction was terminated by addition of 500 μL of cooled acetonitrile (−20 °C) with 1 μM internal standard [IS, compound 67 (ref. 89)] and centrifuged for 5 min (14 000 rpm, 20 °C). Subsequently, 400 μL of supernatant was transferred to the vial and analyzed by LC-MS. Three types of blank samples were prepared in the same way, with a one-step exception. In the biological blank sample, 5 μL of DMSO was added instead of 5 μL stock solution, in the chemical blanks, 50 μL of water was added instead of HLM, and in the case of the control blank sample, 25 μL of water was added instead of 25 μL of RapidStart System.

#### 4.9.2. Human plasma incubation

Compound 33 was incubated with human pooled plasma (Batch S00G71, Biowest, France). Briefly, compounds were dissolved in DMSO to produce stock sample solutions. 10 μL of stock solution was added to 990 μL of human plasma to initiate the reaction. The final concentration of DMSO in the incubation mixture did not exceed 0.5% (v/v) and the concentration of tested compounds was set at 1 μM. The reactions were stopped by transferring 200 μL of incubate to 200 μL of acetonitrile containing the internal standard (IS; 67) at appropriate time points (0, 15, 30, 60 and 120 min) and centrifuged at 12 000 rpm for 5 min at 4 °C to precipitate the protein. After that, 400 μL of supernatant was transferred to the vial and analyzed by LC-MS.

#### 4.9.3. HPLC-MS analysis

The HPLC system used in this study was Dionex Ultimate 3000 UHPLC RS consisting of an RS pump, RS column compartment, RS autosampler and diode array detector controlled by Chromeleon (version 7.2.9. build 11323) software (Thermo Fisher Scientific, Germering, Germany) connected to a Q Exactive Plus Orbitrap mass spectrometer with Thermo Xcalibur (version 3.1.66.10.) software (Thermo Fisher Scientific, Bremen, Germany). A Reverse-phase C18 column Kinetex EVO (Phenomenex, Torrance, CA, USA) was used as a stationary phase, and purified water with 0.1% formic acid (mobile phase A) and LC-MS grade acetonitrile with 0.1% formic acid (mobile phase B) were used as the mobile phases. Gradient elution was used to determine purities and mass spectra. The method started with 5% B for 0.3 min, then the gradient switched to 100% B in the third min, remained at 100% B for 0.7 min and then went back to 5% B with equilibration for 3.5 min. The total run time of the method was 7.5 min. The column temperature was kept constant at 27 °C, the flow of the mobile phase was 0.5 mL min^−1^, and the injection volume was 1 μL. Detection was performed by a UV detector (*λ* = 254 nm) and by mass spectrometry in positive mode. HRMS spectra were collected from the total ion current in the scan range 105–1000 *m*/*z*, with the resolution set to 140 000. Settings of the heated electrospray source were: spray voltage 3.5 kV; capillary temperature 220 °C; sheath gas 55 arbitrary units; auxiliary gas 15 arbitrary units; spare gas 3 arbitrary units; probe heater temperature 220 °C; max spray current 100 mA and S-lens RF Level 50. Solvents and other common chemicals were purchased from VWR (Stribrna Skalice, Czech Republic). Solvents for chromatographic procedures were supplied in LC-MS grade.

#### 4.9.4. Determination of *T*½ value, CL_int_, and plasma stability

The peak areas of the compounds (*A*_cmp_) and internal standards (*A*_IS_) were detected in positive mode in extracted ion chromatograms from the mass spectrometer data. For the determination of microsomal stability, the *A*_cmp_/*A*_IS_ was calculated as logarithmized. From a plot of ln *A*_cmp_/*A*_IS_ against incubation time, the gradient (*k* value) was established. The *T*½ value and CL_int_ were calculated according to the Cyprotex protocol and compared with known fast and slow metabolically degraded verapamil and diazepam standards.

For the determination of plasma stability, the percentage of the remaining compound was calculated from the ratio between peak area ratios (compound peak area/internal standard peak area) obtained at incubation times 0 and 120 min.

## List of abbreviations

AAs: Amaryllidaceae alkaloids
ACh: Acetylcholine
AChE: Acetylcholinesterase
AD: Alzheimer's disease
Aβ: Amyloid-β
BBB: blood–brain barrier
BChE: Butyrylcholinesterase
Boc_2_O: di-*tert*-Butyl dicarbonate
CH_3_CN: Acetonitrile
ChE: Cholinesterase
cHx: Cyclohexane
CL_int_: Intrinsic clearance
CNS: Central nervous system
DCM: Dichloromethane
DEA: Diethylamine
EtOAc: Ethyl acetate
*h*AChE: Human acetylcholinesterase
*h*BChE: Human butyrylcholinesterase
HLM: Human liver microsomes
K_2_CO_3_: Potassium carbonate
KI: Potassium iodide
LLE: Liquid–liquid extraction
MeOH: Methanol
NaBH_4_: Sodium borohydride
NaH: Sodium hydride
NFTs: Neurofibrillary tangles
NH_4_OH: Ammonium hydroxide
NPs: Natural products
PAMPA: Parallel artificial membrane permeability assay
RT: Room temperature
SAR: Structure–activity relationship
SEM: Standard error of mean
TEA: Triethylamine
TFA: Trifluoroacetic acid
THF: Tetrahydrofuran
TLC: Thin-layer chromatography
To: Toluene

## Author contributions

Filip Pidany – investigation, data collection, writing original draft, visualization; Jana Kroustkova – data analysis, data collection; Jaroslav Jenco – data analysis, data collection; Katerina Hradiska Breiterova – data analysis, data collection; Lubica Muckova – data analysis, data collection; Lucie Nováková – data analysis, data collection; Jiri Kunes – data analysis, data collection; Jakub Fibigar – data analysis, data collection, visualization; Tomas Kucera – data analysis, data collection, visualization; Martin Novak – data analysis, data collection; Ales Sorf – data analysis, data collection; Martina Hrabinova – data analysis, data collection; Lenka Pulkrabkova – data analysis, data collection; Jiri Janousek – data analysis, data collection; Ondrej Soukup – supervision, visualization, writing – review & editing, funding acquisition; Daniel Jun – visualization, funding acquisition; Jan Korabecny – supervision, visualization, writing – review & editing, funding acquisition; Lucie Cahlikova – supervision, visualization, writing – review & editing, funding acquisition.

## Conflicts of interest

The authors declare no conflict of interests.

## Supplementary Material

MD-015-D4MD00060A-s001
