# Supplementary material for: Carltonine-derived compounds for targeted butyrylcholinesterase inhibition
Source: RSC Med Chem. 2024 Mar 22;15(5):1601–25. doi: 10.1039/d4md00060a (PMC11110763; doi:10.1039/d4md00060a)

Electronic Supplementary Material (ESI) for RSC Medicinal Chemistry.
This journal is © The Royal Society of Chemistry 2024

Carltonine-Derived Compounds for Targeted Butyrylcholinesterase Inhibition

Filip Pidany[a], Jana Kroustkova[a], Jaroslav Jenco[a], Katerina Hradiska Breiterova[a], Lubica Muckova[b,c], Lucie Nováková[d], Jiri Kunes[e], Jakub Fibigar[c], Tomas Kucera[c], Martin Novak[b], Ales Sorf[c], Martina Hrabinova[b,c], Lenka Pulkrabkova[b,c], Jiri Janousek[b], Ondrej Soukup[b,c], Daniel Jun[c], Jan Korabecny[b,c]*, Lucie Cahlikova[a,]*

[a] *Charles University, Faculty of Pharmacy in Hradec Kralove, Department of Pharmacognosy and Pharmaceutical Botany, Akademika Heyrovskeho 1203, 500 05 Hradec Kralove, Czech Republic*

[b] *University Hospital Hradec Kralove, Biomedical Research Center, Sokolska 581, 500 05 Hradec Kralove, Czech Republic*

[c] *University of Defence, Military Faculty of Medicine, Department of Toxicology and Military Pharmacy, Trebesska 1575, 500 01 Hradec Kralove, Czech Republic*

[d] *Charles University, Faculty of Pharmacy in Hradec Kralove, Department of Analytical Chemistry, Akademika Heyrovskeho 1203, 500 05 Hradec Kralove, Czech Republic*

[e] *Charles University, Faculty of Pharmacy in Hradec Kralove, Department of Bioorganic and Organic Chemistry, Akademika Heyrovskeho 1203, 500 05 Hradec Kralove, Czech Republic*

* corresponding authors: Jan Korabecny, ORCID ID: 0000-0001-6977-7596, e-mail: jan.korabecny@fnhk.cz;

Lucie Cahlikova, ORCID ID: 0000-0002-1555-8870, e-mail: cahlikova@faf.cuni.cz

^{1}H NMR, ^{13}C NMR, ESI-HRMS spectra and PDA chromatogram for final compounds

4-[2-({[3-(Benzyloxy)phenyl]methyl}amino)ethyl]phenol (4):

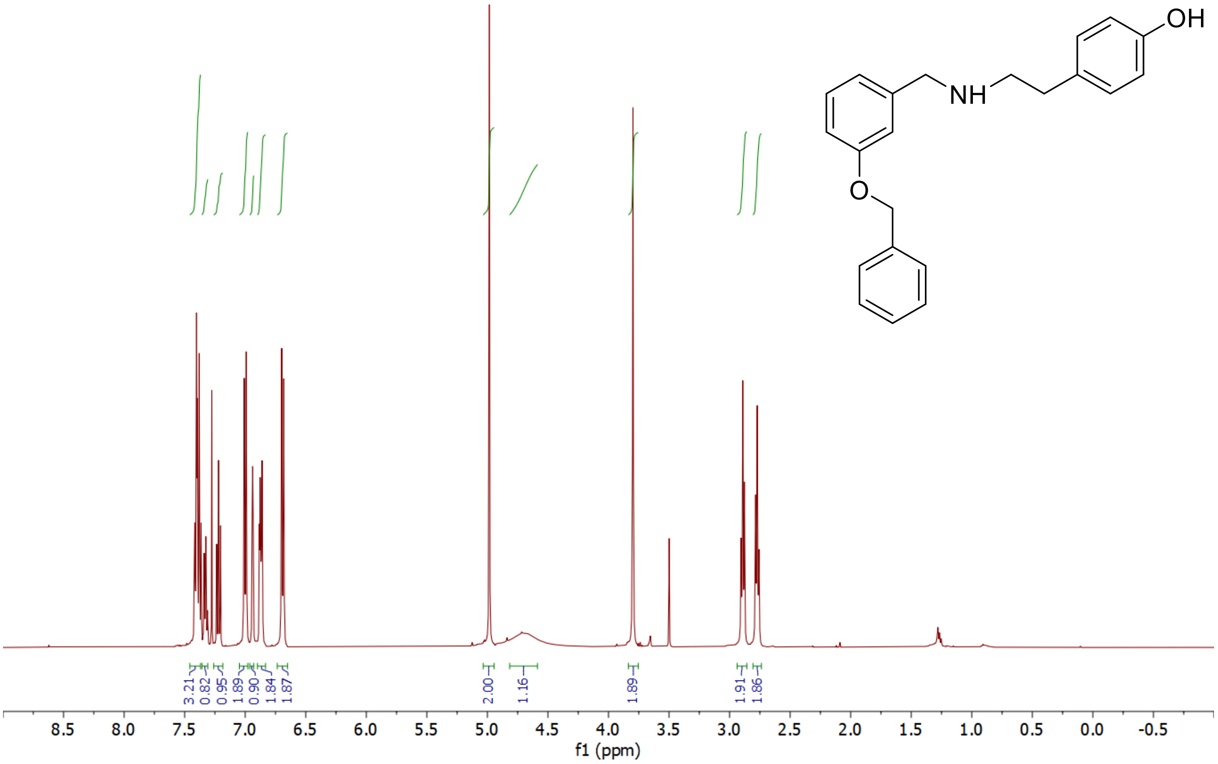

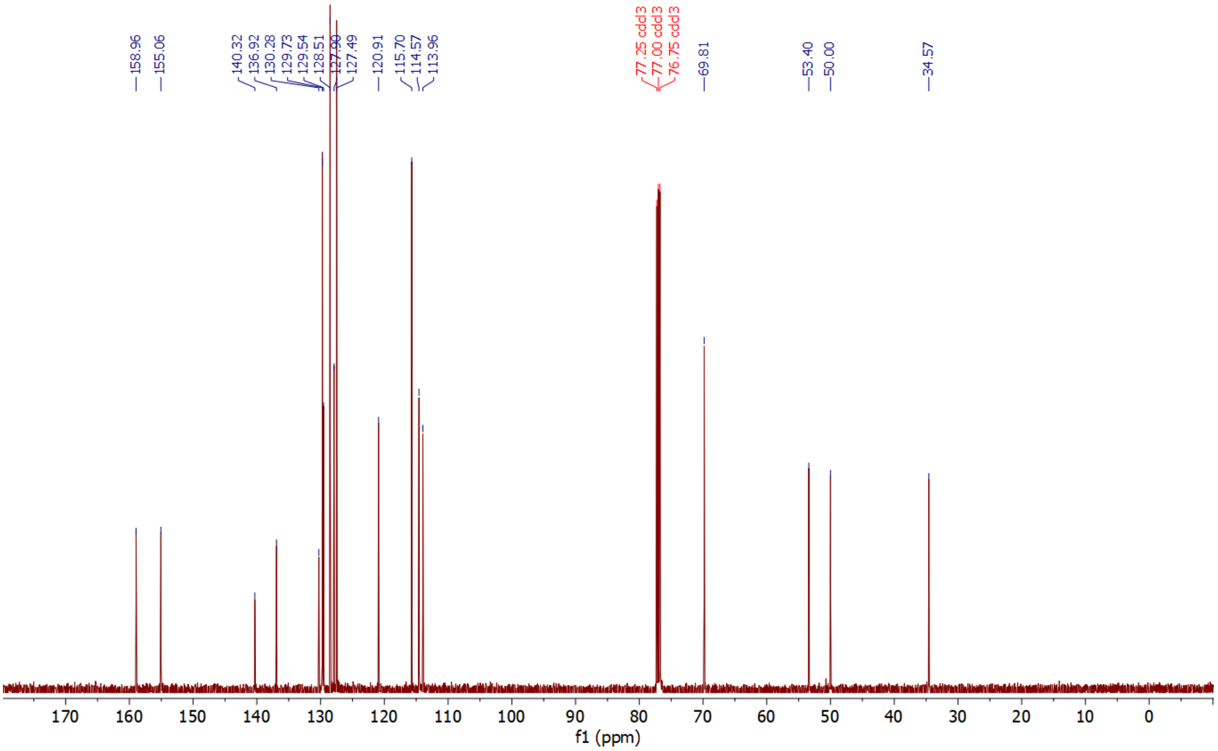

334.1802

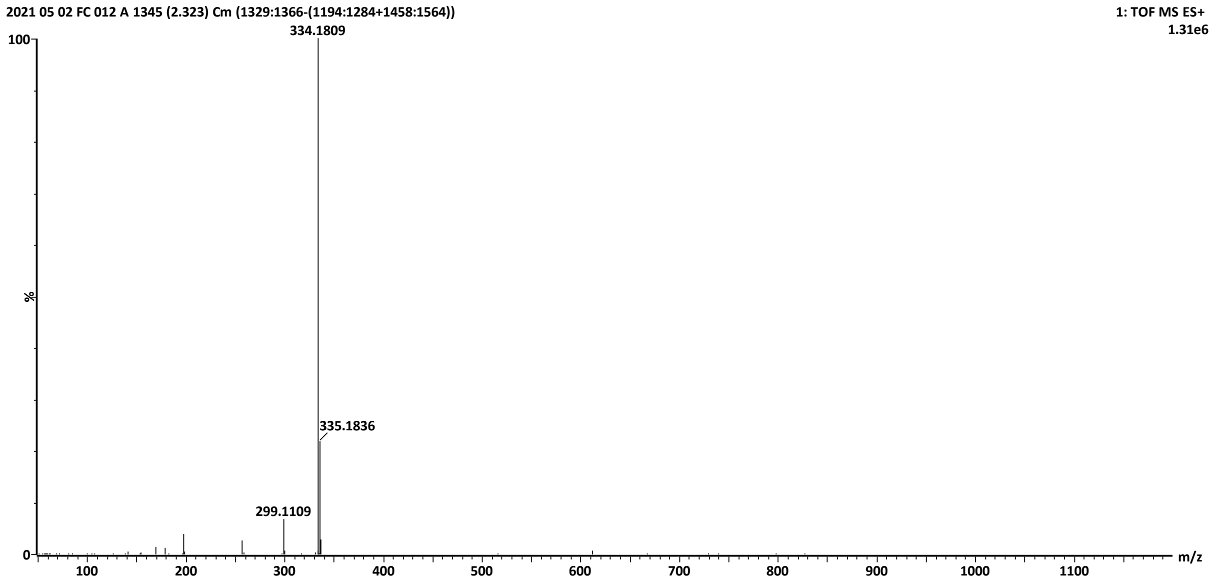
2021 05 02 FC 012 A 1345 (2.323) Cm (1329:1366-(1194:1284+1458:1564))
1: TOF MS ES+
1.31e6
334.1809
335.1836
299.1109
%
m/z

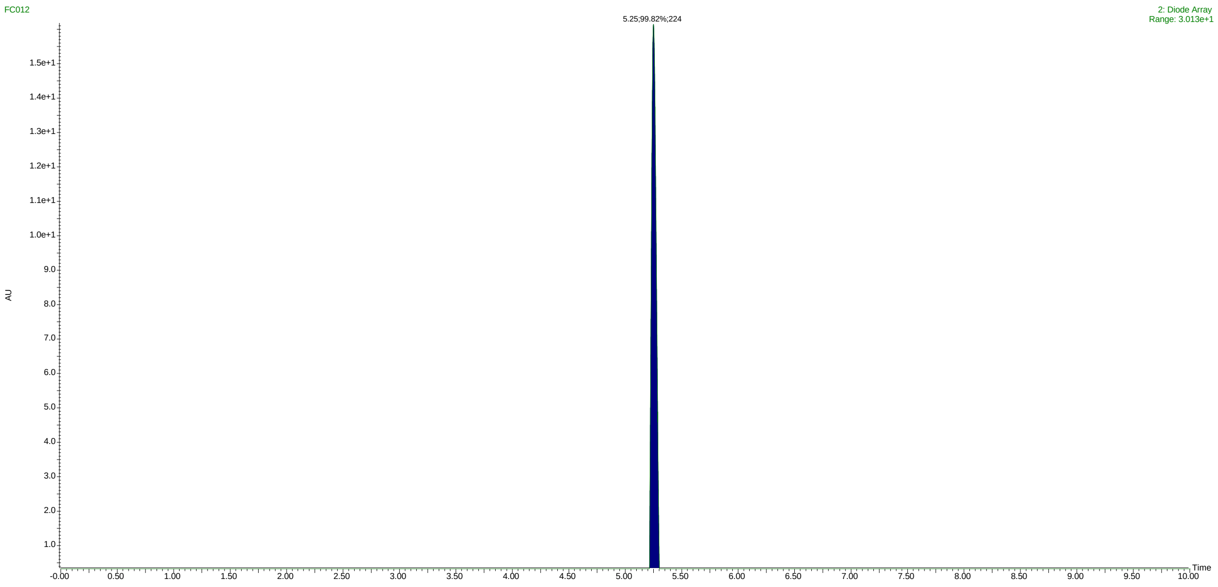
FC012
2: Diode Array
Range: 3.013e+1
5.25;99.82%;224
AU
Time

{[3-(Benzyloxy)phenyl]methyl}[2-(2-methoxyphenyl)ethyl]amine (5):

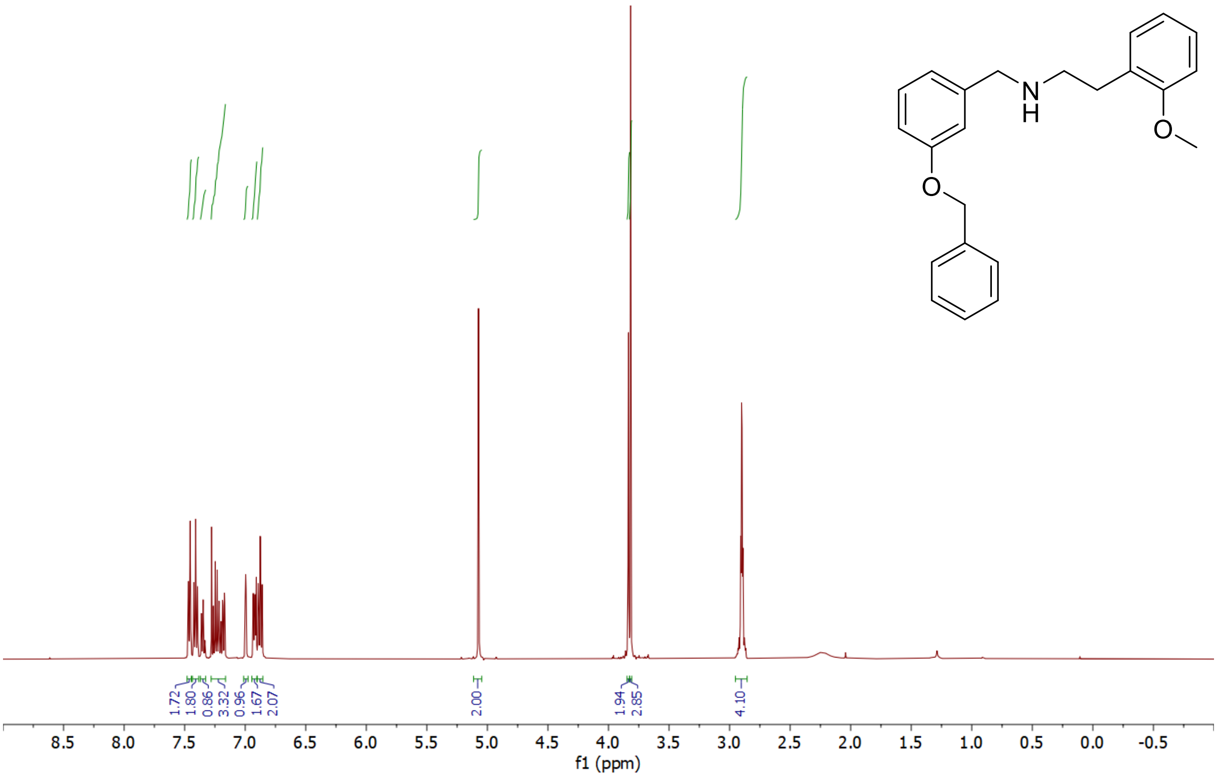

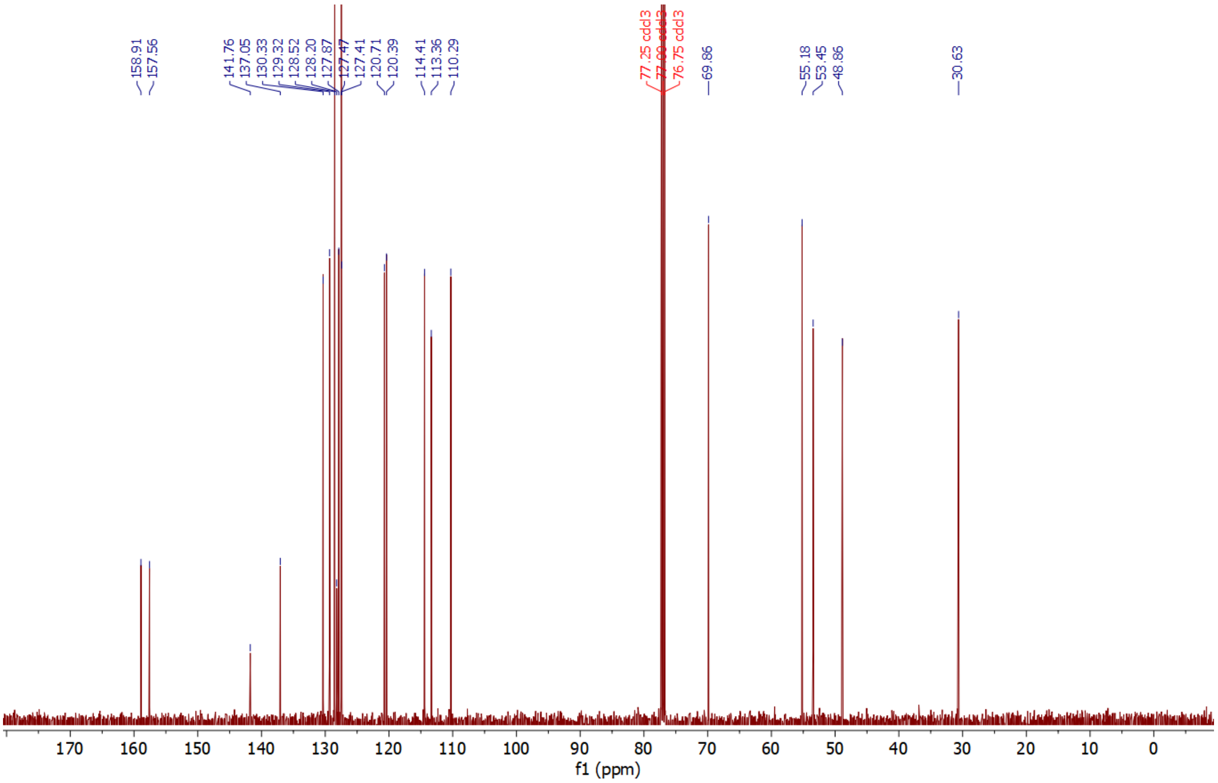

348,1958

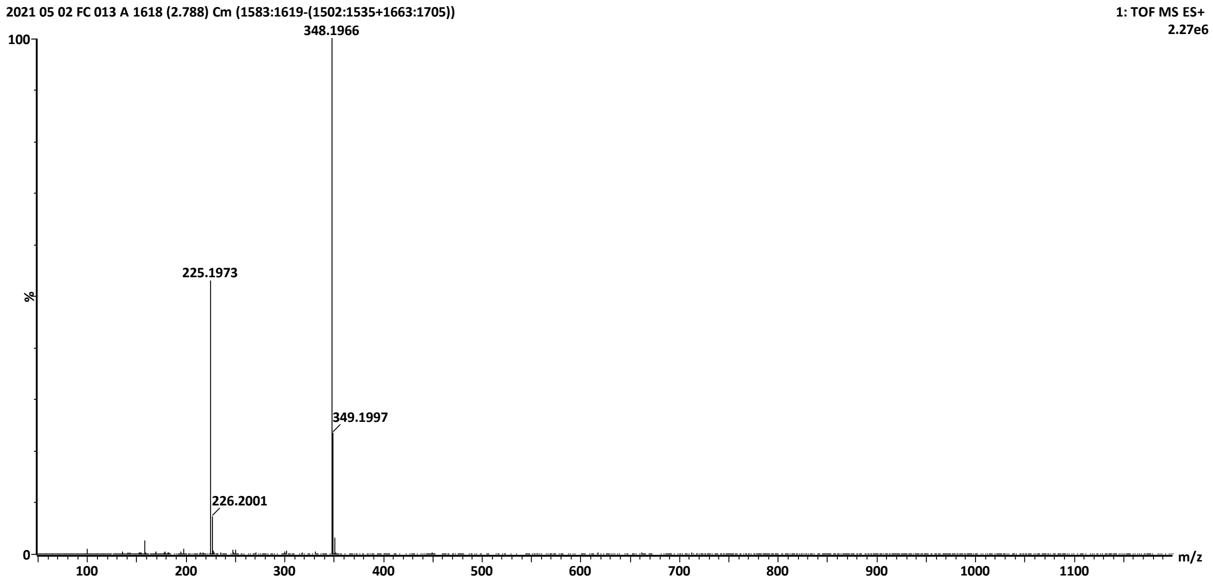

2021 05 02 FC 013 A 1618 (2.788) Cm (1583:1619-(1502:1535+1663:1705))
1: TOF MS ES+
2.27e6
348.1966
225.1973
349.1997
226.2001
100
%
0
m/z
100
200
300
400
500
600
700
800
900
1000
1100

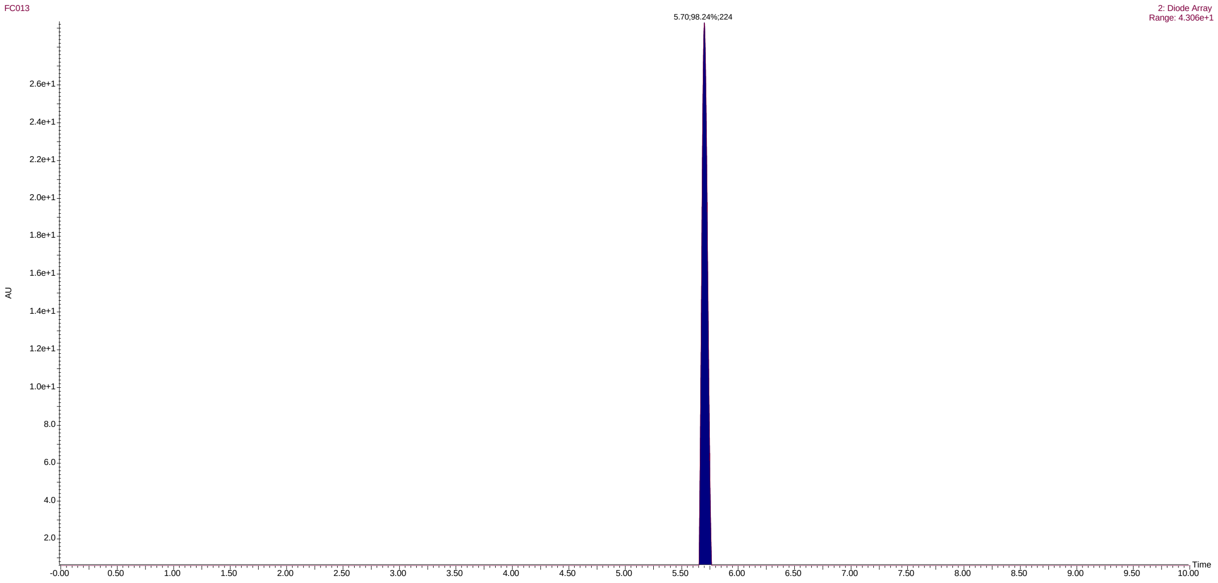

FC013
2: Diode Array
Range: 4.306e+1
5.70;98.24%;224
AU
Time

{[3-(Benzyloxy)phenyl]methyl}[2-(3-methoxyphenyl)ethyl]amine (6):

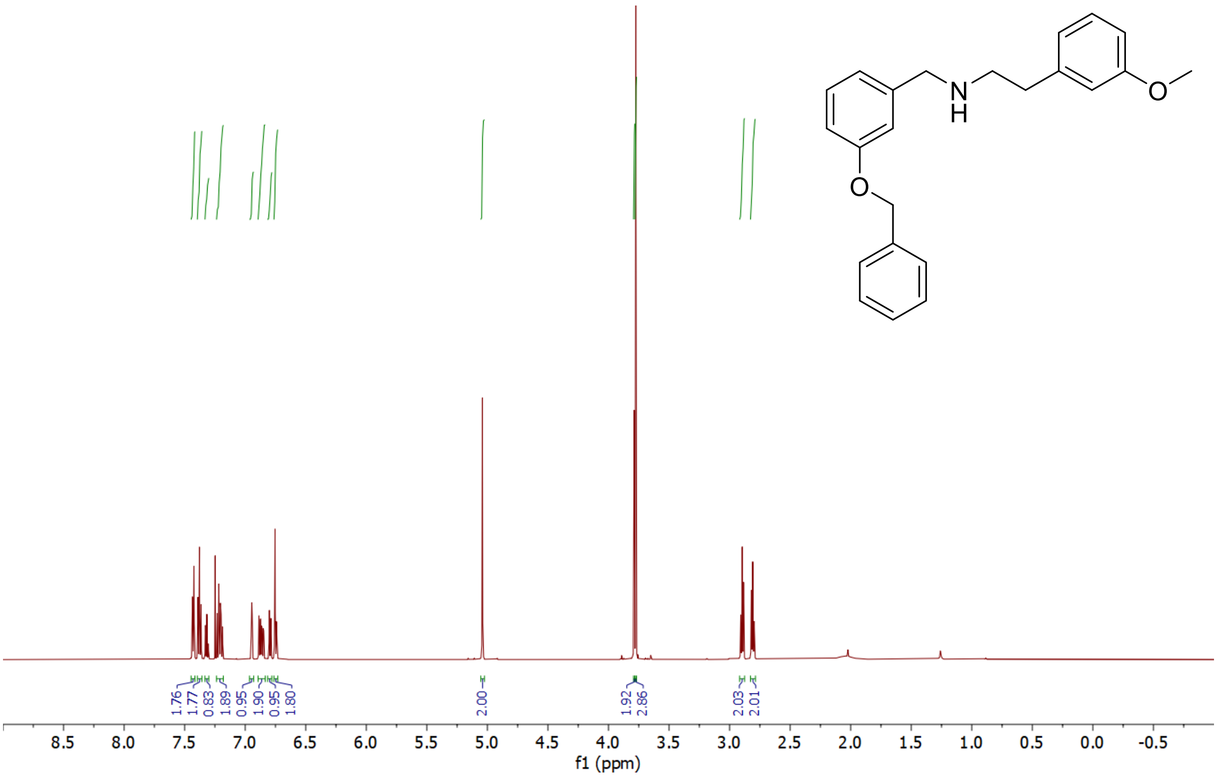

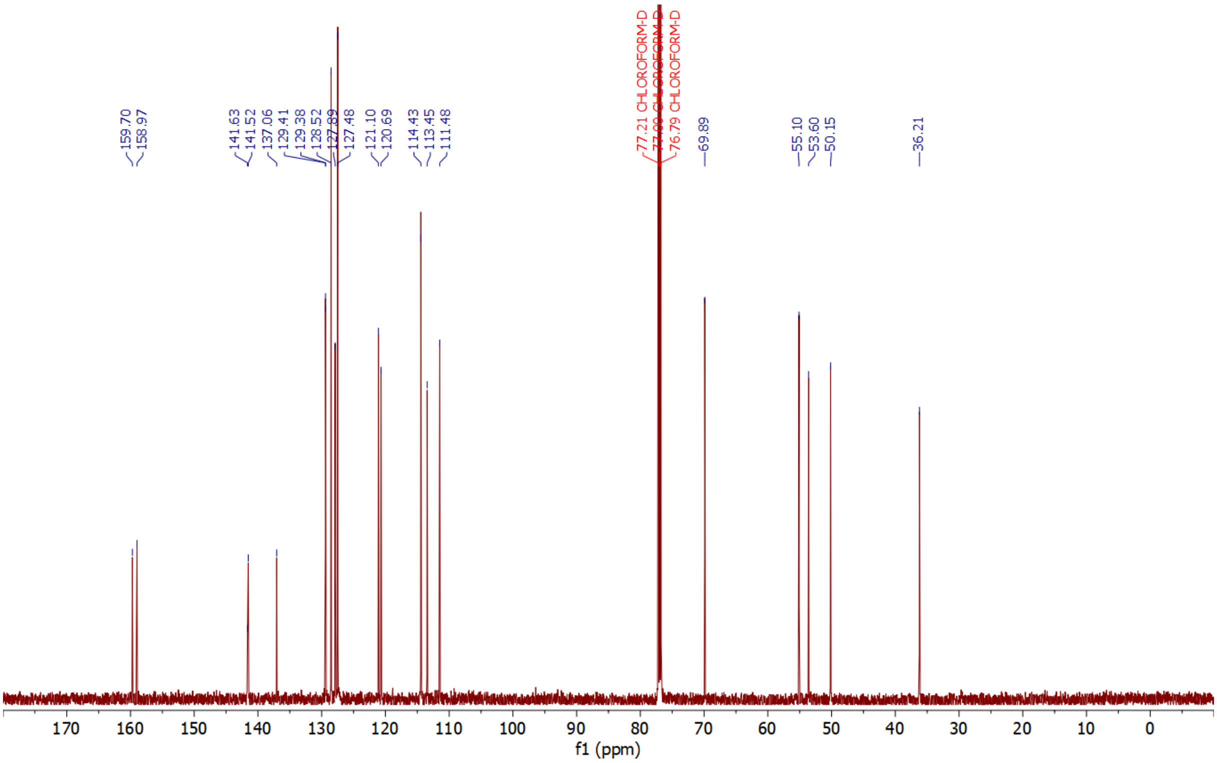

348.1958

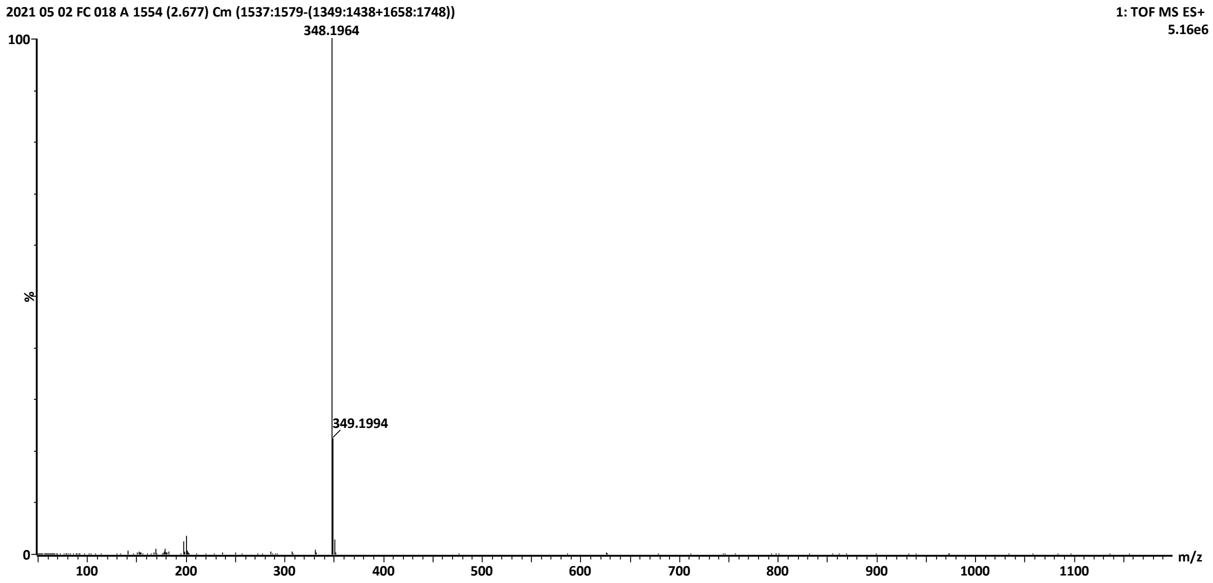
2021 05 02 FC 018 A 1554 (2.677) Cm (1537:1579-(1349:1438+1658:1748))
1: TOF MS ES+
5.16e6
348.1964
349.1994
100
%
0
m/z
100
200
300
400
500
600
700
800
900
1000
1100

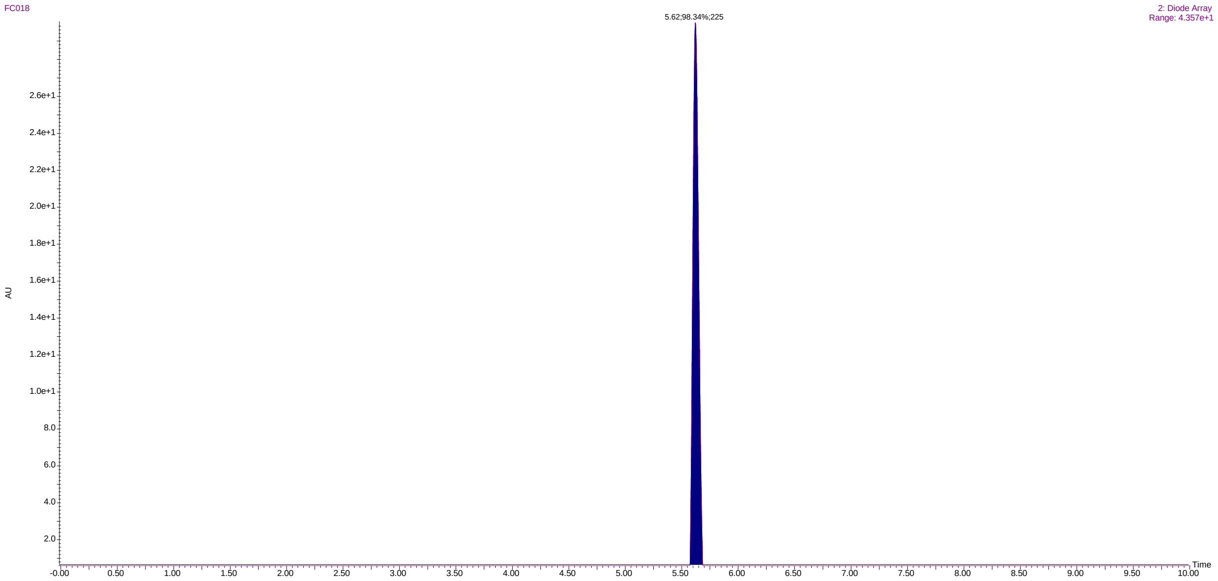
FC018
2: Diode Array
Range: 4.357e+1
5.62;98.34%;225
AU
Time

{[3-(Benzyloxy)phenyl]methyl}[2-(4-methoxyphenyl)ethyl]amine (7):

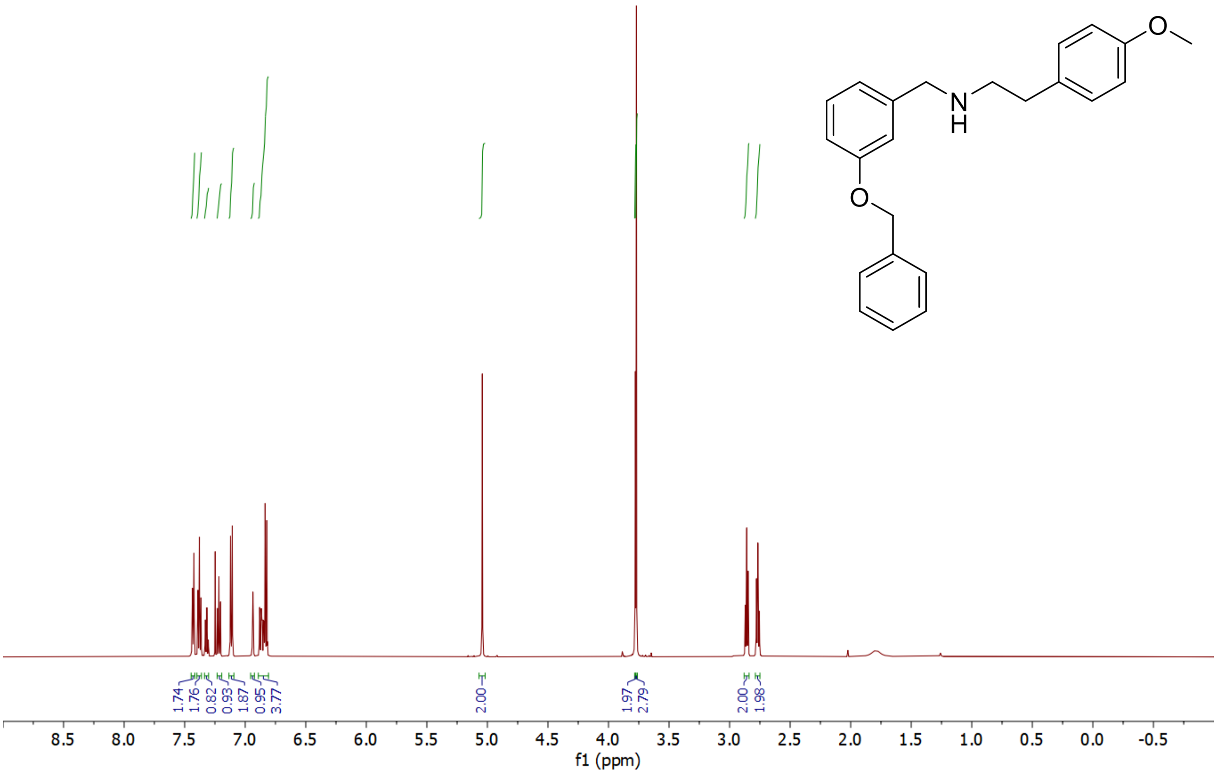

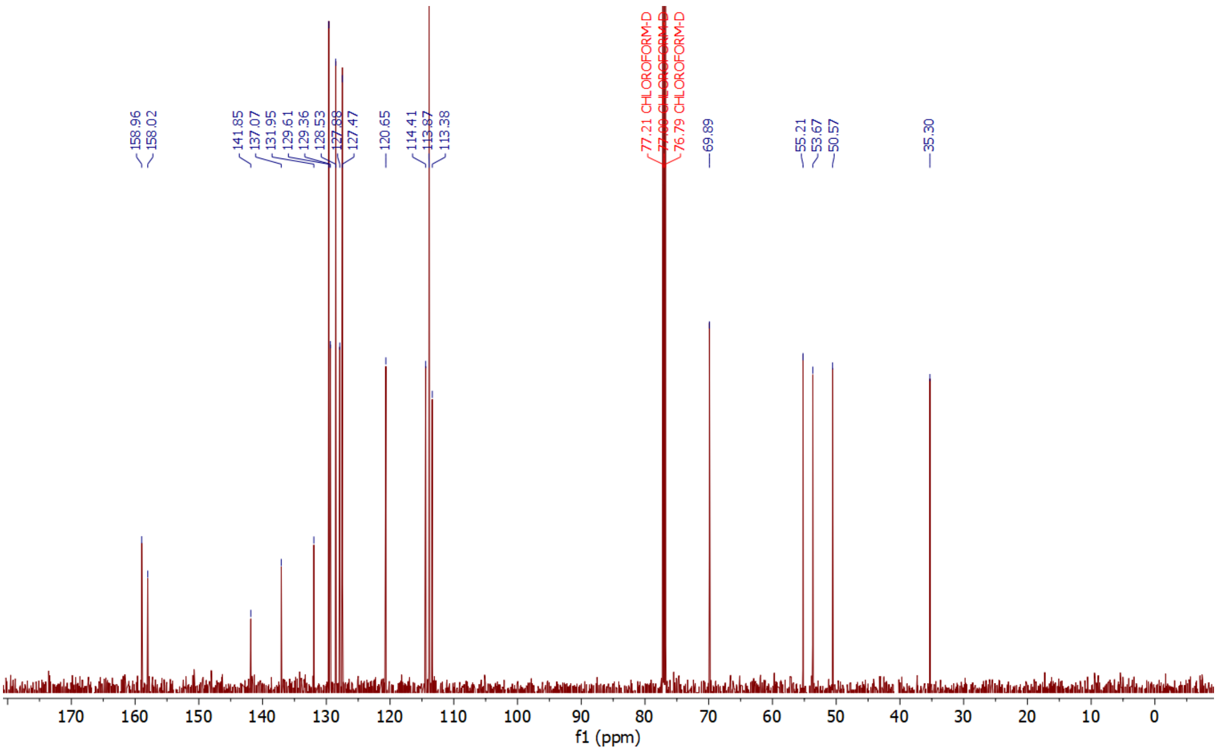

348.1958

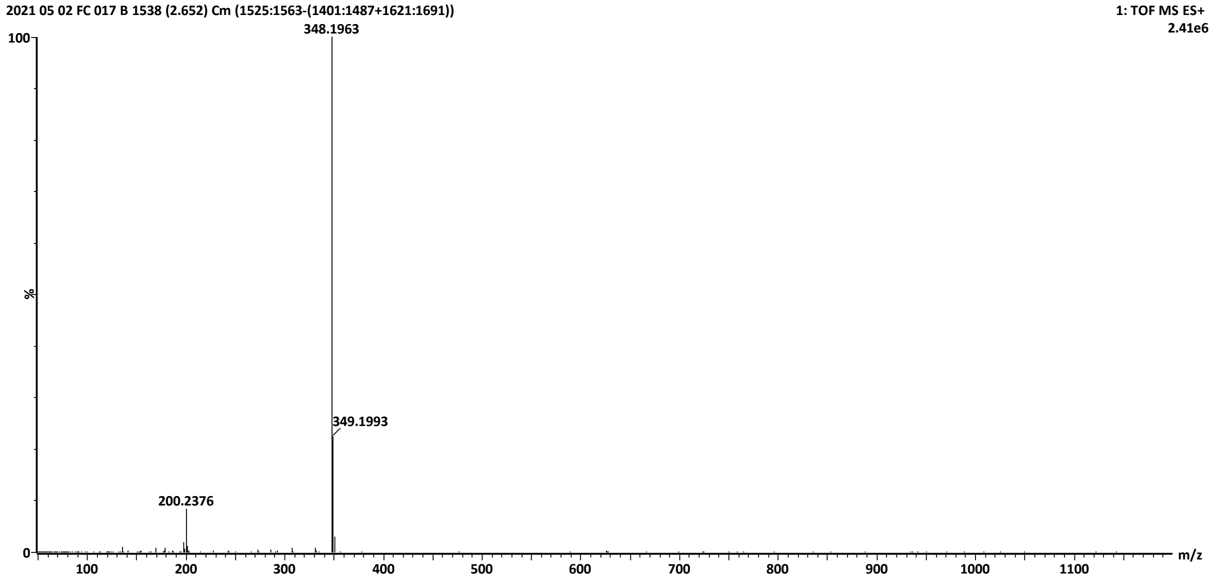

2021 05 02 FC 017 B 1538 (2.652) Cm (1525:1563-(1401:1487+1621:1691))
1: TOF MS ES+
2.41e6
348.1963
349.1993
200.2376
100
%
0
m/z
100
200
300
400
500
600
700
800
900
1000
1100

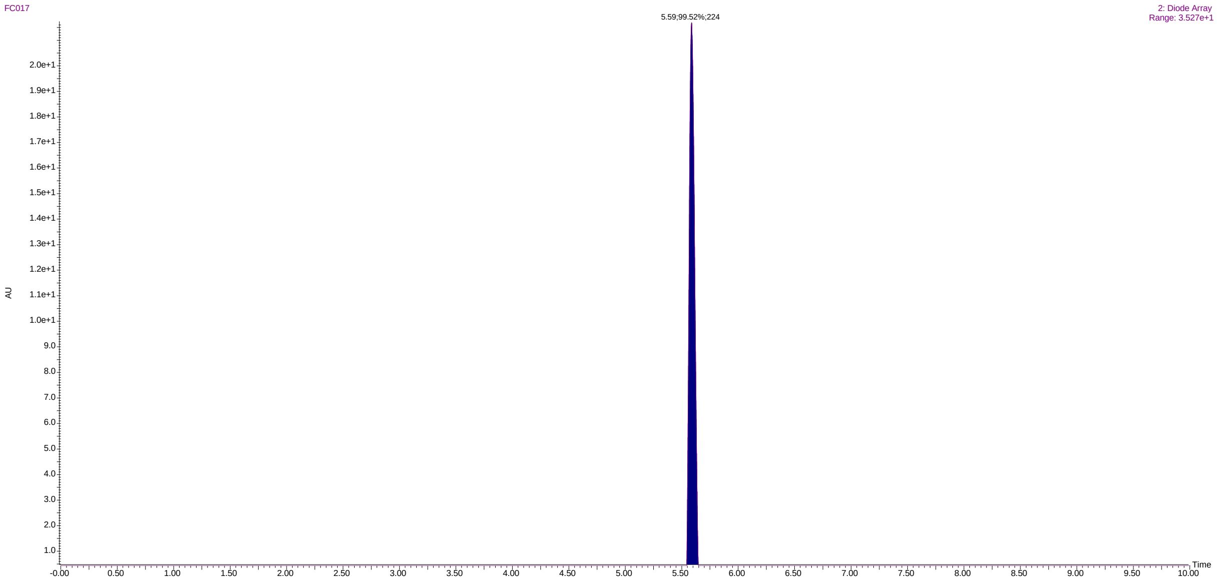

FC017
2: Diode Array
Range: 3.527e+1
5.59;99.52%;224
AU
Time

4-[2-({[3-(Benzyloxy)phenyl]methyl}(prop-2-en-1-yl)amino)ethyl]phenol (8):

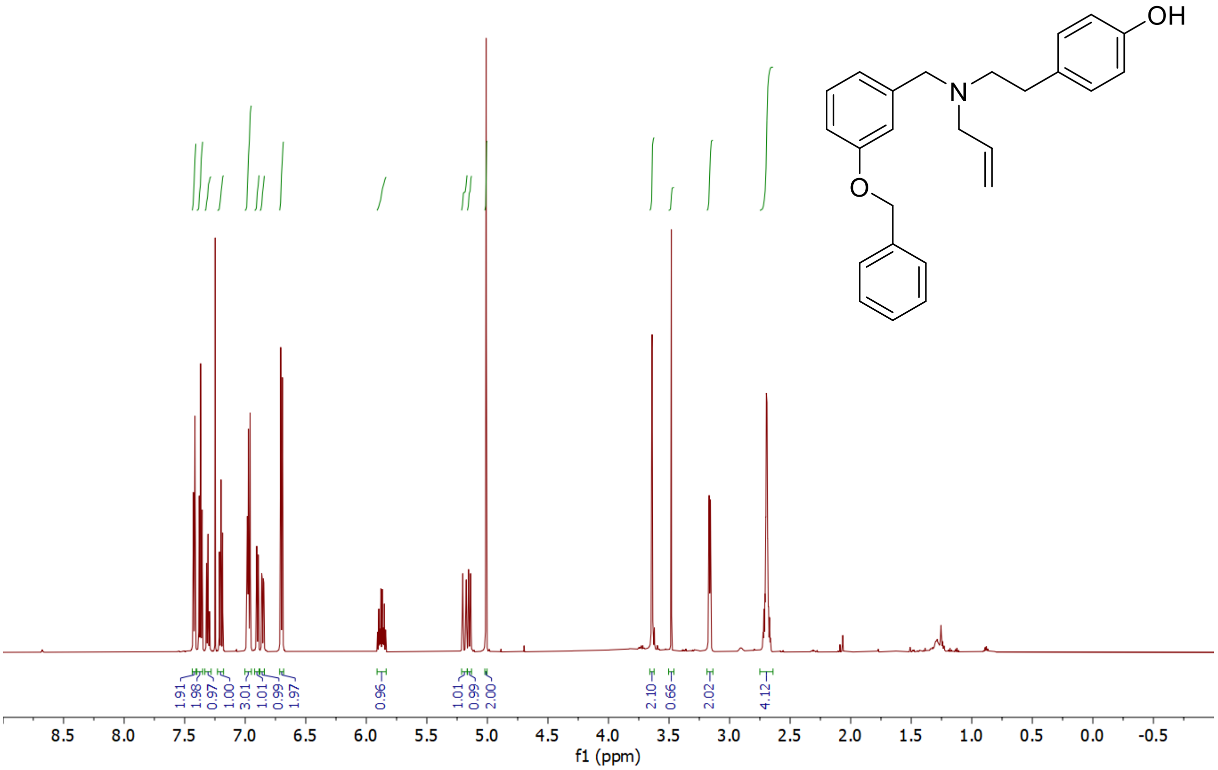

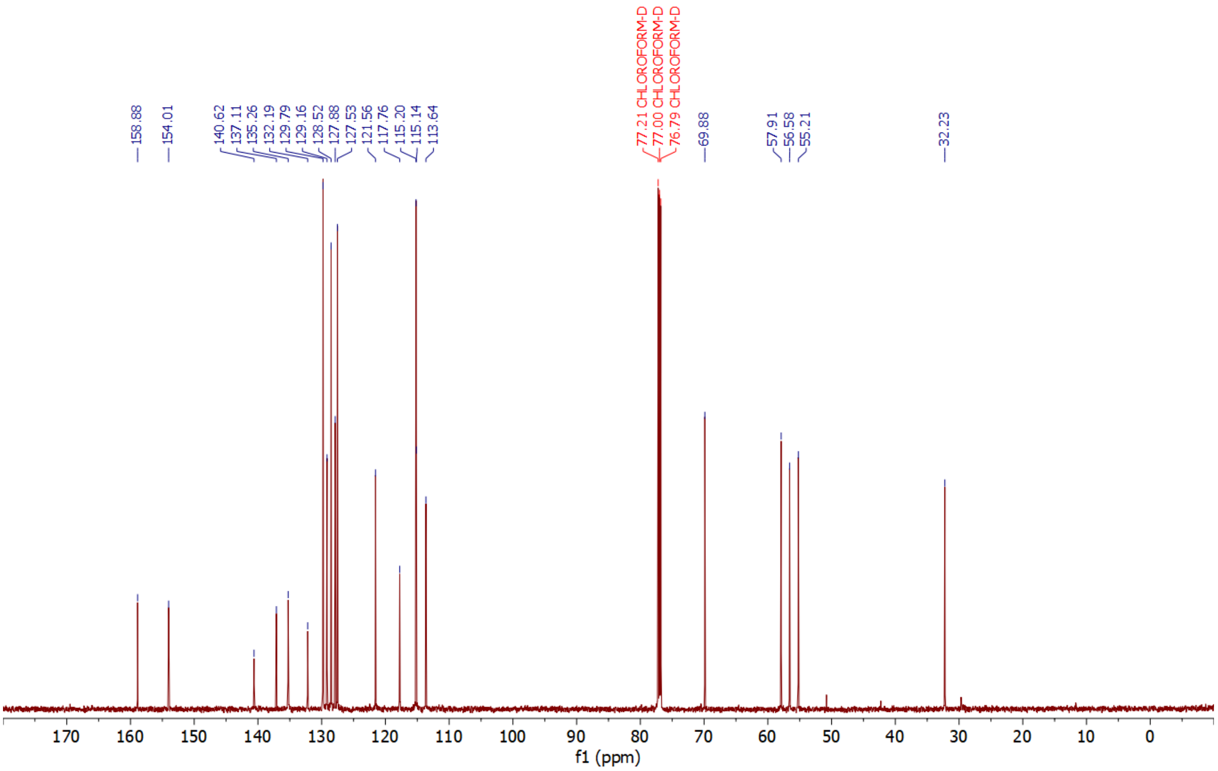

374,2115

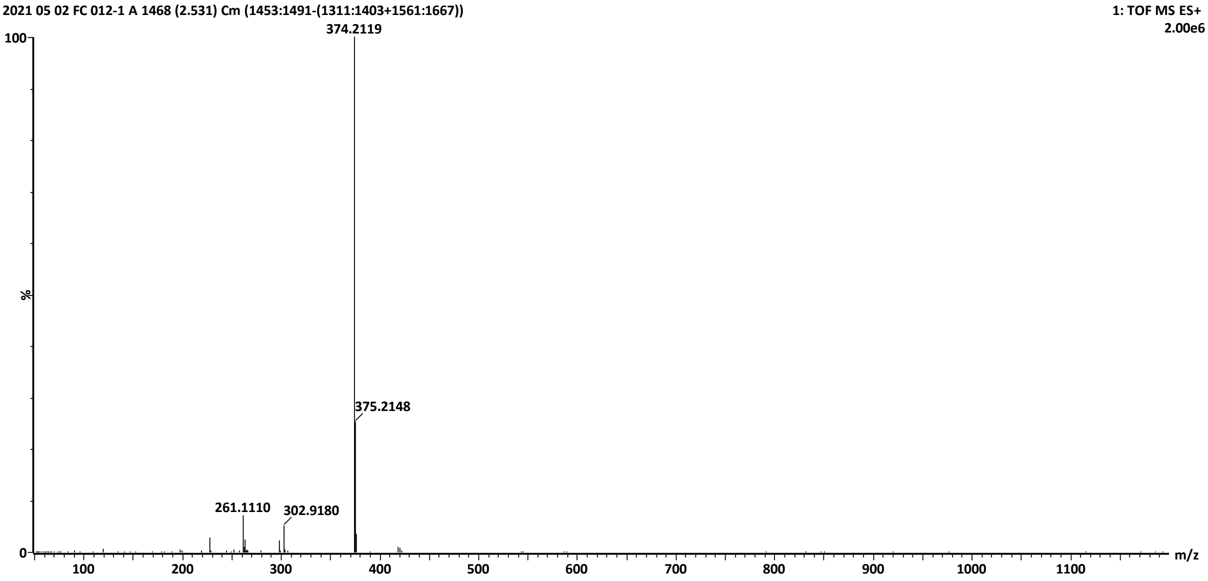
2021 05 02 FC 012-1 A 1468 (2.531) Cm (1453:1491-(1311:1403+1561:1667))
1: TOF MS ES+
2.00e6
374.2119
375.2148
261.1110
302.9180
%
m/z

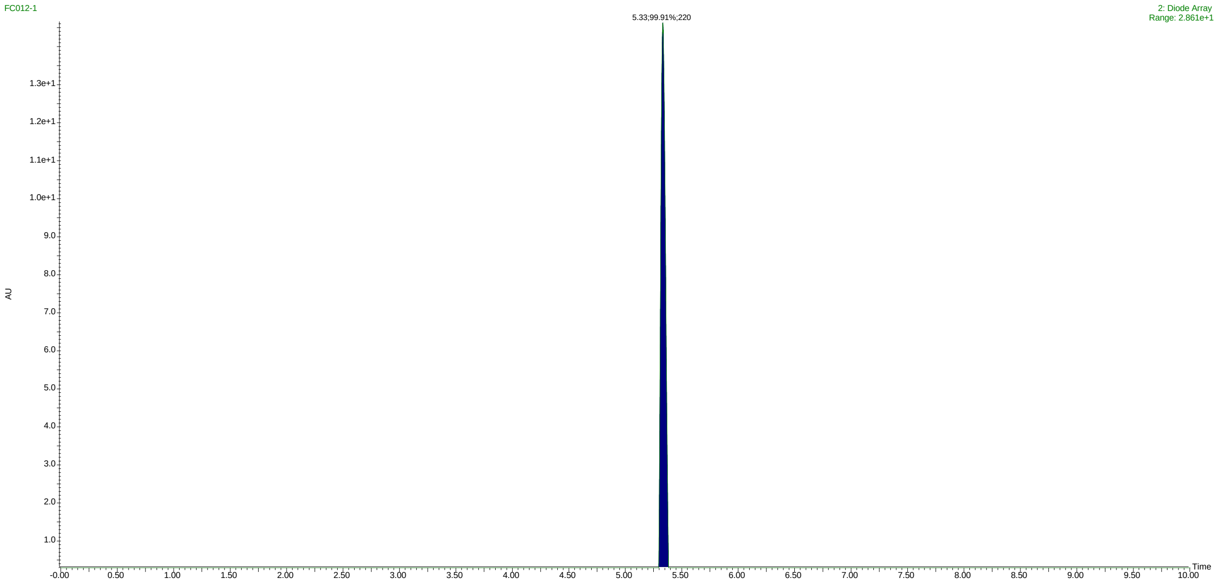
FC012-1
2: Diode Array
Range: 2.861e+1
5.33;99.91%;220
AU
Time

{[3-(Benzyloxy)phenyl]methyl}(prop-2-en-1-yl){2-[4-(prop-2-en-1-yloxy)phenyl]ethyl}amine (9):

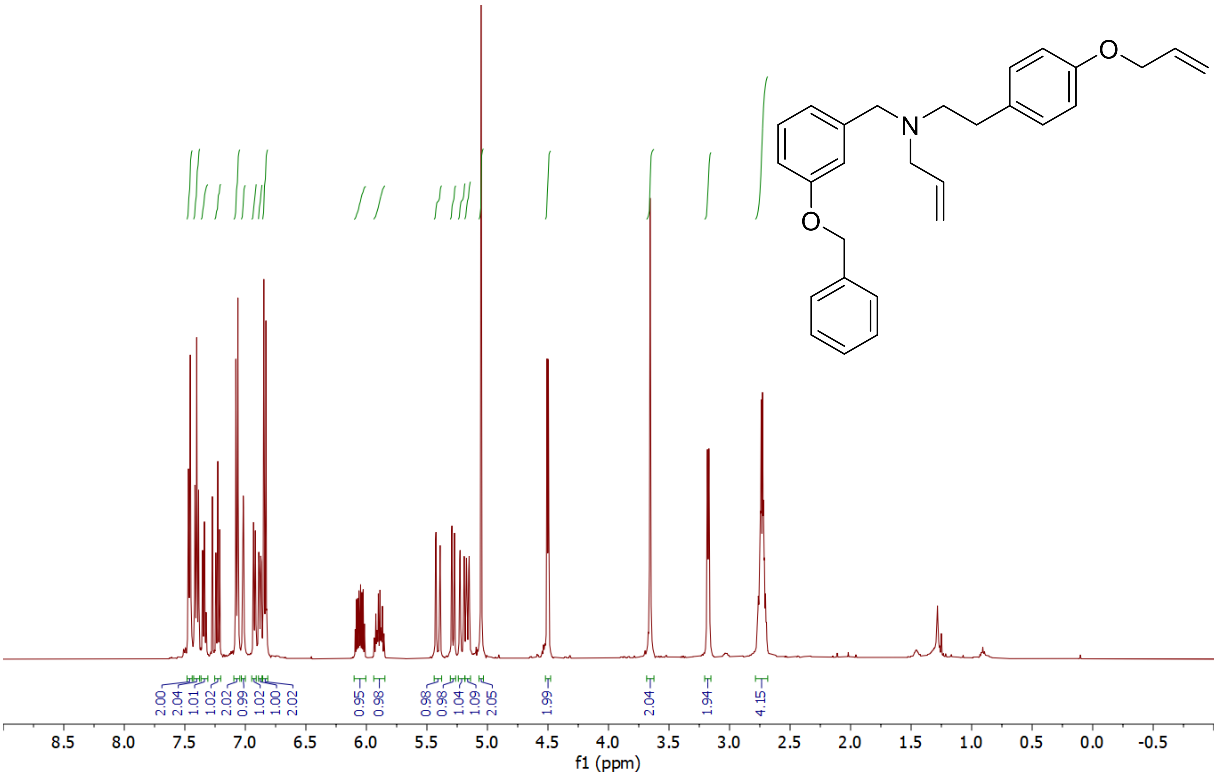

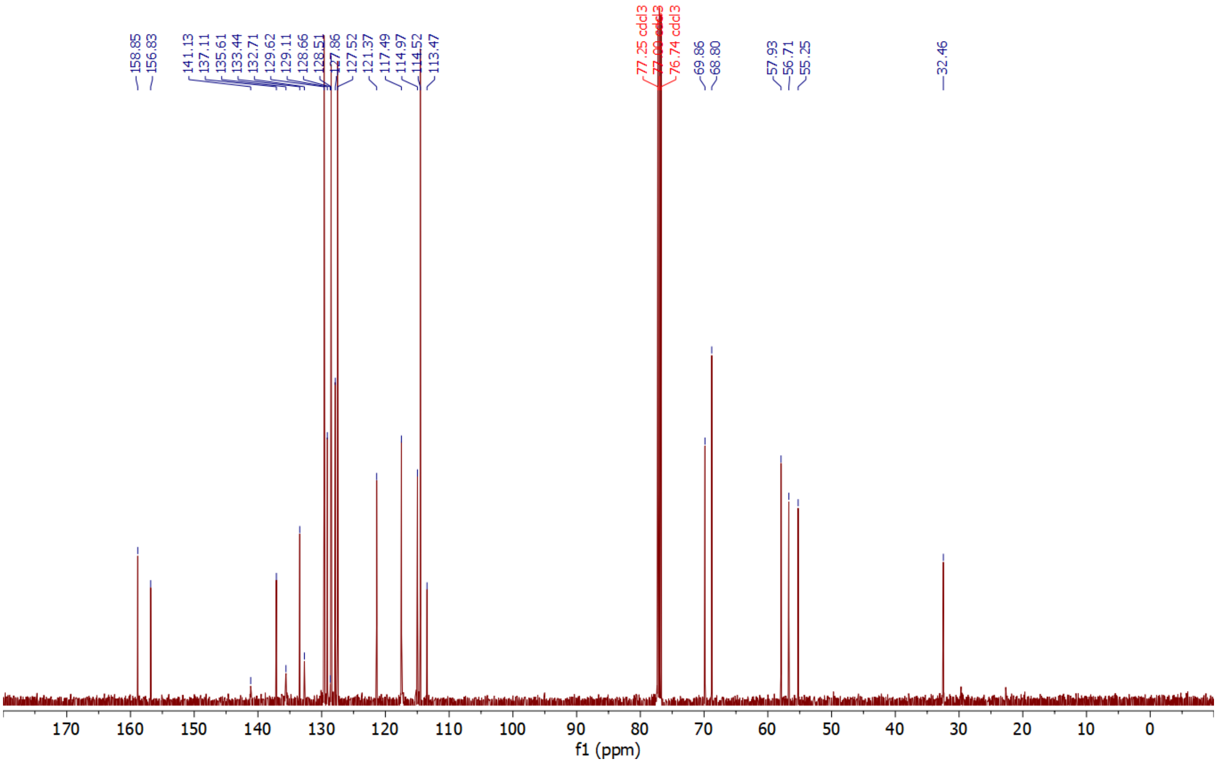

414,2428

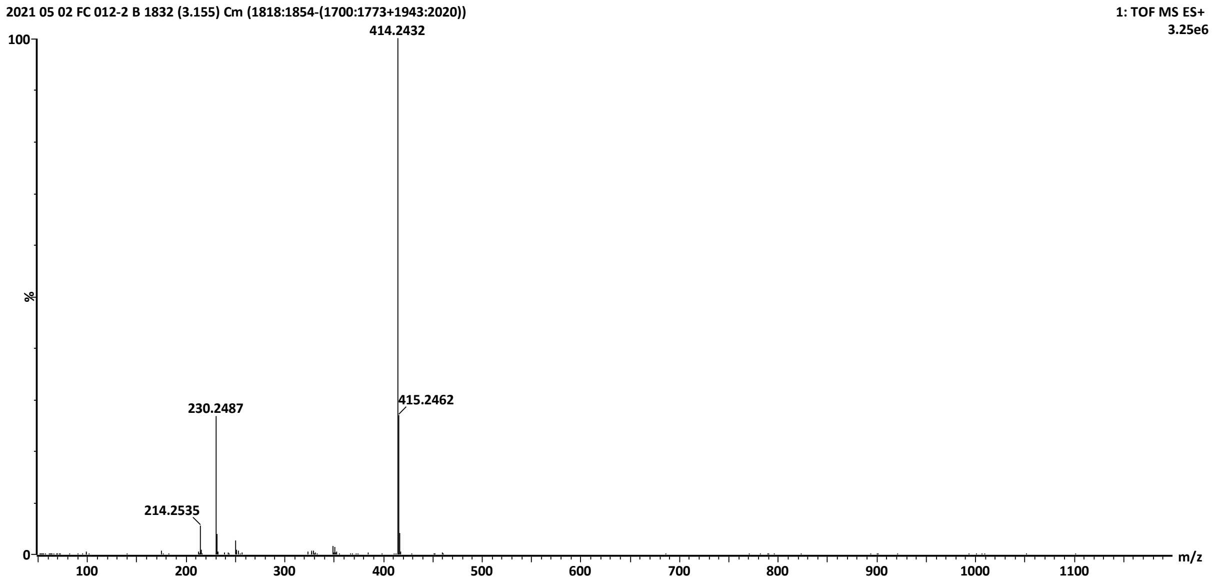
2021 05 02 FC 012-2 B 1832 (3.155) Cm (1818:1854-(1700:1773+1943:2020))
1: TOF MS ES+
3.25e6
414.2432
415.2462
230.2487
214.2535
%
m/z

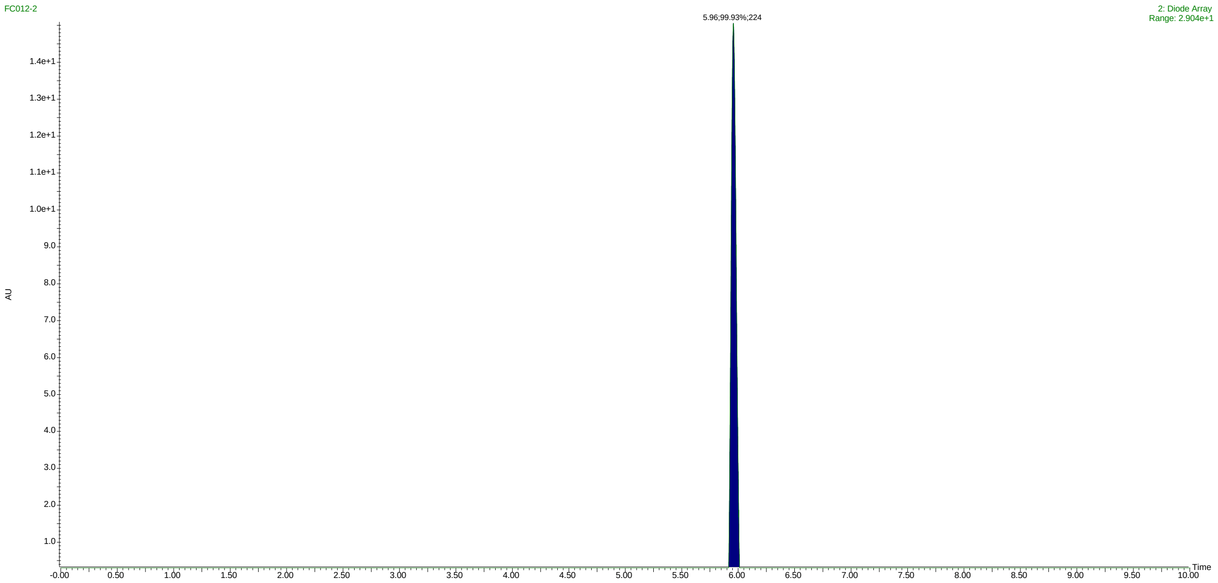
FC012-2
2: Diode Array
Range: 2.904e+1
5.96;99.93%;224
AU
Time

Tert-butyl N-{[3-(benzyloxy)phenyl]methyl}-N-{2-[4-(prop-2-en-1-yloxy)phenyl]ethyl}carbamate (11):

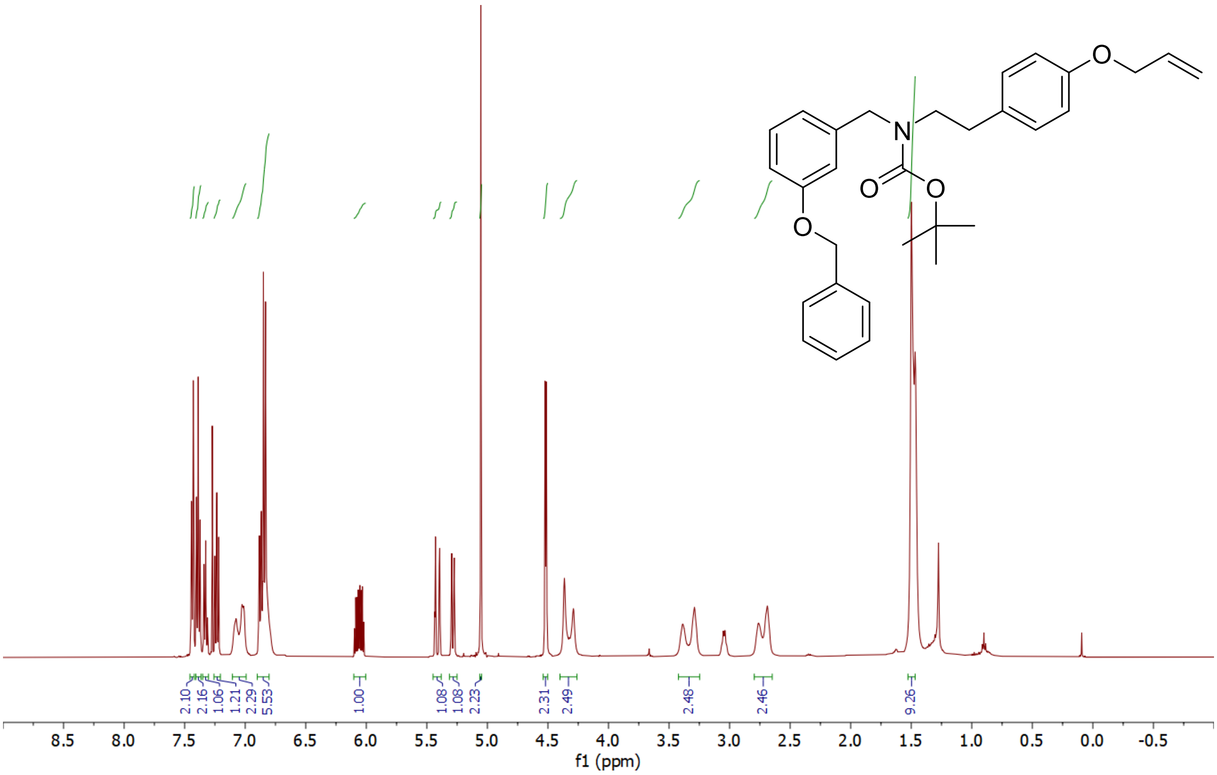

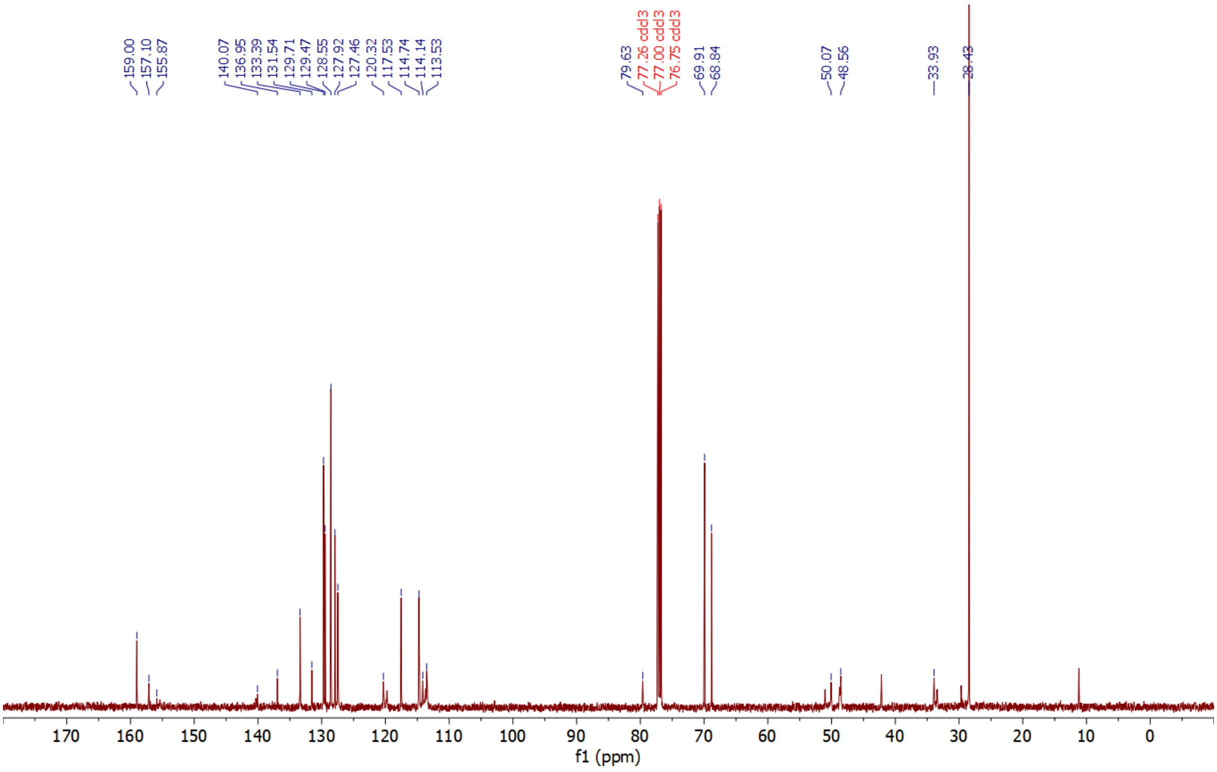

{[3-(Benzyloxy)phenyl]methyl}({2-[4-(prop-2-en-1-yloxy)phenyl]ethyl})amine (12):

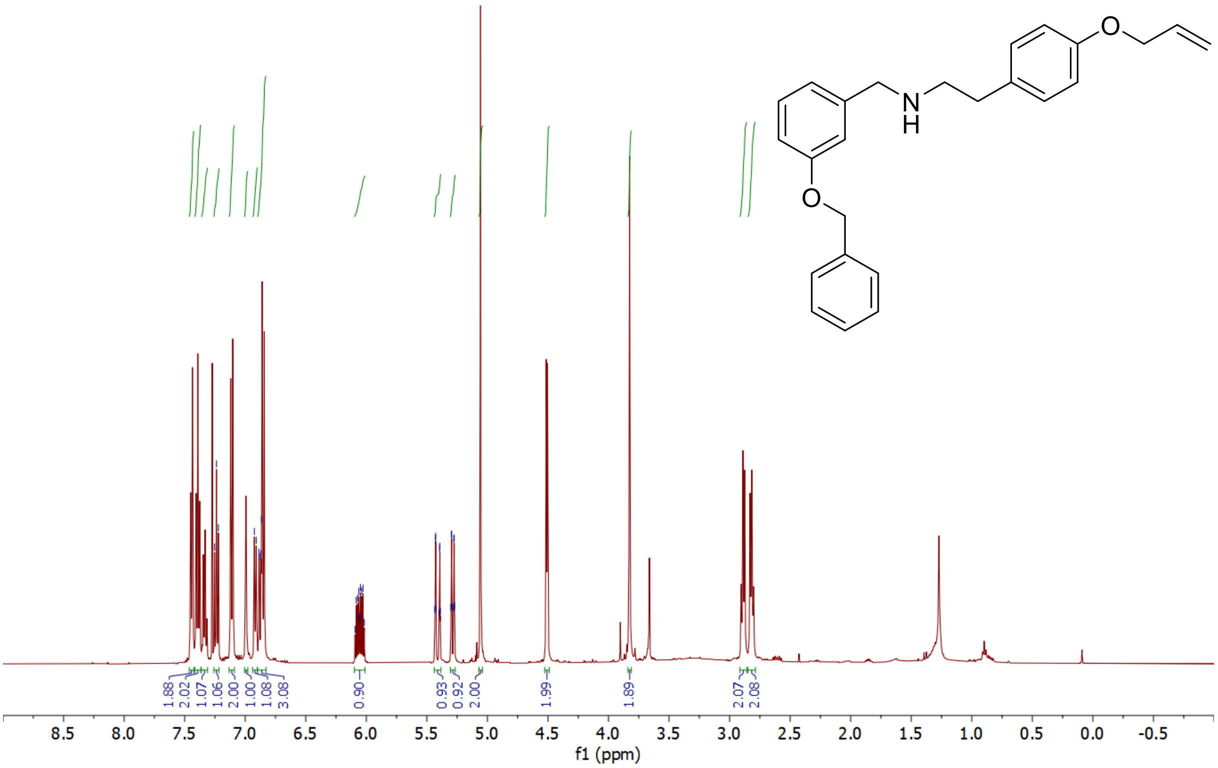

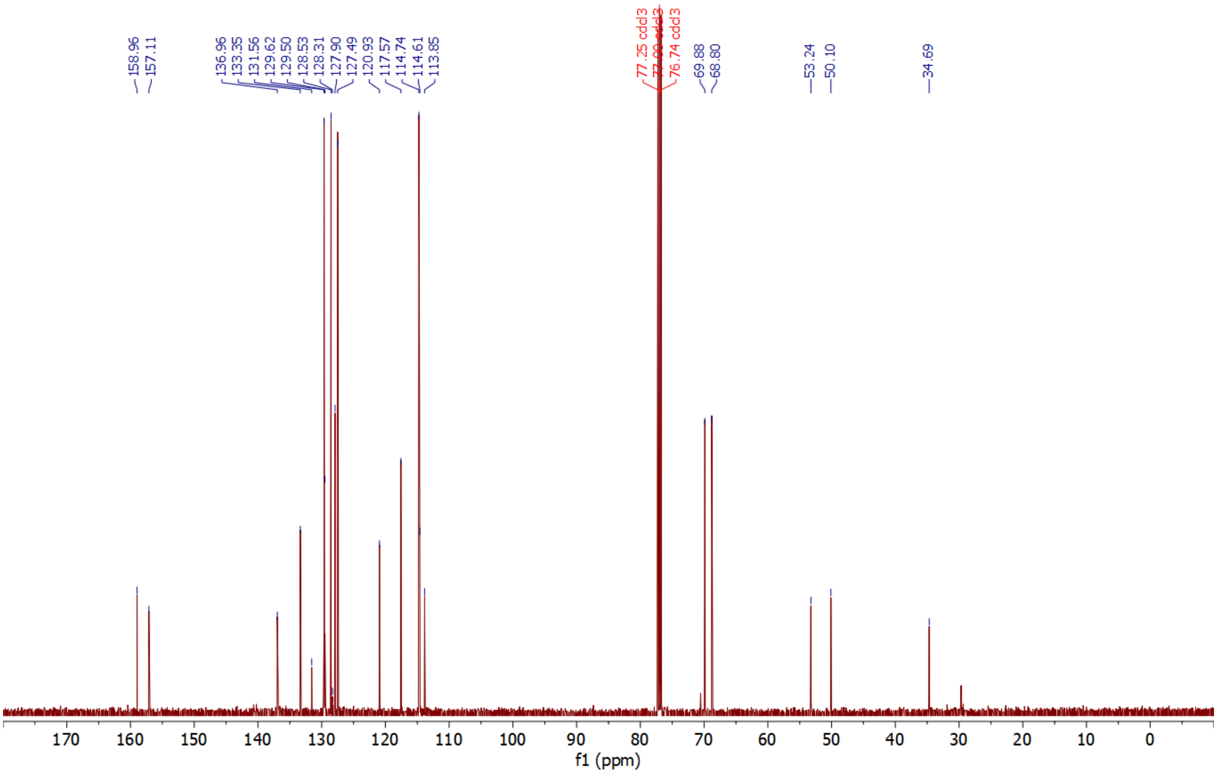

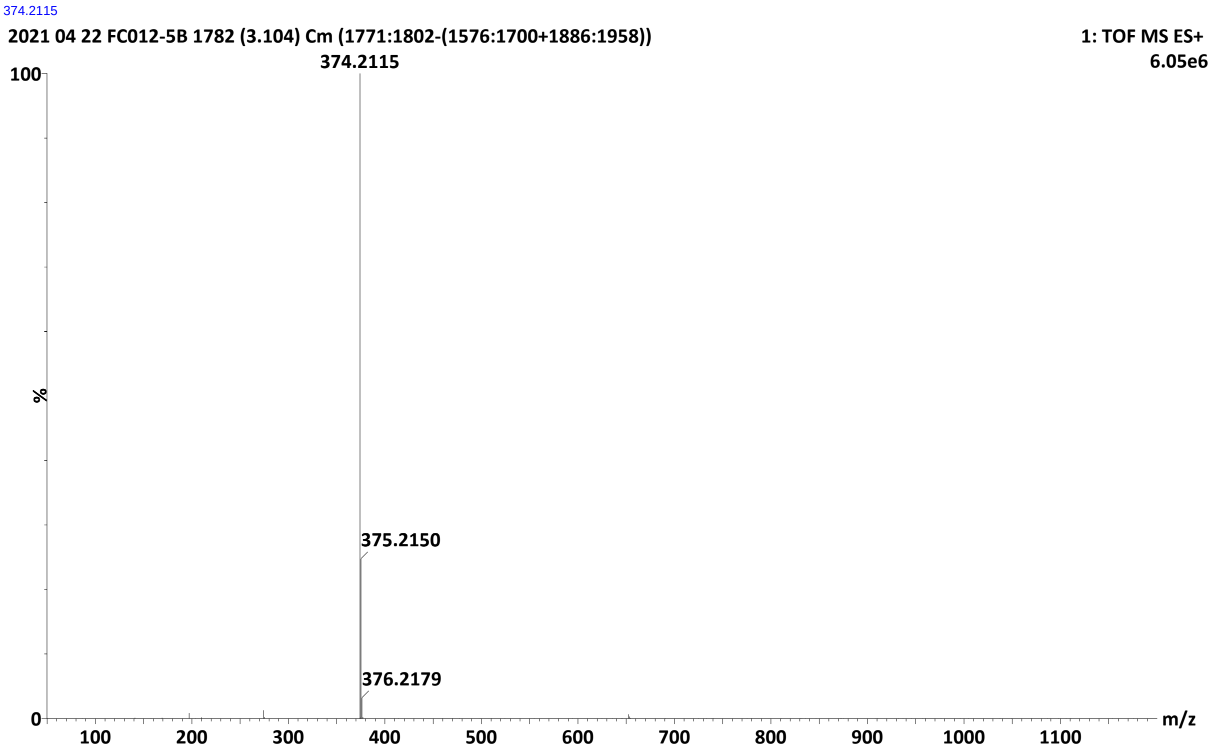
374.2115
2021 04 22 FC012-5B 1782 (3.104) Cm (1771:1802-(1576:1700+1886:1958))
1: TOF MS ES+
6.05e6
374.2115
375.2150
376.2179
100
%
0
m/z
100
200
300
400
500
600
700
800
900
1000
1100

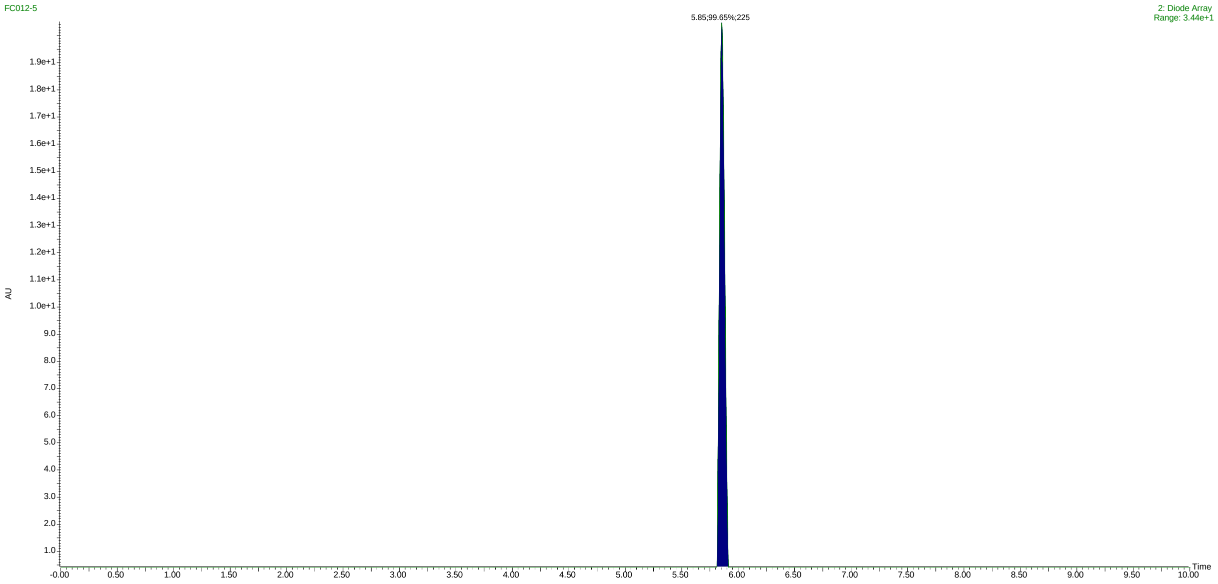
FC012-5
2: Diode Array
Range: 3.44e+1
5.85;99.65%;225
AU
Time

N-{[3-(Benzyloxy)phenyl]methyl}-3-chloroaniline (13):

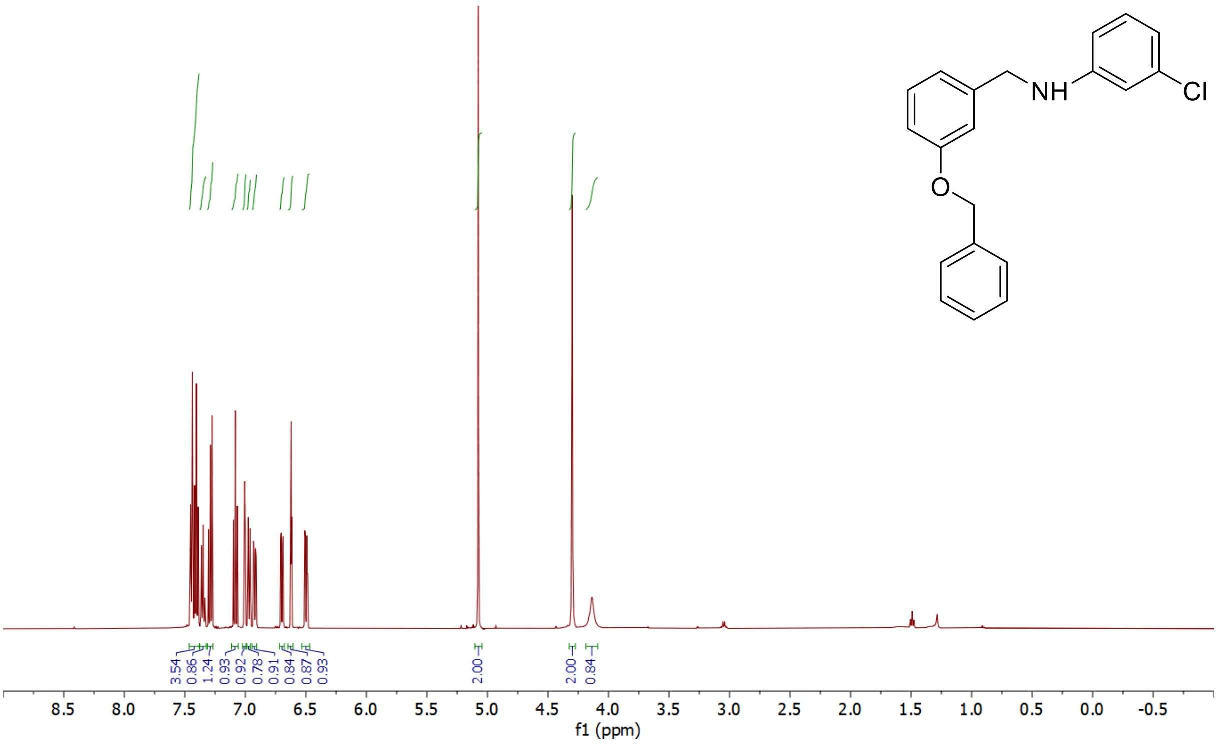

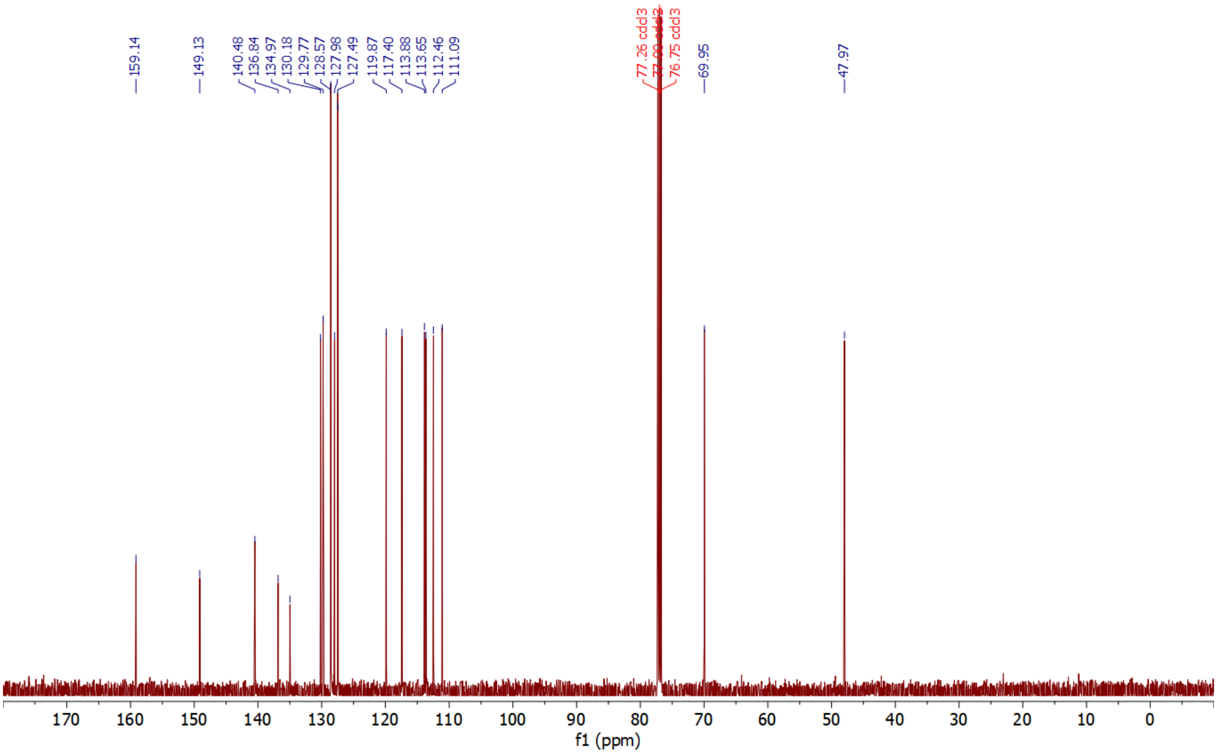

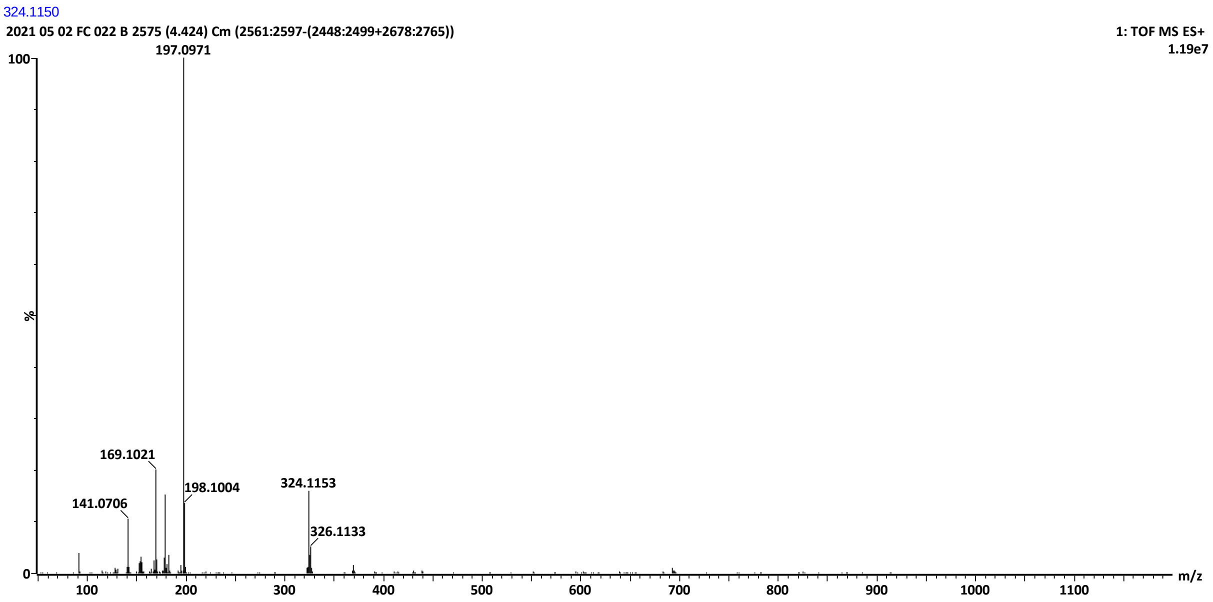
324.1150
2021 05 02 FC 022 B 2575 (4.424) Cm (2561:2597-(2448:2499+2678:2765))
1: TOF MS ES+
1.19e7
197.0971
169.1021
198.1004
141.0706
324.1153
326.1133
%
m/z

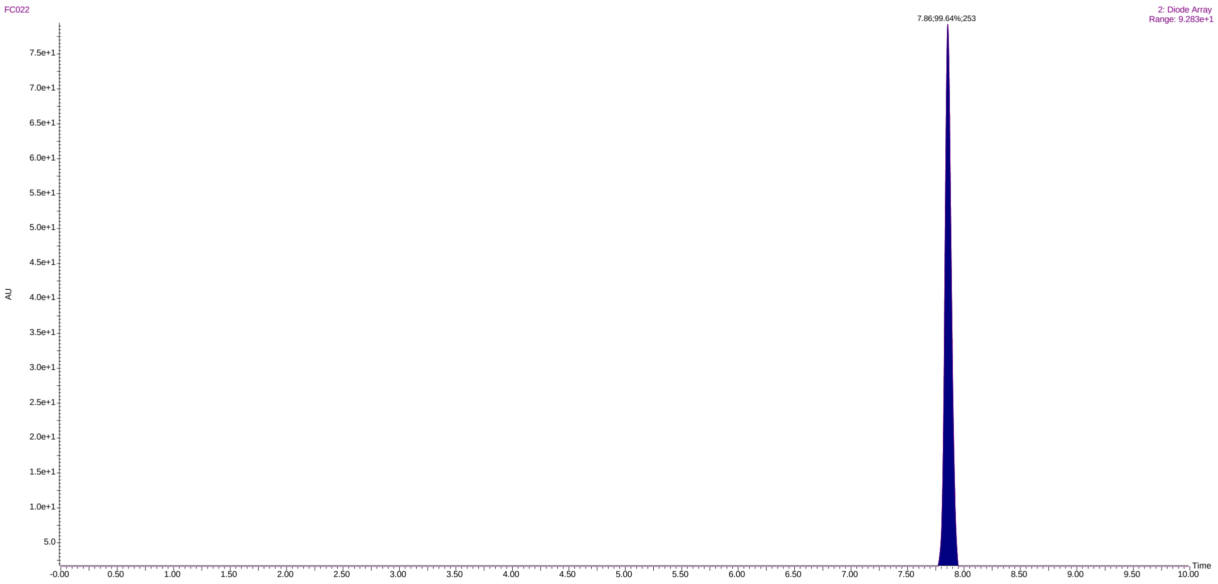
FC022
2: Diode Array
Range: 9.283e+1
7.86;99.64%;253
AU
Time

Benzyl({[3-(benzyloxy)phenyl]methyl})amine (14):

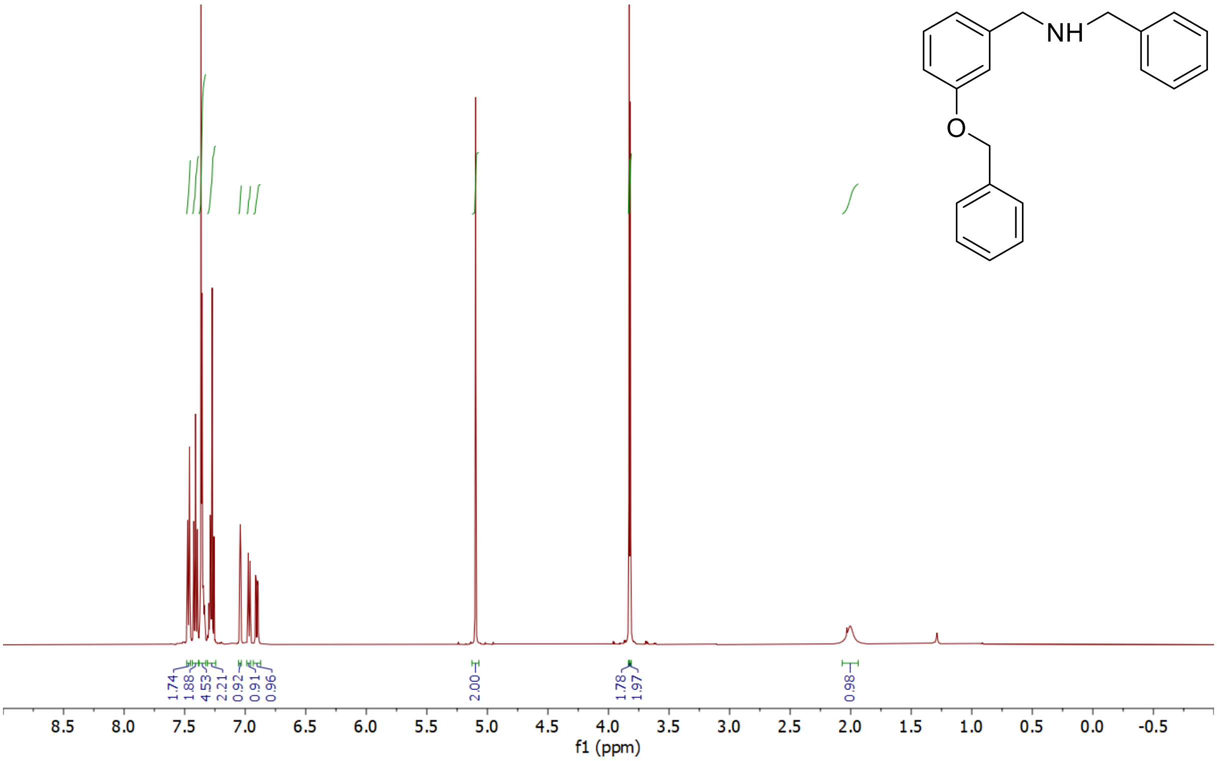

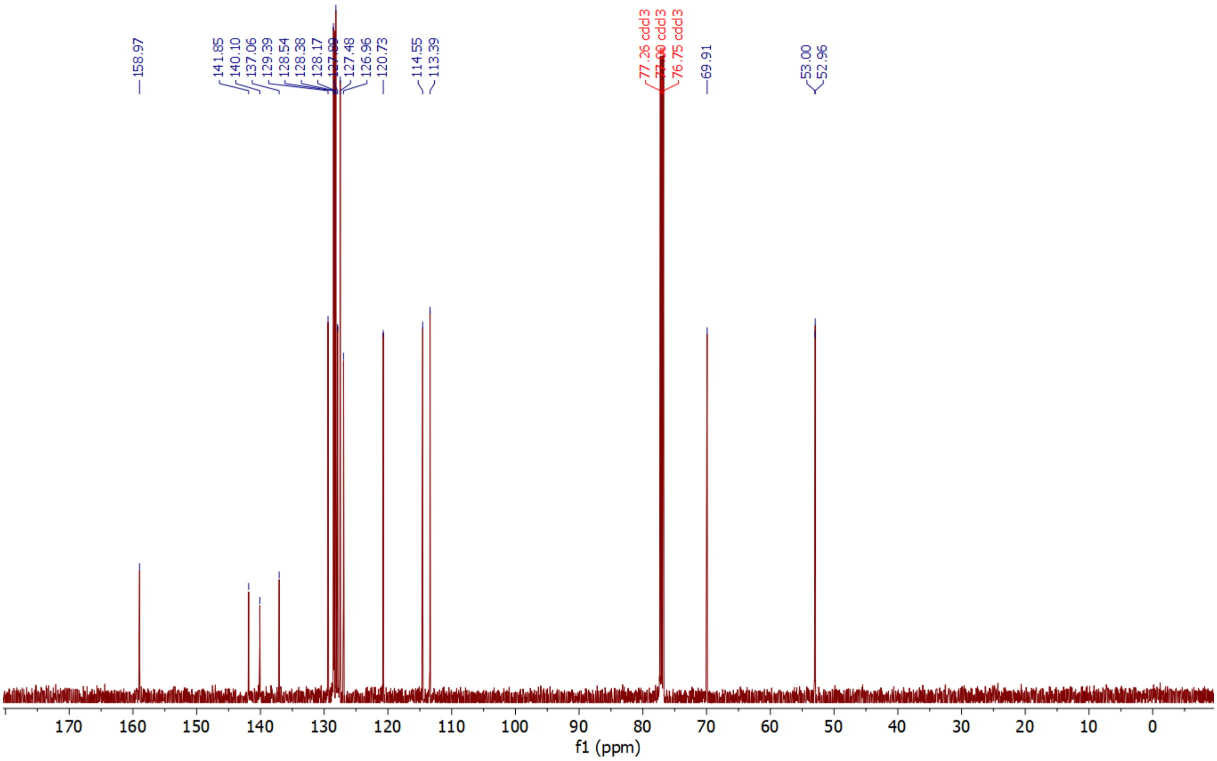

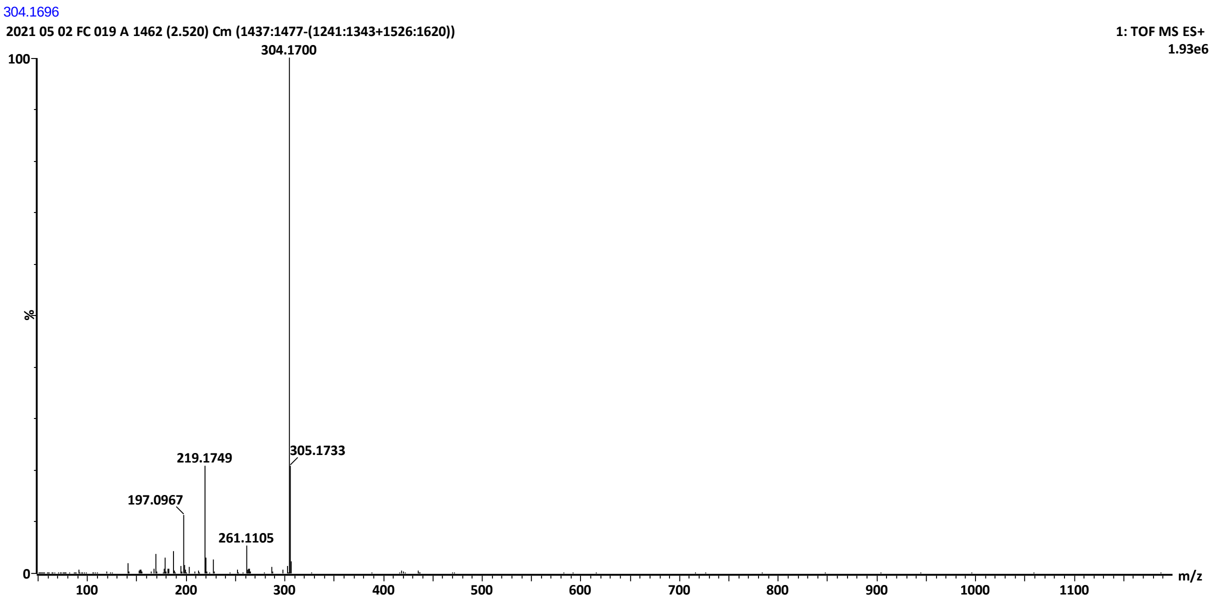
304.1696
2021 05 02 FC 019 A 1462 (2.520) Cm (1437:1477-(1241:1343+1526:1620))
1: TOF MS ES+
1.93e6
304.1700
305.1733
219.1749
197.0967
261.1105
100
%
0
m/z
100
200
300
400
500
600
700
800
900
1000
1100

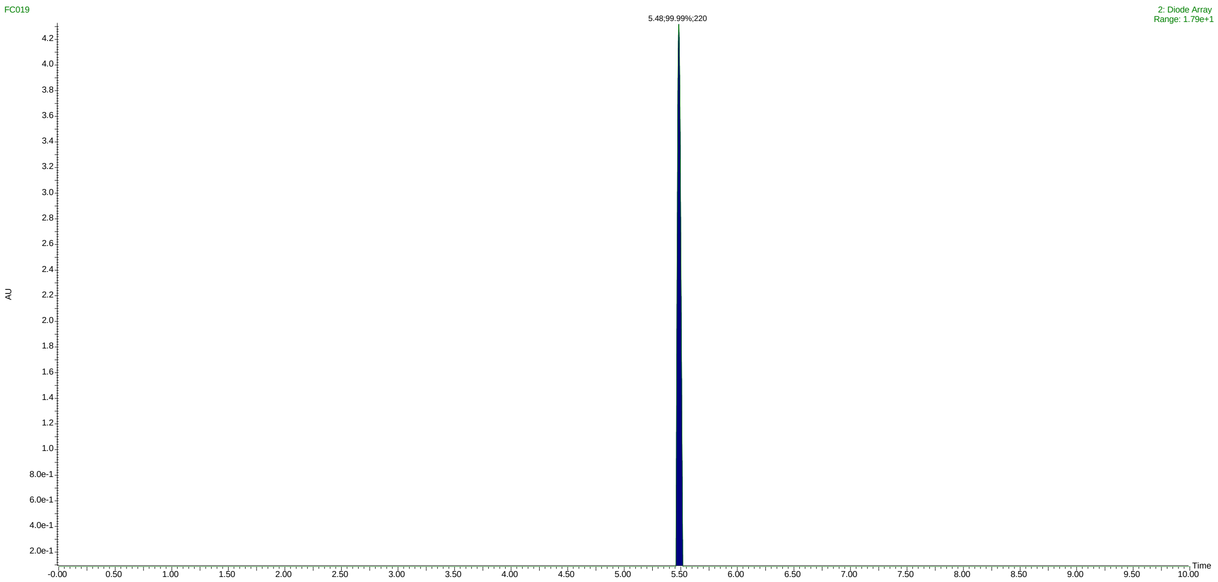
FC019
2: Diode Array
Range: 1.79e+1
5.48;99.99%;220
AU
Time

{[3-(Benzyloxy)phenyl]methyl}[(3,4-dimethoxyphenyl)methyl]amine (15):

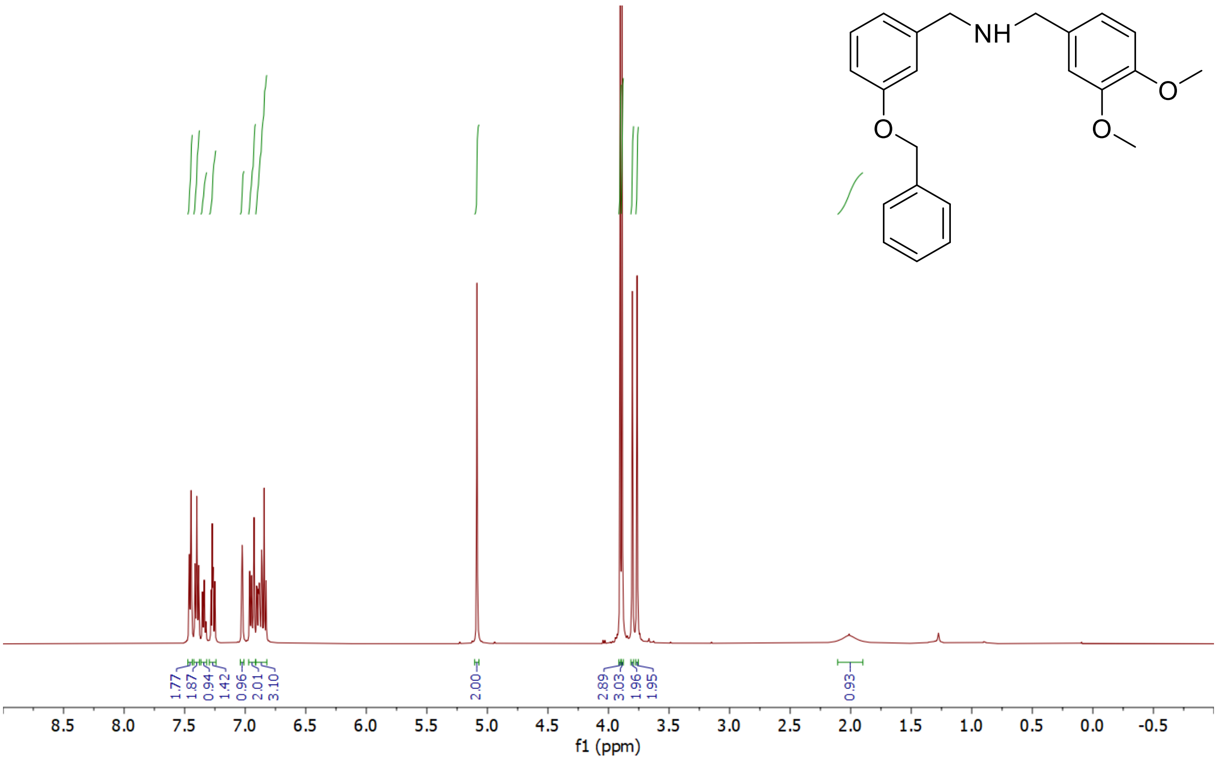

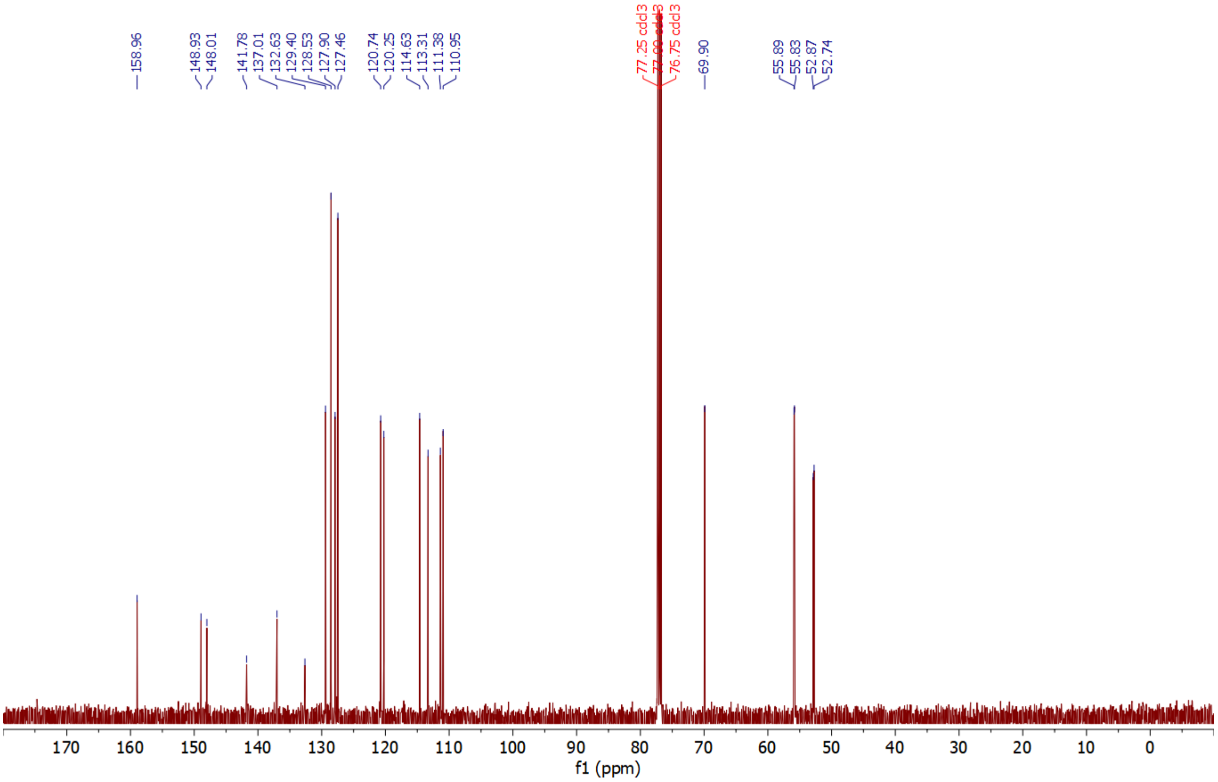

364.1907

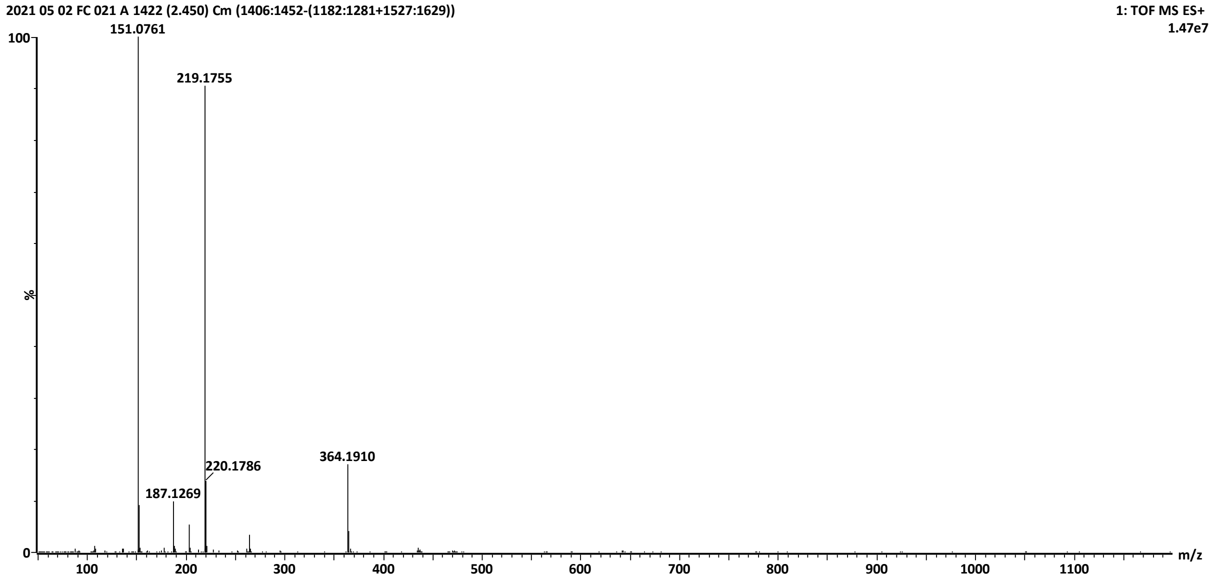
2021 05 02 FC 021 A 1422 (2.450) Cm (1406:1452-(1182:1281+1527:1629))
1: TOF MS ES+
1.47e7
151.0761
219.1755
220.1786
364.1910
187.1269
100
%
0
m/z
100
200
300
400
500
600
700
800
900
1000
1100

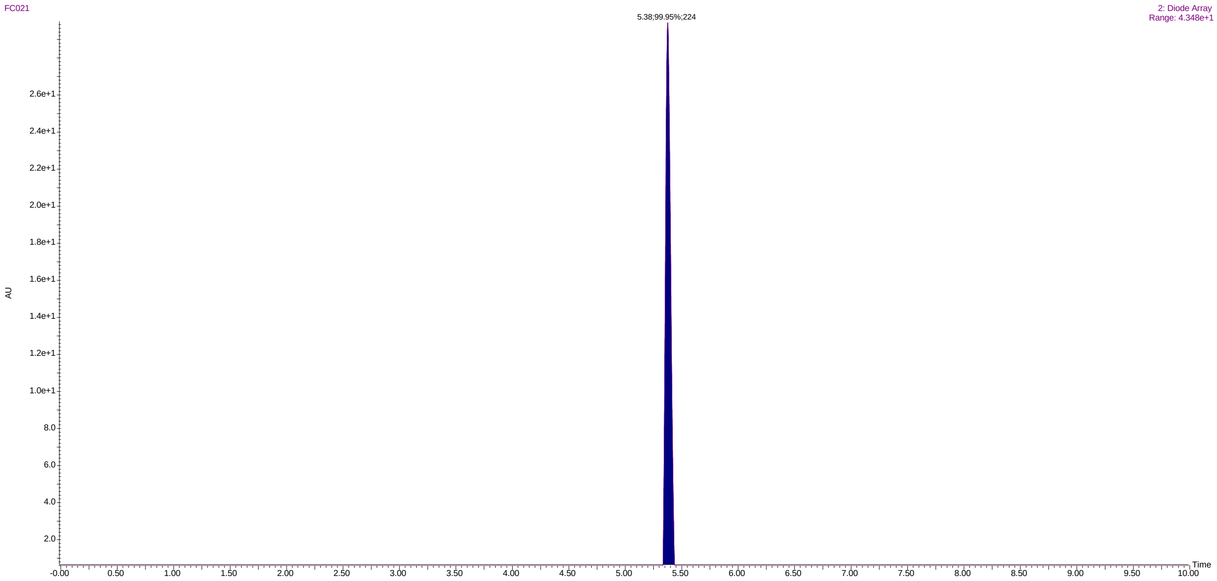
FC021
2: Diode Array
Range: 4.348e+1
5.38;99.95%;224
AU
2.6e+1
2.4e+1
2.2e+1
2.0e+1
1.8e+1
1.6e+1
1.4e+1
1.2e+1
1.0e+1
8.0
6.0
4.0
2.0
Time
-0.00
0.50
1.00
1.50
2.00
2.50
3.00
3.50
4.00
4.50
5.00
5.50
6.00
6.50
7.00
7.50
8.00
8.50
9.00
9.50
10.00

{[3-(Benzyloxy)phenyl]methyl}(2-phenylethyl)amine (16):

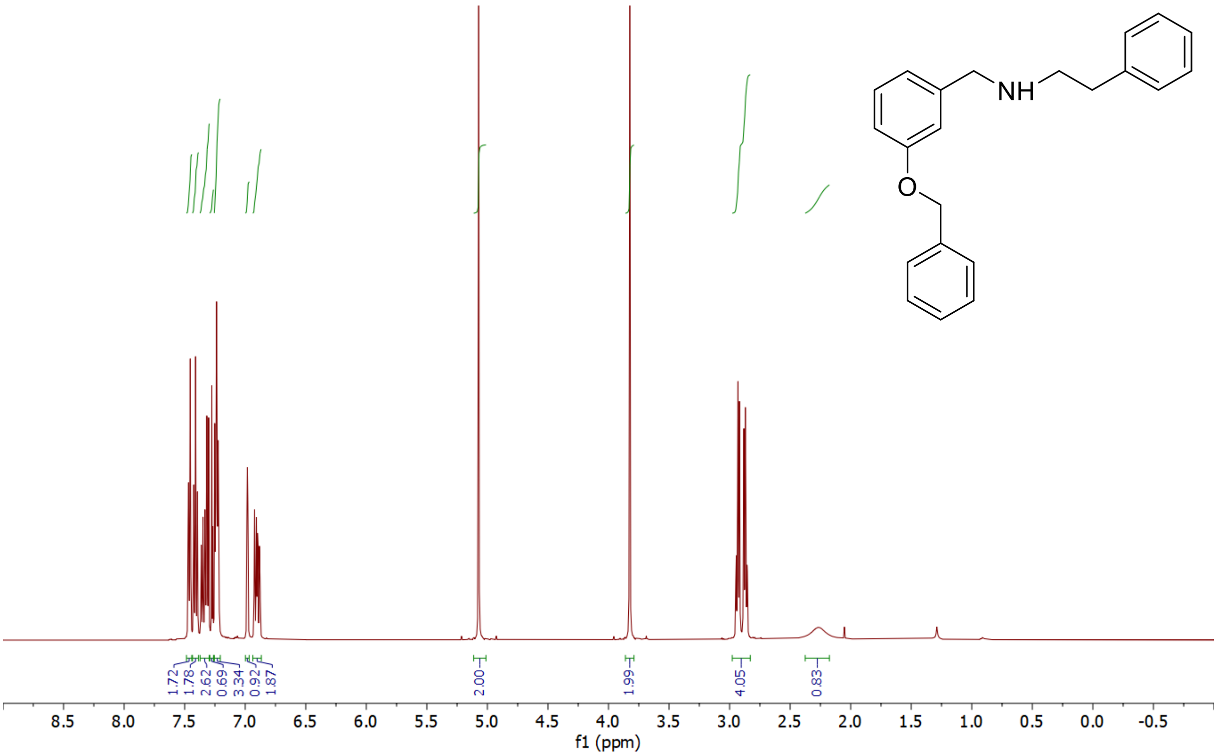

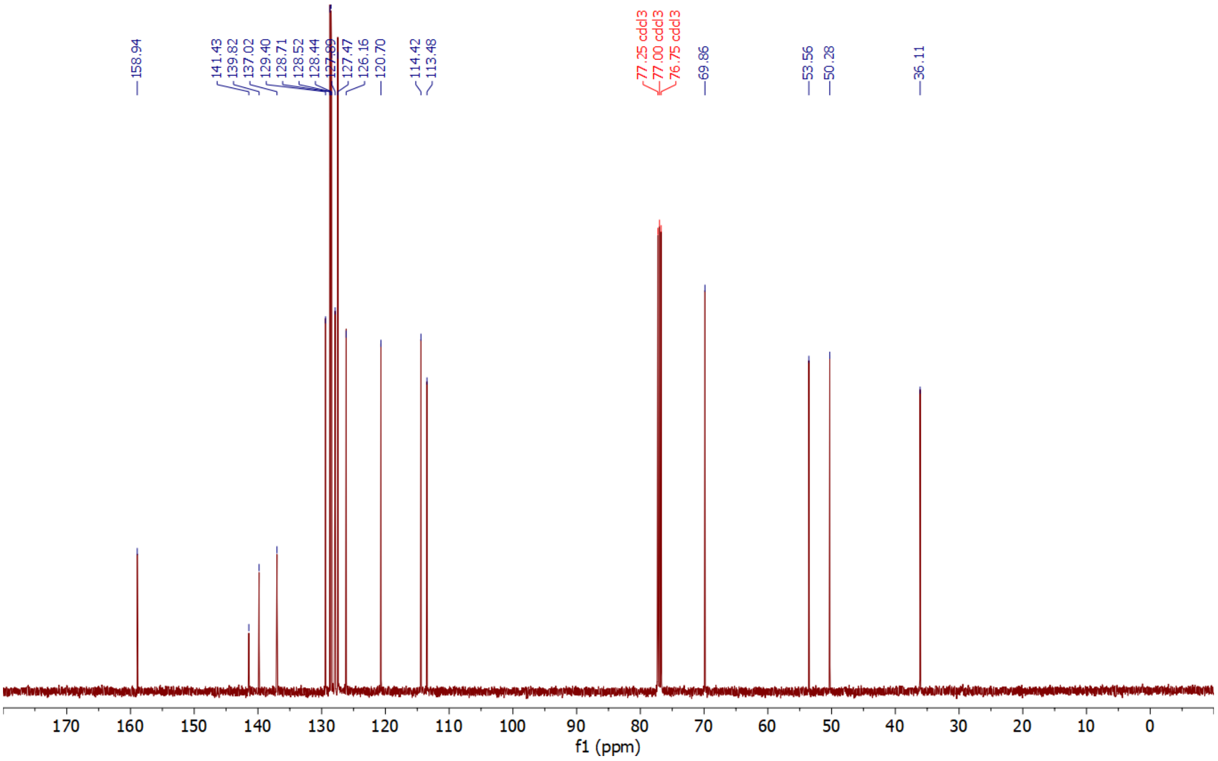

318,1852

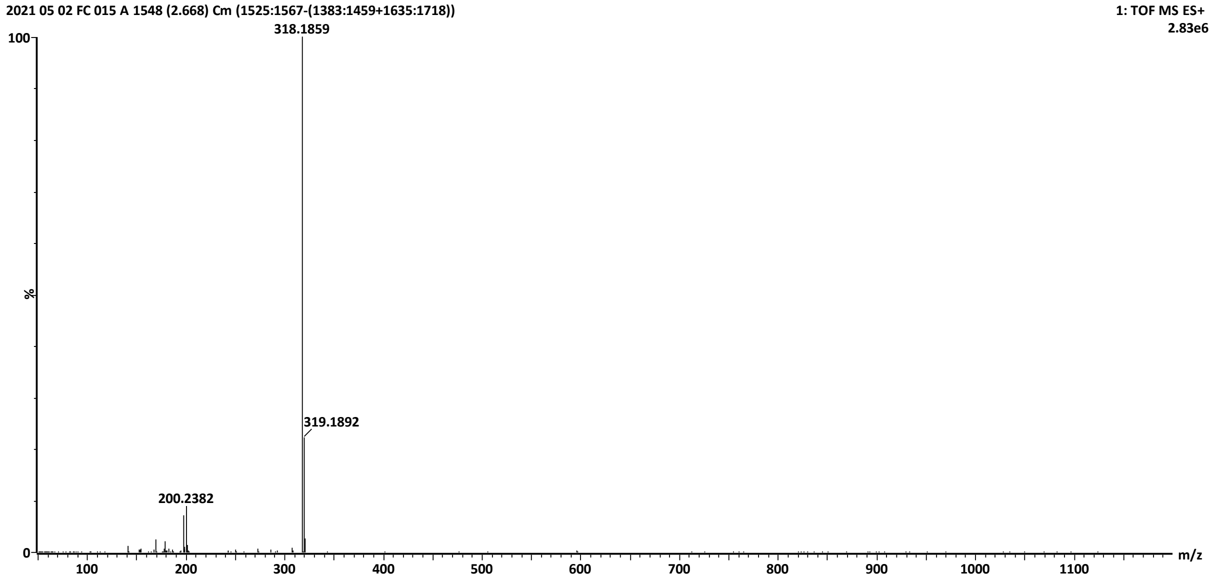
2021 05 02 FC 015 A 1548 (2.668) Cm (1525:1567-(1383:1459+1635:1718))
1: TOF MS ES+
2.83e6
318.1859
319.1892
200.2382
100
%
0
m/z
100
200
300
400
500
600
700
800
900
1000
1100

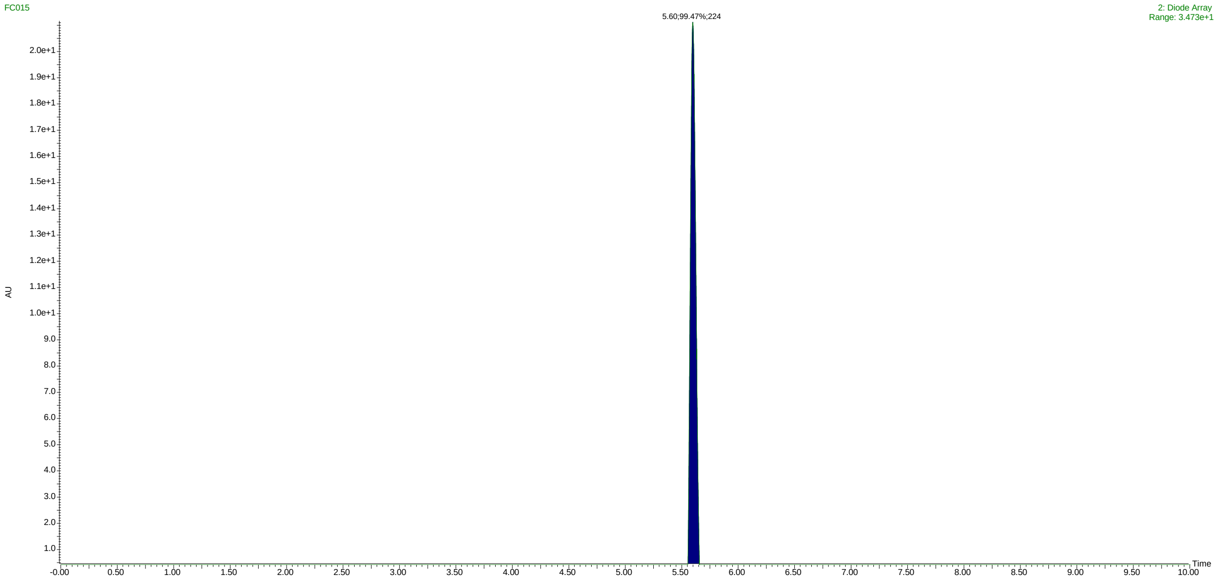
FC015
2: Diode Array
Range: 3.473e+1
5.60;99.47%;224
AU
Time

{[3-(Benzyloxy)phenyl]methyl}(3-phenylpropyl)amine (17):

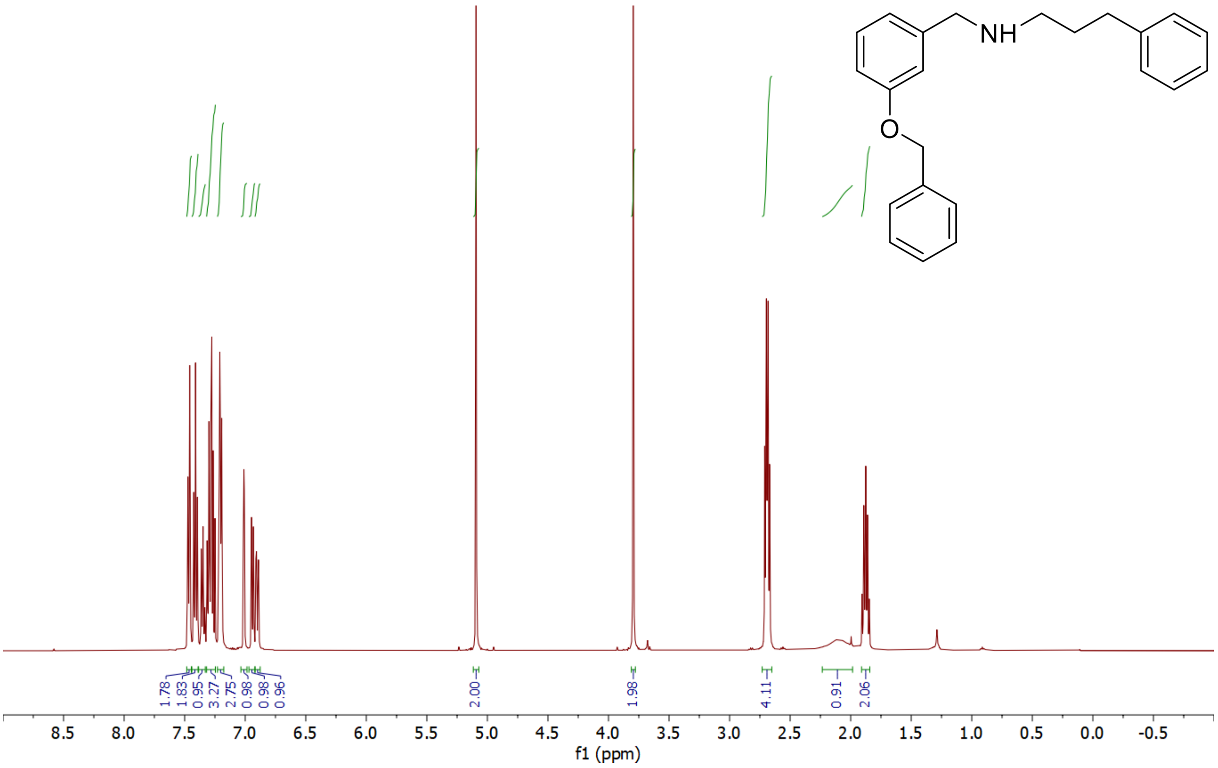

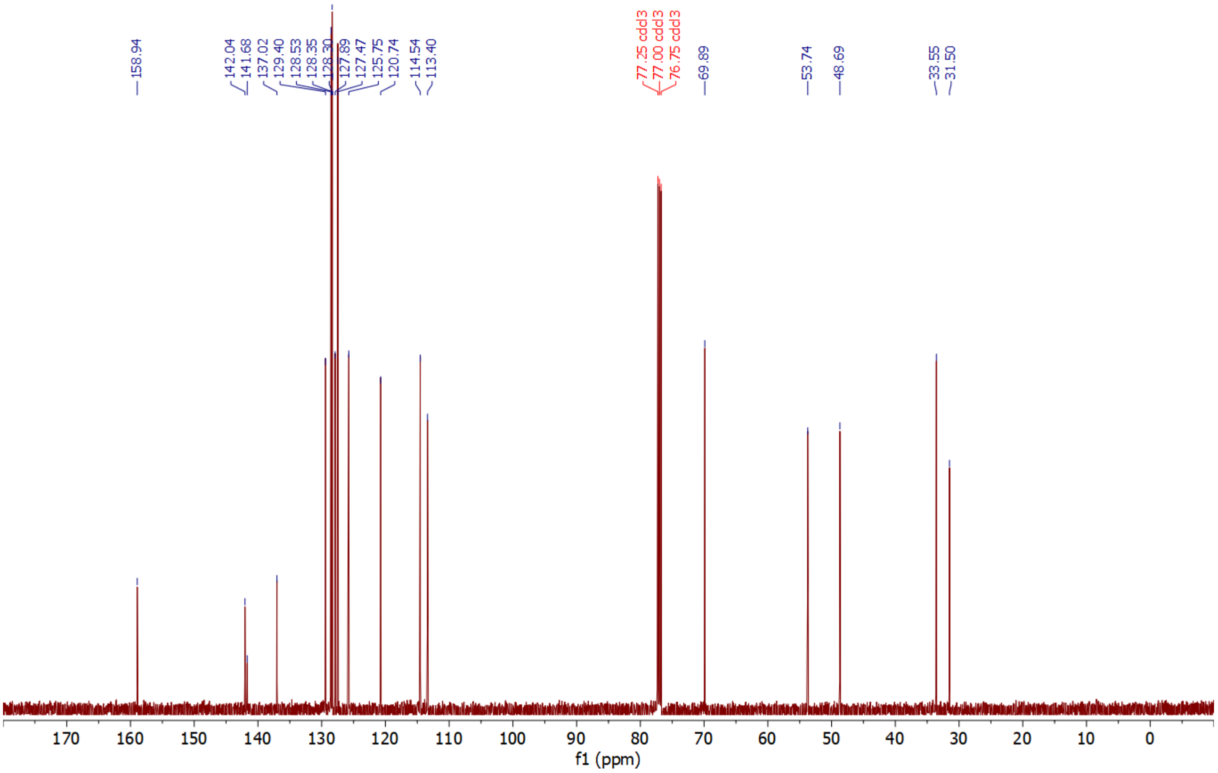

332,2009

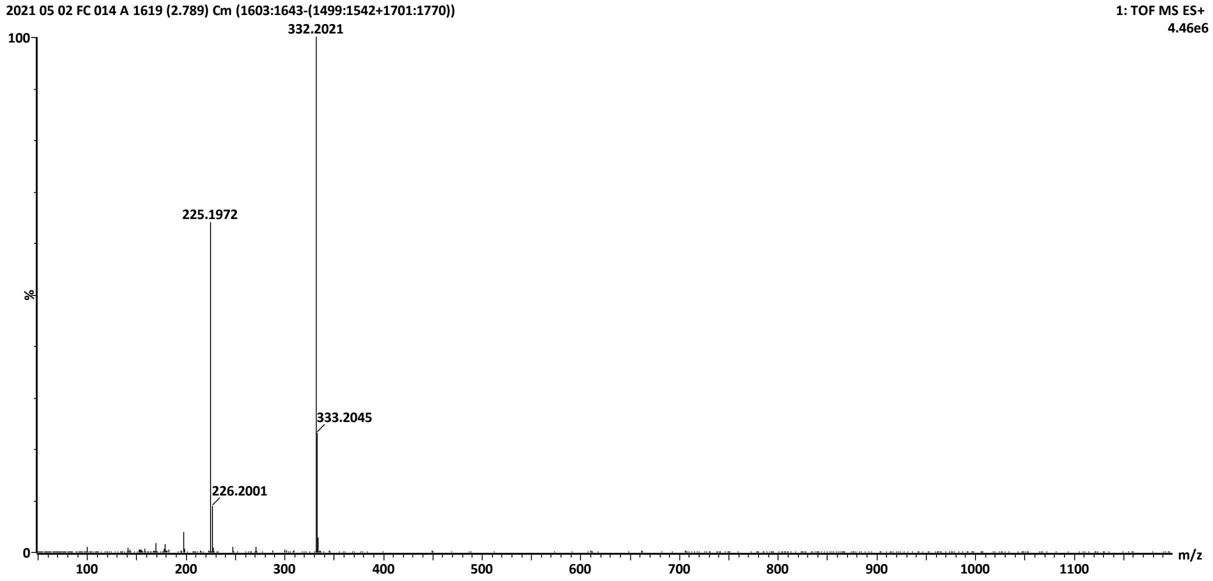
2021 05 02 FC 014 A 1619 (2.789) Cm (1603:1643-(1499:1542+1701:1770))
1: TOF MS ES+
4.46e6
332.2021
225.1972
333.2045
226.2001
%
m/z

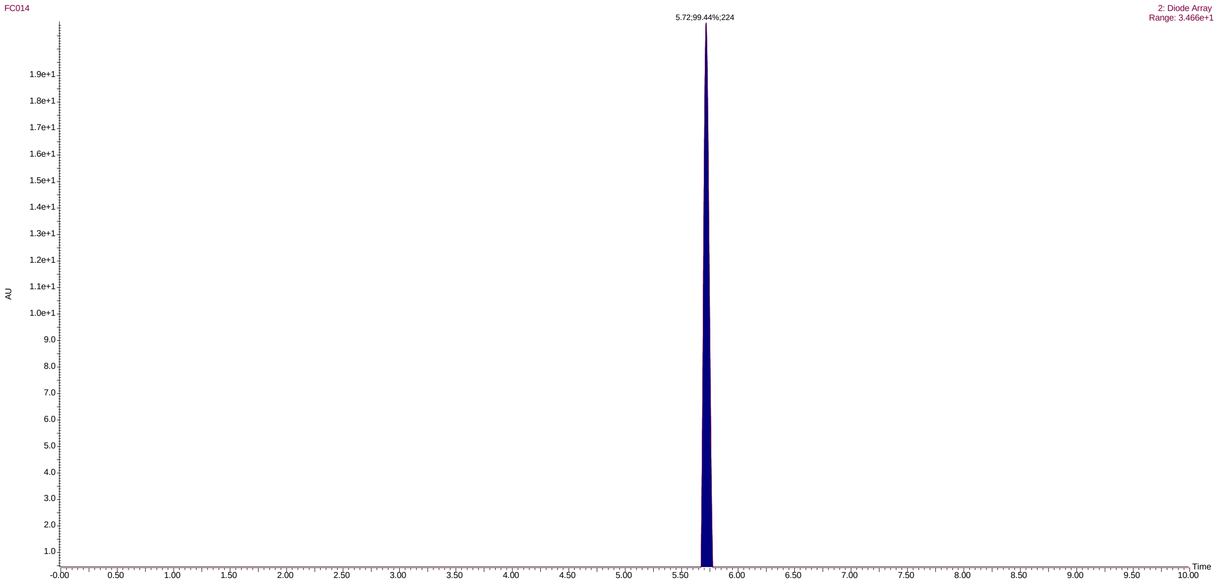
FC014
2: Diode Array
Range: 3.466e+1
5.72;99.44%;224
AU
Time

{[3-(Benzyloxy)phenyl]methyl}(2-phenylpropyl)amine (18):

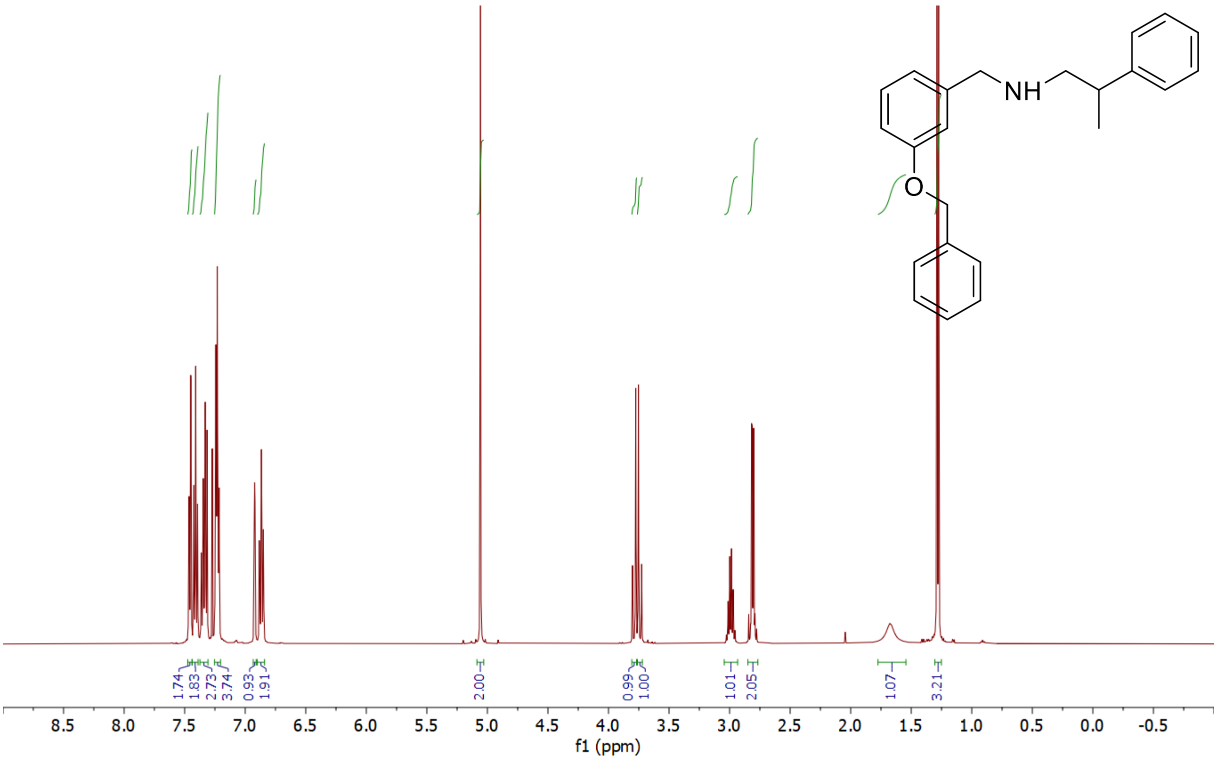

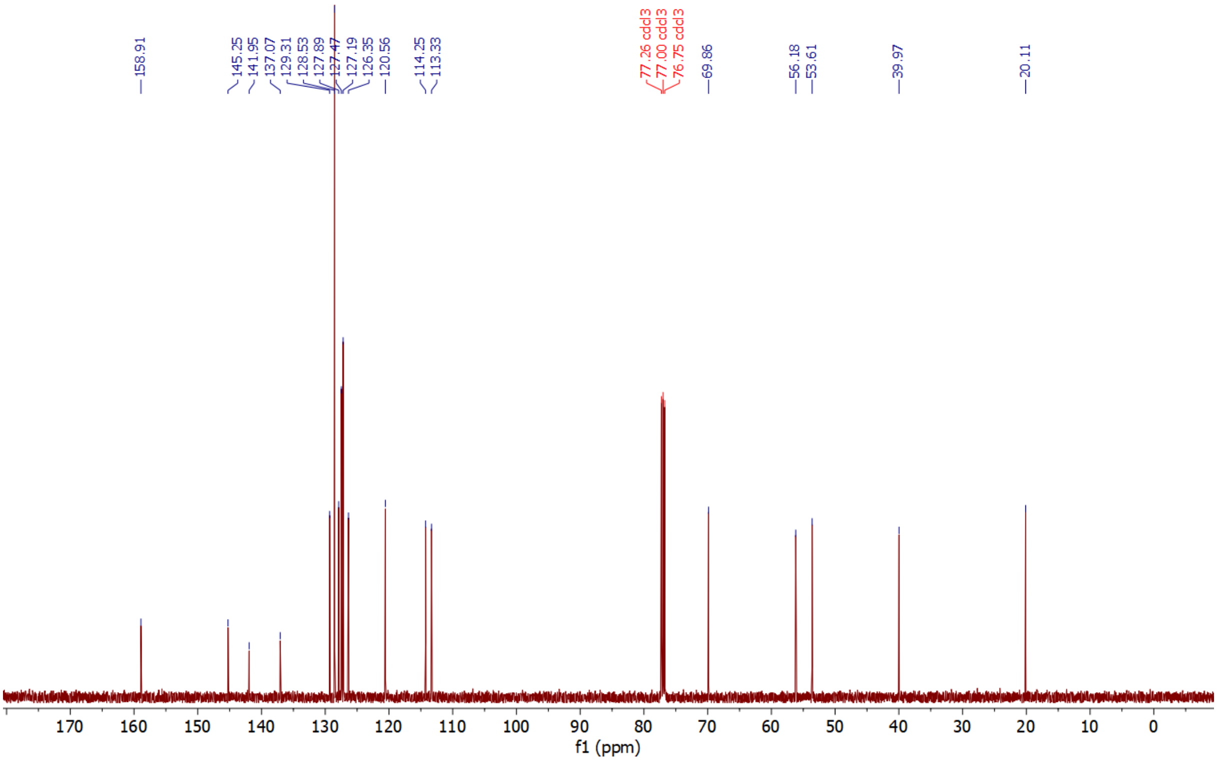

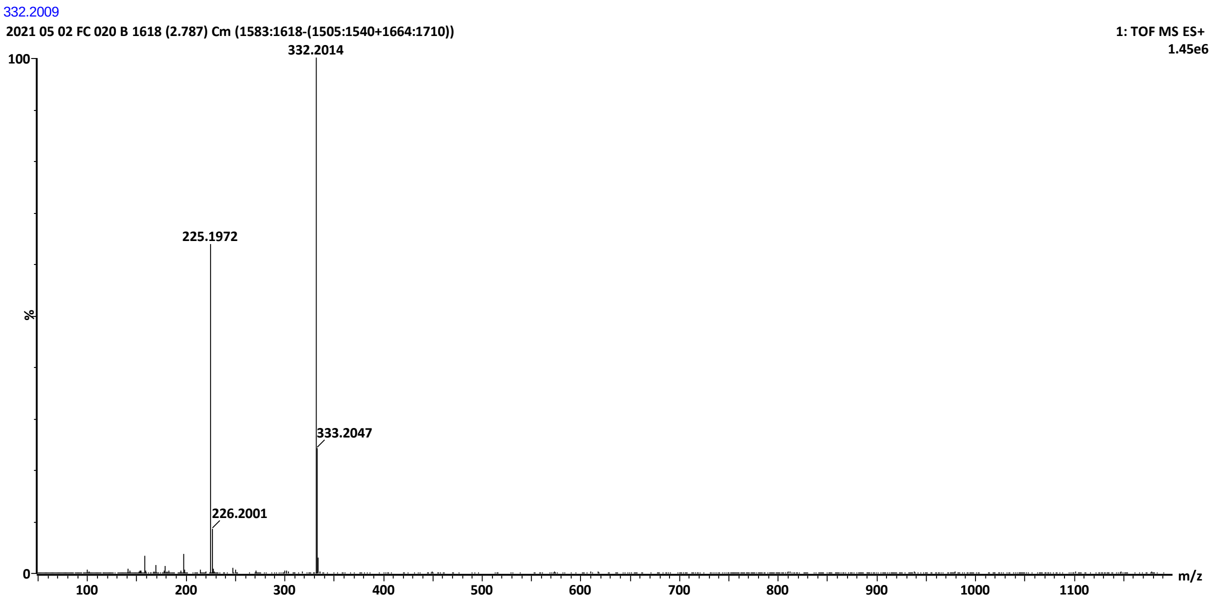
332.2009
2021 05 02 FC 020 B 1618 (2.787) Cm (1583:1618-(1505:1540+1664:1710))
1: TOF MS ES+
1.45e6
332.2014
225.1972
333.2047
226.2001
%
m/z

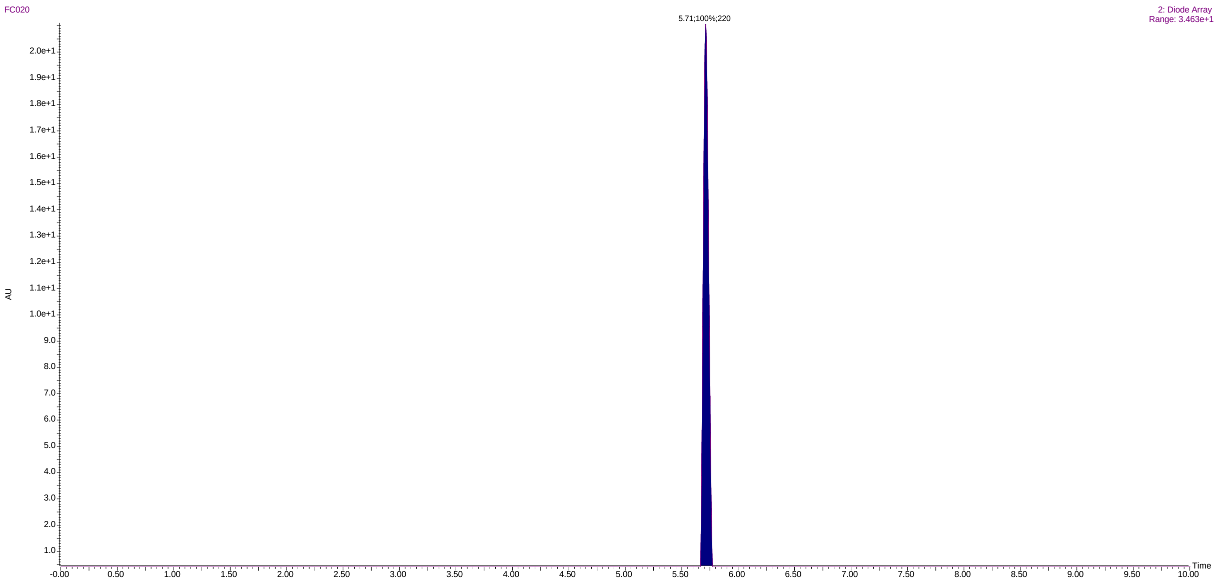
FC020
2: Diode Array
Range: 3.463e+1
5.71;100%;220
AU
Time

Benzyl({[3-(benzyloxy)phenyl]methyl})ethylamine (19):

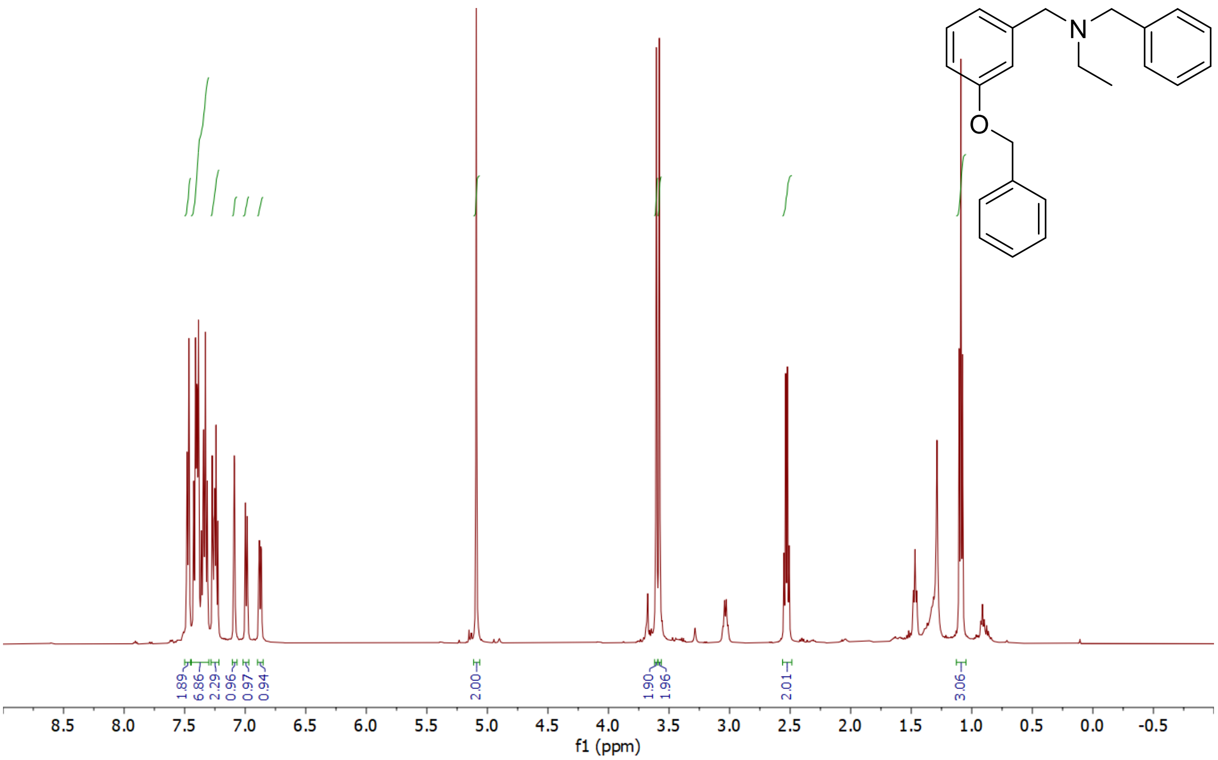

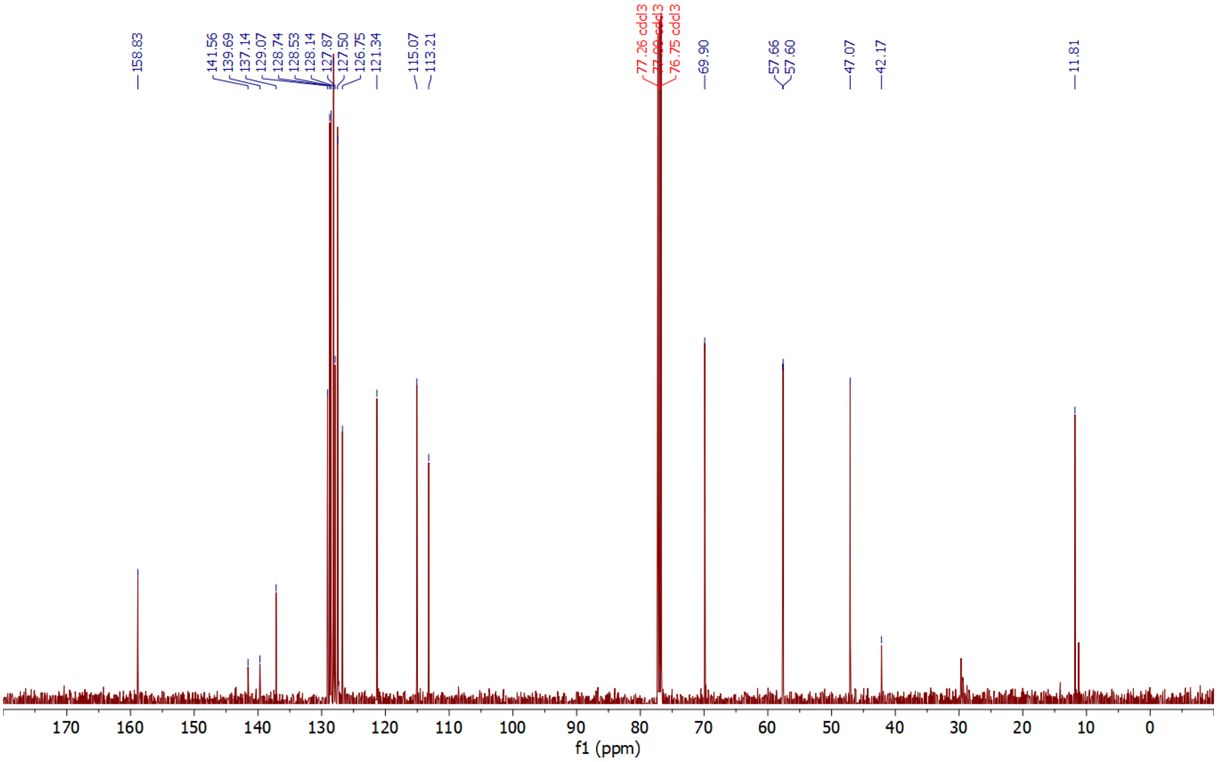

332.2009

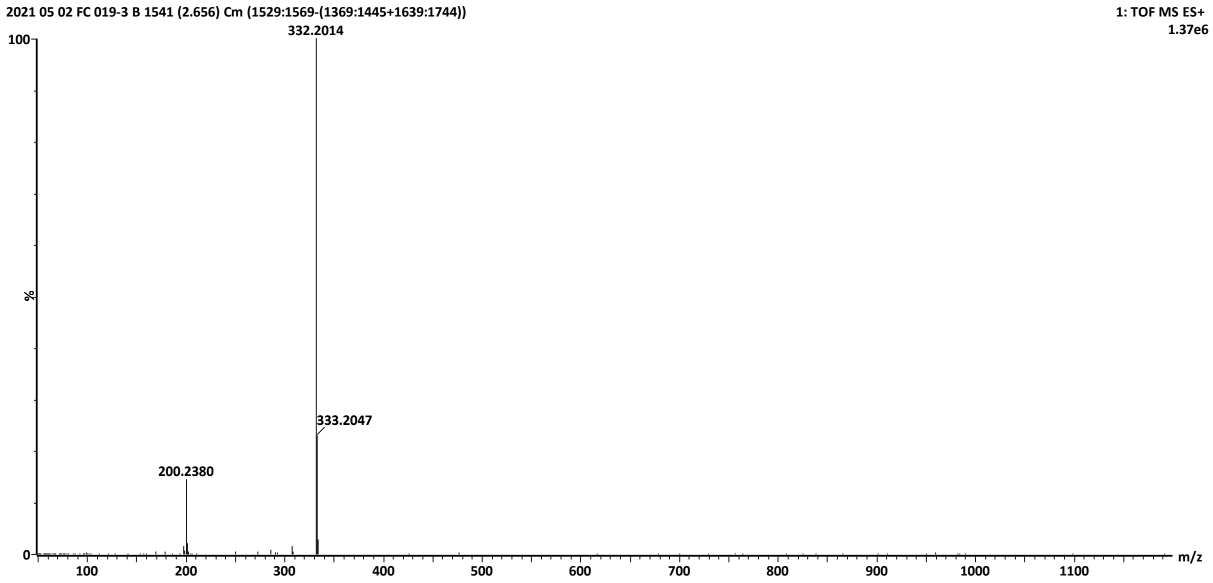
2021 05 02 FC 019-3 B 1541 (2.656) Cm (1529:1569-(1369:1445+1639:1744))
1: TOF MS ES+
1.37e6
332.2014
333.2047
200.2380
100
%
0
m/z
100
200
300
400
500
600
700
800
900
1000
1100

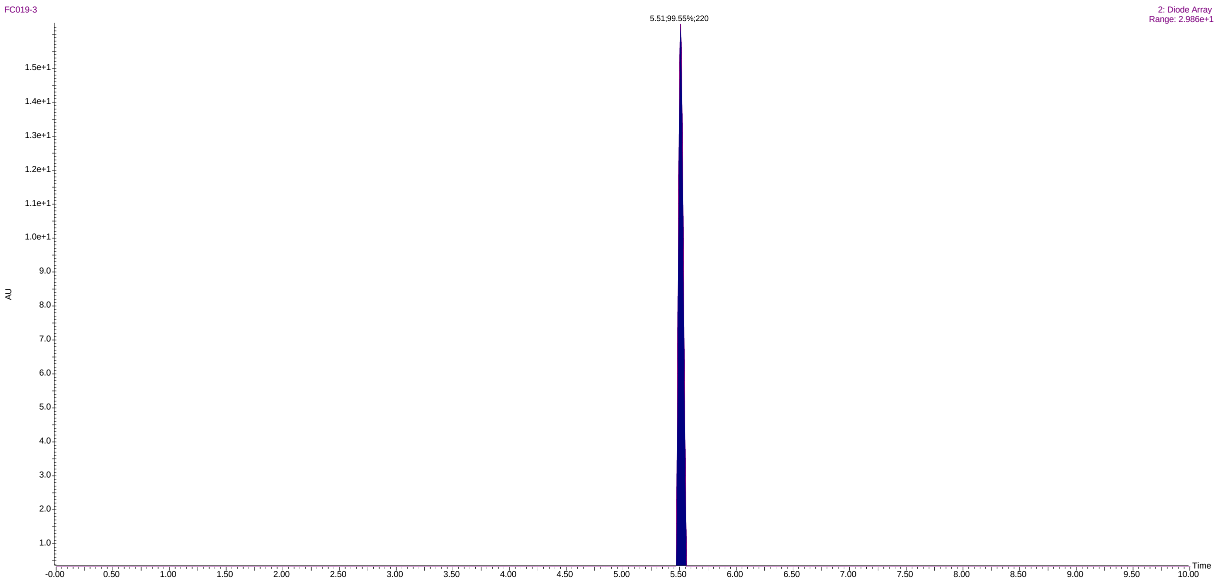
FC019-3
2: Diode Array
Range: 2.986e+1
5.51;99.55%;220
AU
1.5e+1
1.4e+1
1.3e+1
1.2e+1
1.1e+1
1.0e+1
9.0
8.0
7.0
6.0
5.0
4.0
3.0
2.0
1.0
Time
-0.00
0.50
1.00
1.50
2.00
2.50
3.00
3.50
4.00
4.50
5.00
5.50
6.00
6.50
7.00
7.50
8.00
8.50
9.00
9.50
10.00

Benzyl({[3-(benzyloxy)phenyl]methyl})(prop-2-en-1-yl)amine (20):

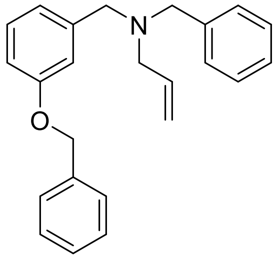

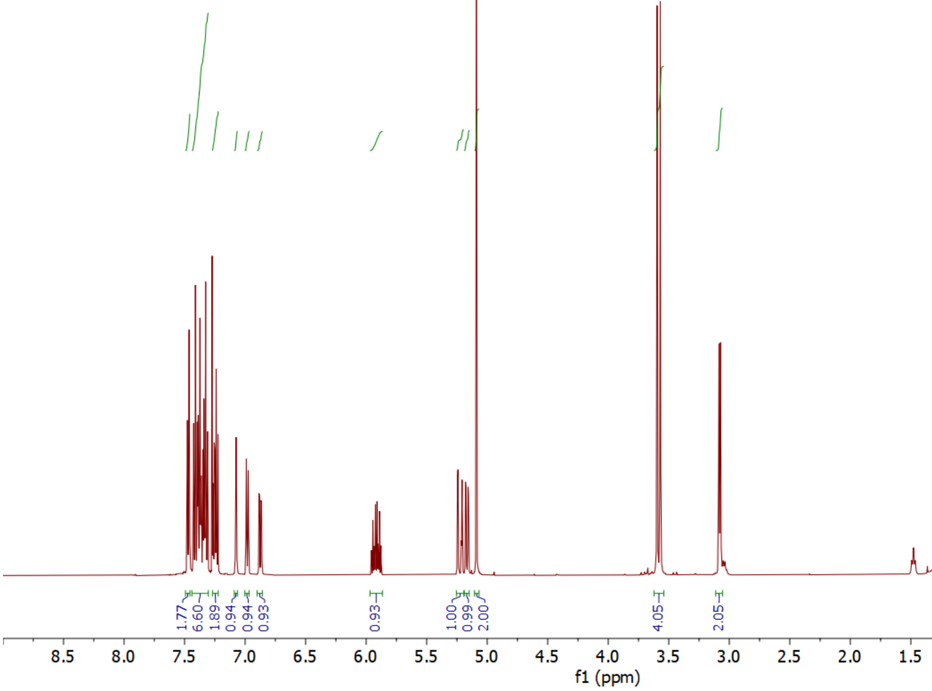

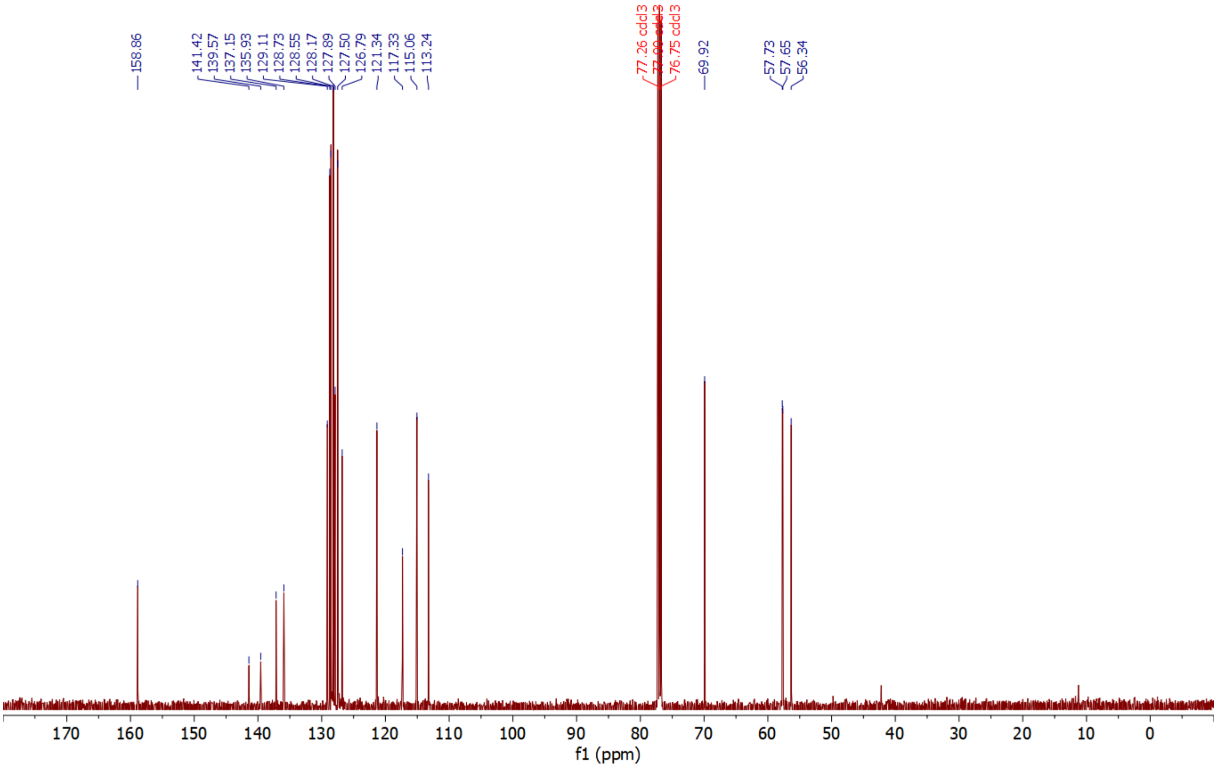

344,2009

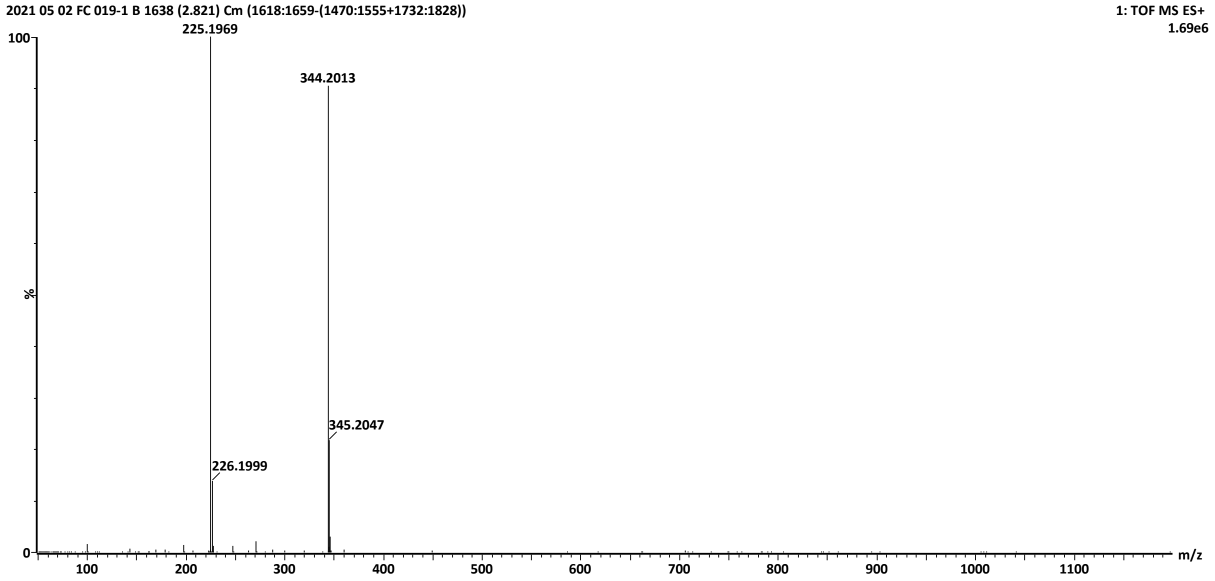
2021 05 02 FC 019-1 B 1638 (2.821) Cm (1618:1659-(1470:1555+1732:1828))
1: TOF MS ES+
1.69e6
225.1969
344.2013
345.2047
226.1999
%
m/z

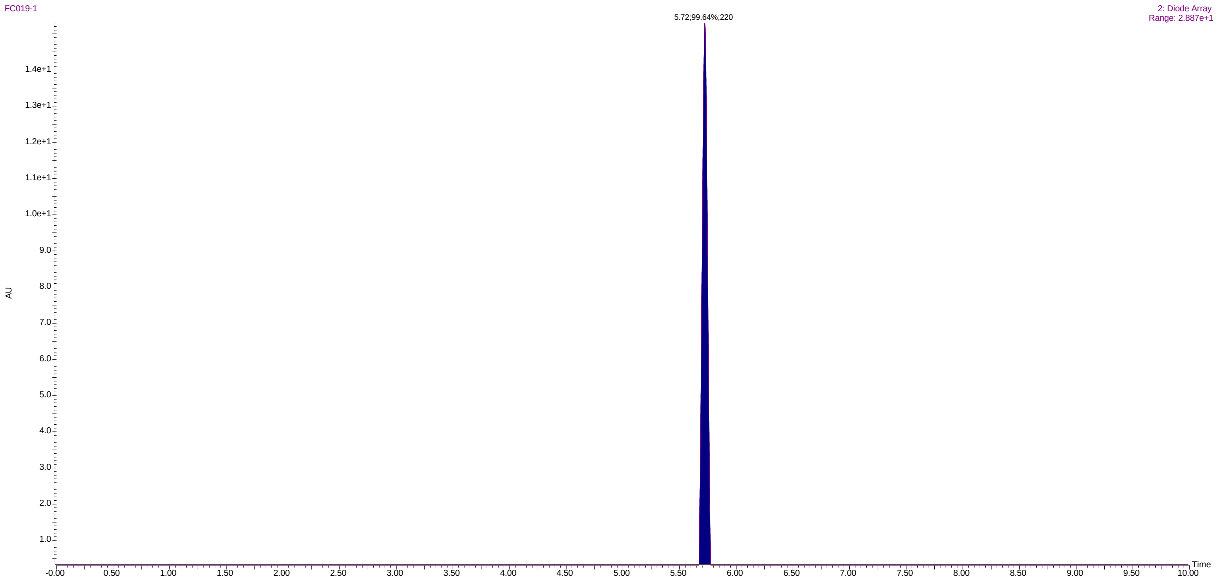
FC019-1
2: Diode Array
Range: 2.887e+1
5.72;99.64%;220
AU
Time

{[3-(Benzyloxy)phenyl]methyl}(ethyl)(2-phenylethyl)amine (21):

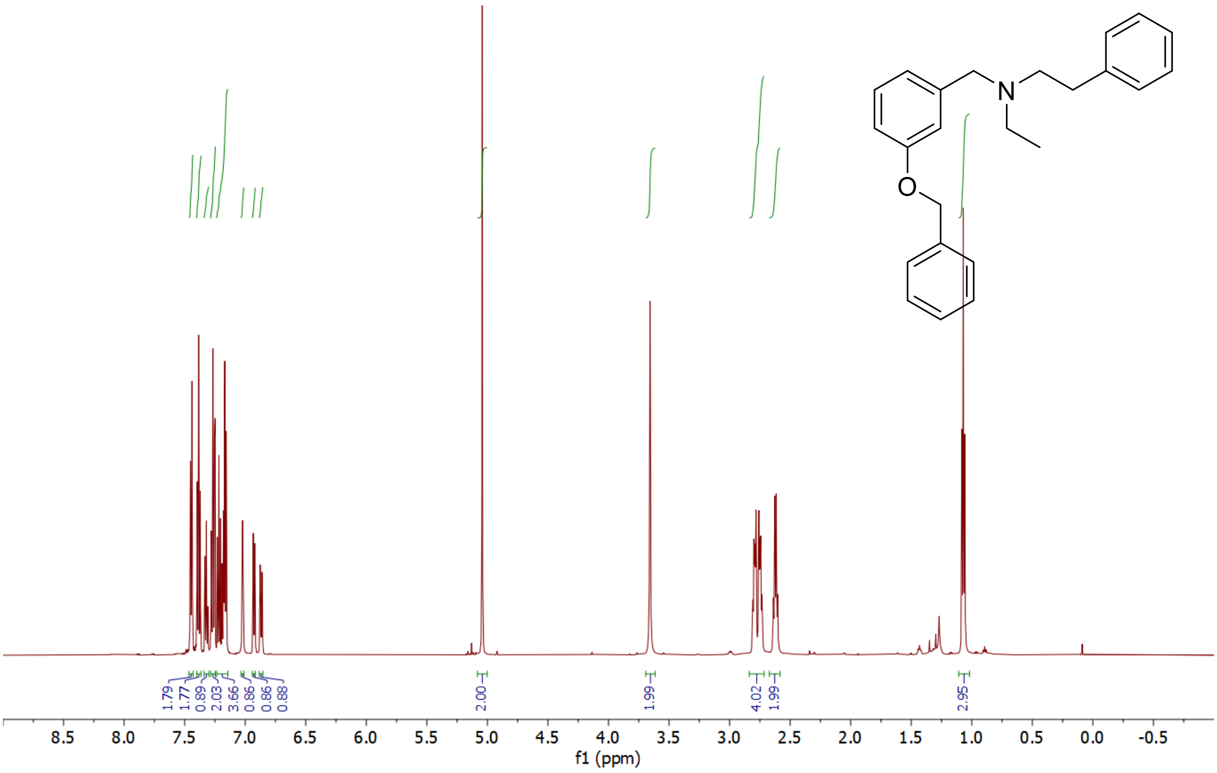

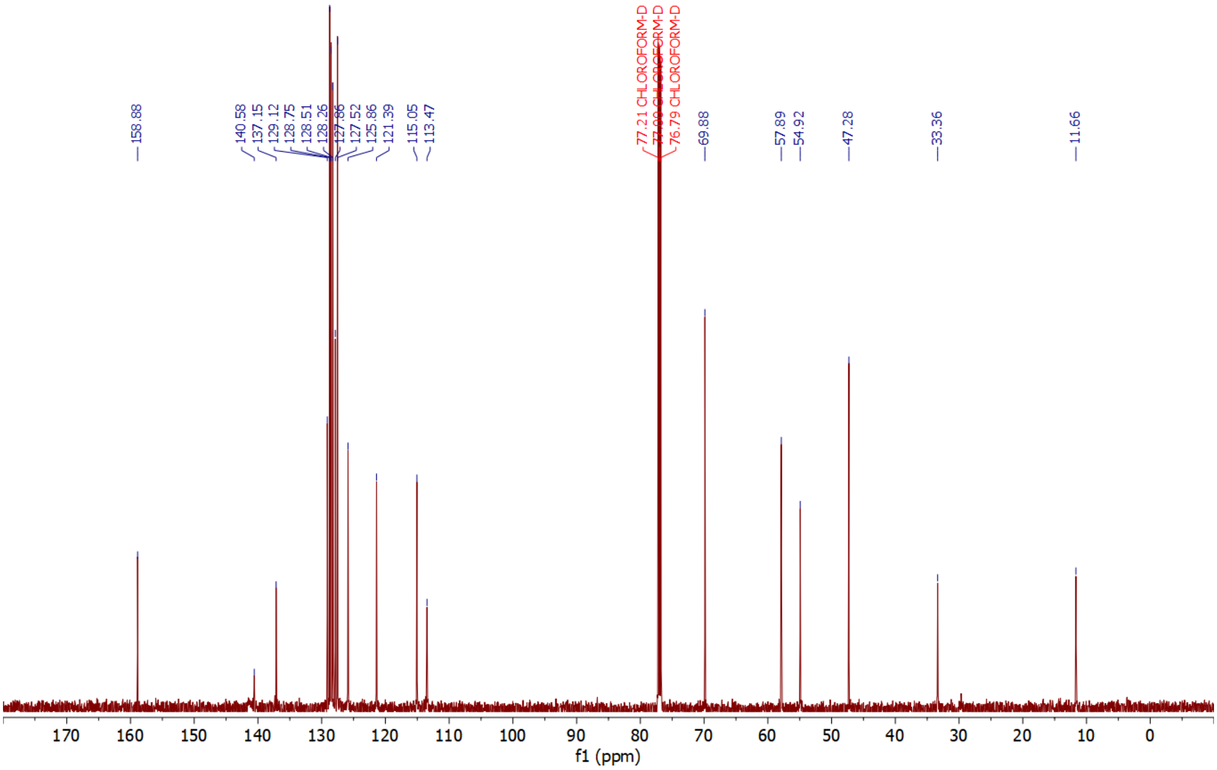

346.2165

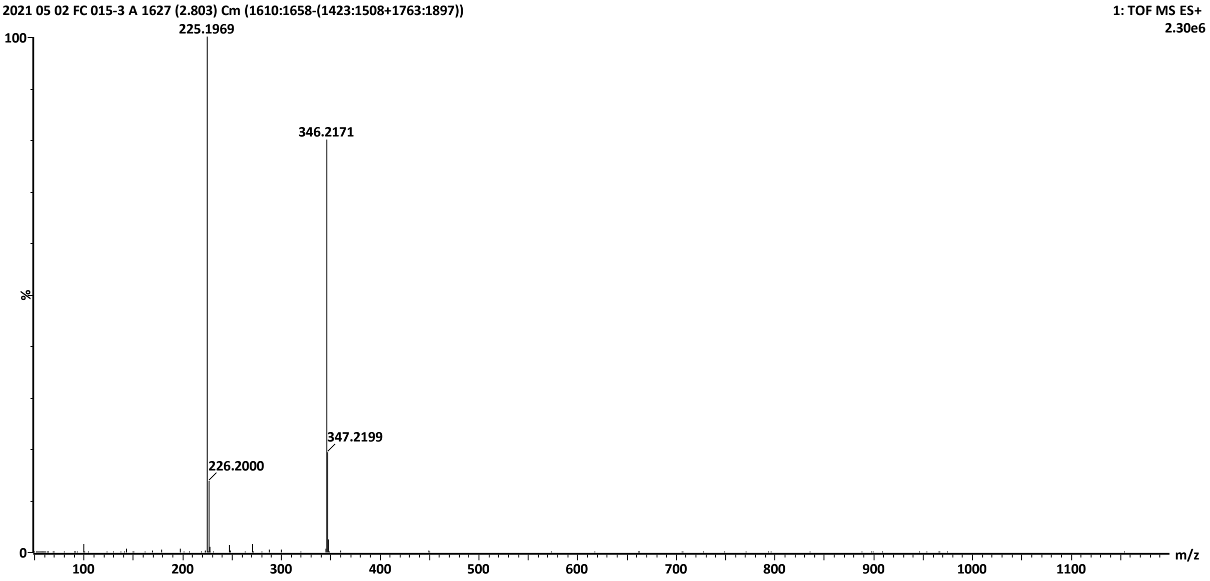
2021 05 02 FC 015-3 A 1627 (2.803) Cm (1610:1658-(1423:1508+1763:1897))
1: TOF MS ES+
2.30e6
225.1969
346.2171
347.2199
226.2000
100
%
0
m/z
100
200
300
400
500
600
700
800
900
1000
1100

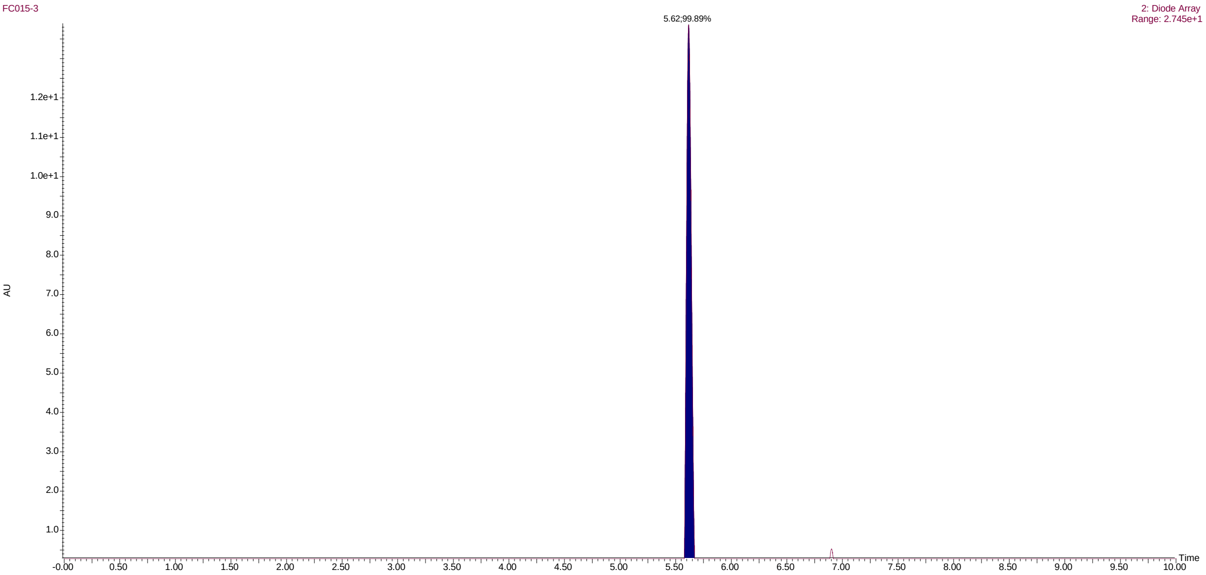
FC015-3
2: Diode Array
Range: 2.745e+1
5.62;99.89%
AU
Time

{[3-(Benzyloxy)phenyl]methyl}(2-phenylethyl)propylamine (22):

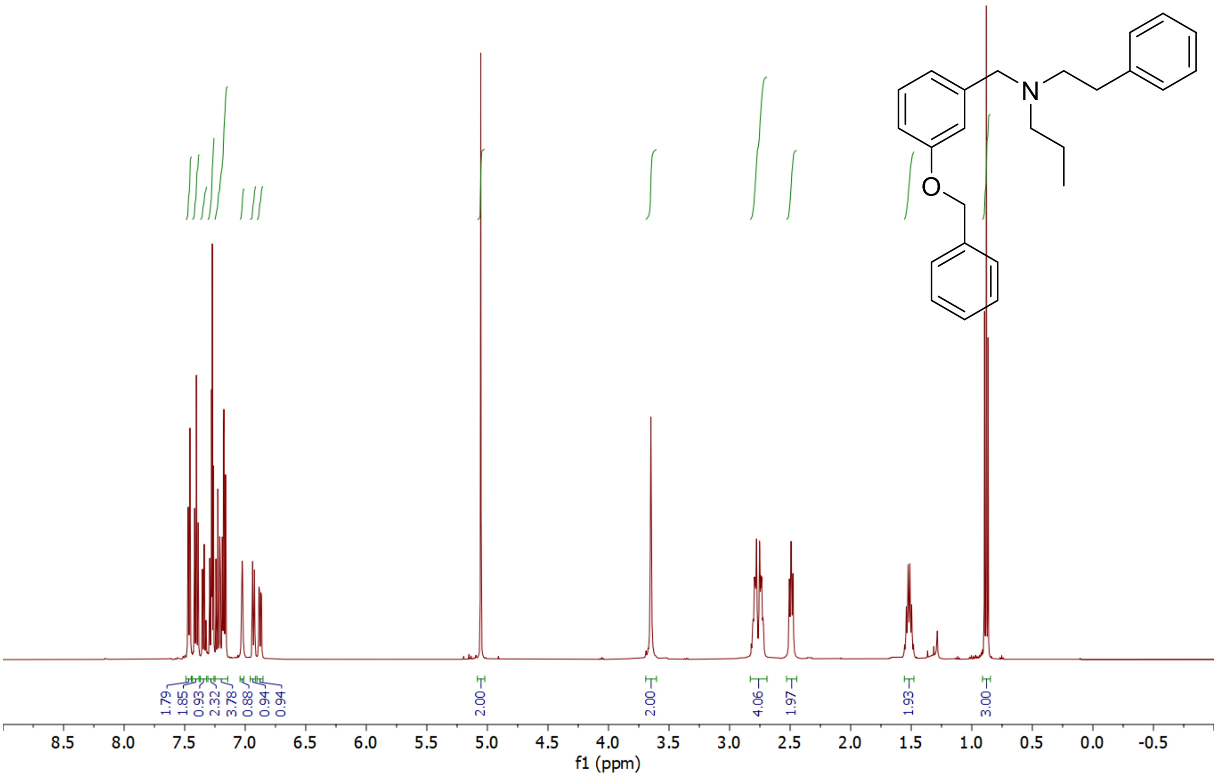

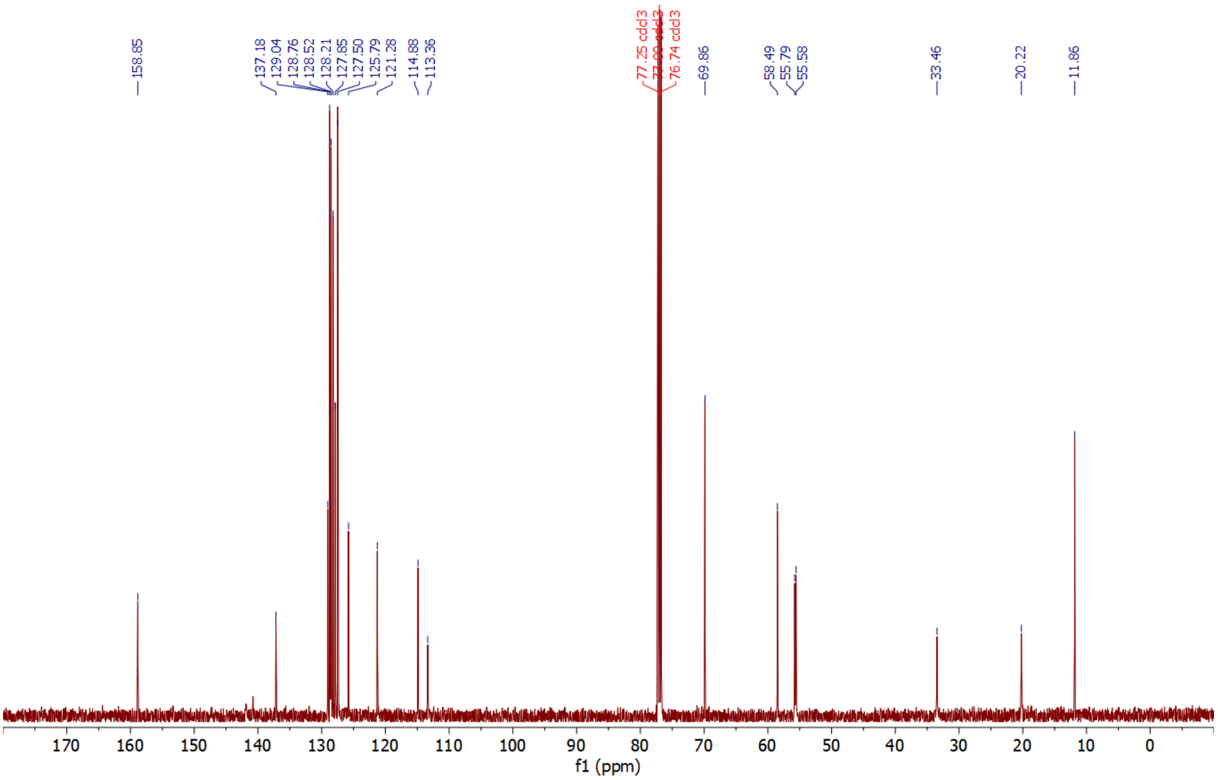

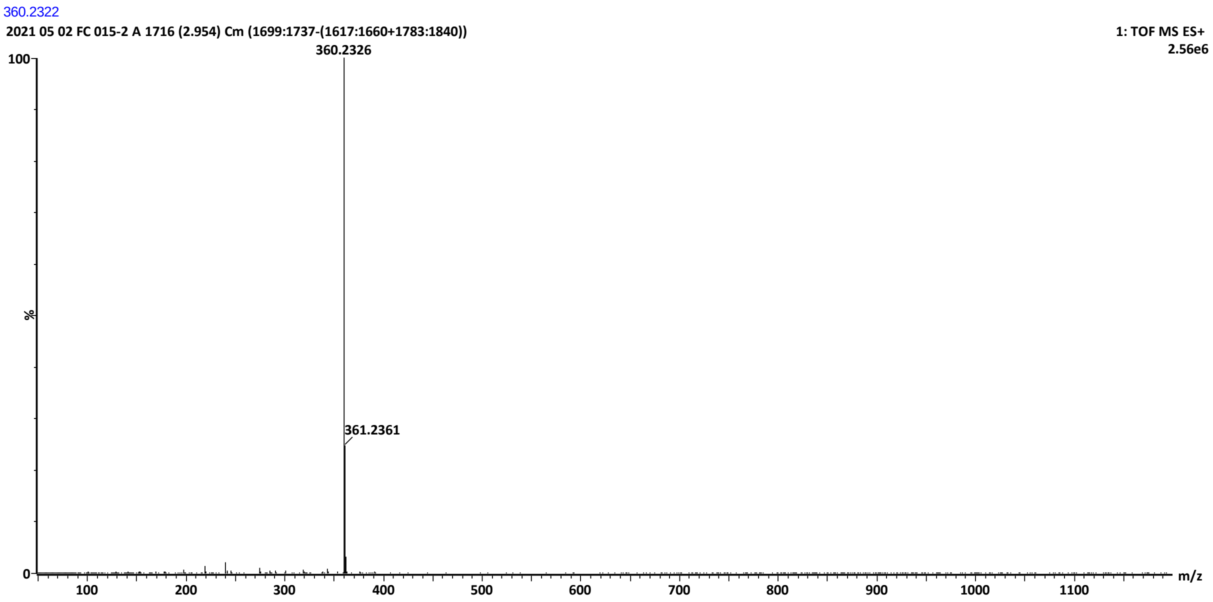
360.2322
2021 05 02 FC 015-2 A 1716 (2.954) Cm (1699:1737-(1617:1660+1783:1840))
1: TOF MS ES+
2.56e6
360.2326
361.2361
100
%
0
m/z
100
200
300
400
500
600
700
800
900
1000
1100

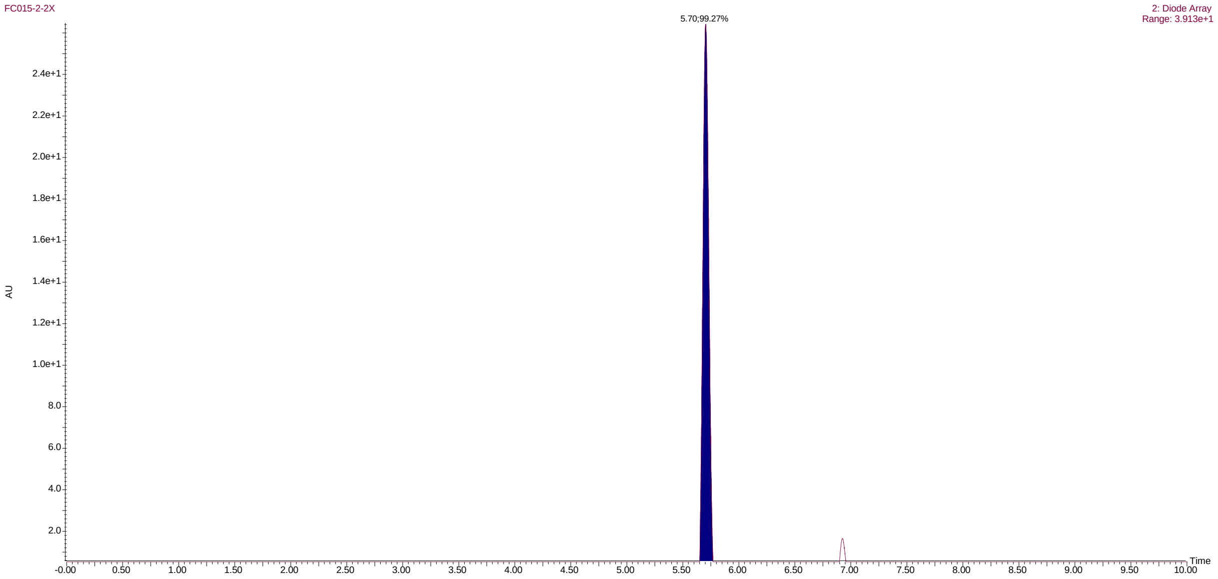
FC015-2-2X
2: Diode Array
Range: 3.913e+1
5.70;99.27%
AU
2.4e+1
2.2e+1
2.0e+1
1.8e+1
1.6e+1
1.4e+1
1.2e+1
1.0e+1
8.0
6.0
4.0
2.0
Time
-0.00
0.50
1.00
1.50
2.00
2.50
3.00
3.50
4.00
4.50
5.00
5.50
6.00
6.50
7.00
7.50
8.00
8.50
9.00
9.50
10.00

{[3-(Benzyloxy)phenyl]methyl}(2-phenylethyl)(prop-2-en-1-yl)amine (23):

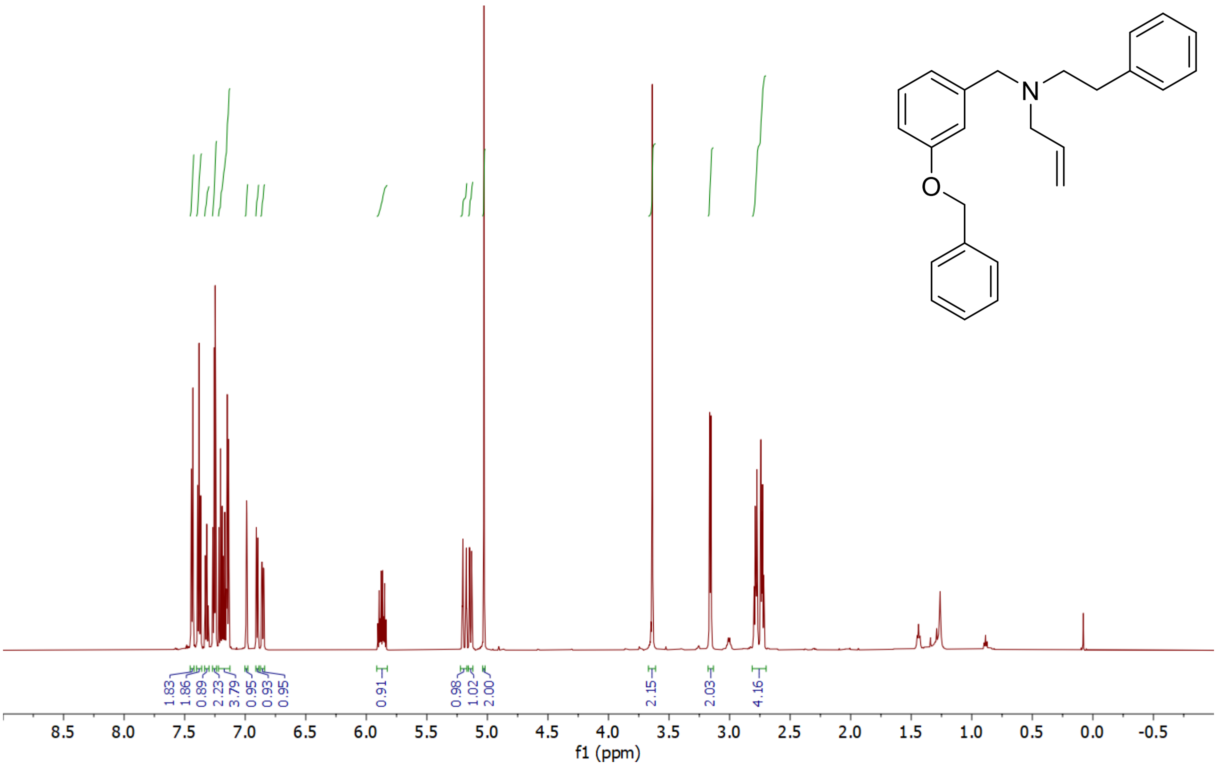

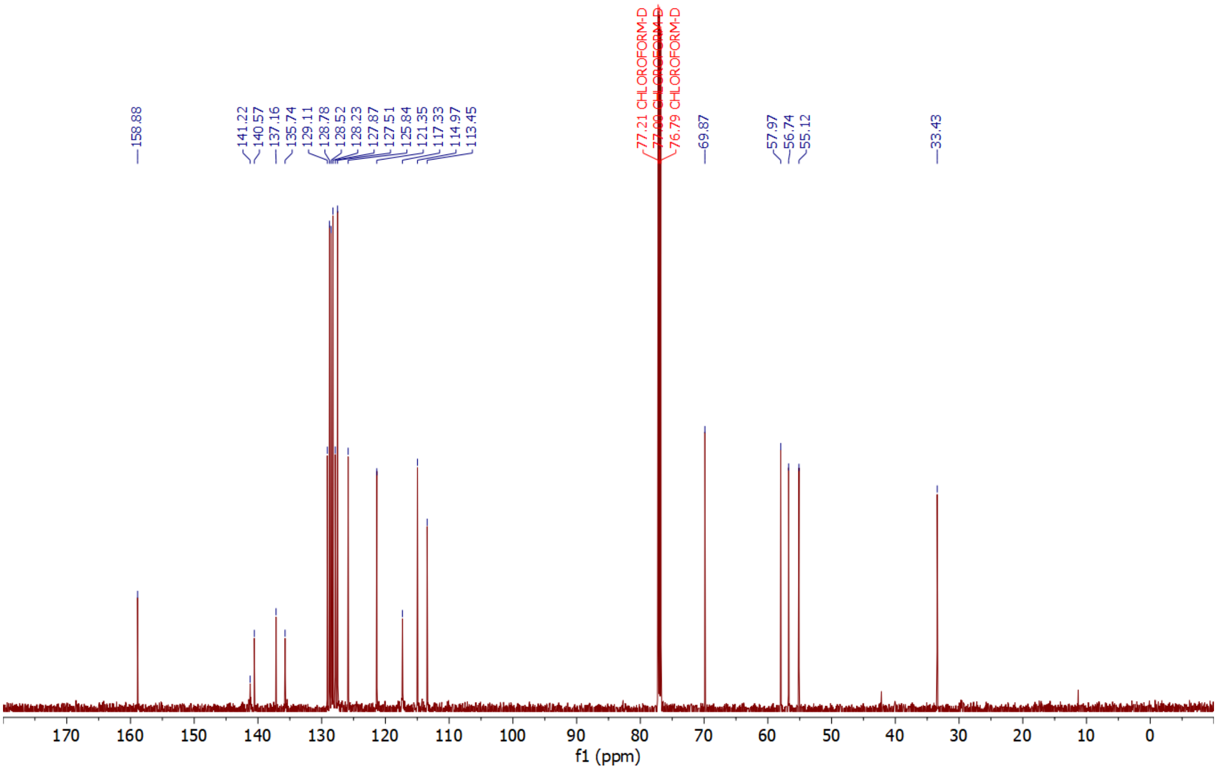

358,2165

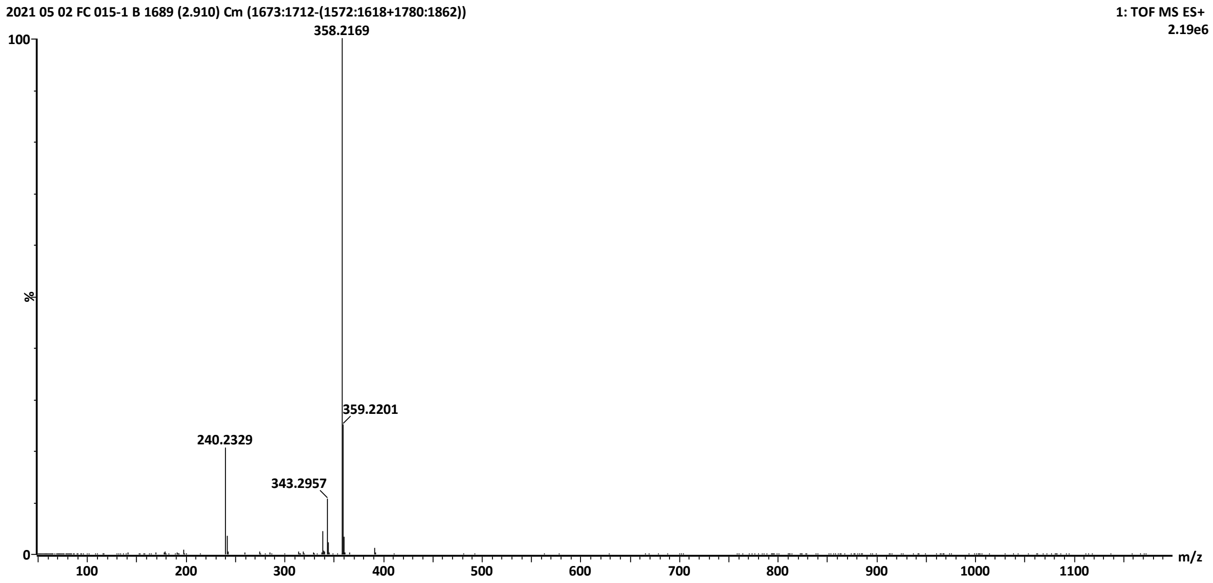
2021 05 02 FC 015-1 B 1689 (2.910) Cm (1673:1712-(1572:1618+1780:1862))
1: TOF MS ES+
2.19e6
358.2169
359.2201
240.2329
343.2957
100
%
0
m/z
100
200
300
400
500
600
700
800
900
1000
1100

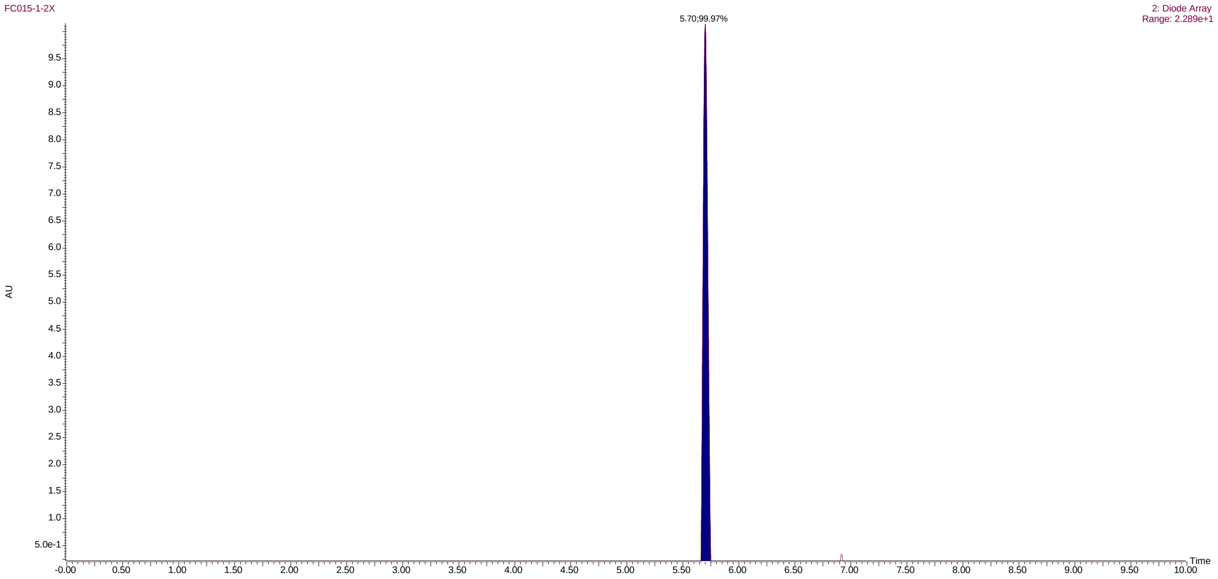
FC015-1-2X
2: Diode Array
Range: 2.289e+1
5.70;99.97%
AU
Time

{[3-(Benzyloxy)phenyl]methyl}(ethyl)(3-phenylpropyl)amine (24):

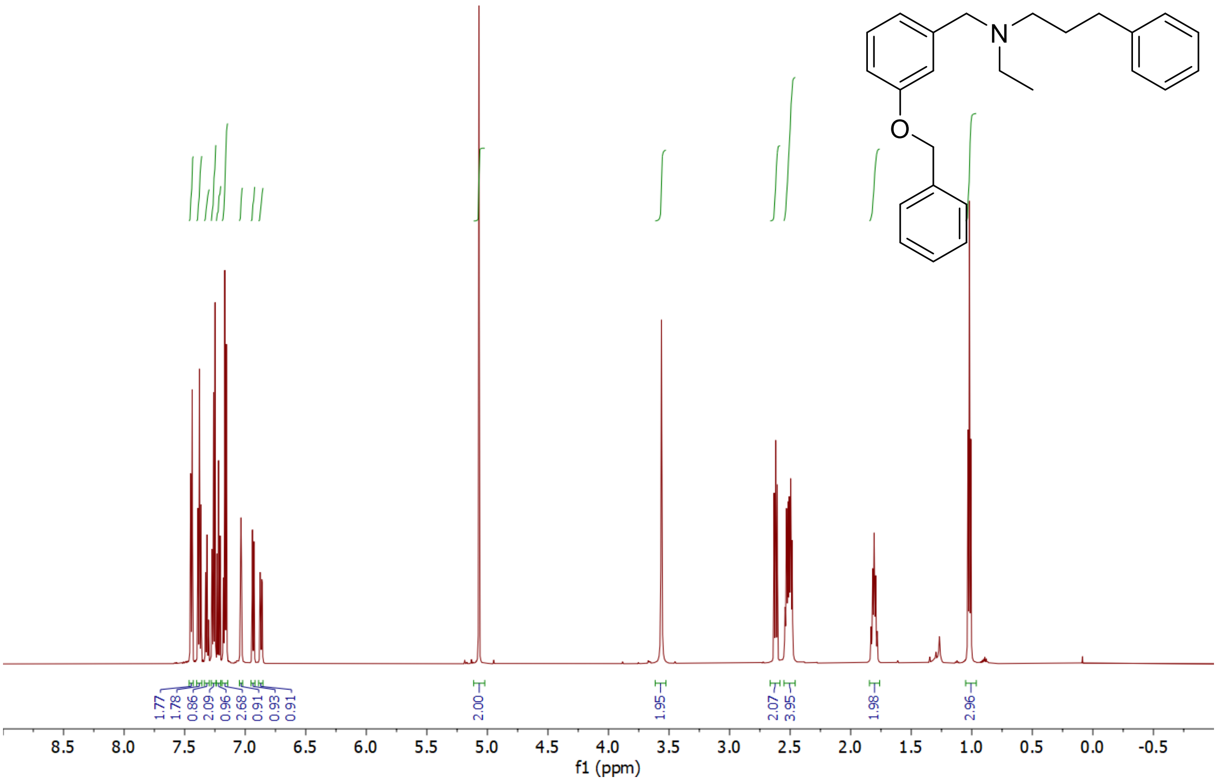

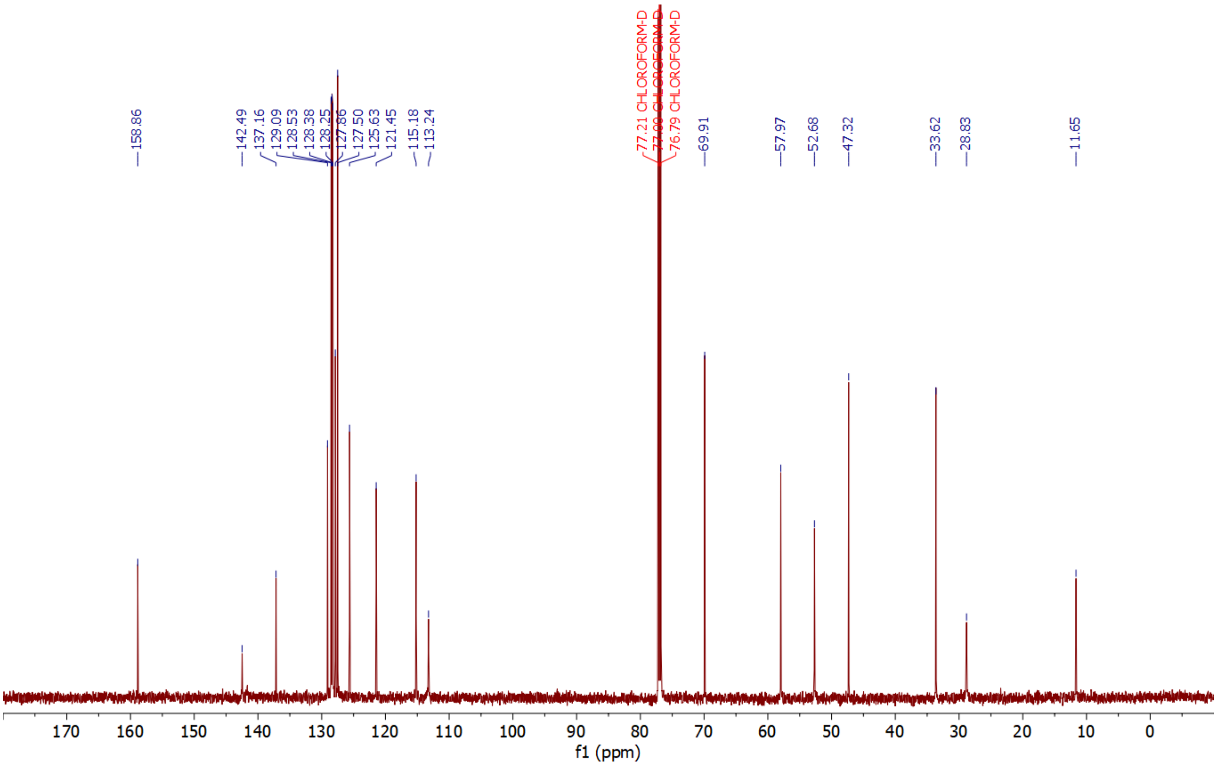

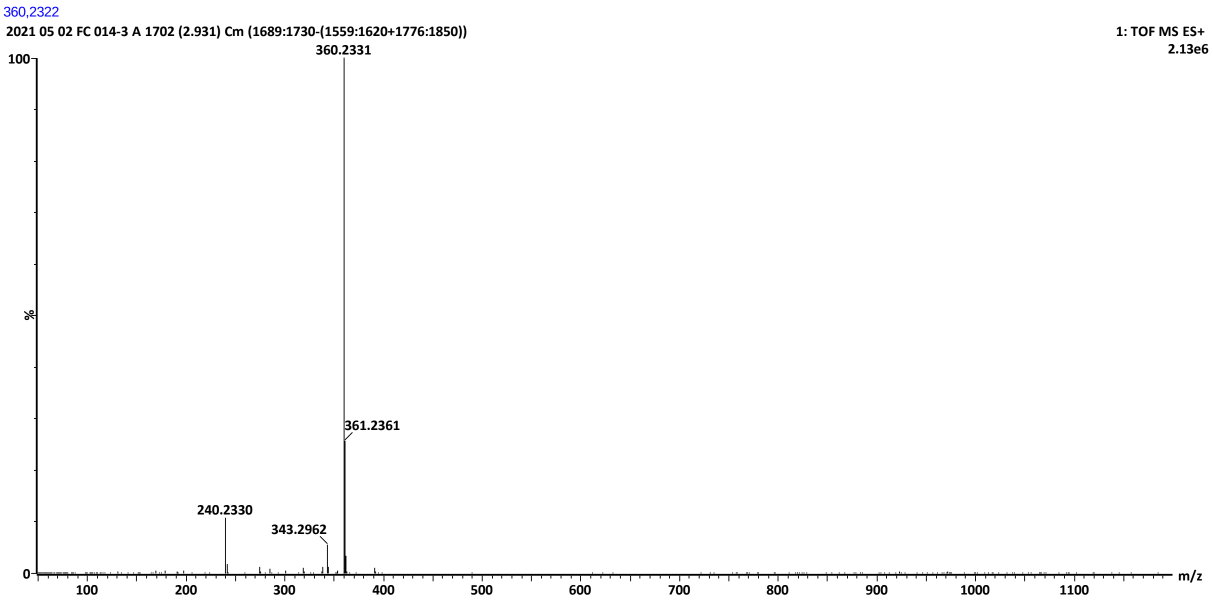
360,2322
2021 05 02 FC 014-3 A 1702 (2.931) Cm (1689:1730-(1559:1620+1776:1850))
1: TOF MS ES+
2.13e6
360.2331
361.2361
240.2330
343.2962
%
m/z

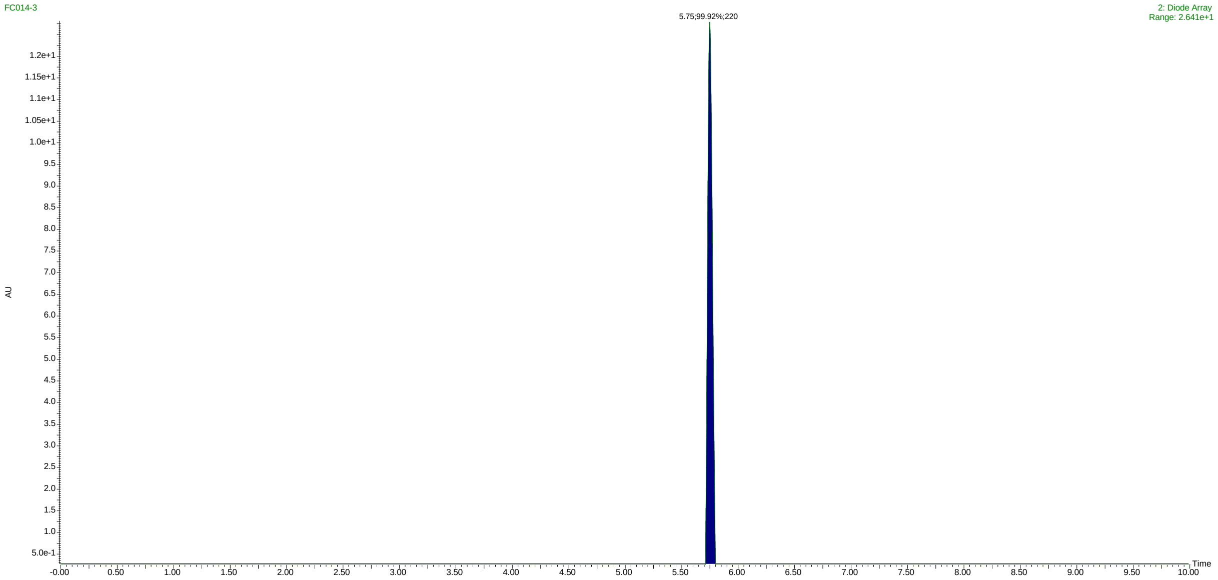
FC014-3
2: Diode Array
Range: 2.641e+1
5.75;99.92%;220
AU
Time

{[3-(Benzyloxy)phenyl]methyl}(3-phenylpropyl)propylamine (25):

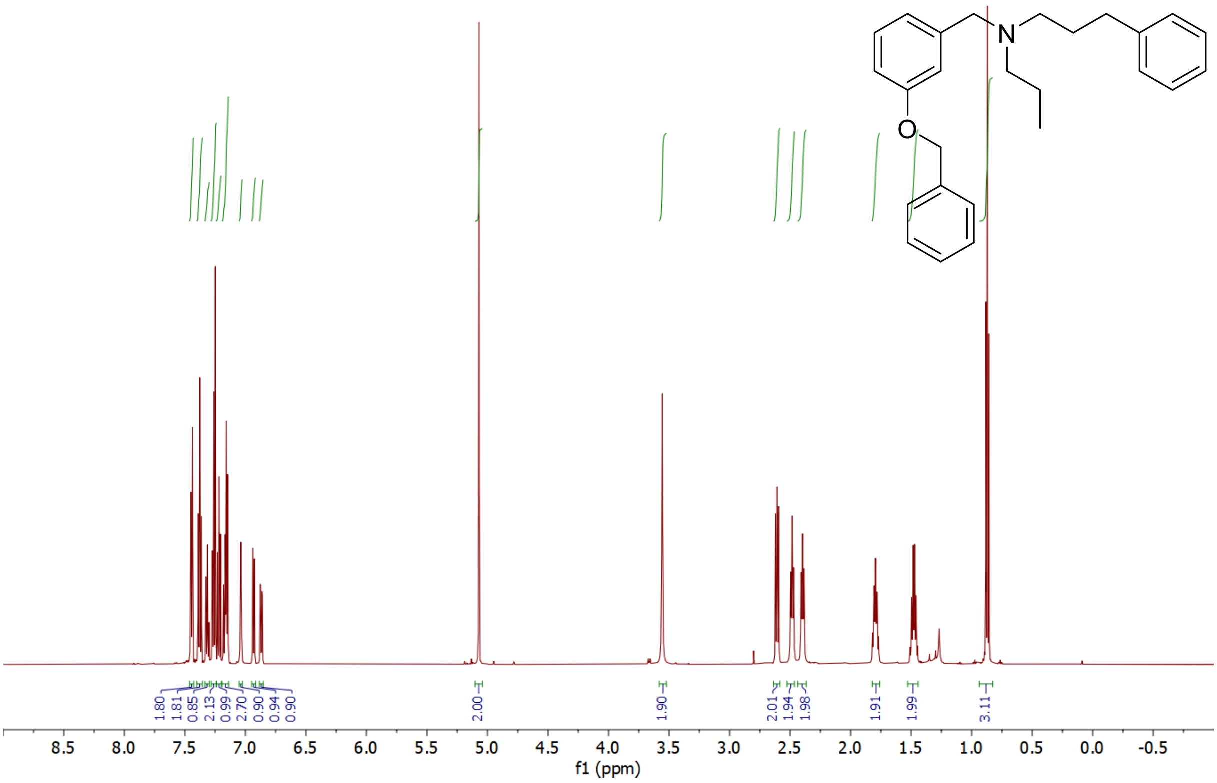

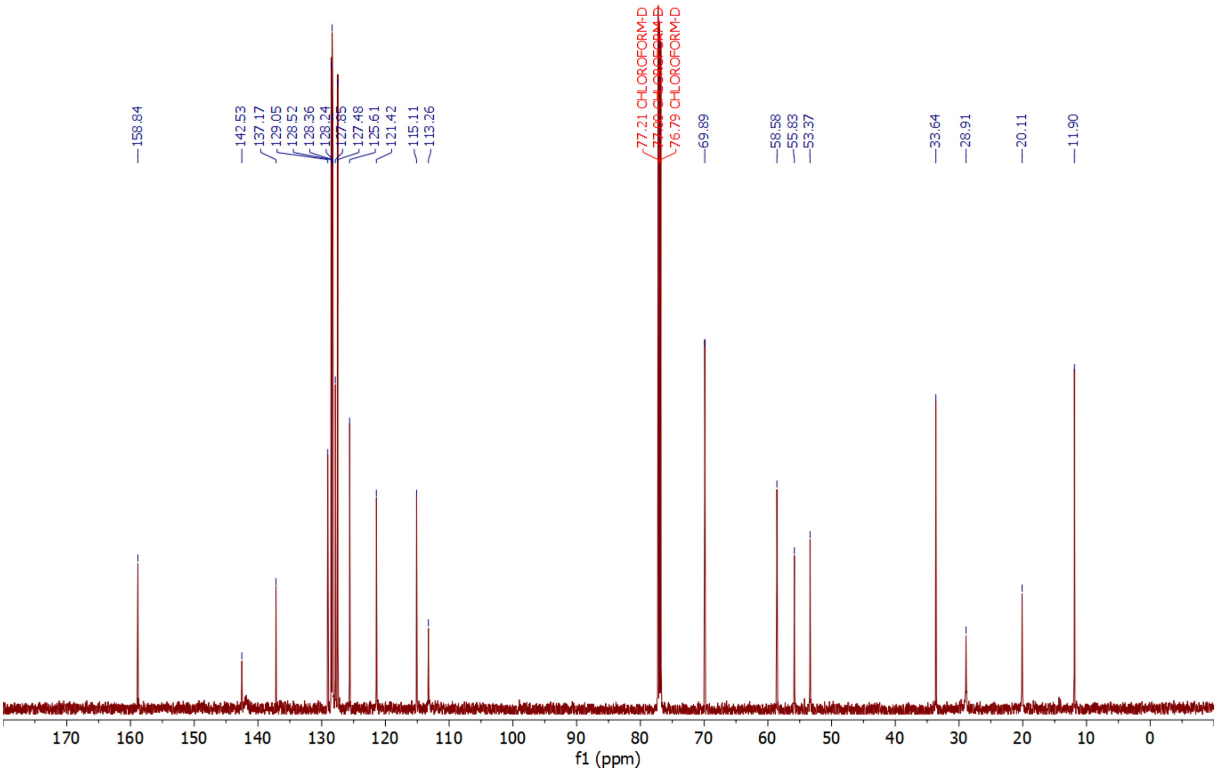

374,2478

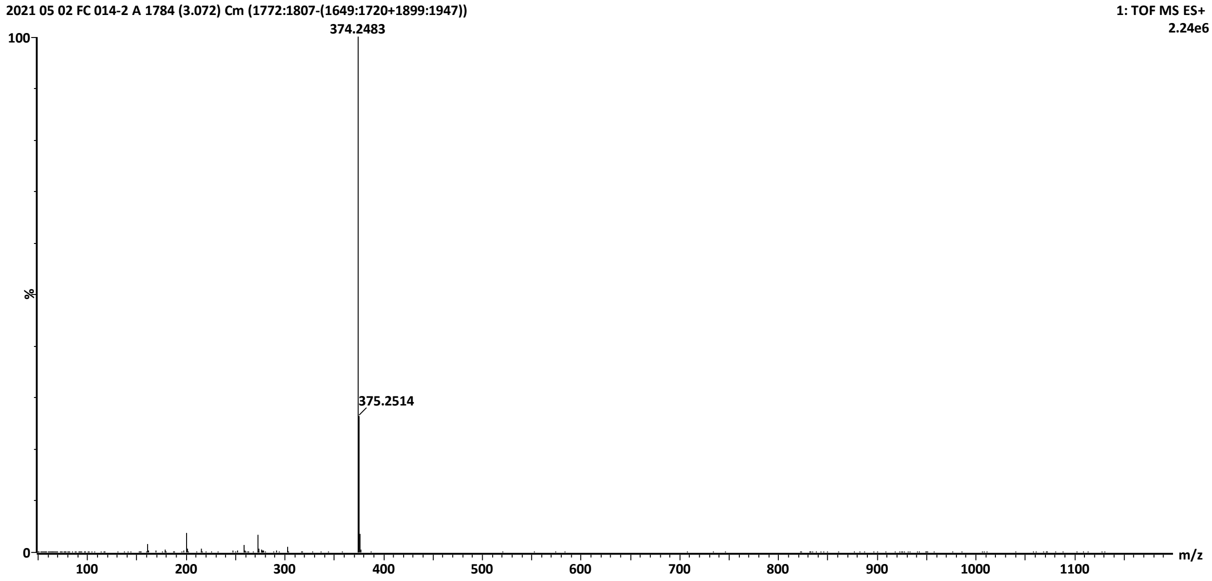
2021 05 02 FC 014-2 A 1784 (3.072) Cm (1772:1807-(1649:1720+1899:1947))
1: TOF MS ES+
2.24e6
374.2483
375.2514
100
%
0
m/z
100
200
300
400
500
600
700
800
900
1000
1100

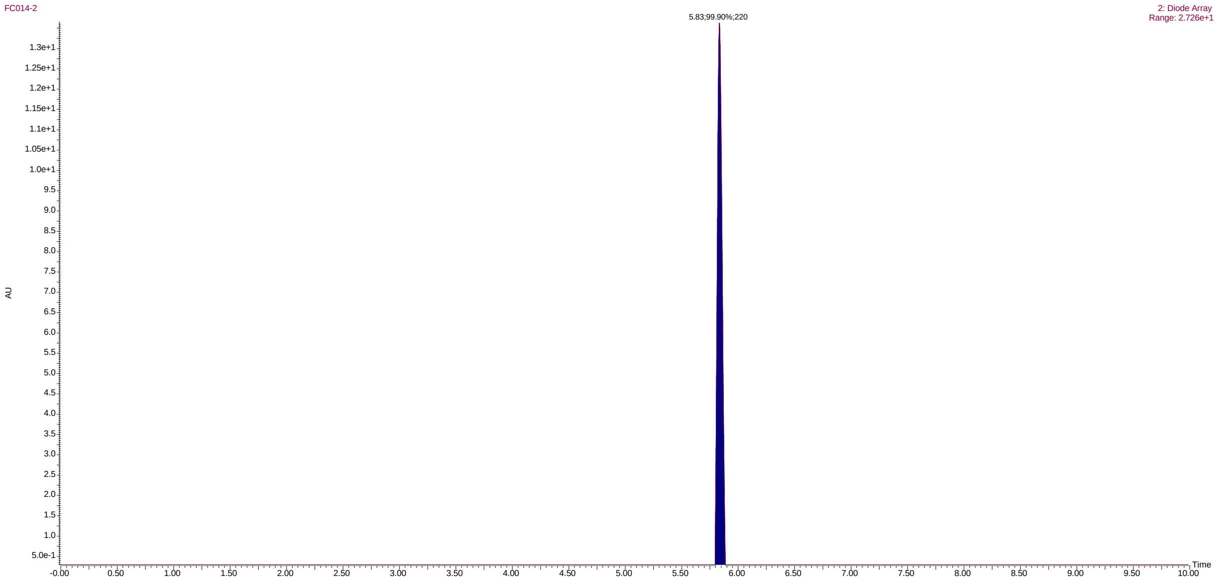
FC014-2
2: Diode Array
Range: 2.726e+1
5.83;99.90%;220
AU
Time

{[3-(Benzyloxy)phenyl]methyl}(3-phenylpropyl)(prop-2-en-1-yl)amine (26):

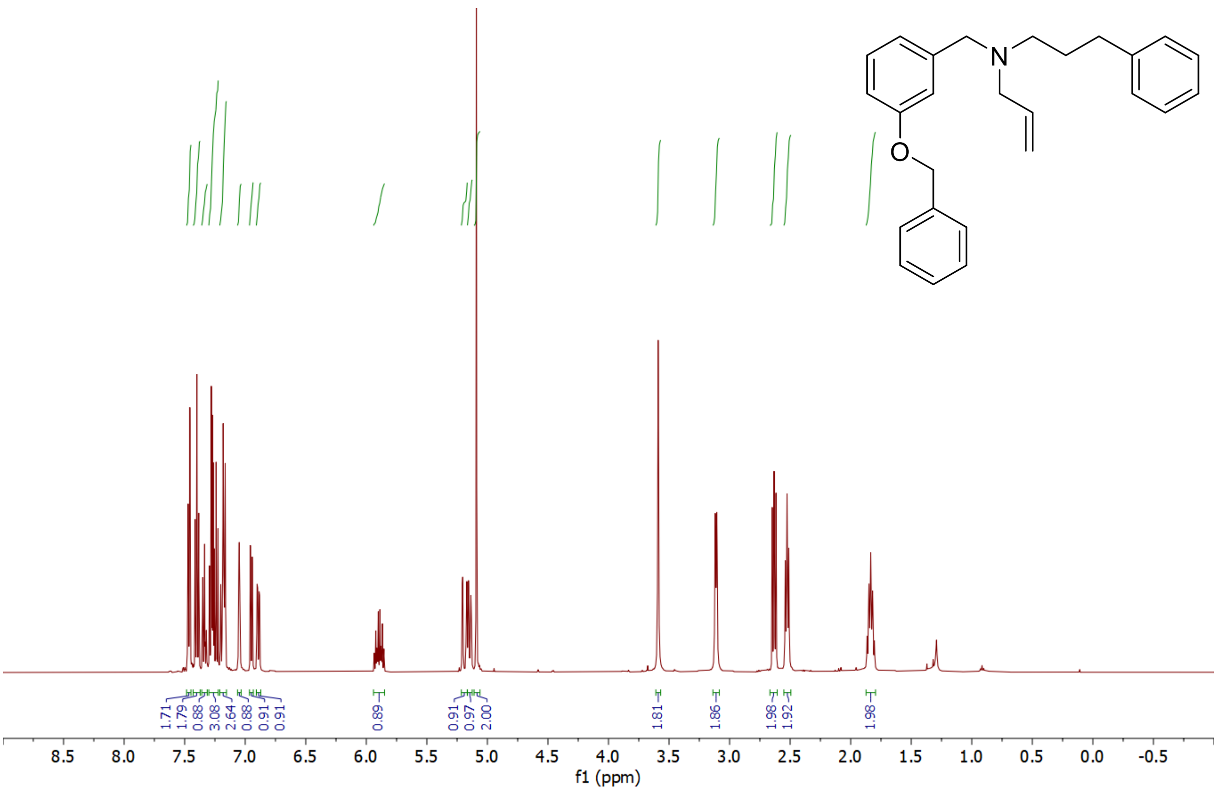

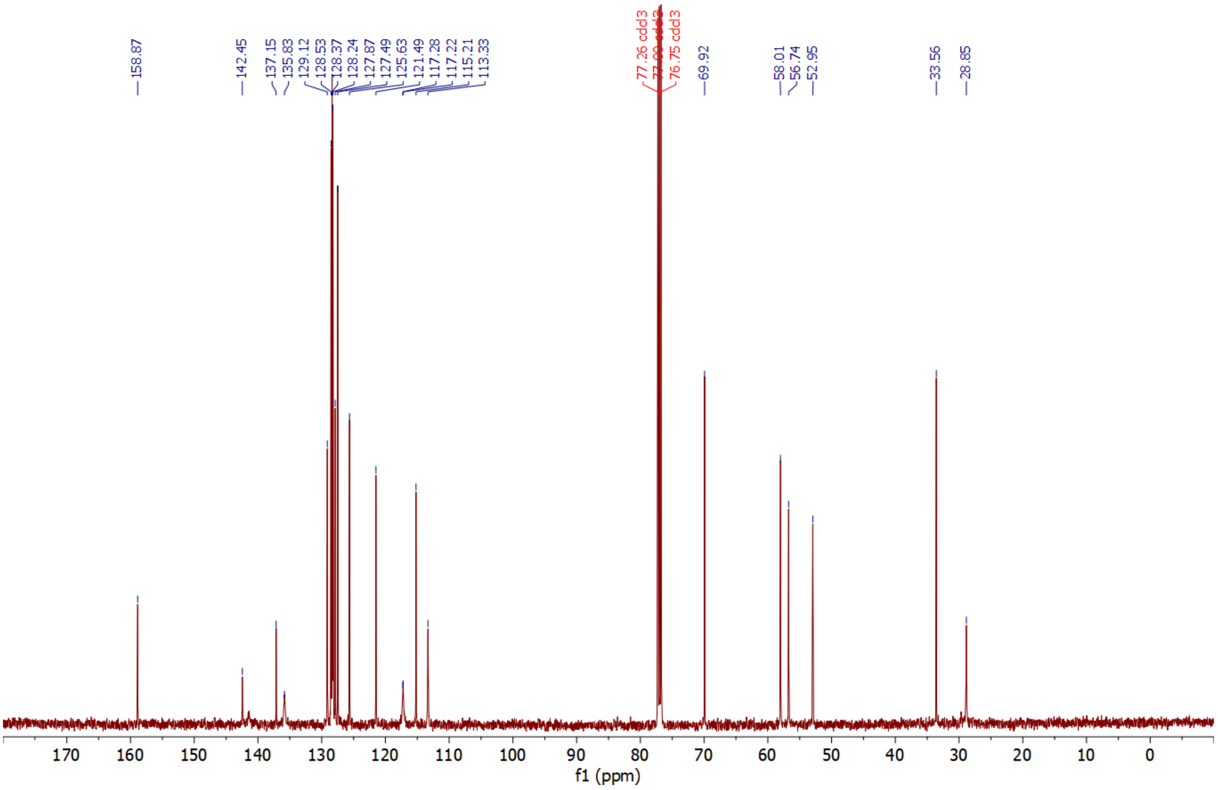

372,2322

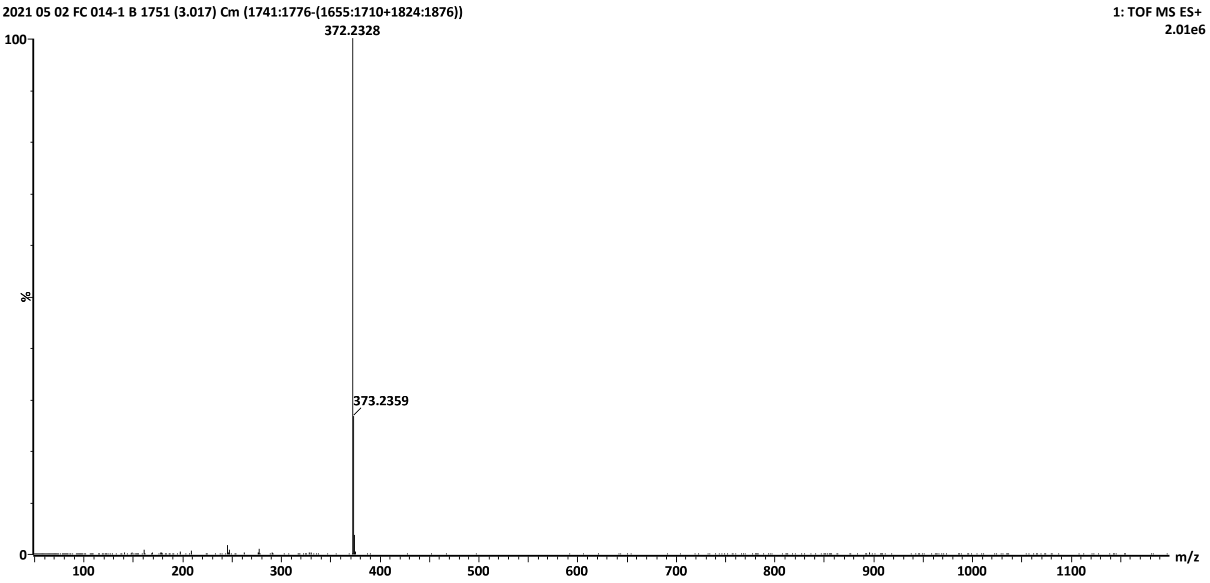
2021 05 02 FC 014-1 B 1751 (3.017) Cm (1741:1776-(1655:1710+1824:1876))
1: TOF MS ES+
2.01e6
372.2328
373.2359
%
m/z

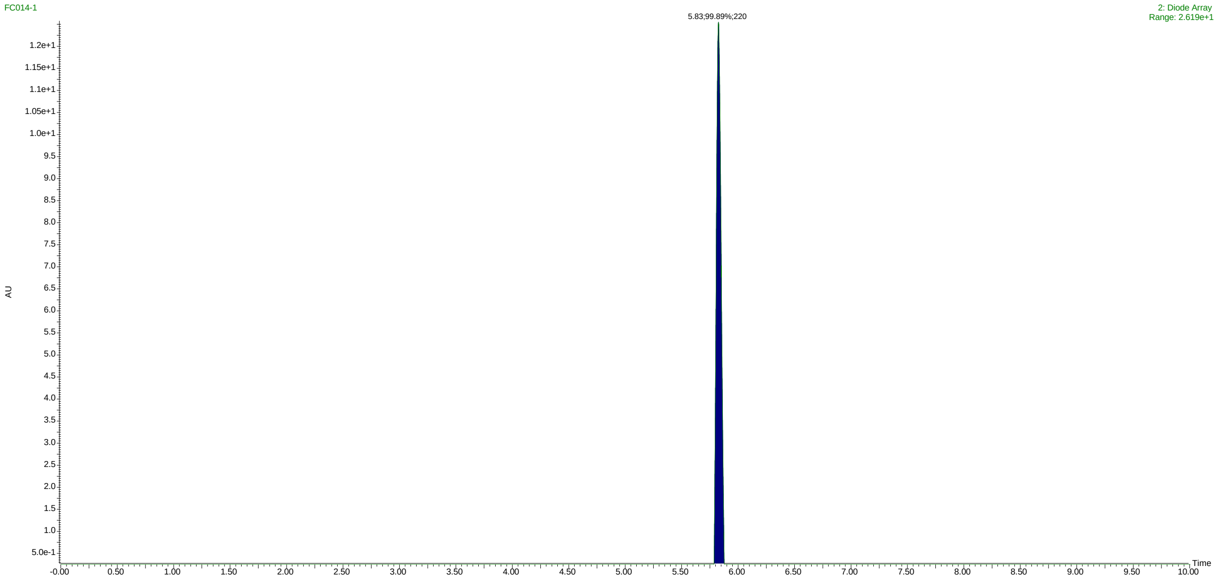
FC014-1
2: Diode Array
Range: 2.619e+1
5.83;99.89%;220
AU
Time

4-[2-({[4-(Benzyloxy)phenyl]methyl}amino)ethyl]phenol (28):

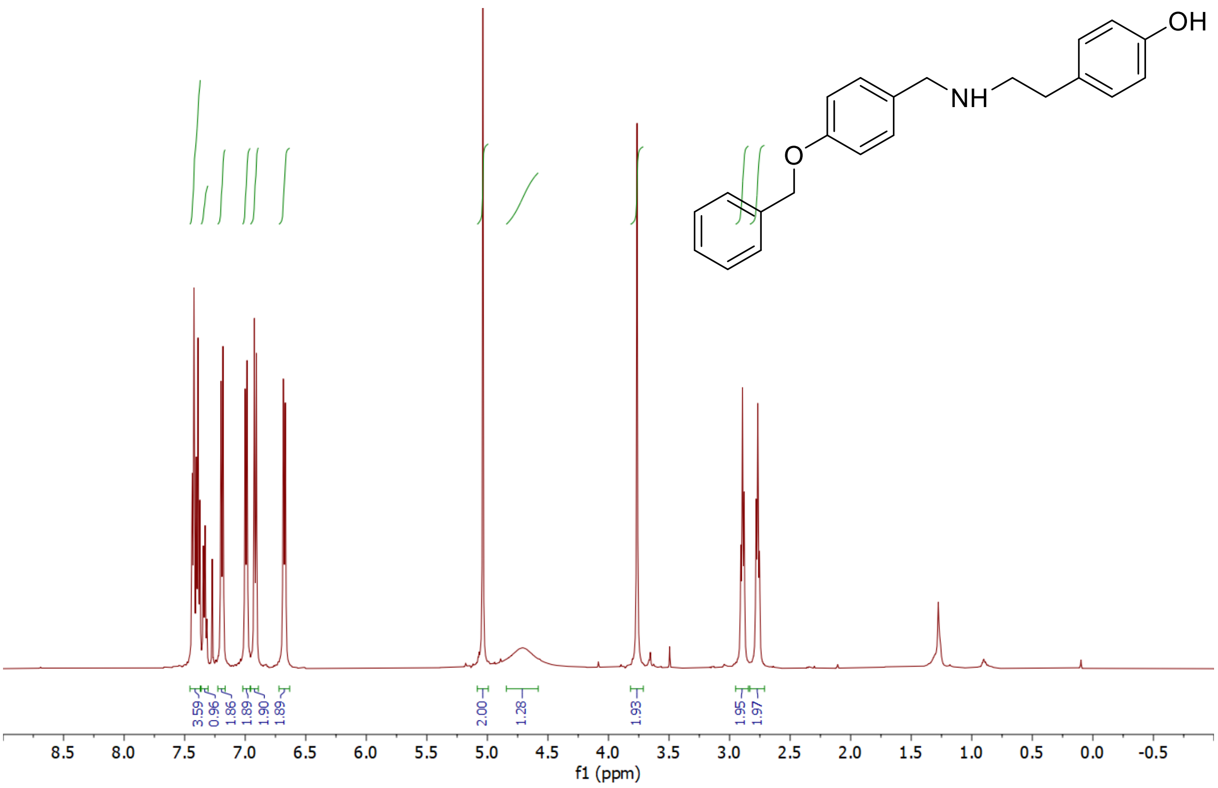

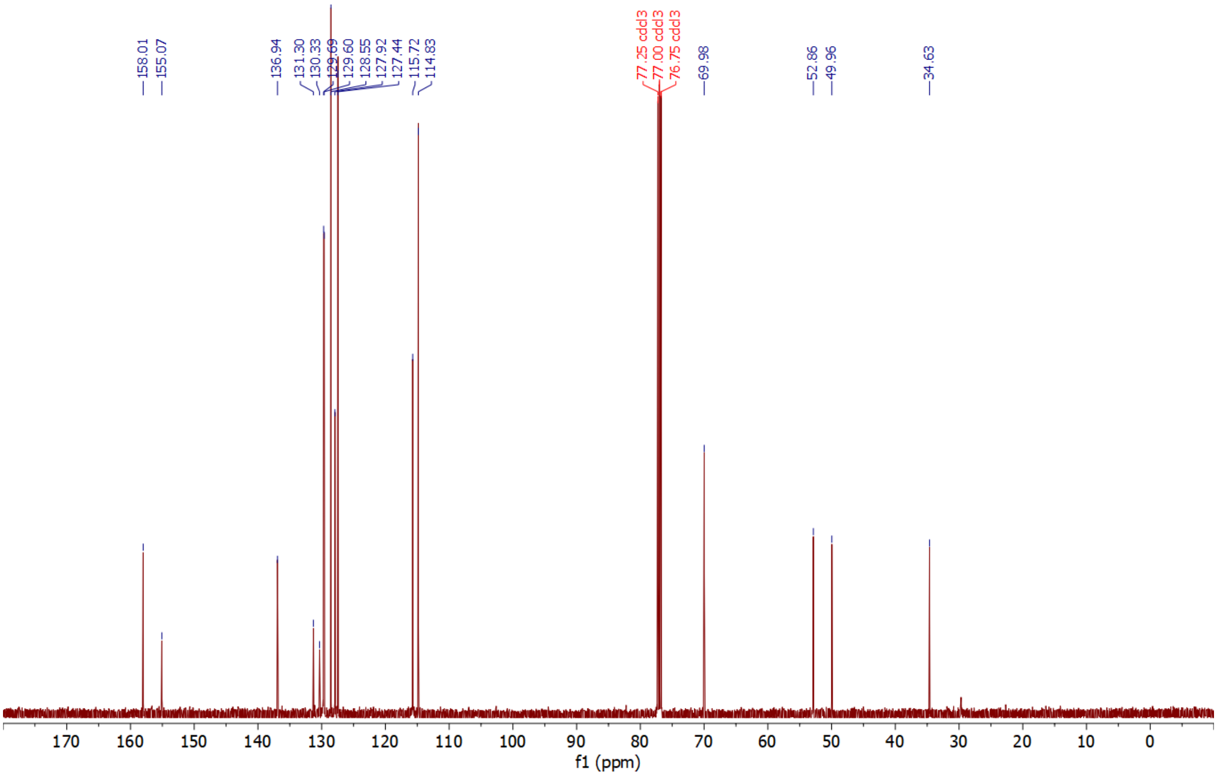

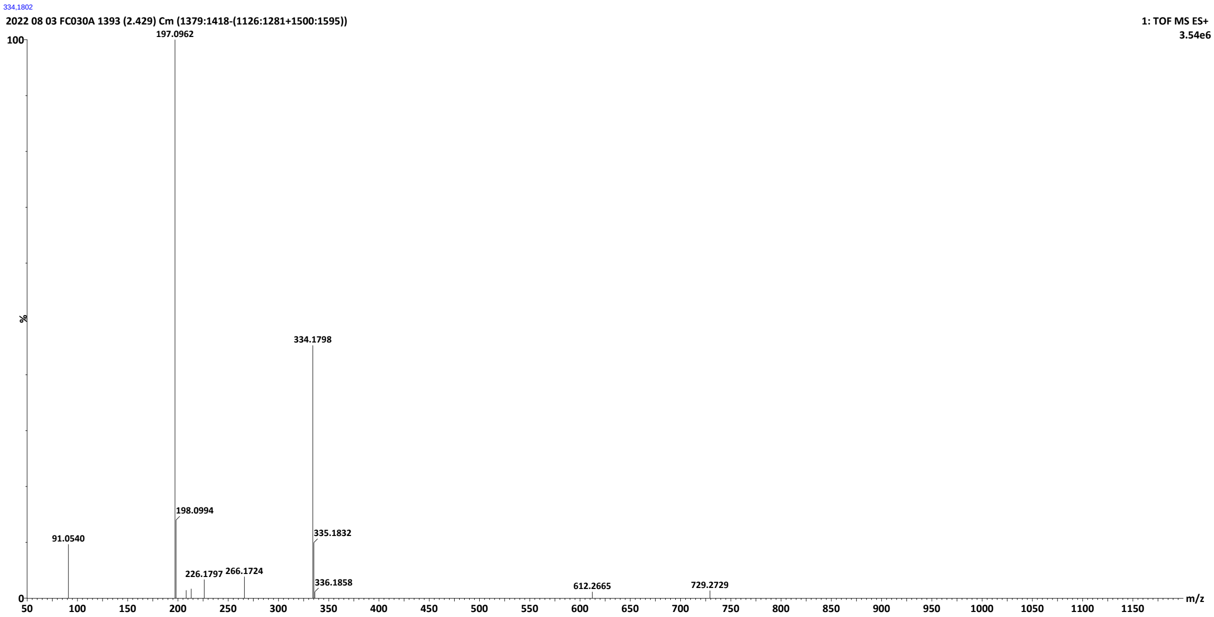
334.1802
2022 08 03 FC030A 1393 (2.429) Cm (1379:1418-(1126:1281+1500:1595))
1: TOF MS ES+
3.54e6
197.0962
334.1798
198.0994
91.0540
335.1832
226.1797
266.1724
336.1858
612.2665
729.2729
m/z
%

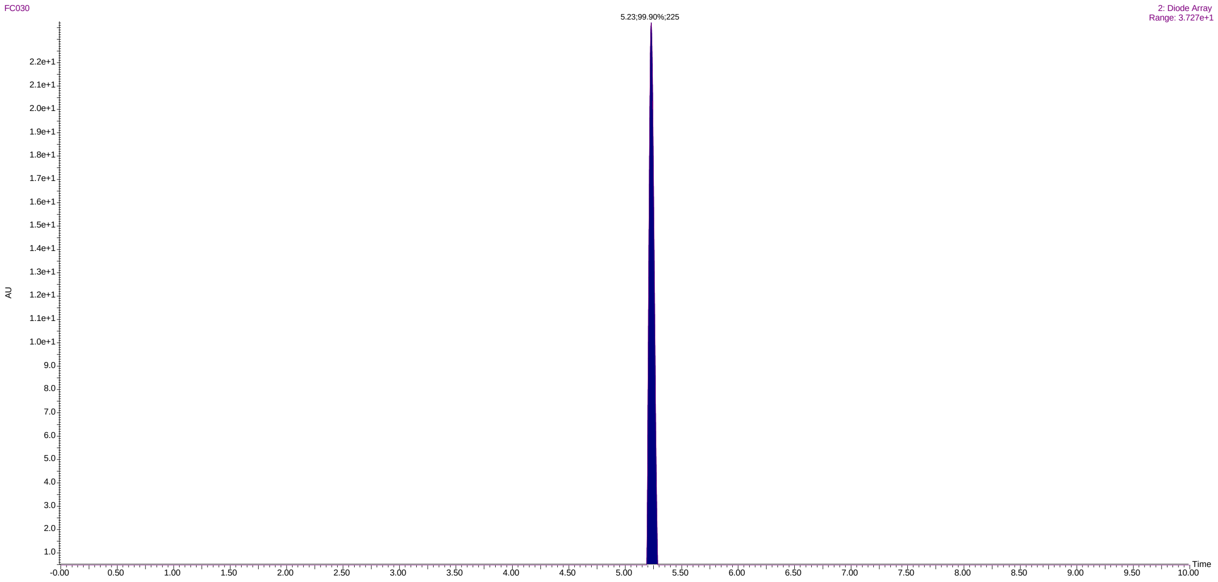
FC030
2: Diode Array
Range: 3.727e+1
5.23;99.90%;225
AU
Time

{[4-(Benzyloxy)phenyl]methyl}[2-(2-methoxyphenyl)ethyl]amine (29):

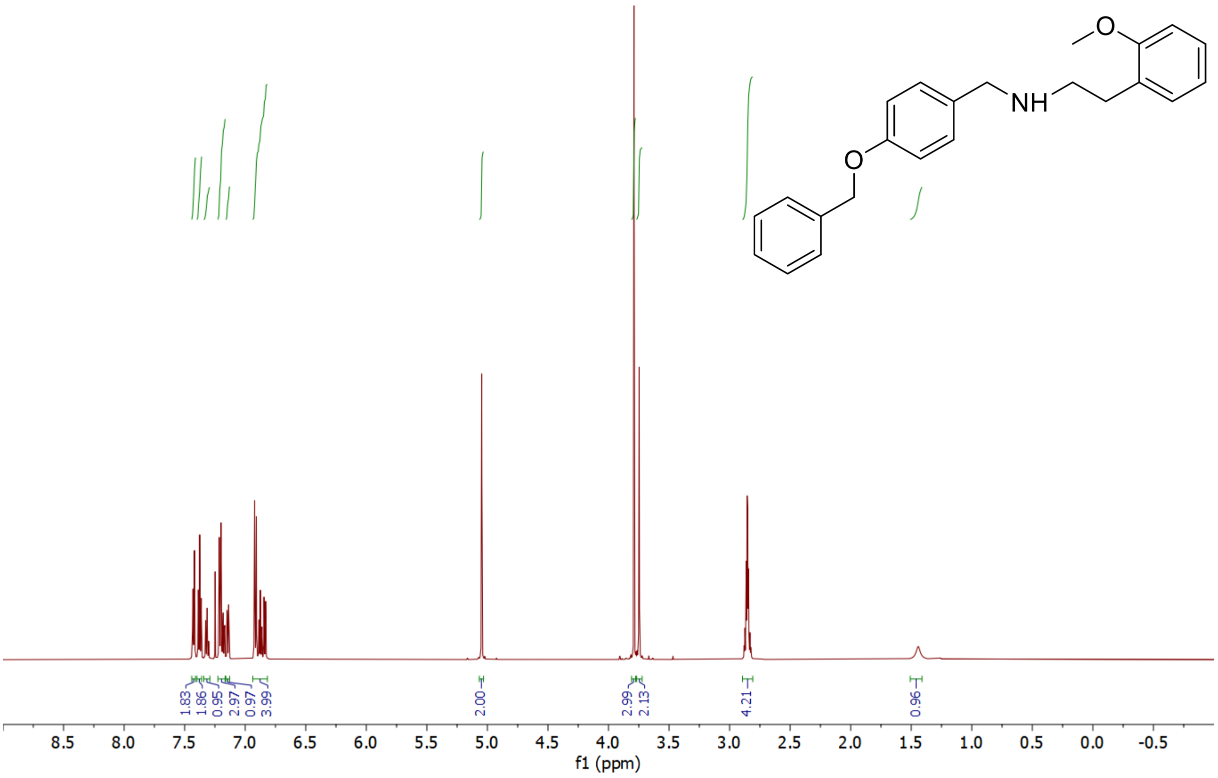

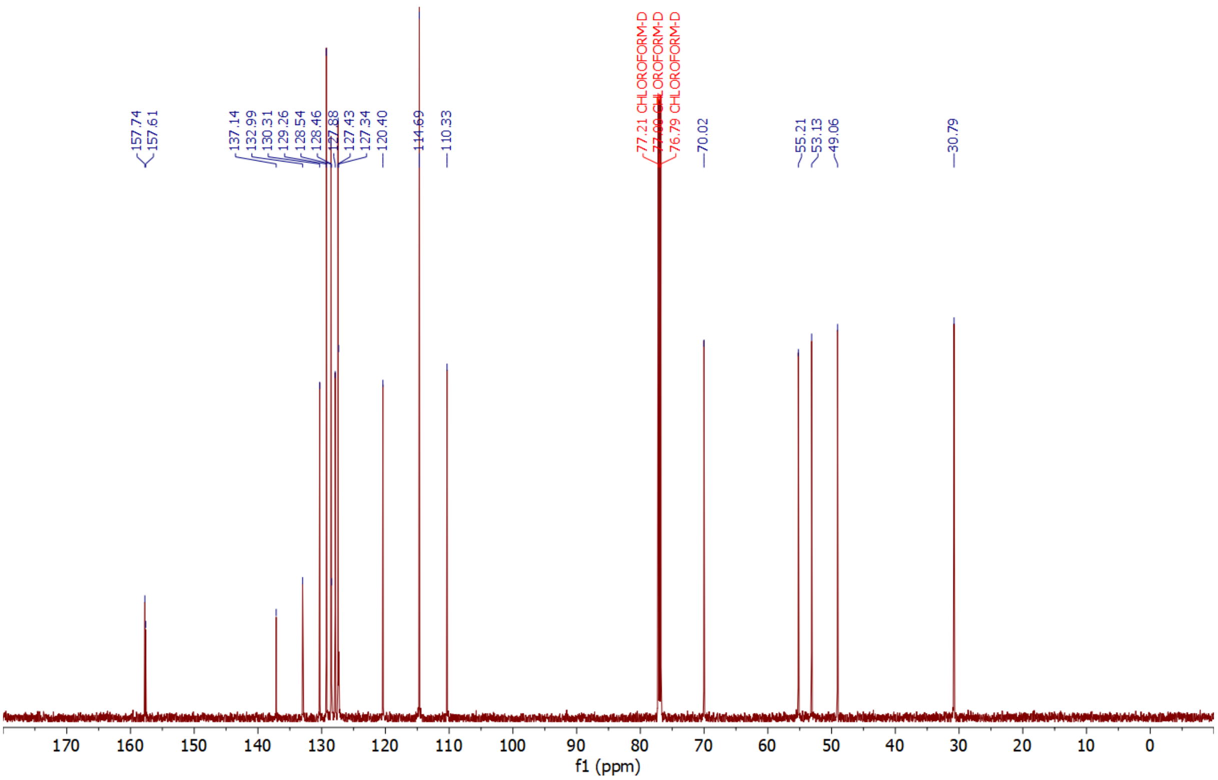

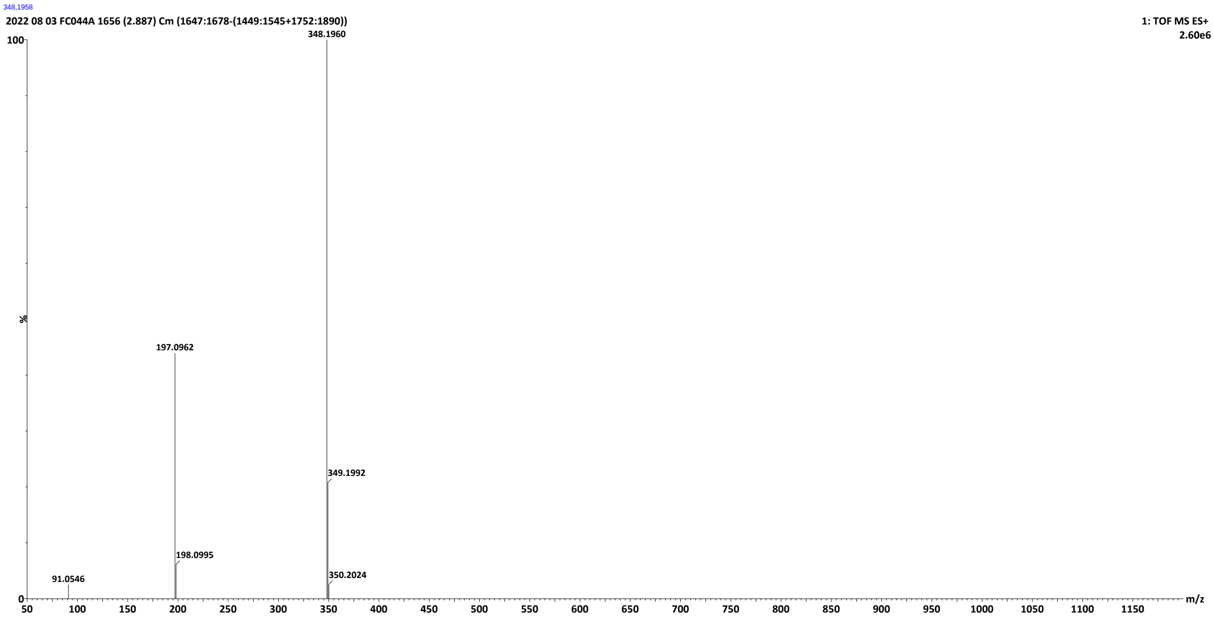
348.1958
2022 08 03 FC044A 1656 (2.887) Cm (1647:1678-(1449:1545+1752:1890))
1: TOF MS ES+
2.60e6
348.1960
197.0962
349.1992
198.0995
91.0546
350.2024
%
m/z

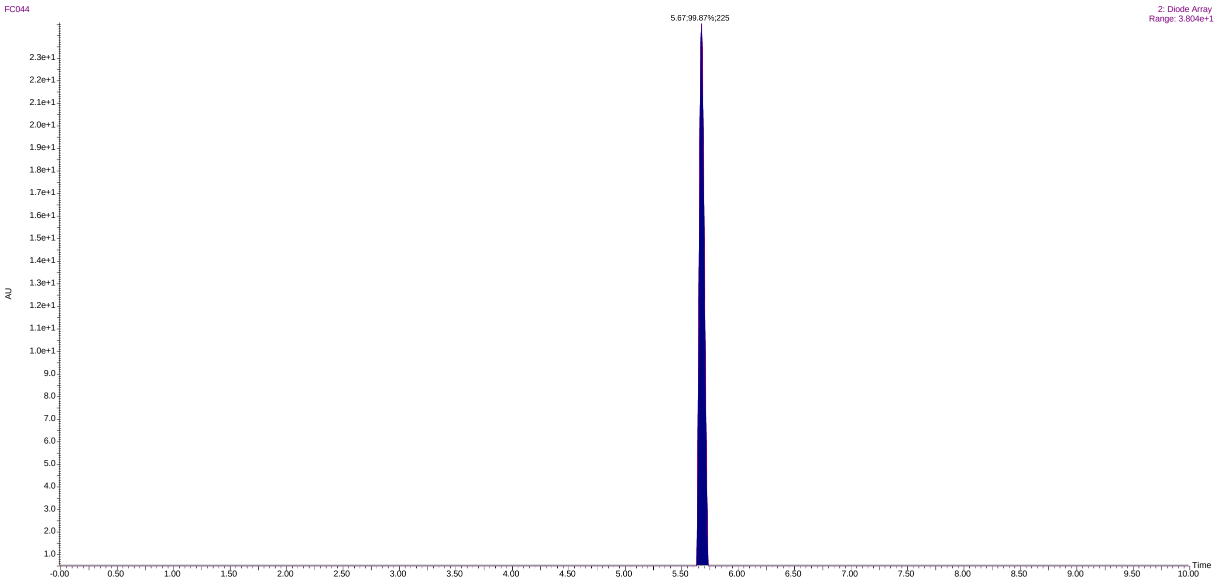
FC044
2: Diode Array
Range: 3.804e+1
5.67;99.87%;225
AU
Time

{[4-(Benzyloxy)phenyl]methyl}[2-(3-methoxyphenyl)ethyl]amine (30):

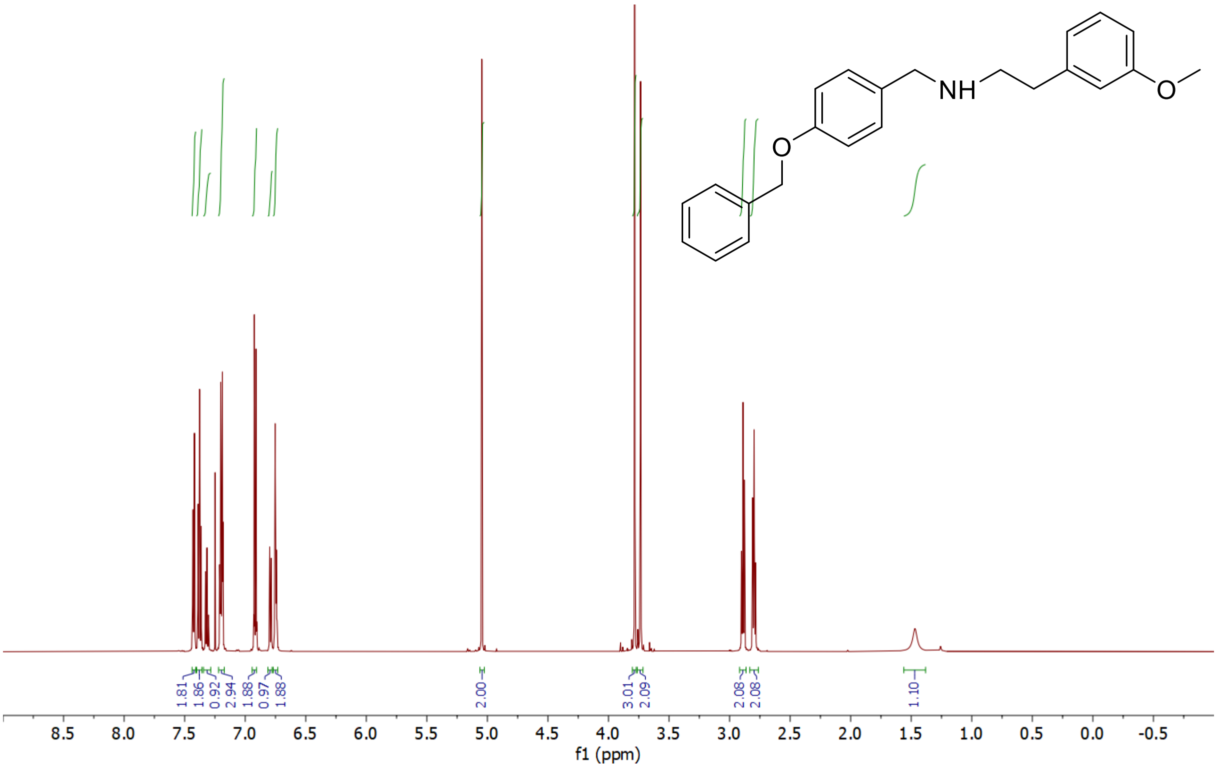

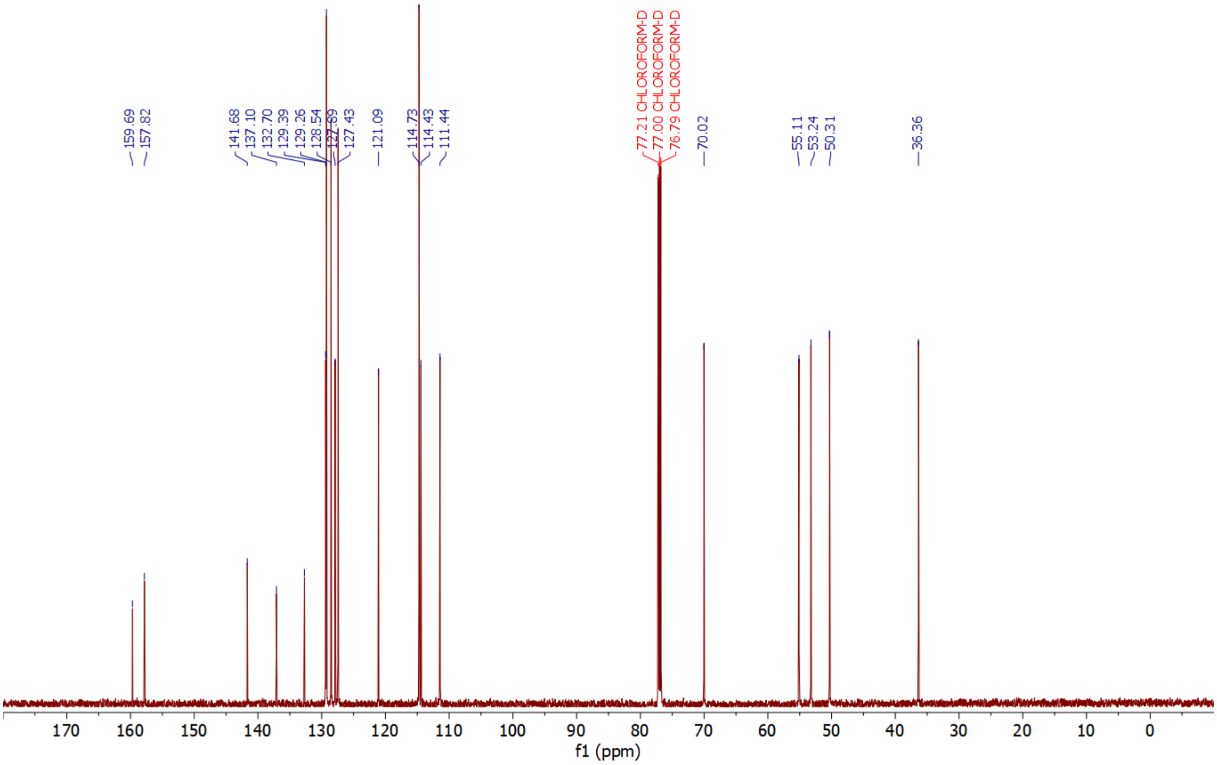

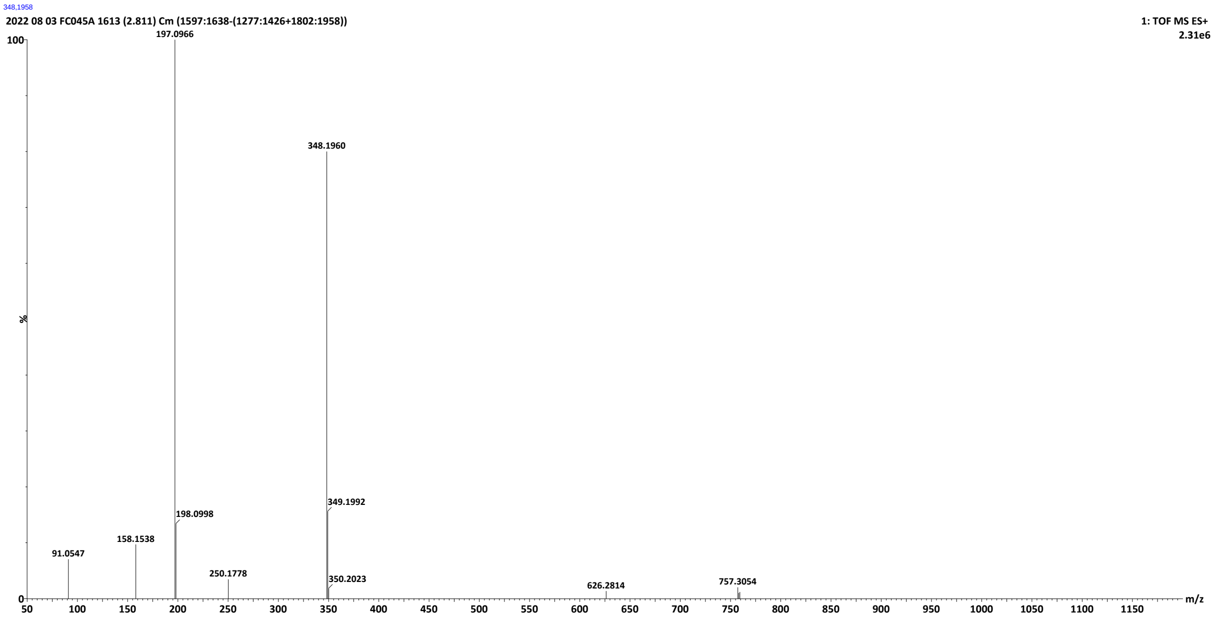
348.1958
2022 08 03 FC045A 1613 (2.811) Cm (1597:1638-(1277:1426+1802:1958))
1: TOF MS ES+
2.31e6
197.0966
348.1960
349.1992
198.0998
158.1538
91.0547
250.1778
350.2023
626.2814
757.3054
%
m/z

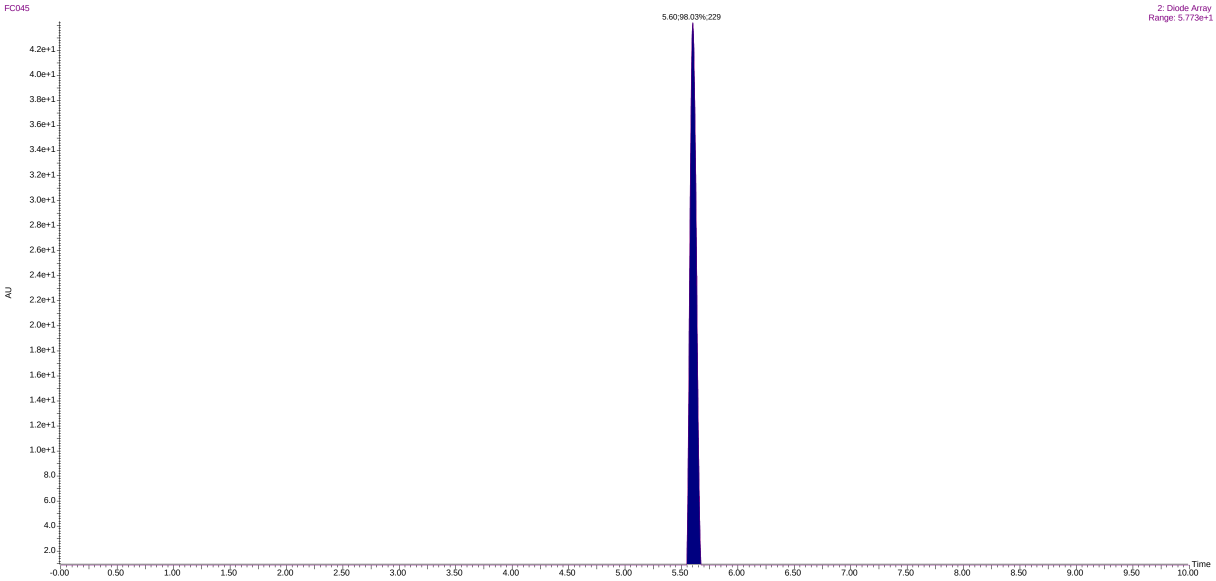
FC045
2: Diode Array
Range: 5.773e+1
5.60;98.03%;229
AU
Time

{[4-(Benzyloxy)phenyl]methyl}[2-(4-methoxyphenyl)ethyl]amine (31):

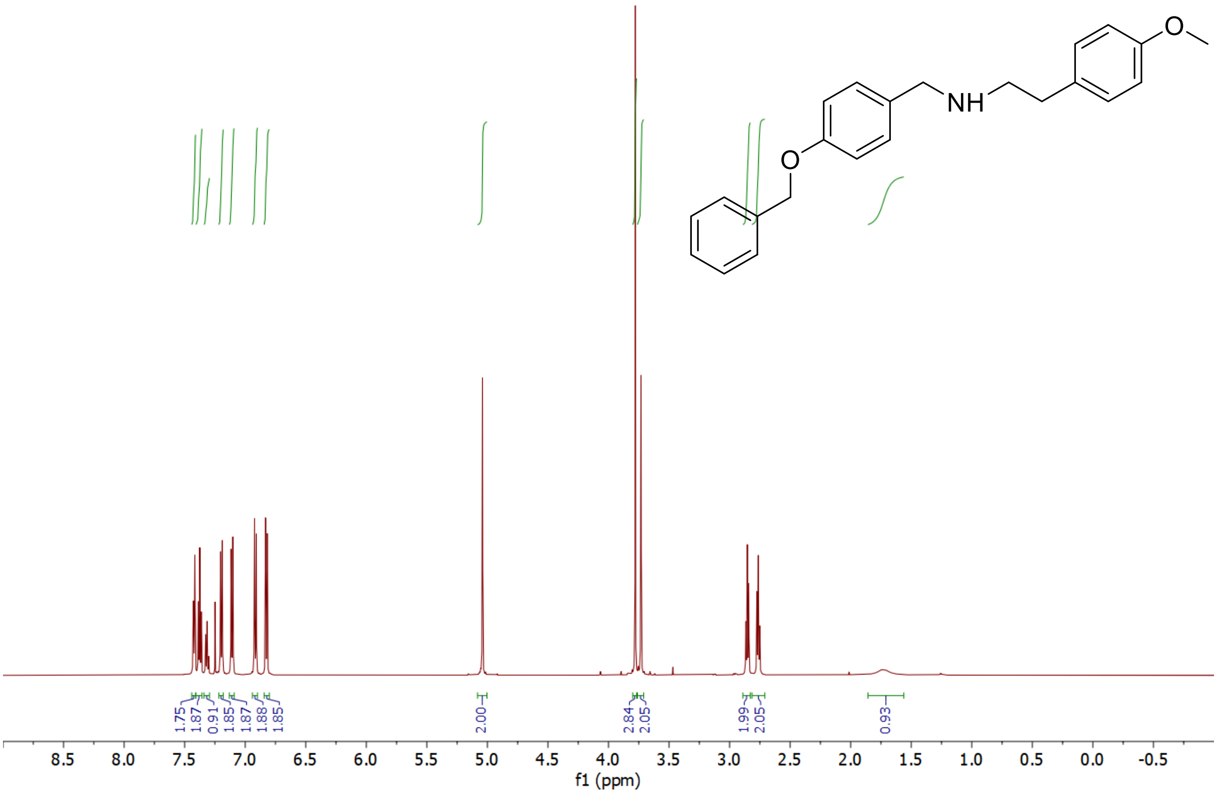

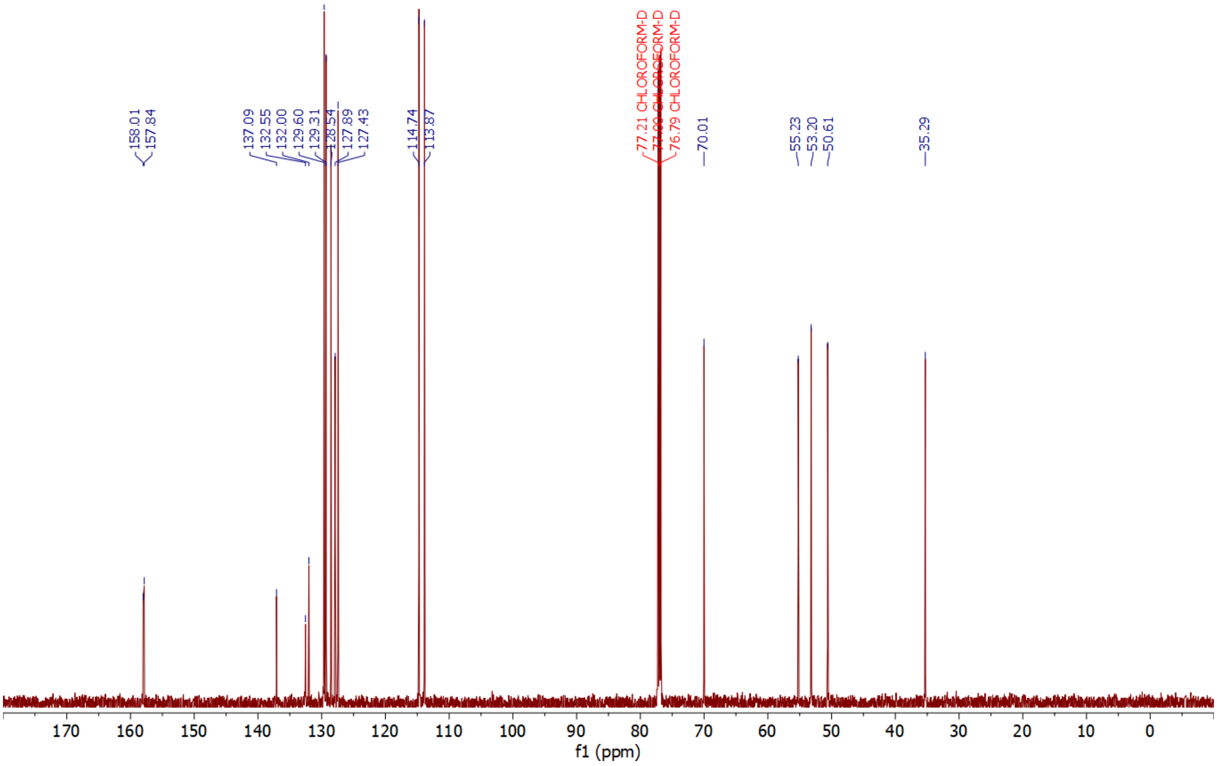

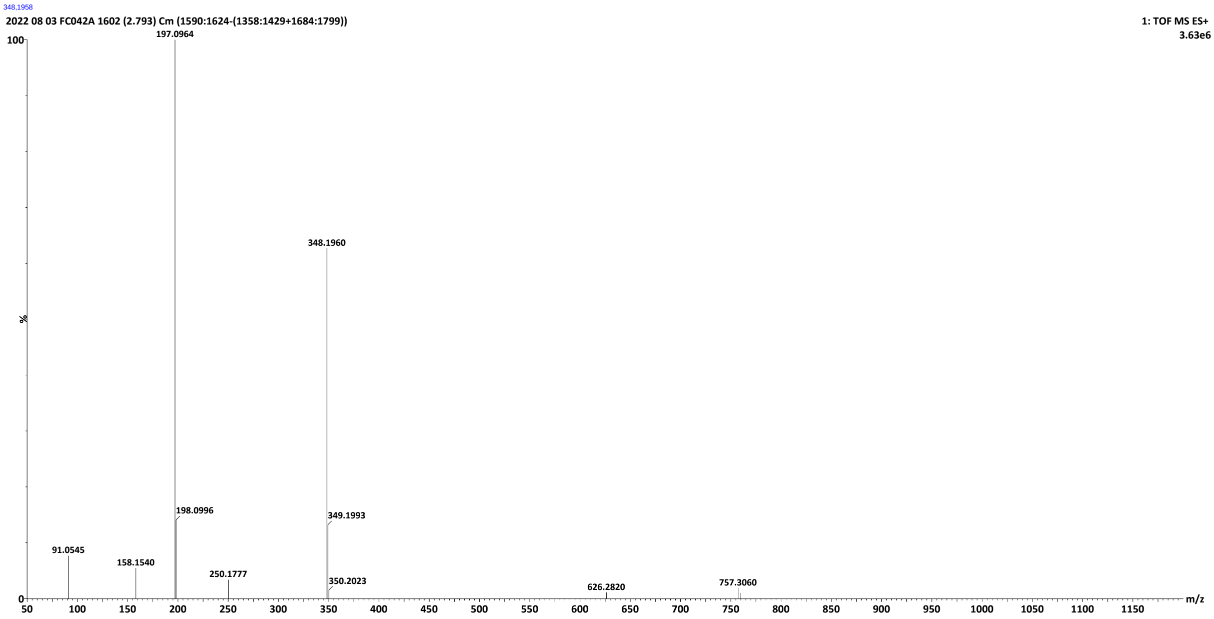
348.1958
2022 08 03 FC042A 1602 (2.793) Cm (1590:1624-(1358:1429+1684:1799))
1: TOF MS ES+
3.63e6
100
%
0
197.0964
348.1960
198.0996
349.1993
91.0545
158.1540
250.1777
350.2023
626.2820
757.3060
50 100 150 200 250 300 350 400 450 500 550 600 650 700 750 800 850 900 950 1000 1050 1100 1150
m/z

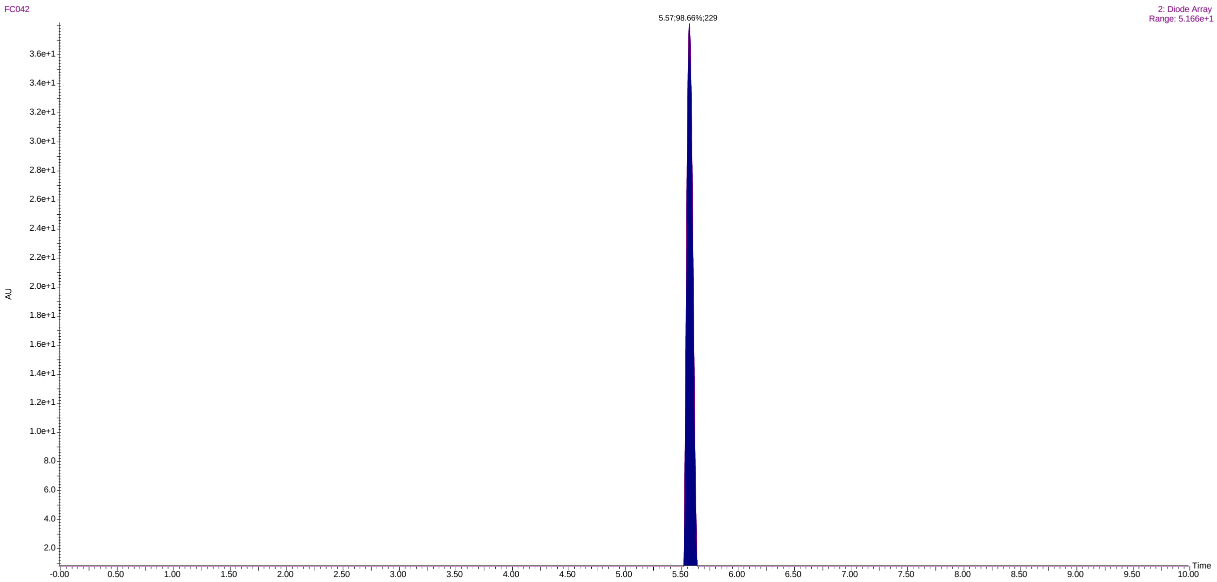
FC042
2: Diode Array
Range: 5.166e+1
5.57;98.66%;229
AU
3.6e+1
3.4e+1
3.2e+1
3.0e+1
2.8e+1
2.6e+1
2.4e+1
2.2e+1
2.0e+1
1.8e+1
1.6e+1
1.4e+1
1.2e+1
1.0e+1
8.0
6.0
4.0
2.0
-0.00 0.50 1.00 1.50 2.00 2.50 3.00 3.50 4.00 4.50 5.00 5.50 6.00 6.50 7.00 7.50 8.00 8.50 9.00 9.50 10.00
Time

{[4-(Benzyloxy)phenyl]methyl}[2-(4-chlorophenyl)ethyl]amine (32):

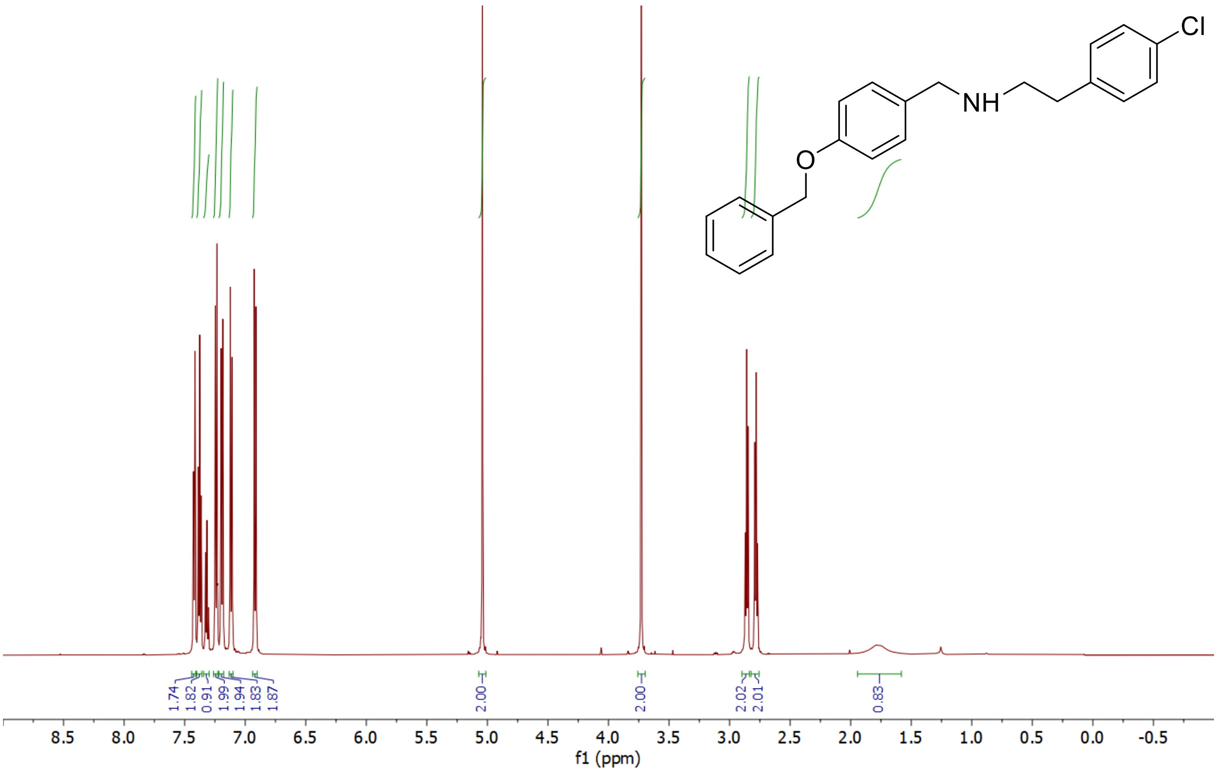

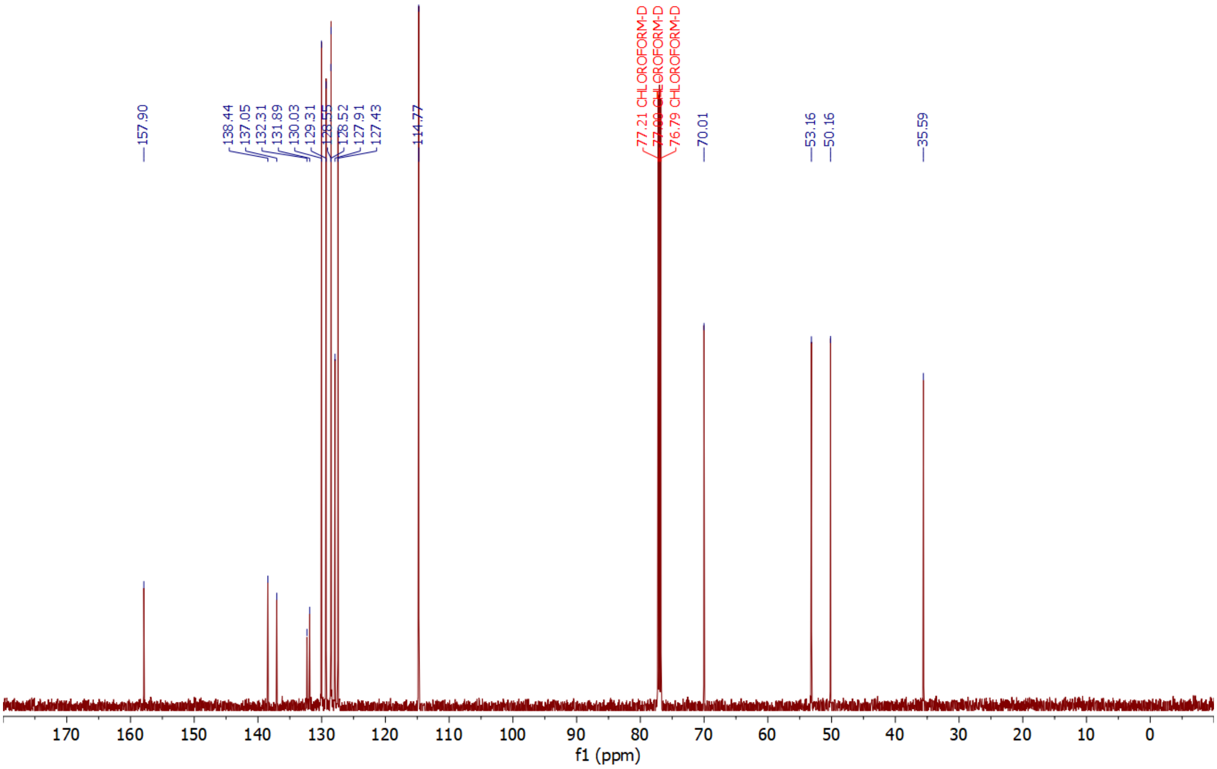

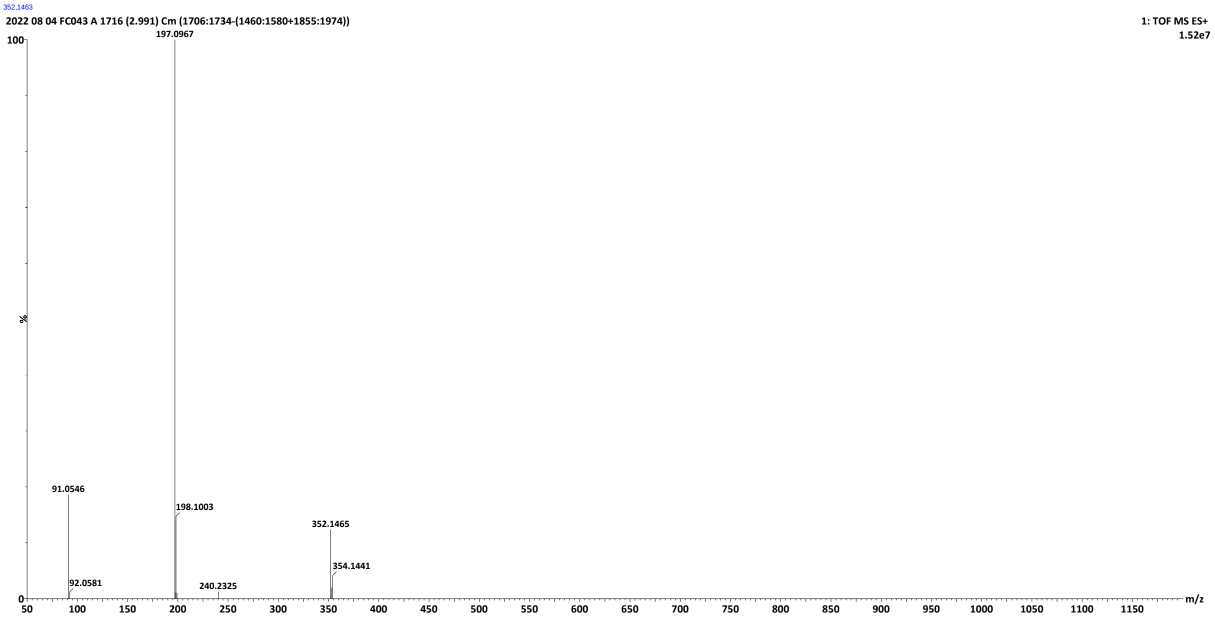
352.1463
2022 08 04 FC043 A 1716 (2.991) Cm (1706:1734-(1460:1580+1855:1974))
1: TOF MS ES+
1.52e7
197.0967
91.0546
198.1003
352.1465
354.1441
92.0581
240.2325
%
m/z

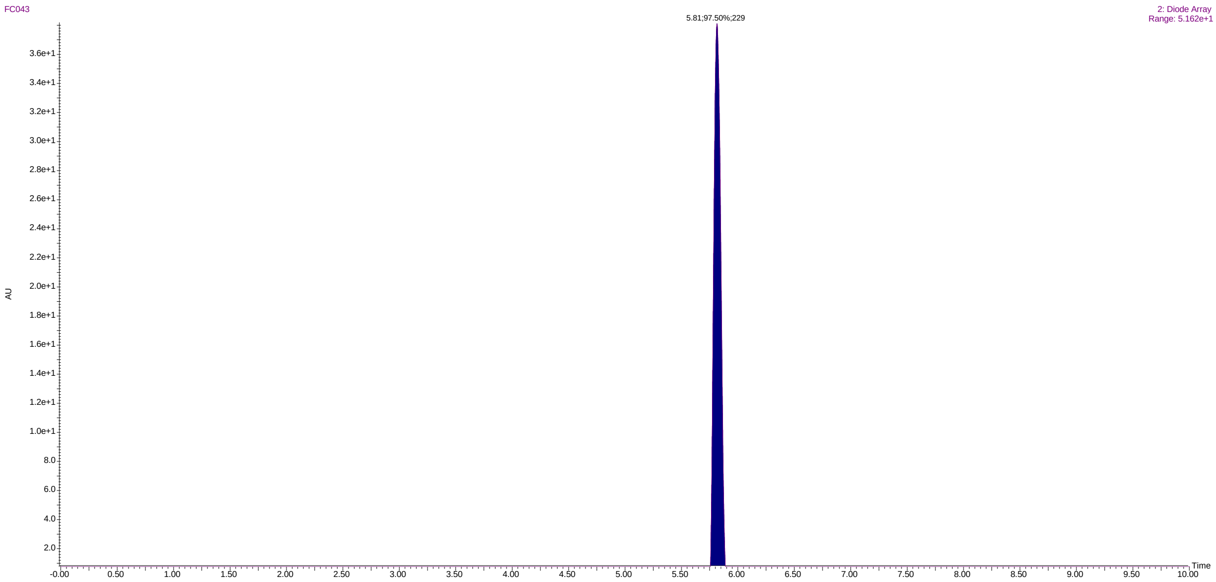
FC043
2: Diode Array
Range: 5.162e+1
5.81;97.50%;229
AU
Time

4-[2-({[4-(Benzyloxy)phenyl]methyl}(propyl)amino)ethyl]phenol (33):

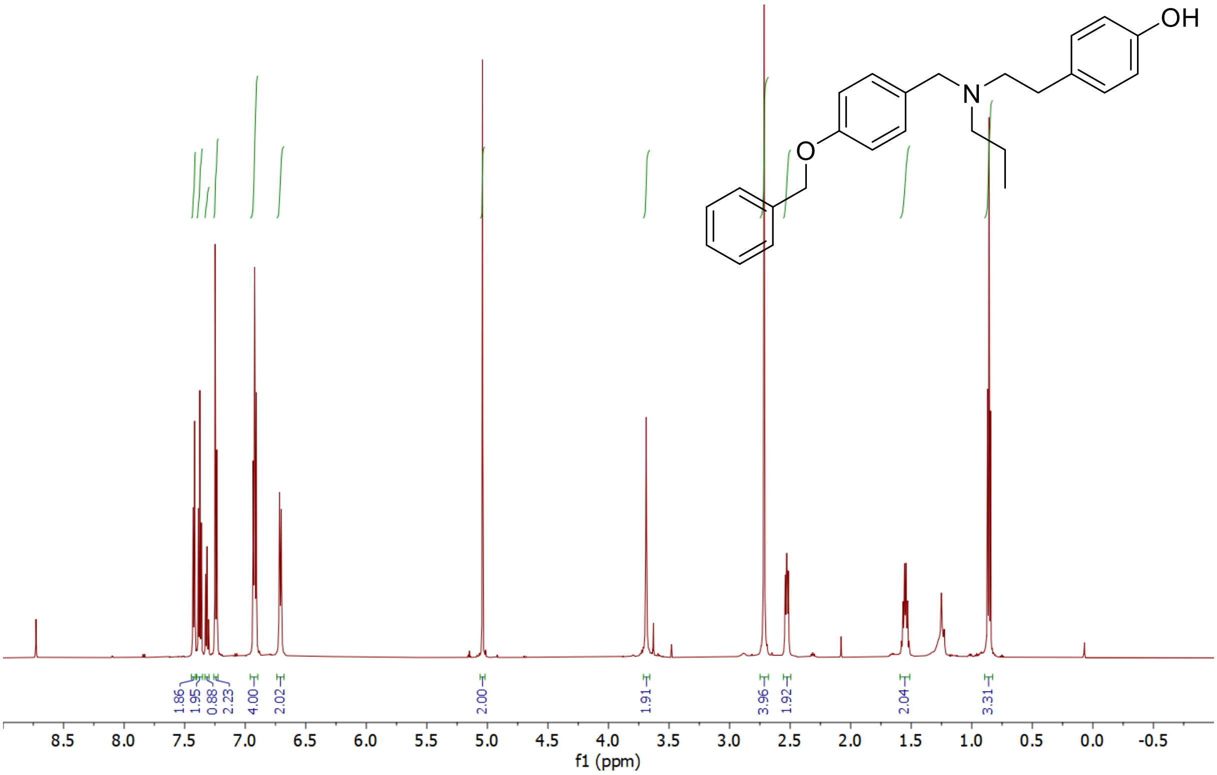

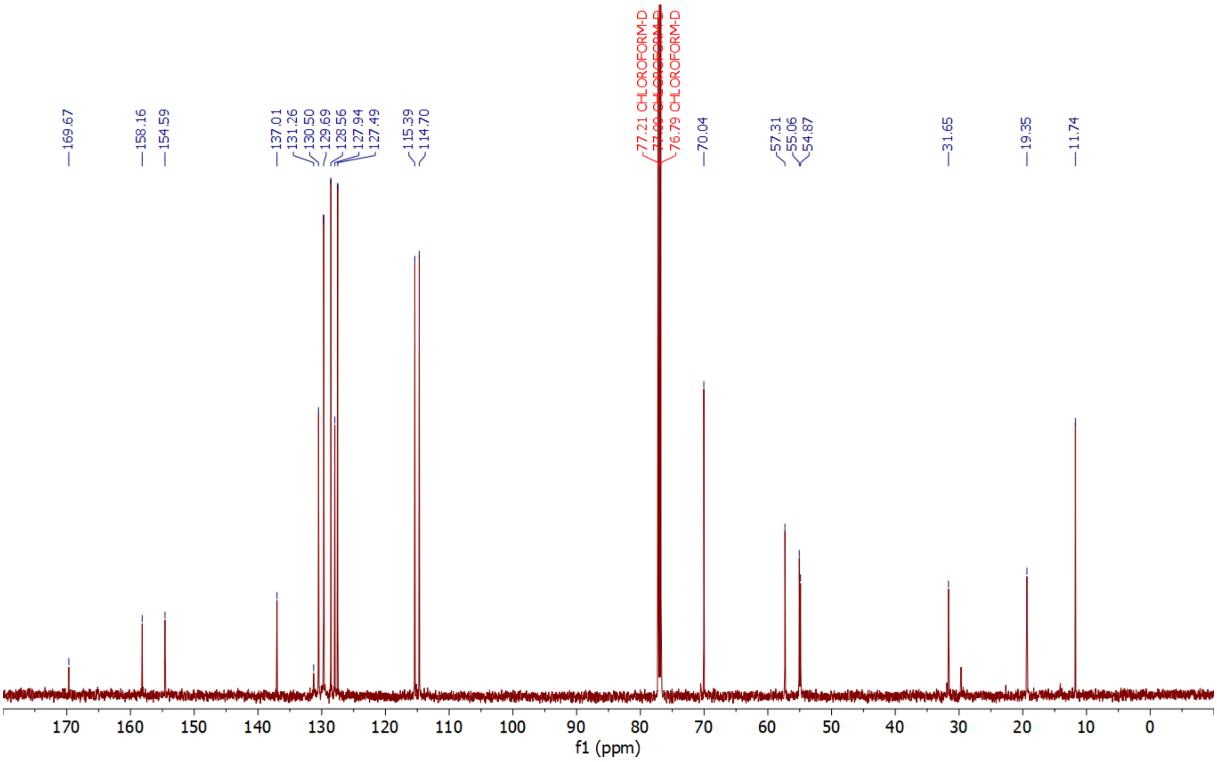

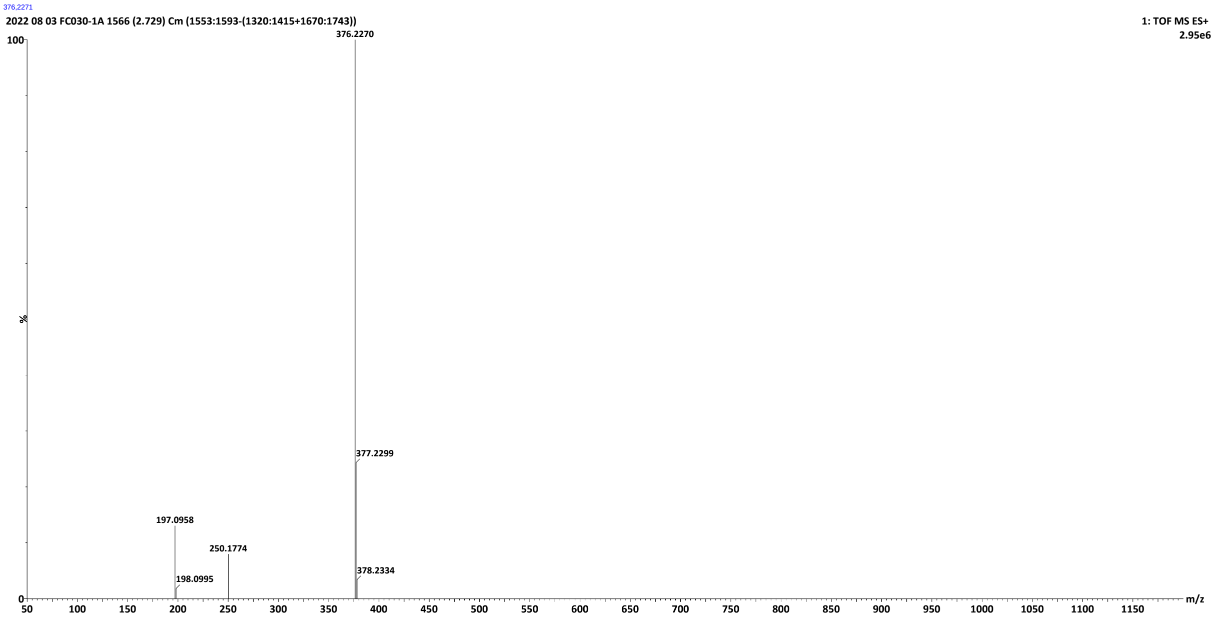
376.2271
2022 08 03 FC030-1A 1566 (2.729) Cm (1553:1593-(1320:1415+1670:1743))
1: TOF MS ES+
2.95e6
376.2270
377.2299
197.0958
250.1774
198.0995
378.2334
%
m/z

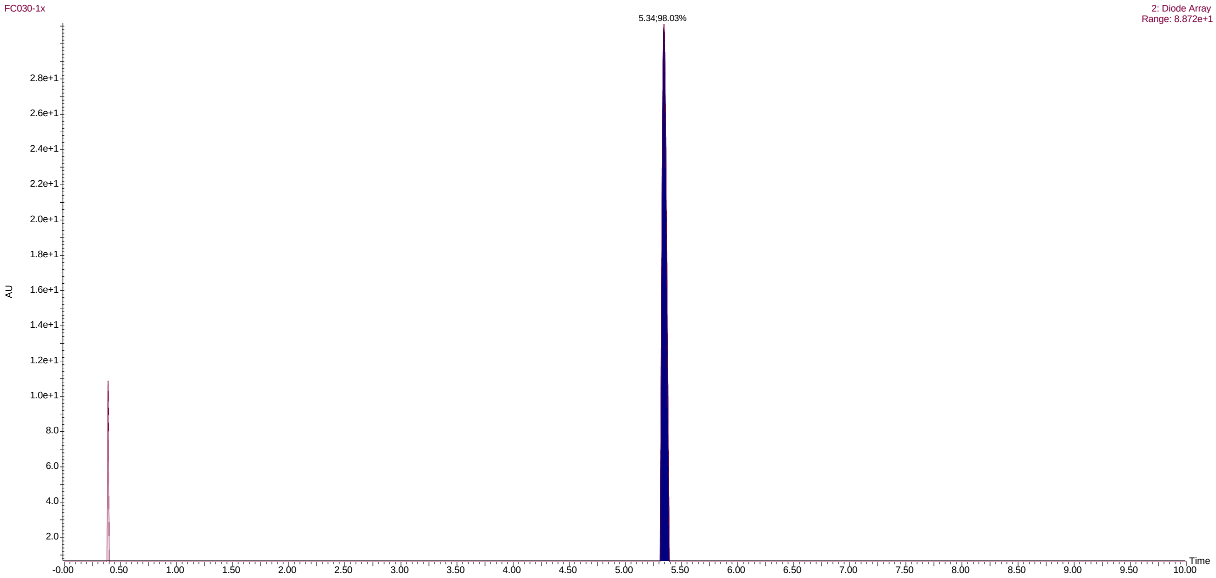
FC030-1x
2: Diode Array
Range: 8.872e+1
5.34;98.03%
AU
Time

{[4-(Benzyloxy)phenyl]methyl}[2-(4-propoxyphenyl)ethyl]propylamine (34):

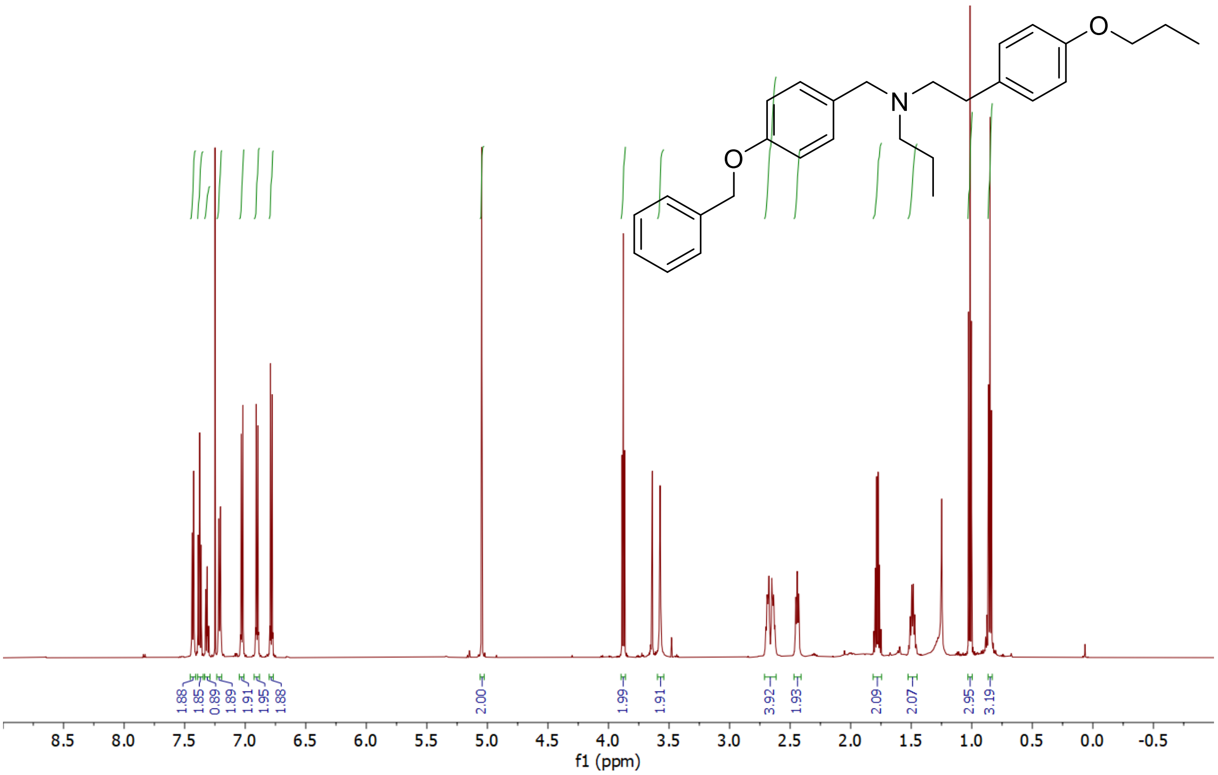

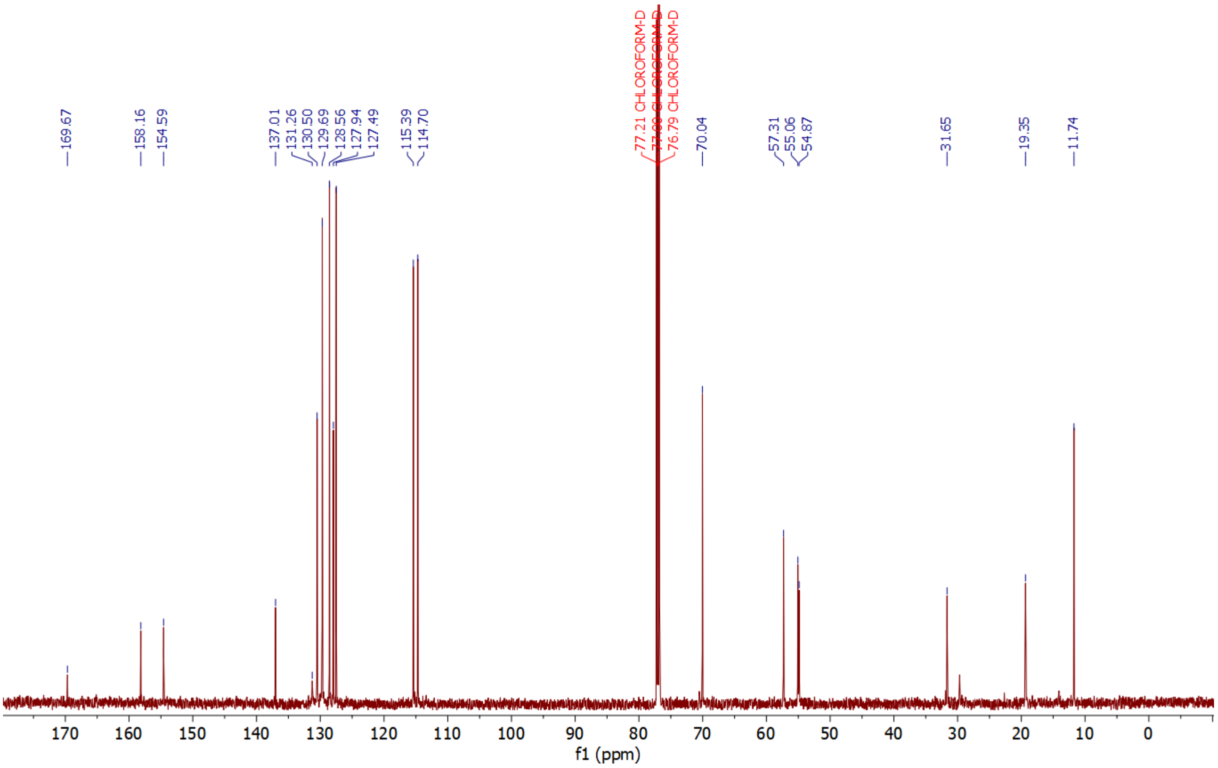

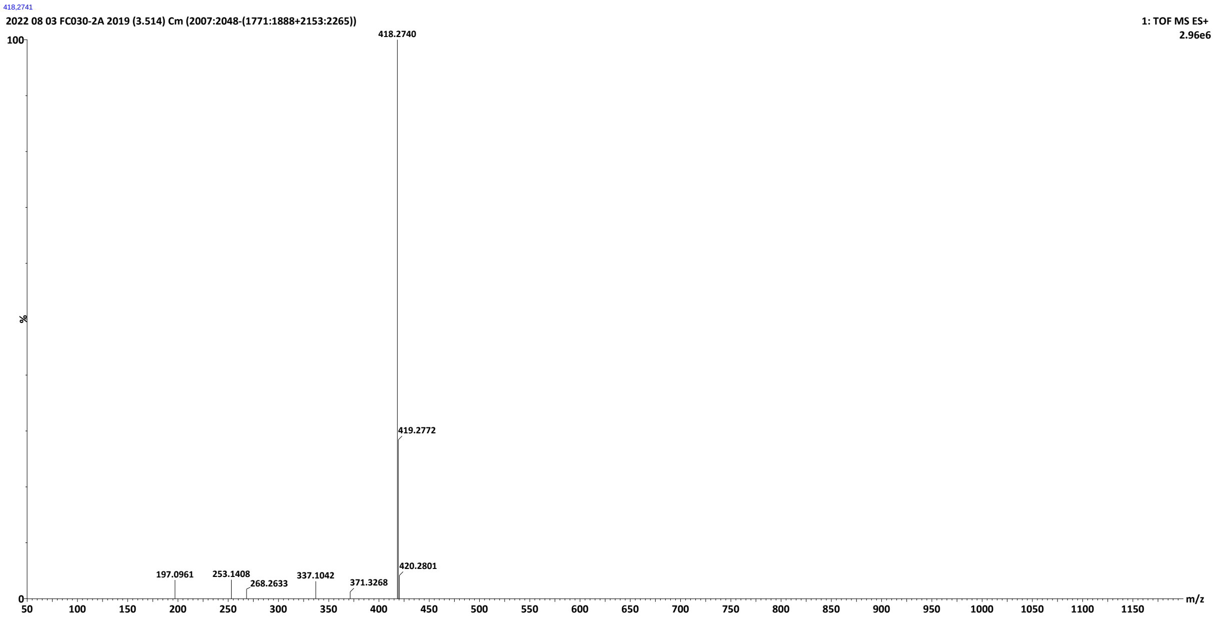
418.2741
2022 08 03 FC030-2A 2019 (3.514) Cm (2007:2048-(1771:1888+2153:2265))
1: TOF MS ES+
2.96e6
418.2740
419.2772
420.2801
197.0961
253.1408
268.2633
337.1042
371.3268
%
m/z

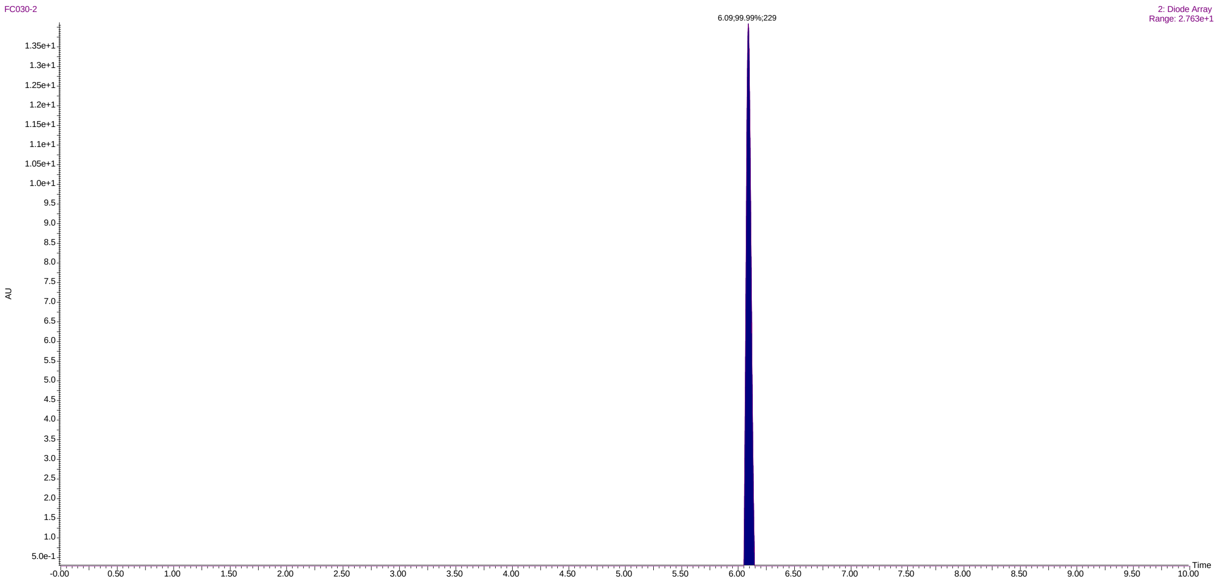
FC030-2
2: Diode Array
Range: 2.763e+1
6.09;99.99%;229
AU
Time

Benzyl({[4-(benzyloxy)phenyl]methyl})amine (35):

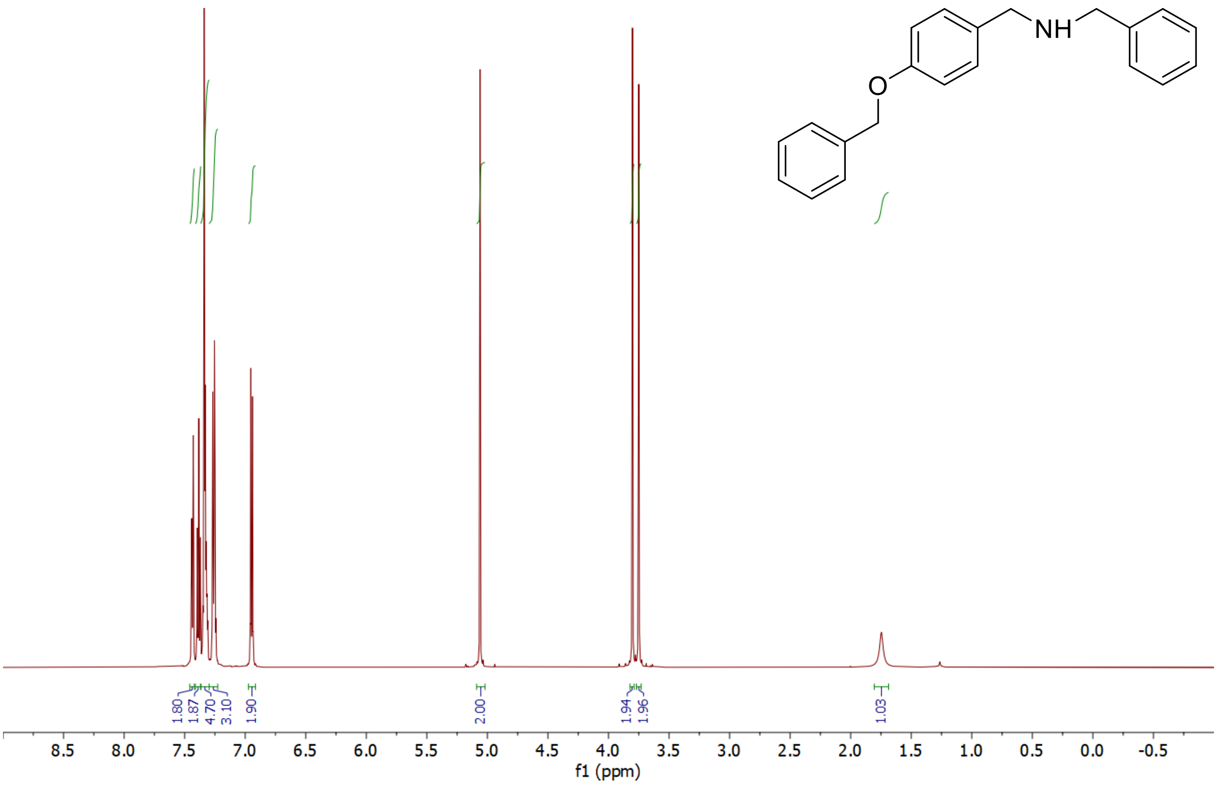

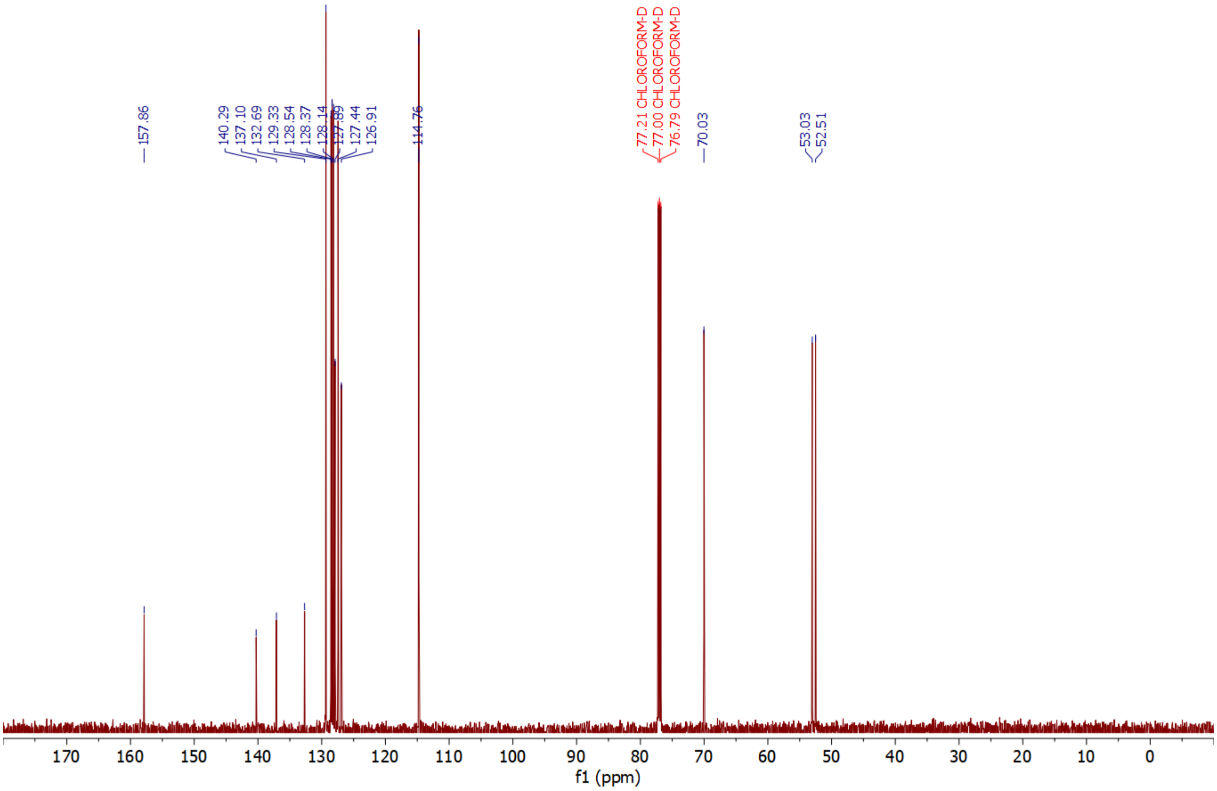

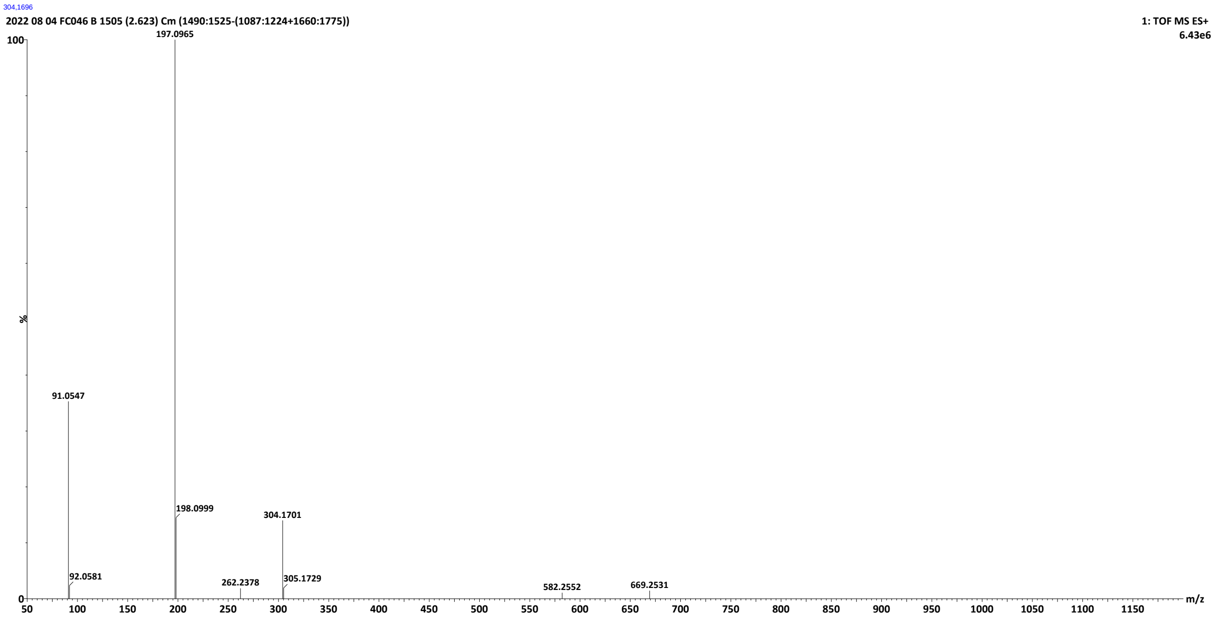
304.1696
2022 08 04 FC046 B 1505 (2.623) Cm (1490:1525-(1087:1224+1660:1775))
1: TOF MS ES+
6.43e6
197.0965
91.0547
198.0999
304.1701
92.0581
262.2378
305.1729
582.2552
669.2531
%
m/z

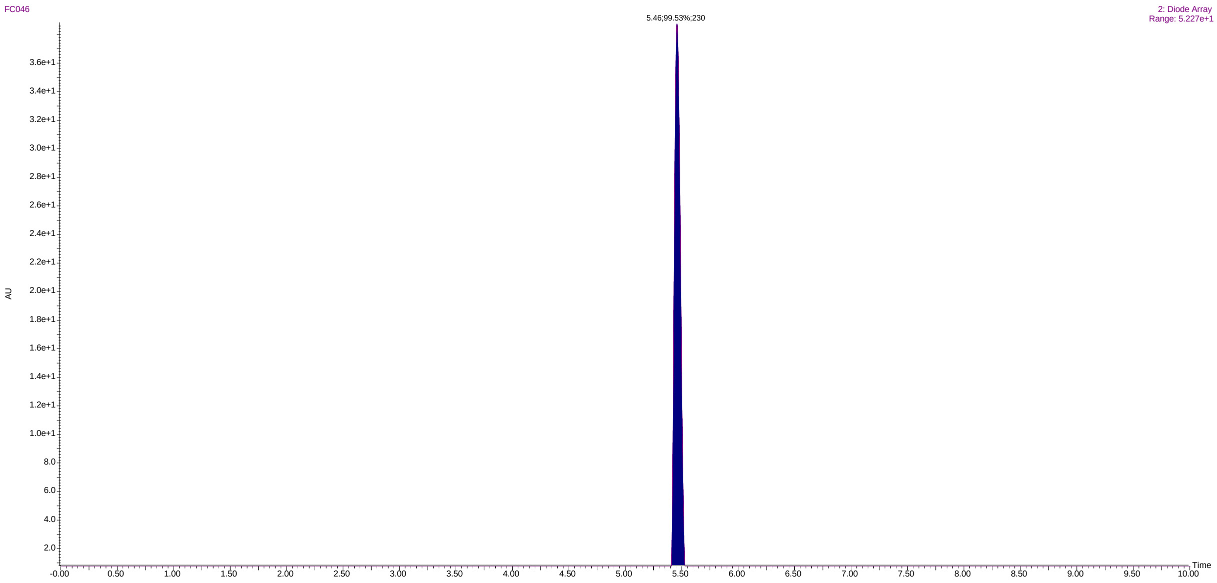
FC046
2: Diode Array
Range: 5.227e+1
5.46;99.53%;230
AU
Time

{[4-(Benzyloxy)phenyl]methyl}[(pyridin-2-yl)methyl]amine (36):

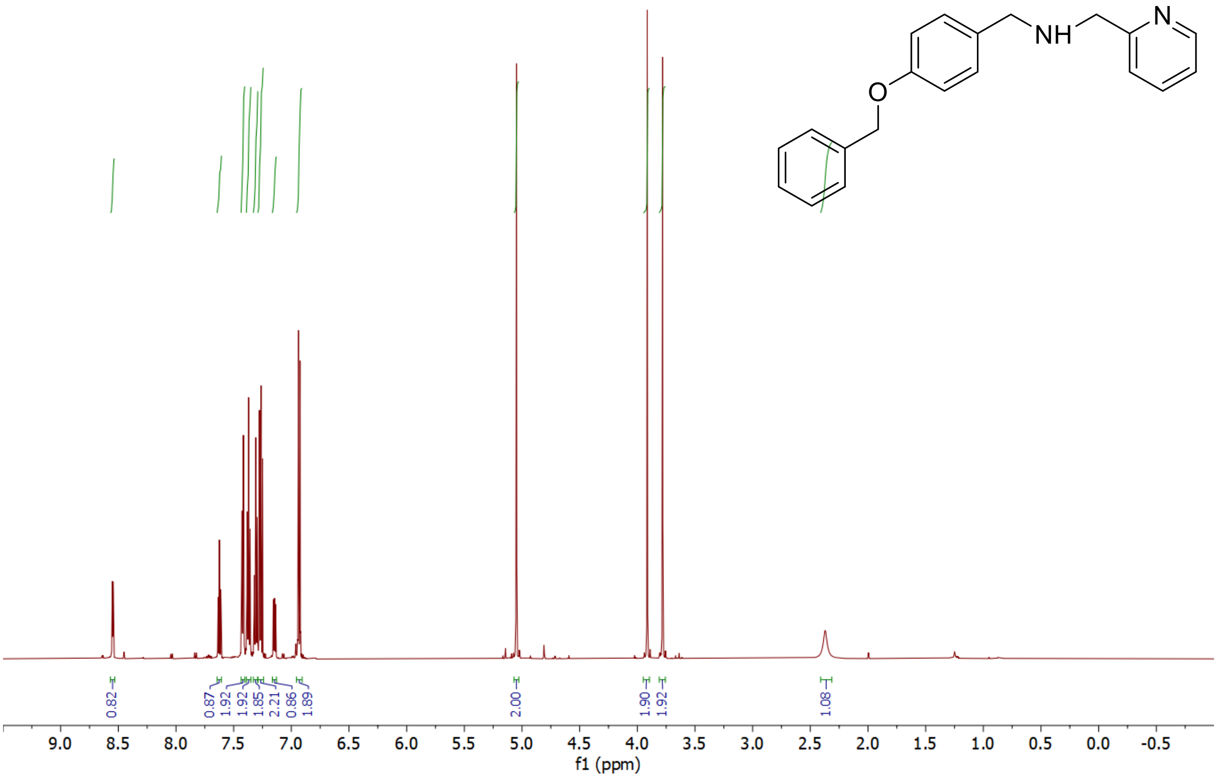

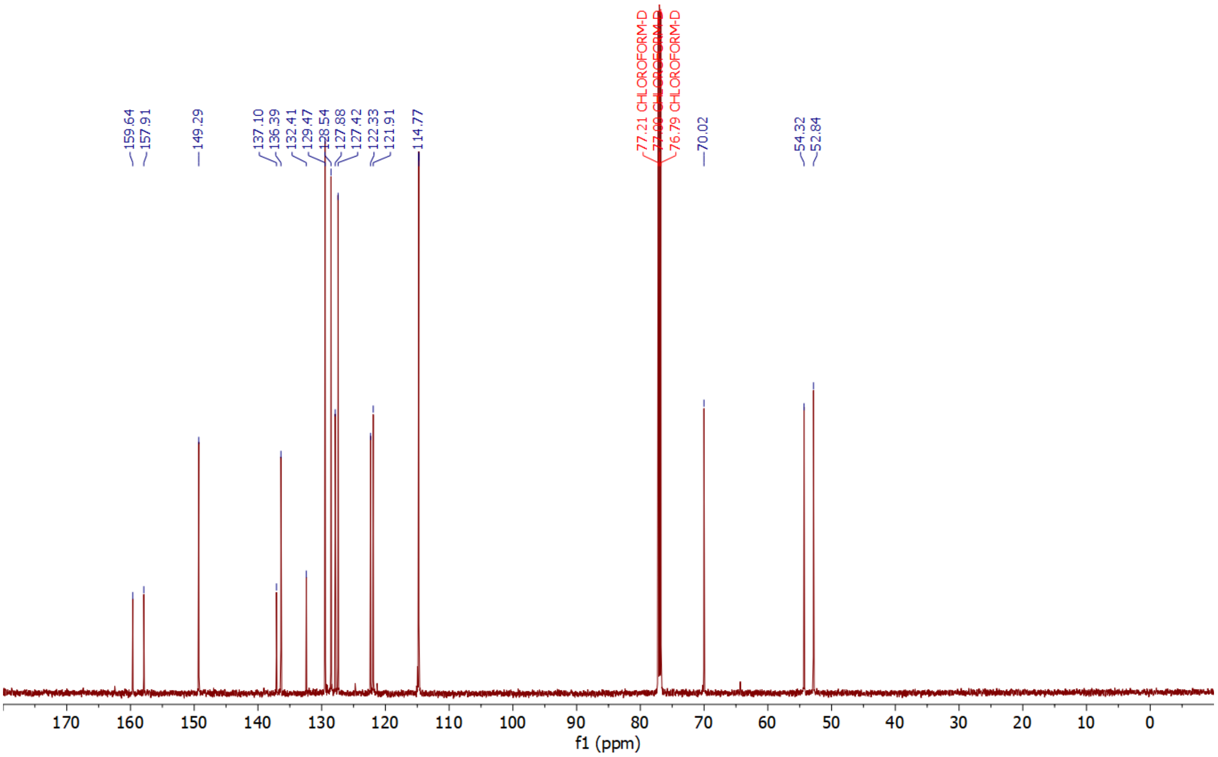

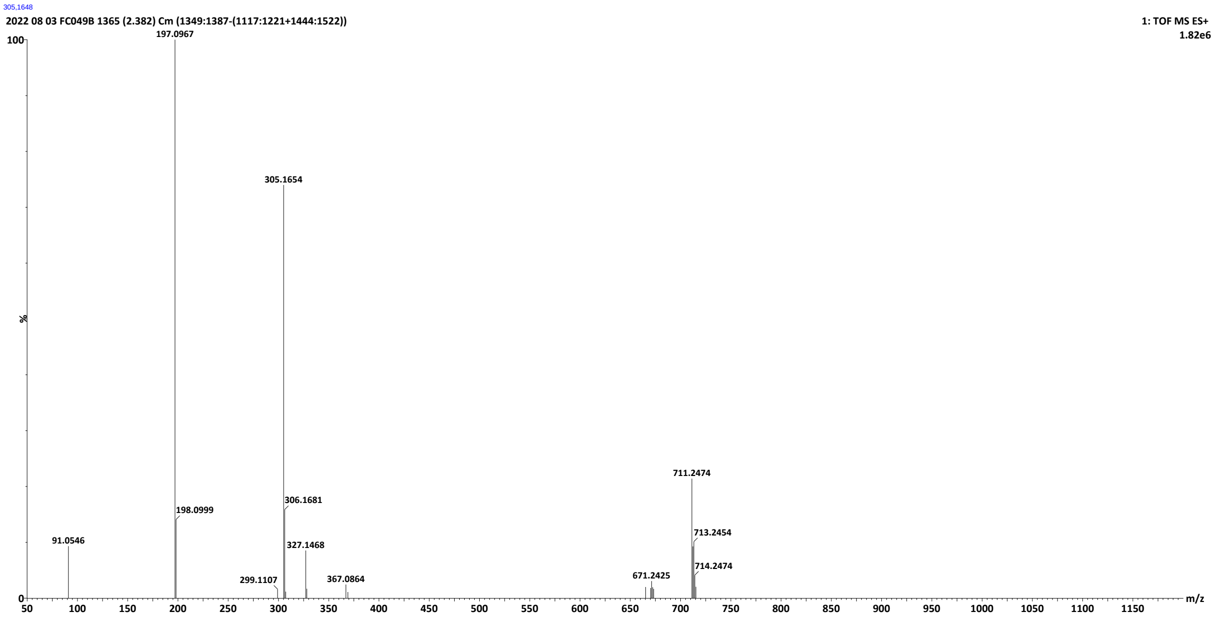
305.1648
2022 08 03 FC049B 1365 (2.382) Cm (1349:1387-(1117:1221+1444:1522))
1: TOF MS ES+
1.82e6
197.0967
305.1654
711.2474
198.0999
306.1681
713.2454
91.0546
327.1468
714.2474
671.2425
299.1107
367.0864
%
m/z

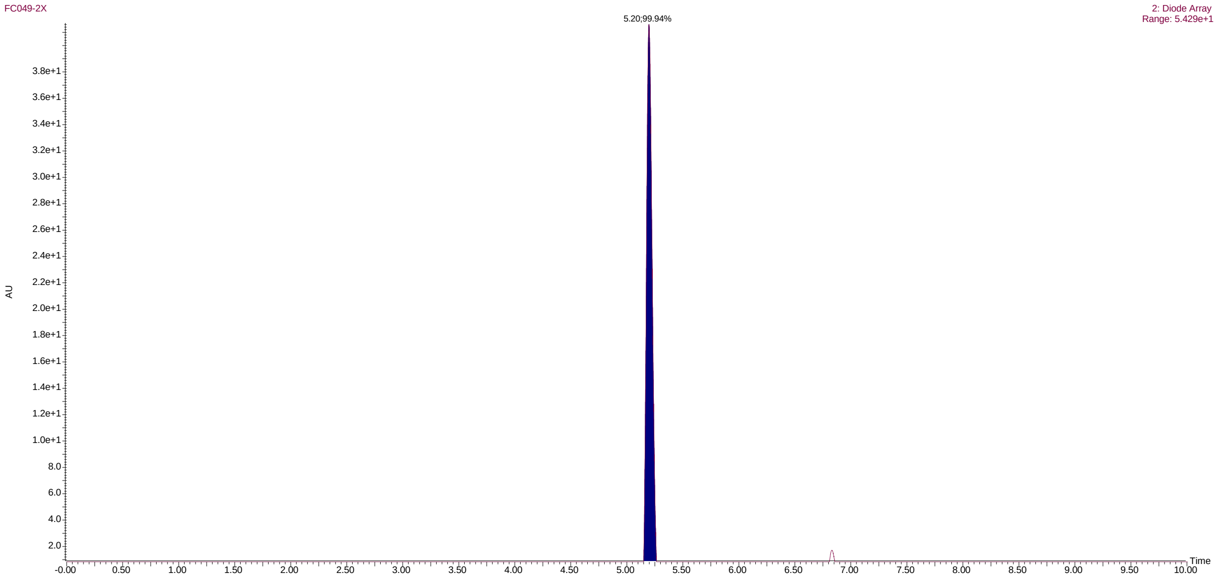
FC049-2X
2: Diode Array
Range: 5.429e+1
5.20;99.94%
AU
Time

{[4-(Benzyloxy)phenyl]methyl}(2-phenylethyl)amine (37):

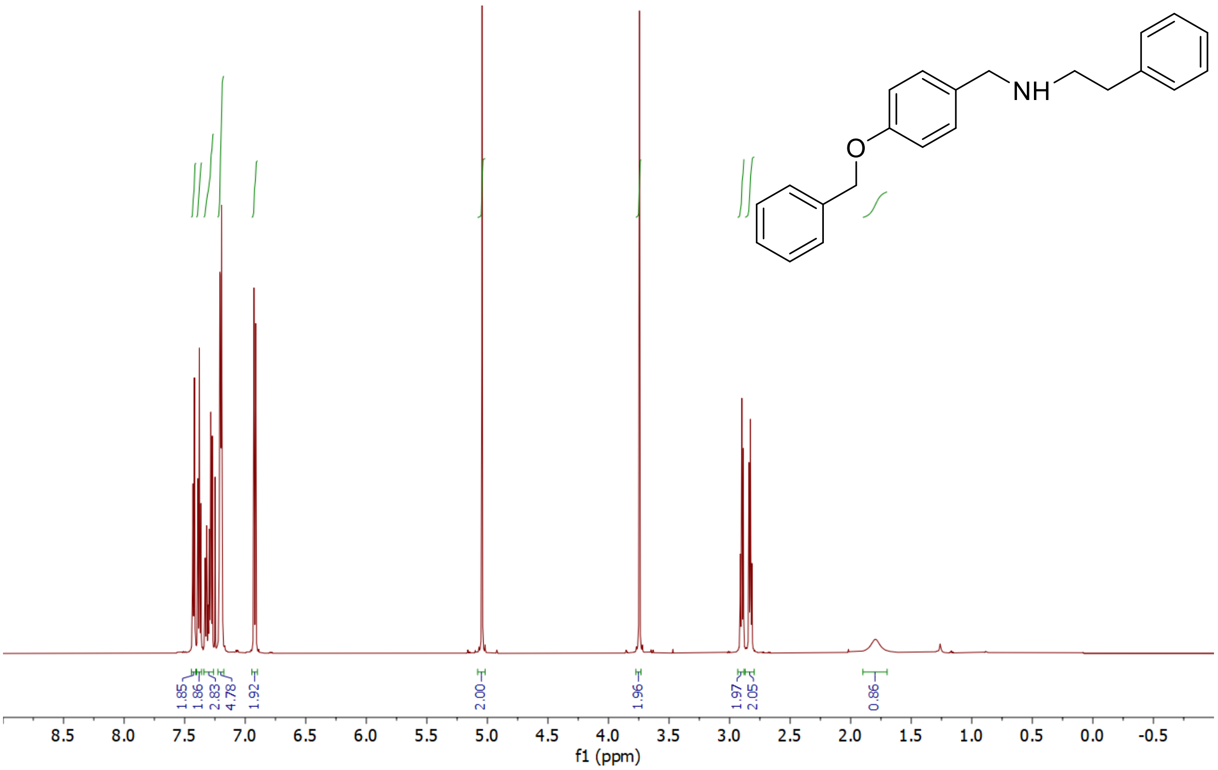

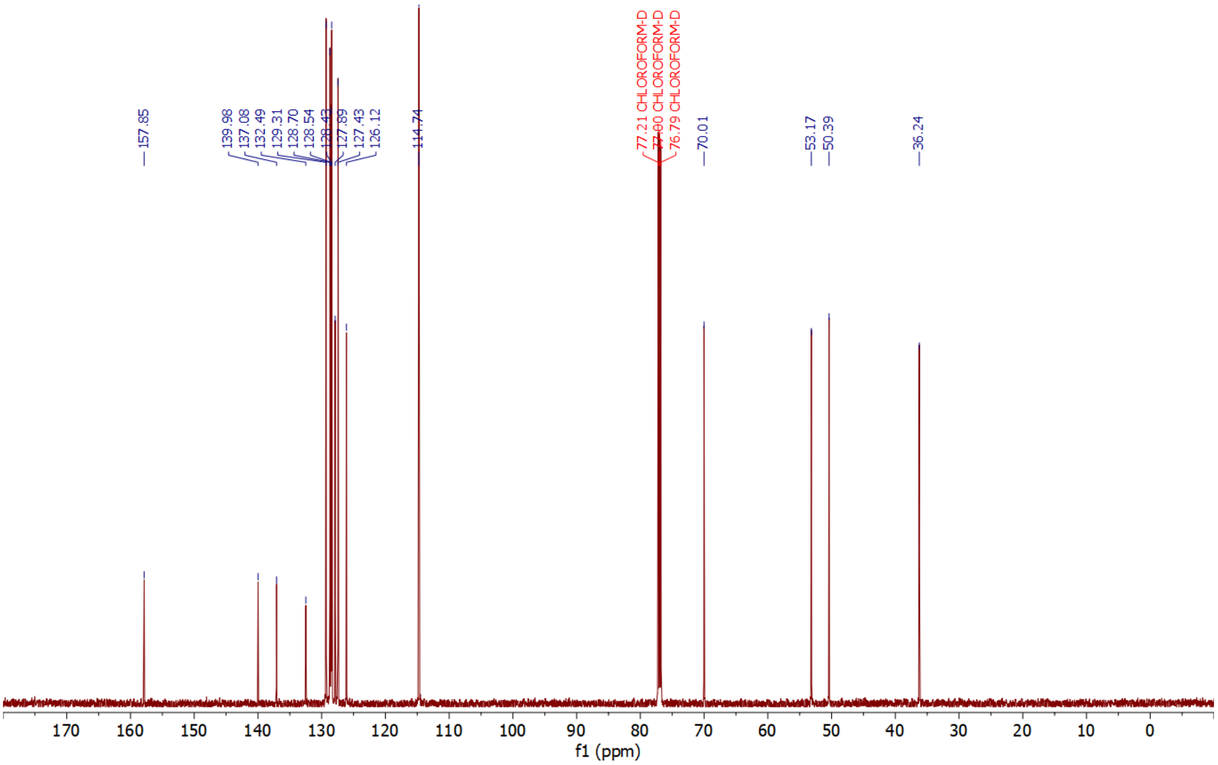

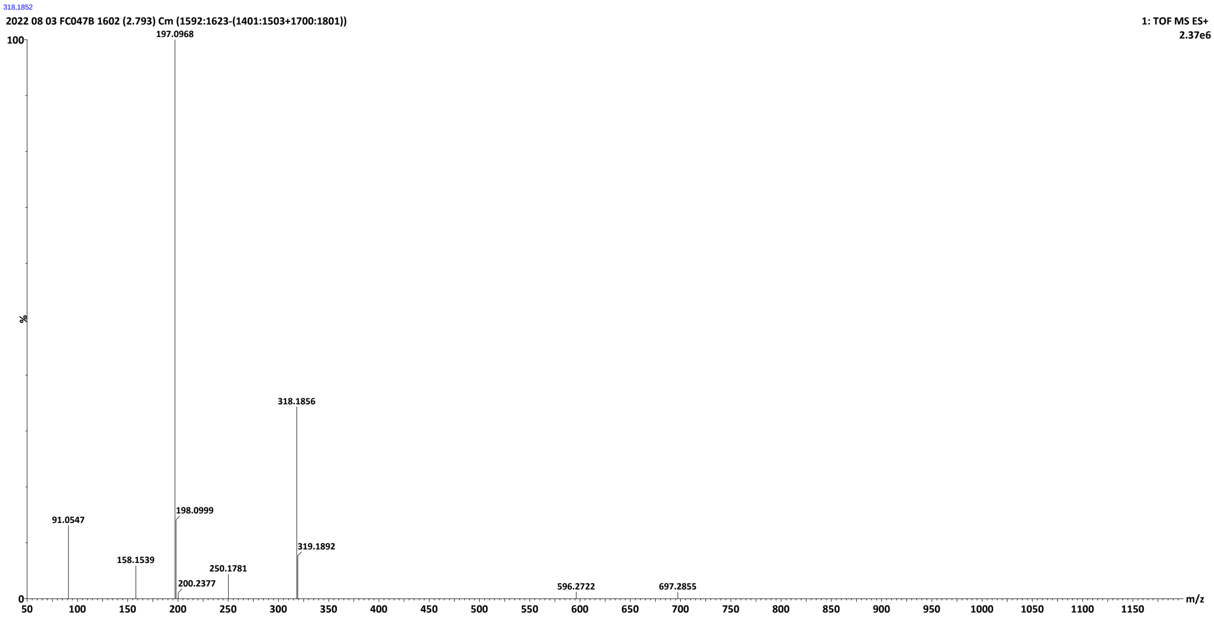
318.1852
2022 08 03 FC047B 1602 (2.793) Cm (1592:1623-(1401:1503+1700:1801))
1: TOF MS ES+
2.37e6
197.0968
318.1856
91.0547
198.0999
319.1892
158.1539
250.1781
200.2377
596.2722
697.2855
%
m/z

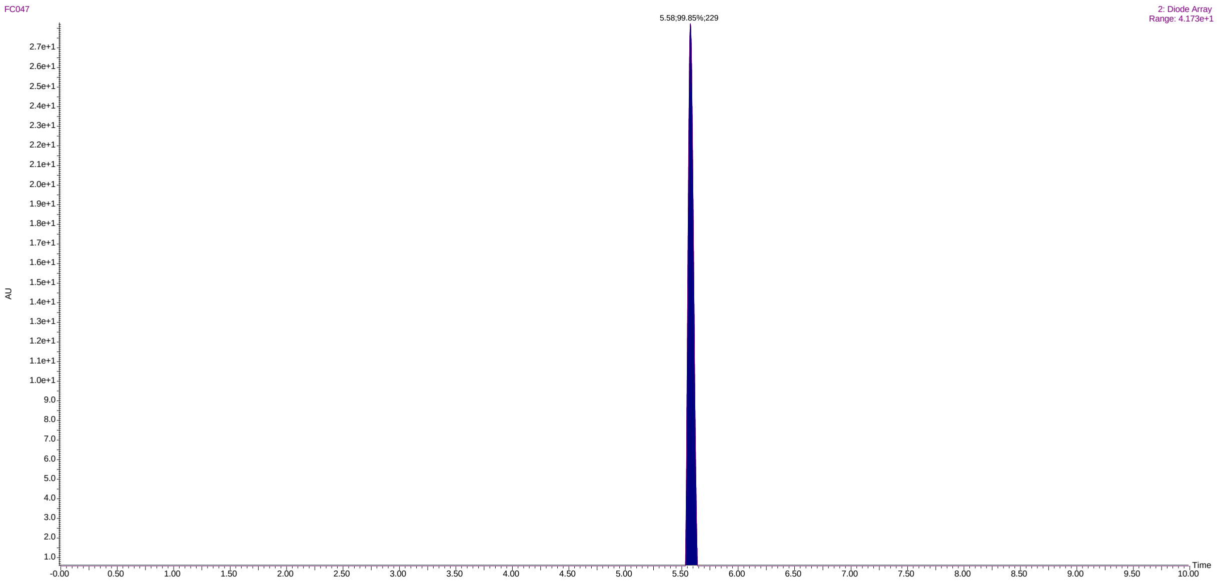
FC047
2: Diode Array
Range: 4.173e+1
5.58;99.85%;229
AU
Time

{[4-(Benzyloxy)phenyl]methyl}[2-(pyridin-2-yl)ethyl]amine (38):

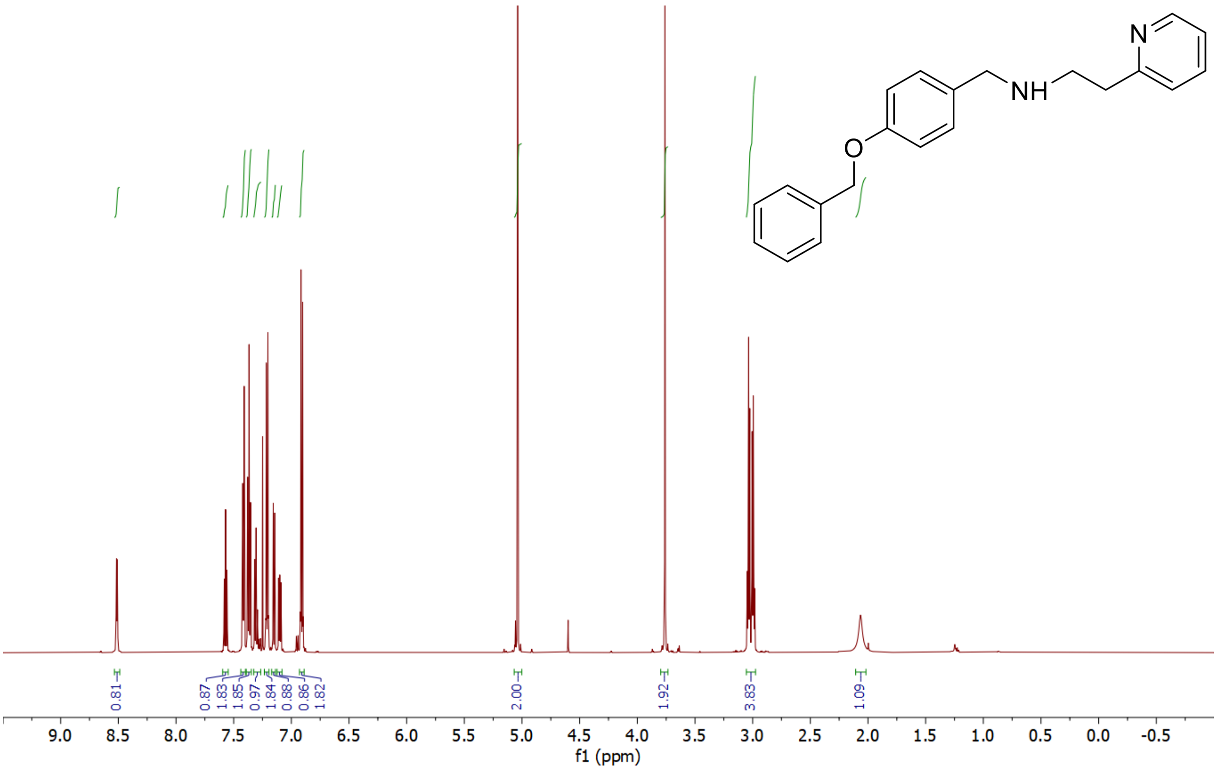

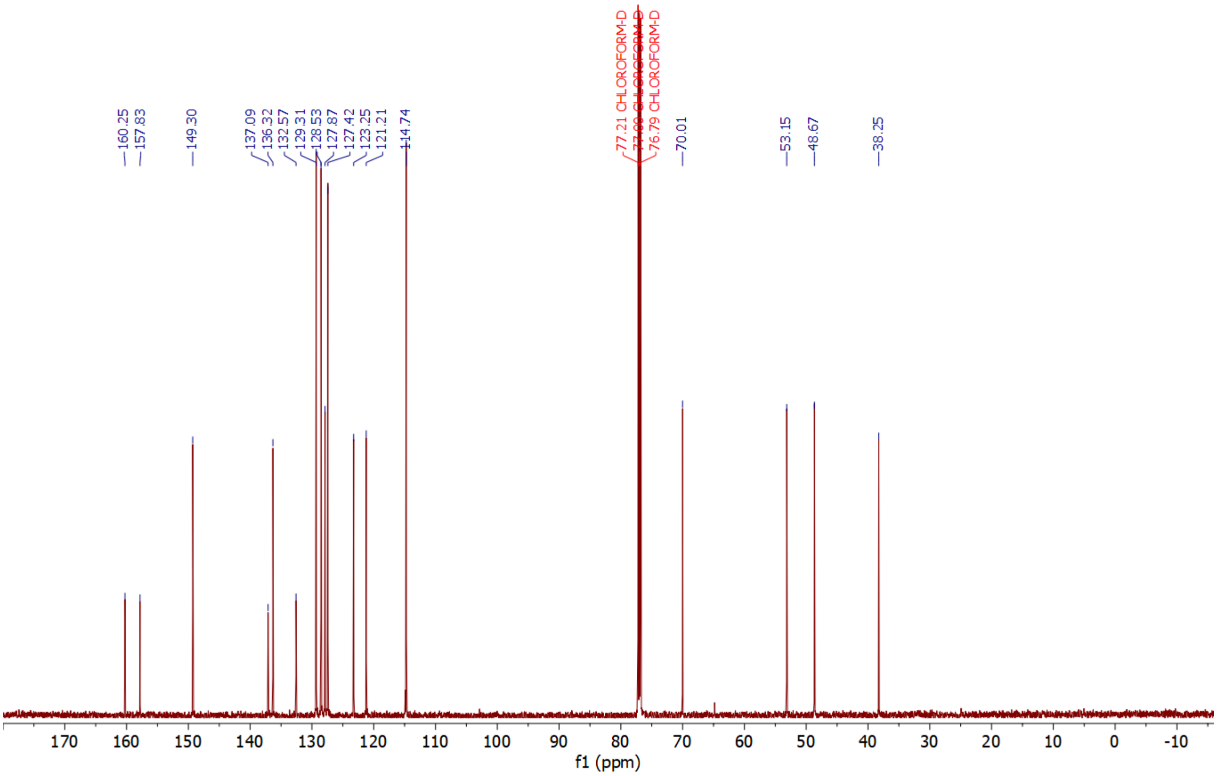

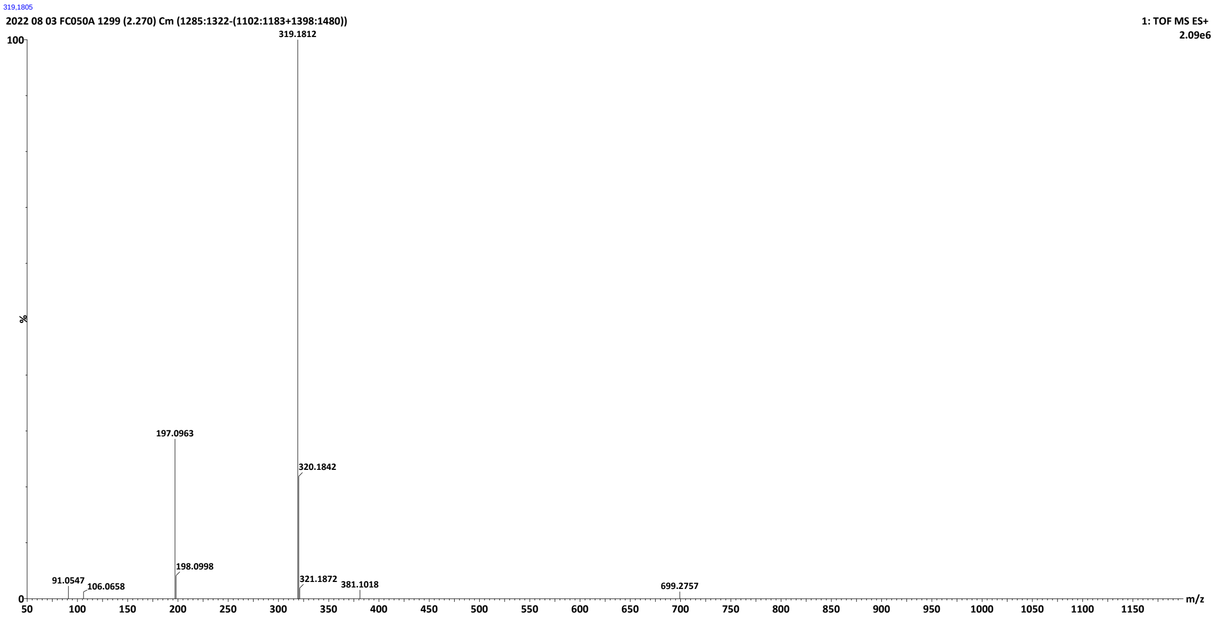
319.1805
2022 08 03 FC050A 1299 (2.270) Cm (1285:1322-(1102:1183+1398:1480))
1: TOF MS ES+
2.09e6
319.1812
197.0963
320.1842
198.0998
91.0547
106.0658
321.1872
381.1018
699.2757
%
m/z

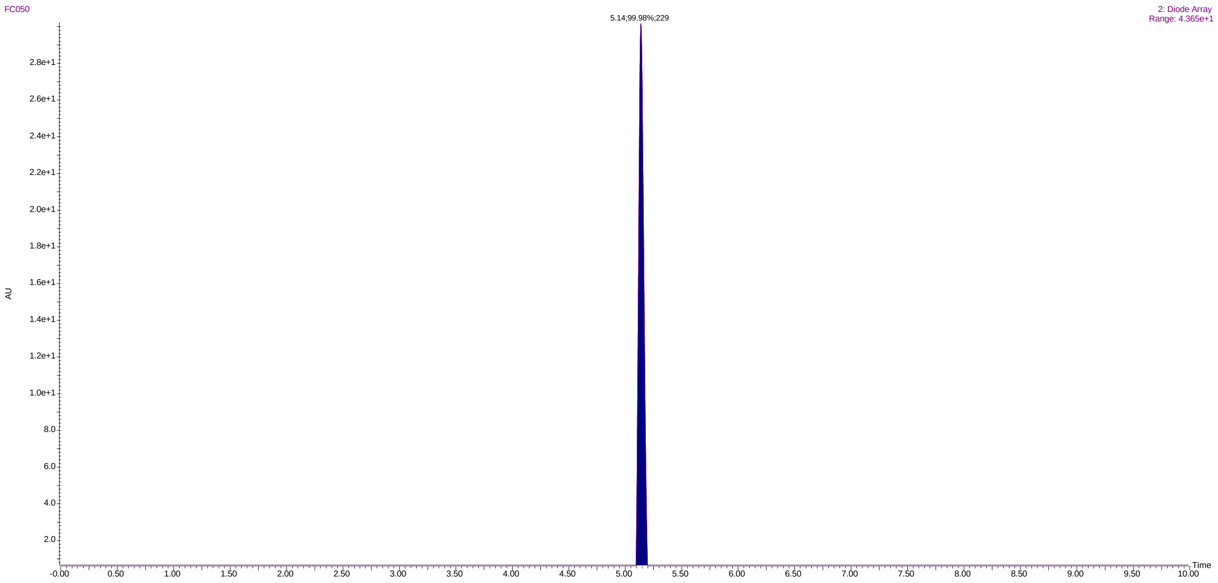
FC050
2: Diode Array
Range: 4.365e+1
5.14;99.98%;229
AU
Time

{[4-(Benzyloxy)phenyl]methyl}(3-phenylpropyl)amine (39):

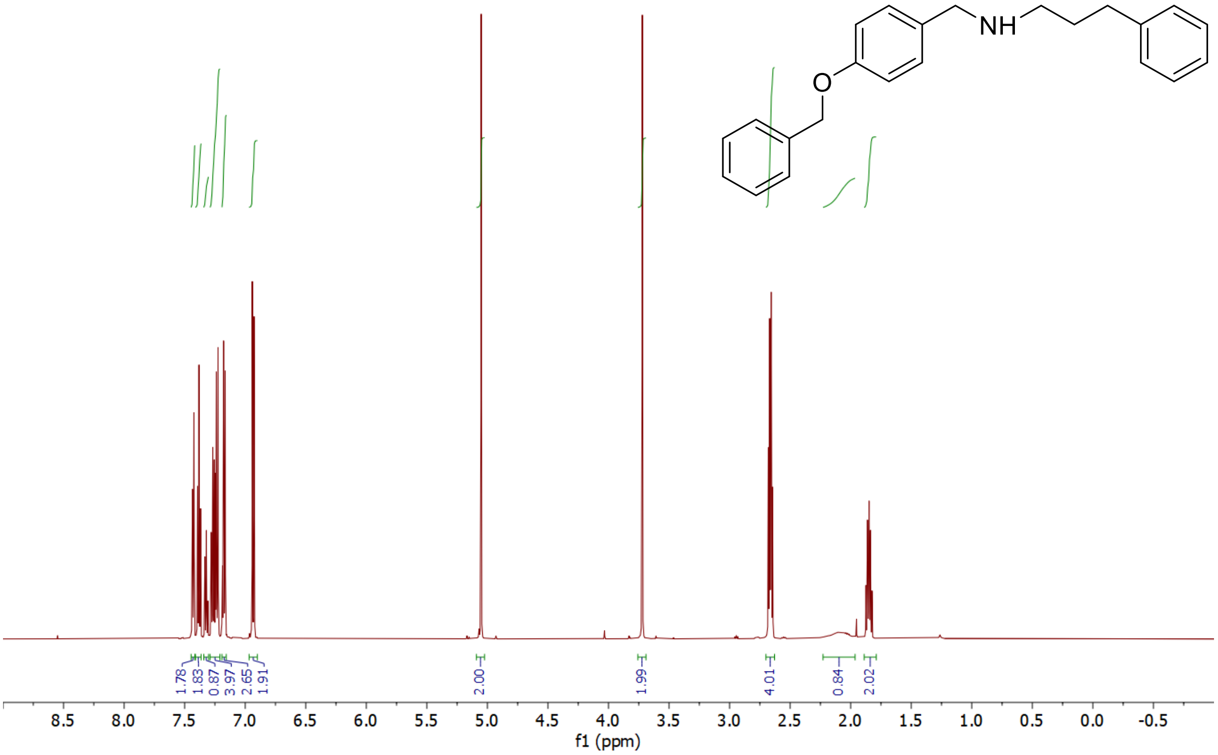

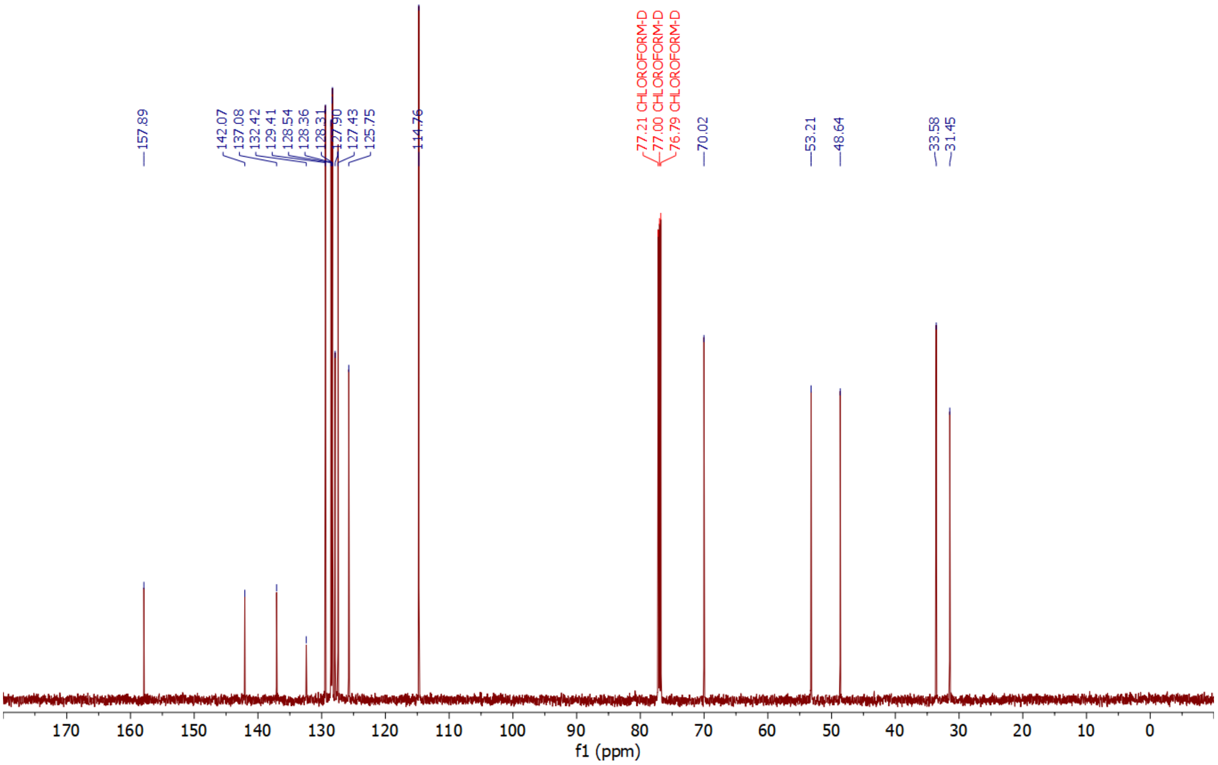

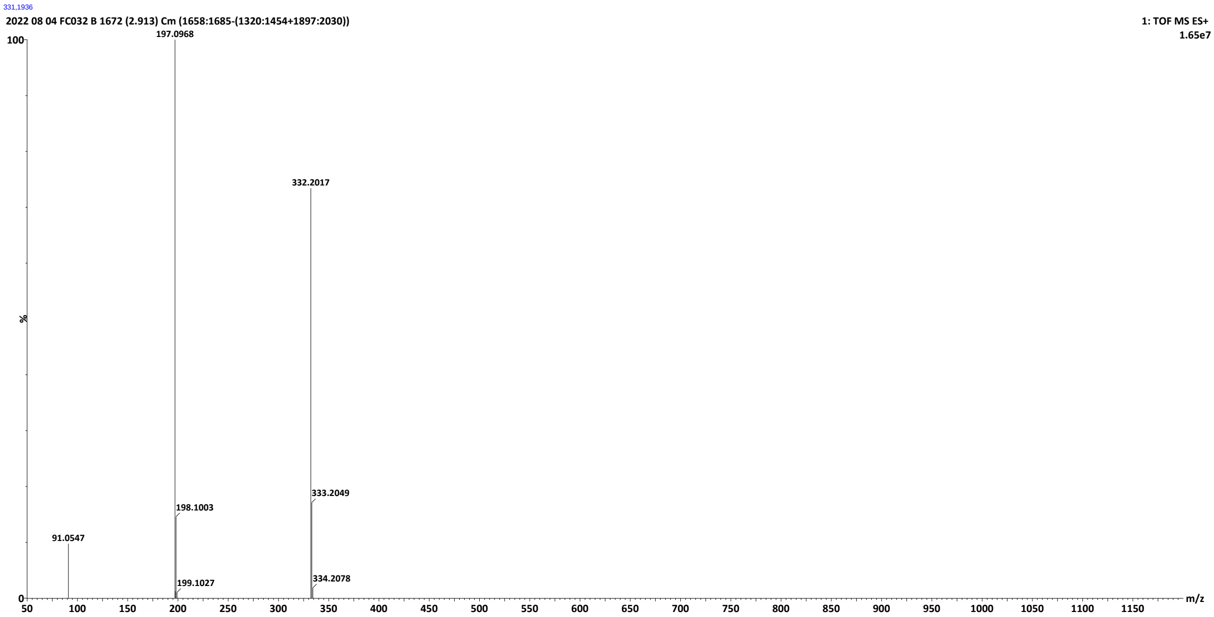
331.1936
2022 08 04 FC032 B 1672 (2.913) Cm (1658:1685-(1320:1454+1897:2030))
1: TOF MS ES+
1.65e7
197.0968
332.2017
333.2049
198.1003
91.0547
199.1027
334.2078
%
m/z

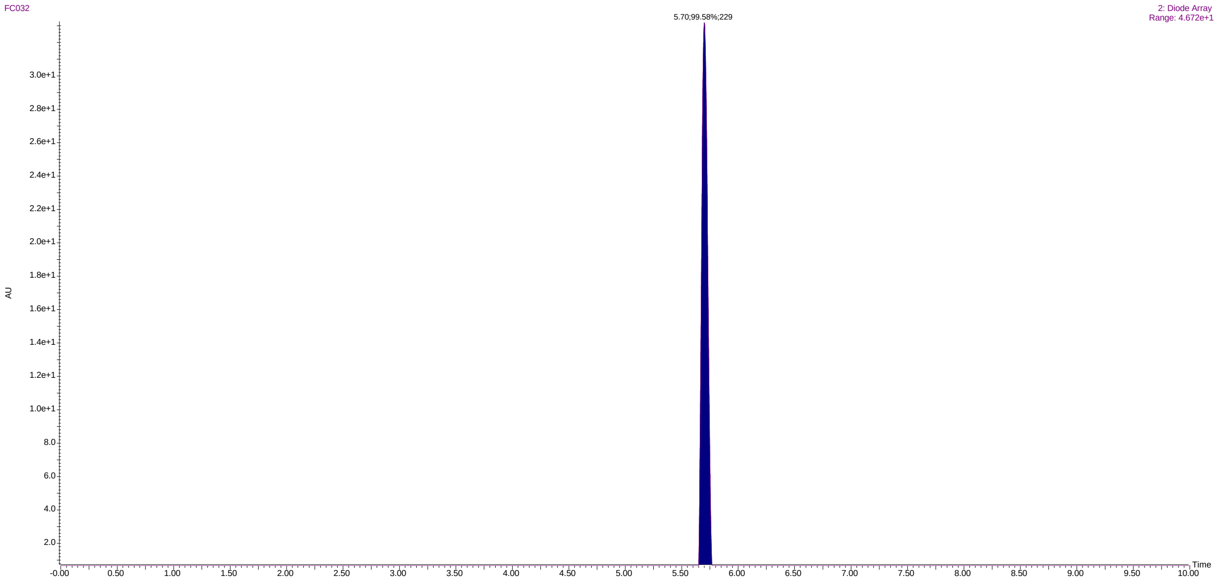
FC032
2: Diode Array
Range: 4.672e+1
5.70;99.58%;229
AU
Time

{[4-(Benzyloxy)phenyl]methyl}(4-phenylbutyl)amine (40):

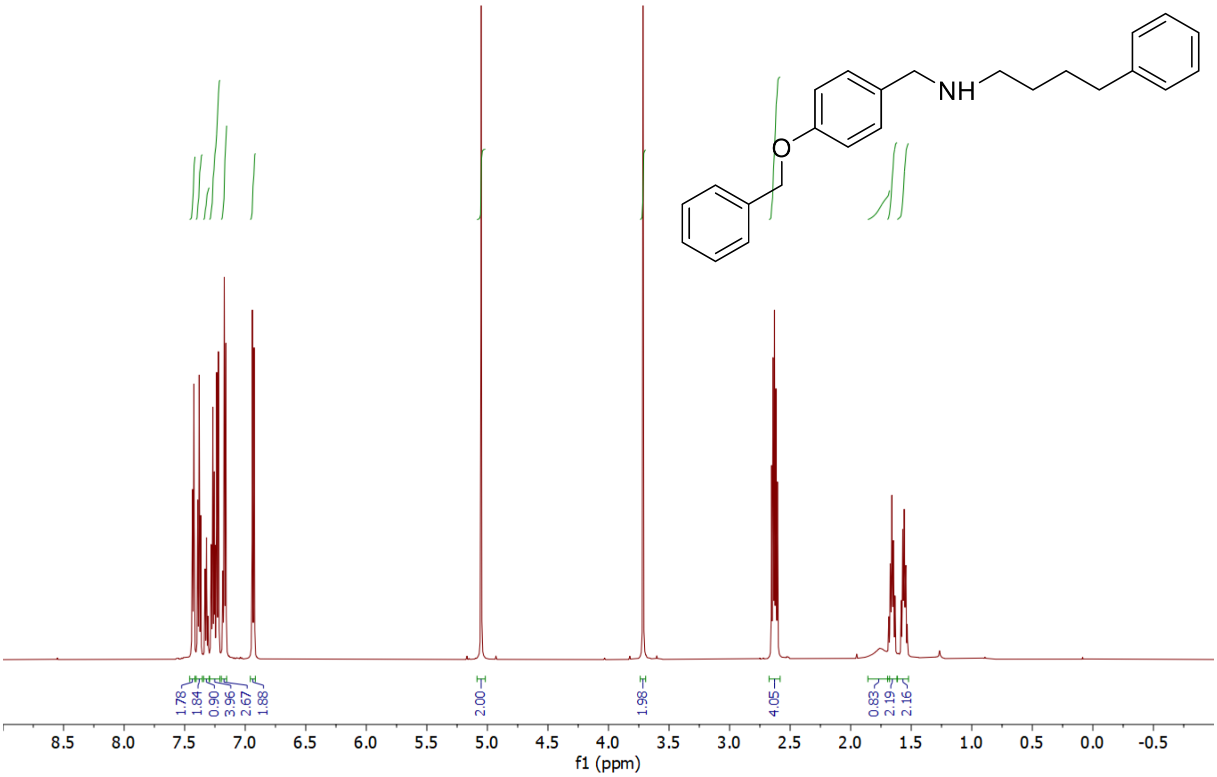

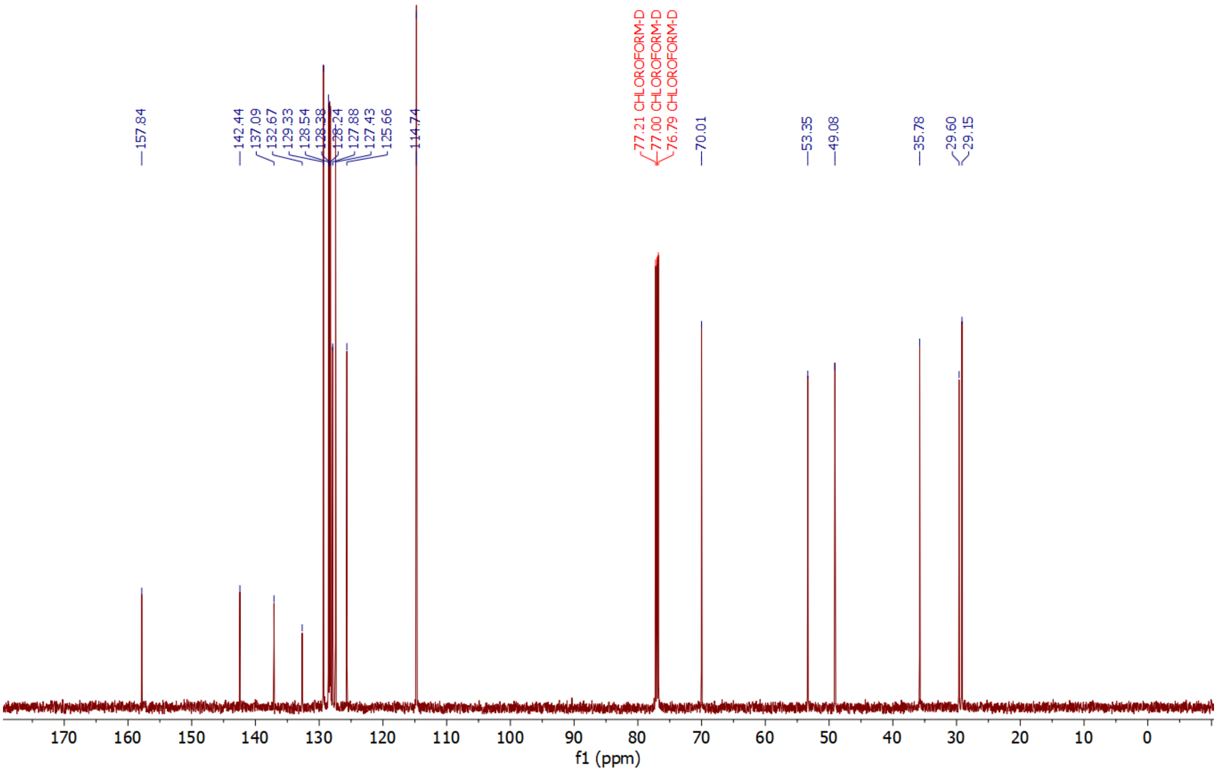

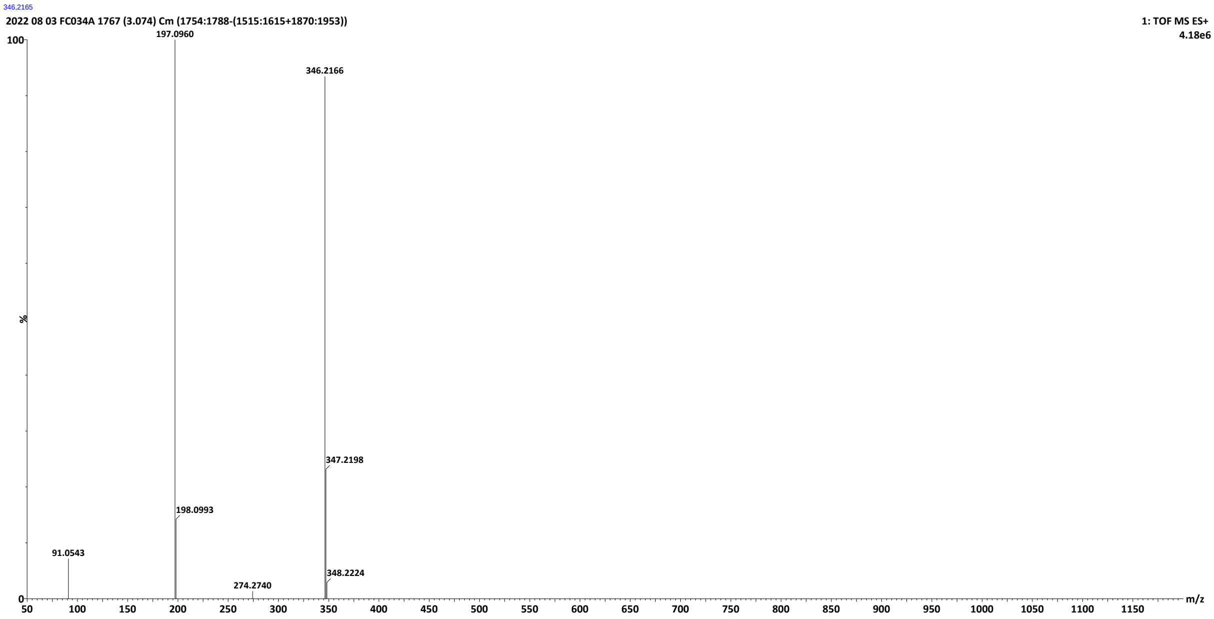
346.2165
2022 08 03 FC034A 1767 (3.074) Cm (1754:1788-(1515:1615+1870:1953))
1: TOF MS ES+
4.18e6
197.0960
346.2166
347.2198
198.0993
91.0543
348.2224
274.2740
%
m/z

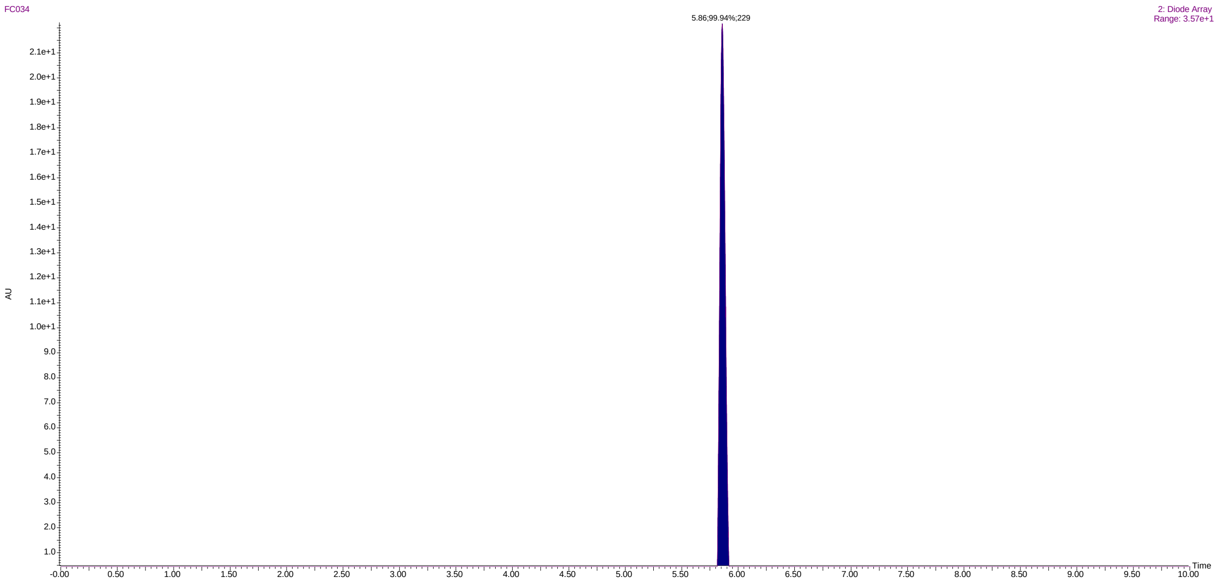
FC034
2: Diode Array
Range: 3.57e+1
5.86;99.94%;229
AU
Time

{[4-(Benzyloxy)phenyl]methyl}(2-phenylpropyl)amine (41):

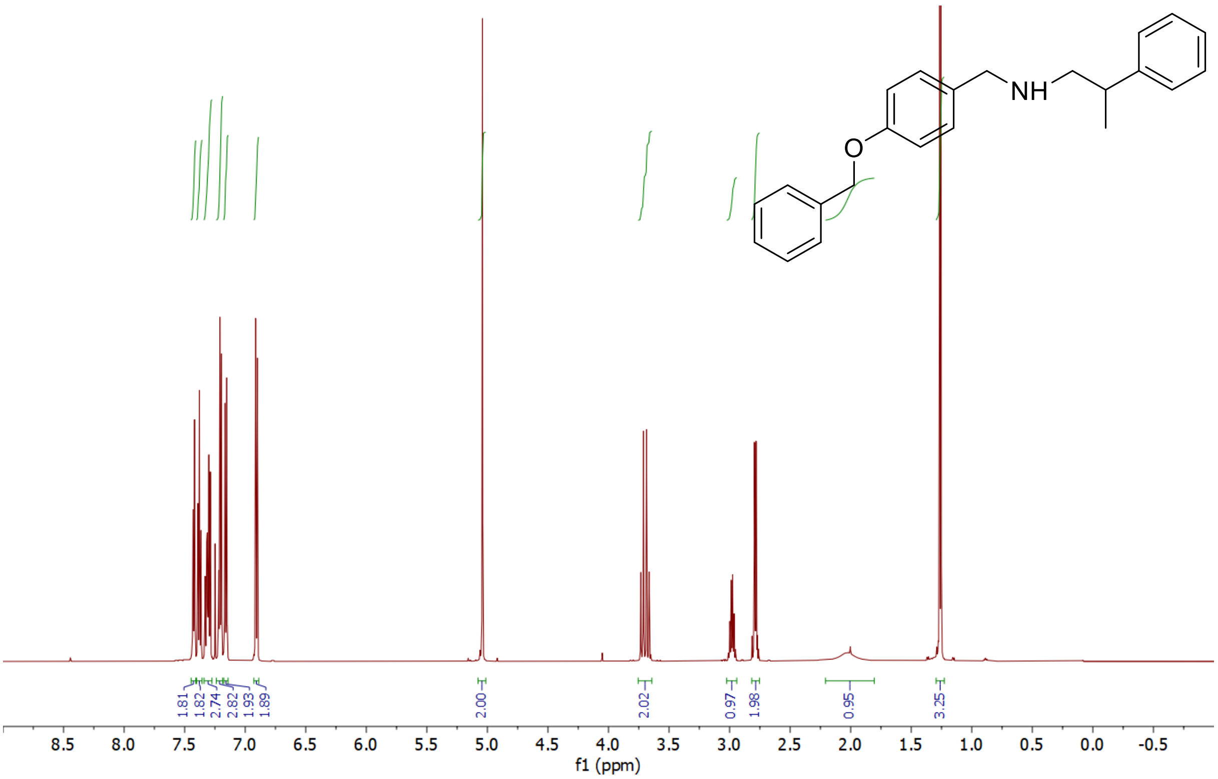

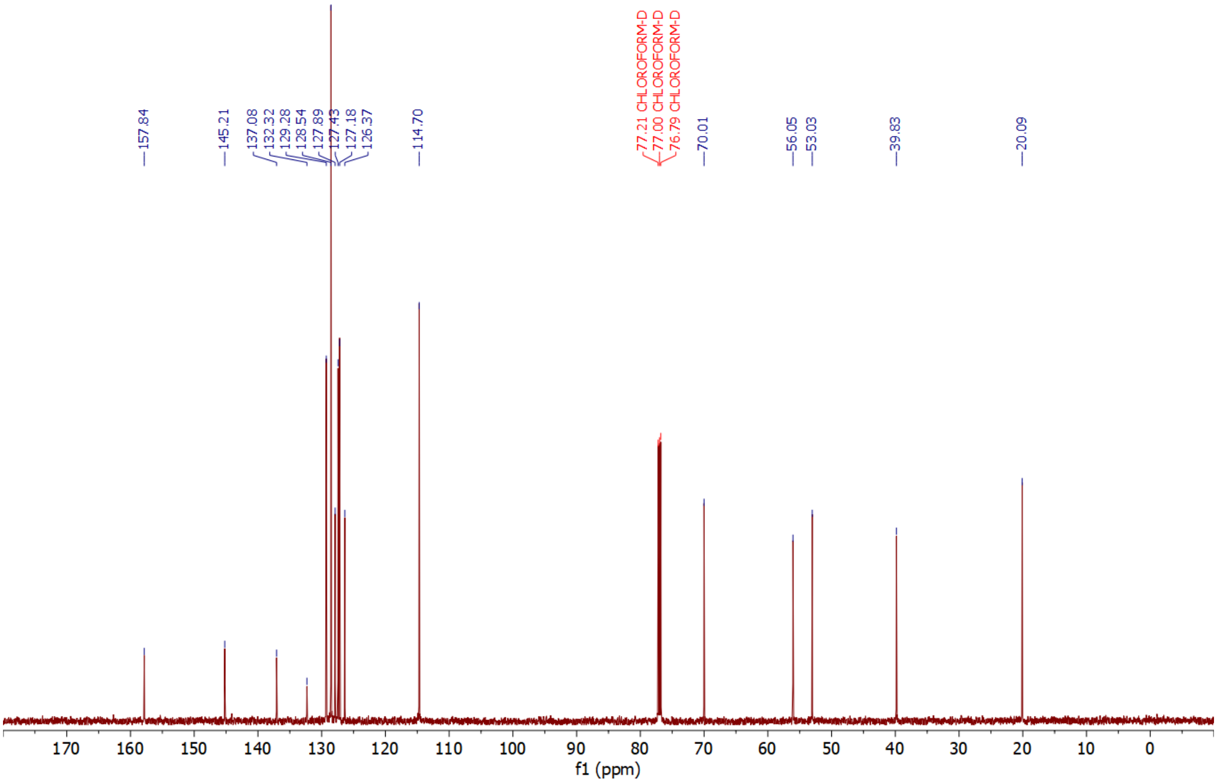

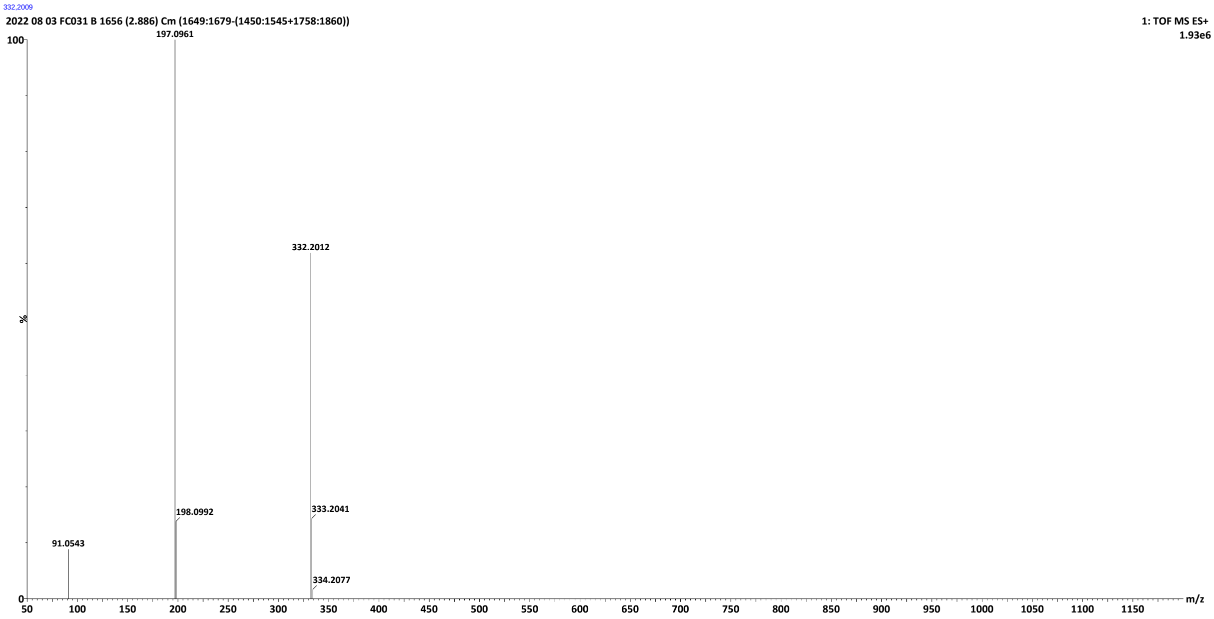
332.2009
2022 08 03 FC031 B 1656 (2.886) Cm (1649:1679-(1450:1545+1758:1860))
1: TOF MS ES+
1.93e6
197.0961
332.2012
198.0992
333.2041
91.0543
334.2077
%
m/z

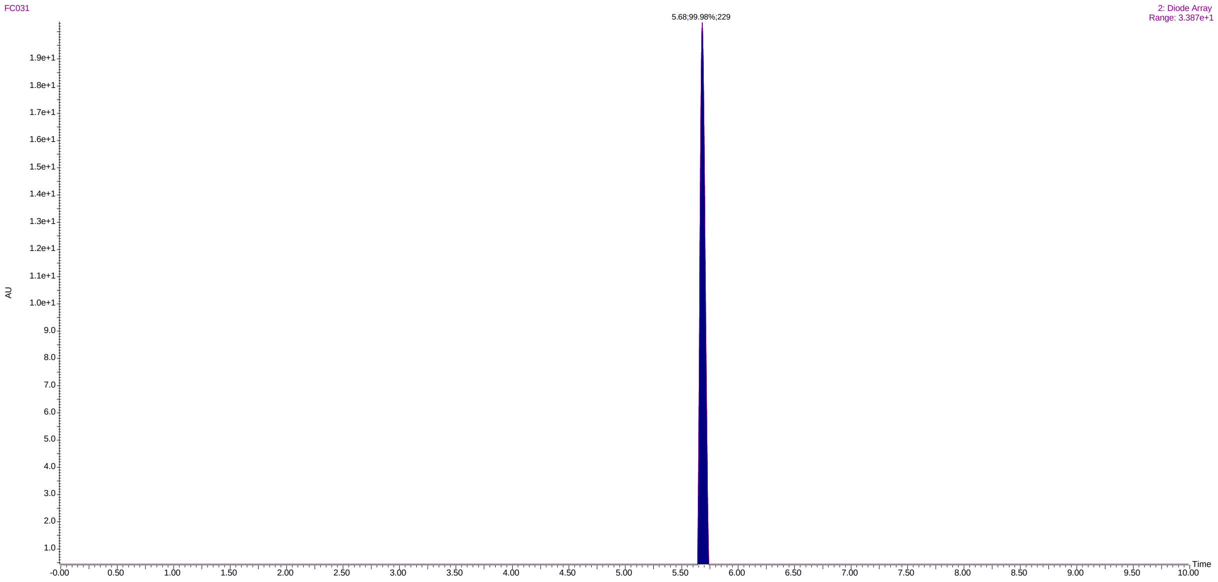
FC031
2: Diode Array
Range: 3.387e+1
5.68;99.98%;229
AU
Time

{[4-(Benzyloxy)phenyl]methyl}(4-phenylbutan-2-yl)amine (42):

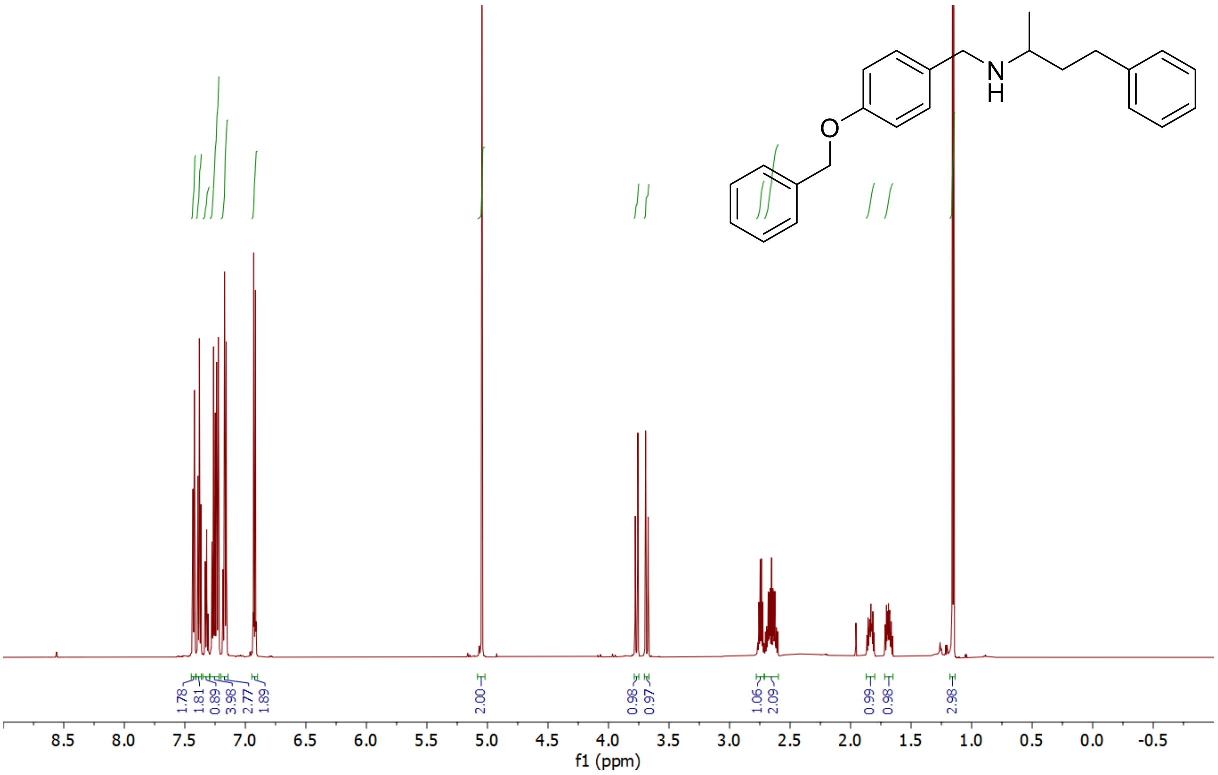

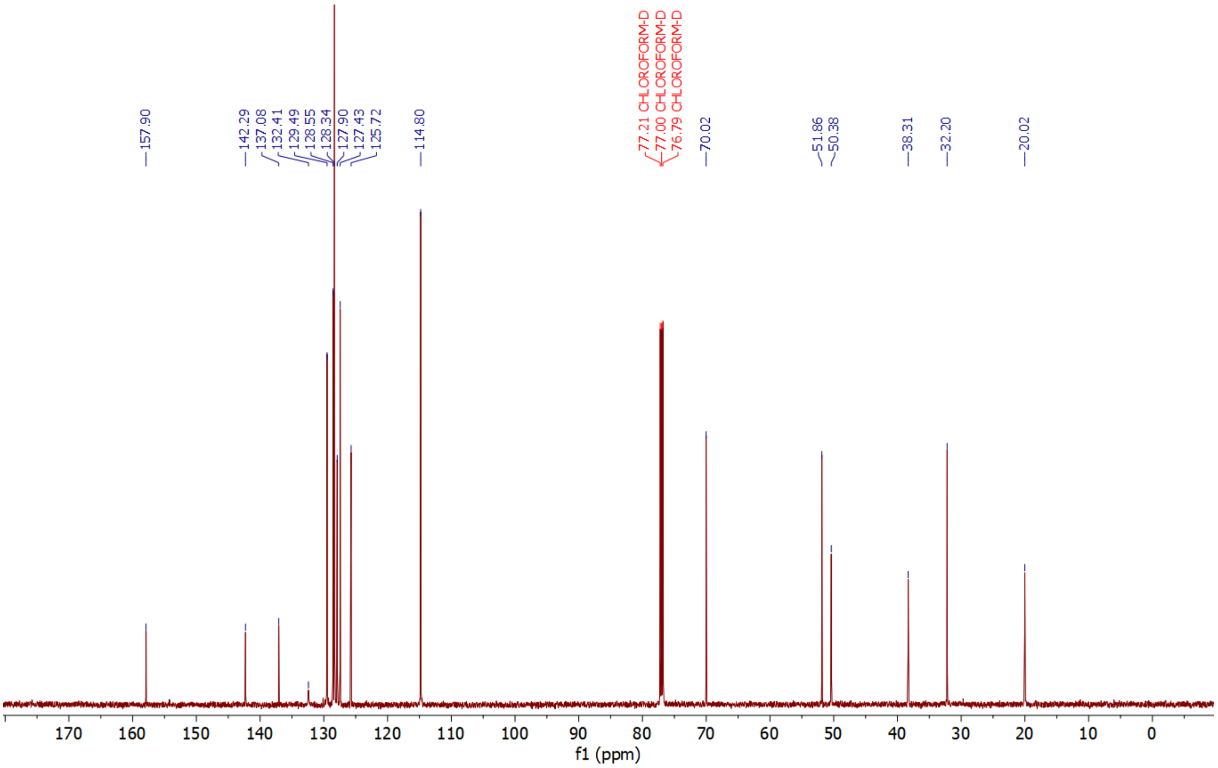

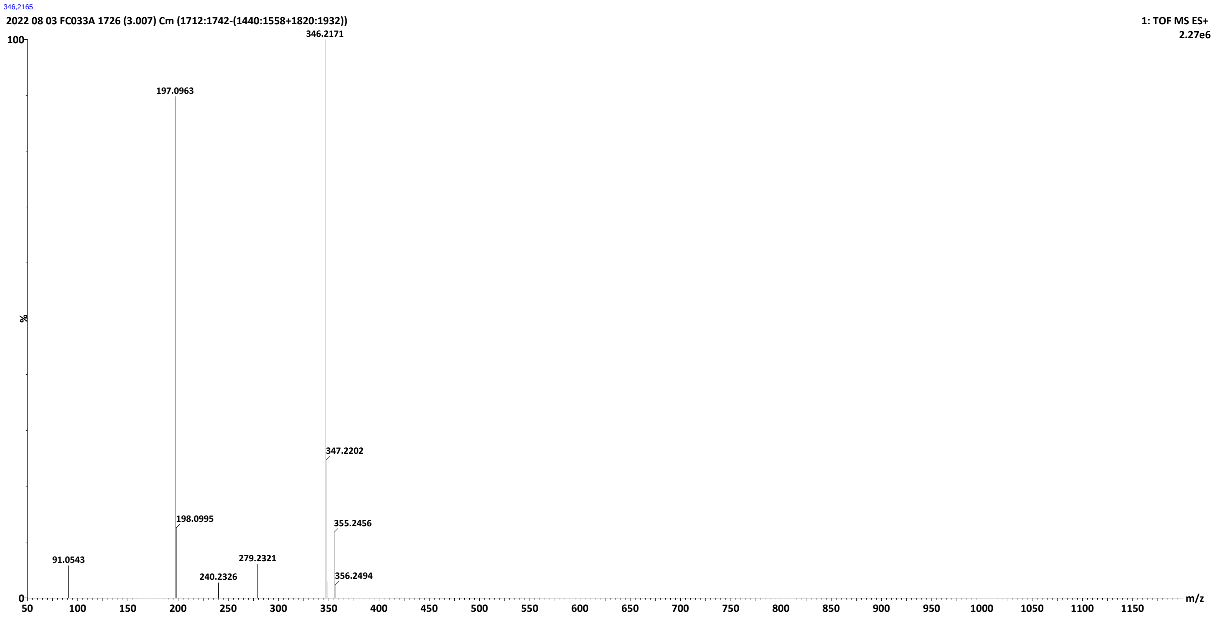

346.2165
2022 08 03 FC033A 1726 (3.007) Cm (1712:1742-(1440:1558+1820:1932))
1: TOF MS ES+
2.27e6
346.2171
197.0963
347.2202
198.0995
355.2456
91.0543
279.2321
240.2326
356.2494
%
m/z

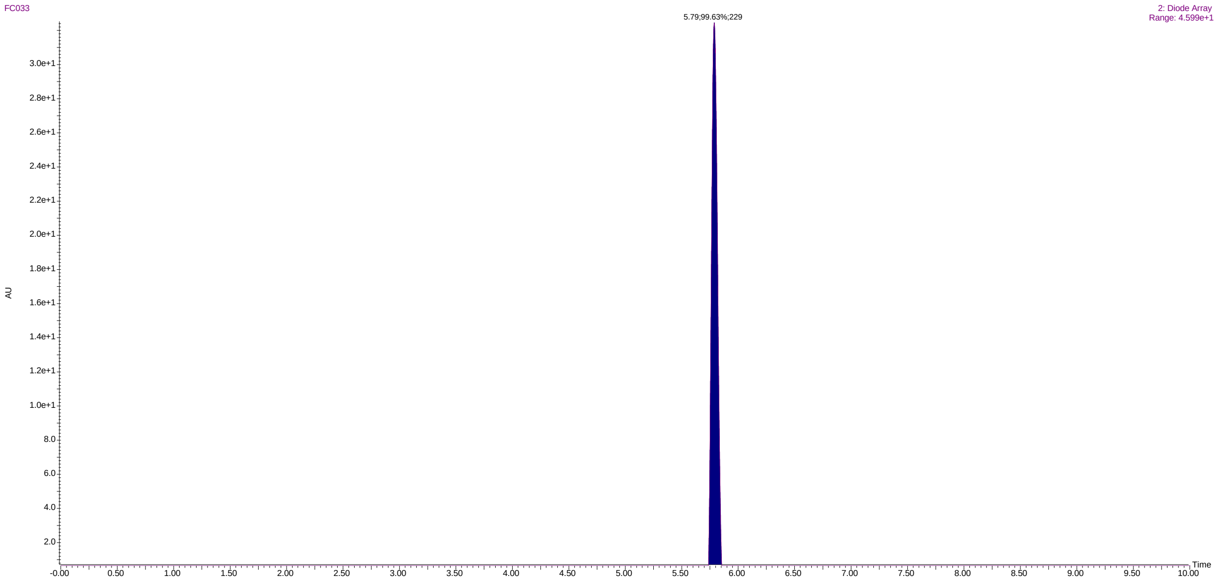

FC033
2: Diode Array
Range: 4.599e+1
5.79;99.63%;229
AU
Time

Benzyl({[4-(benzyloxy)phenyl]methyl})ethylamine (43):

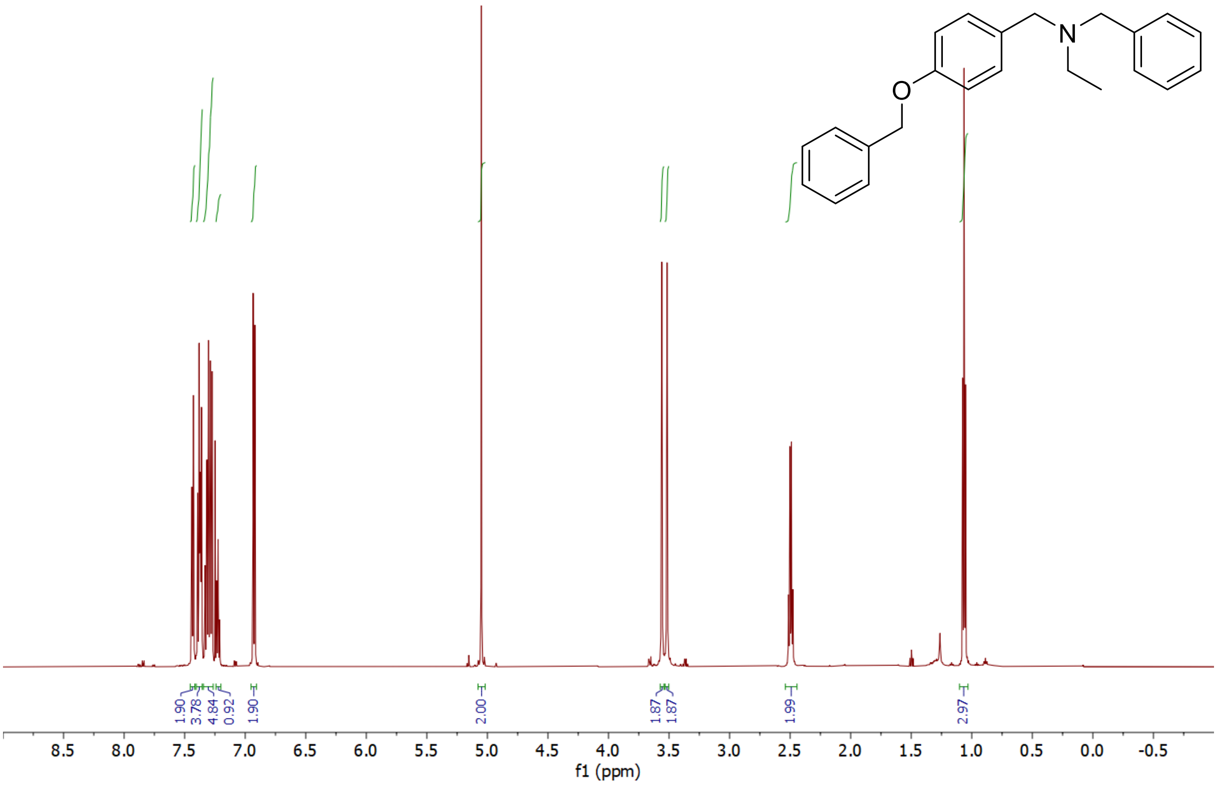

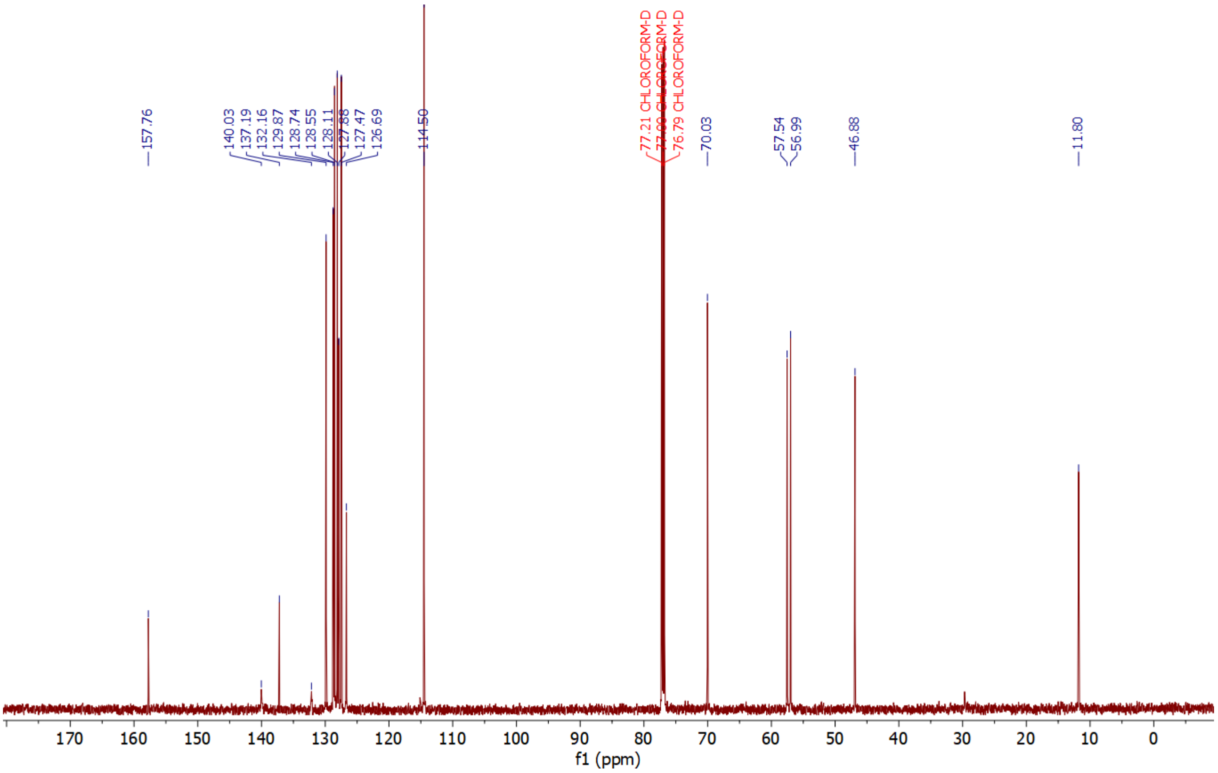

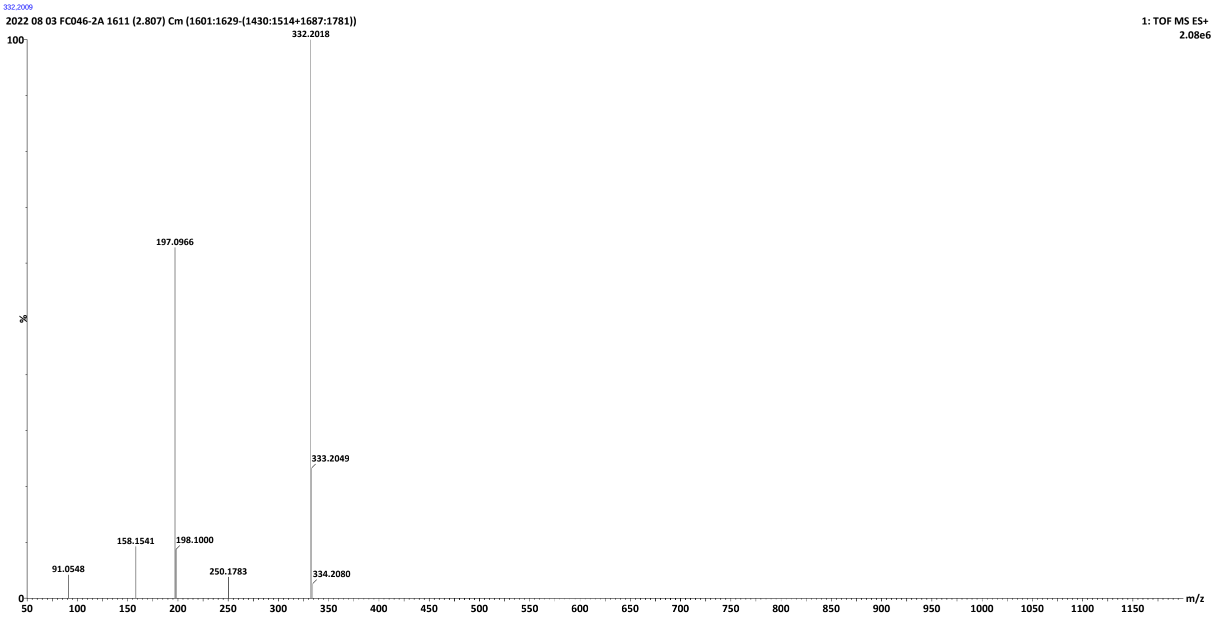
332.2009
2022 08 03 FC046-2A 1611 (2.807) Cm (1601:1629-(1430:1514+1687:1781))
1: TOF MS ES+
2.08e6
332.2018
197.0966
333.2049
158.1541
198.1000
91.0548
250.1783
334.2080
%
m/z

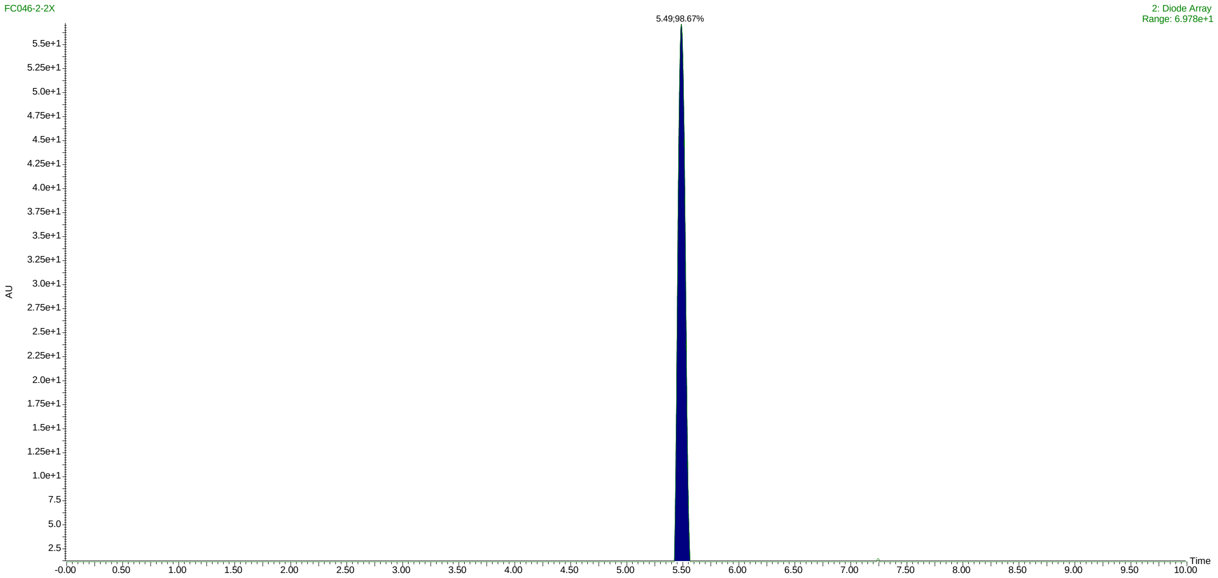
FC046-2-2X
2: Diode Array
Range: 6.978e+1
5.49;98.67%
AU
Time

Benzyl({[4-(benzyloxy)phenyl]methyl})propylamine (44):

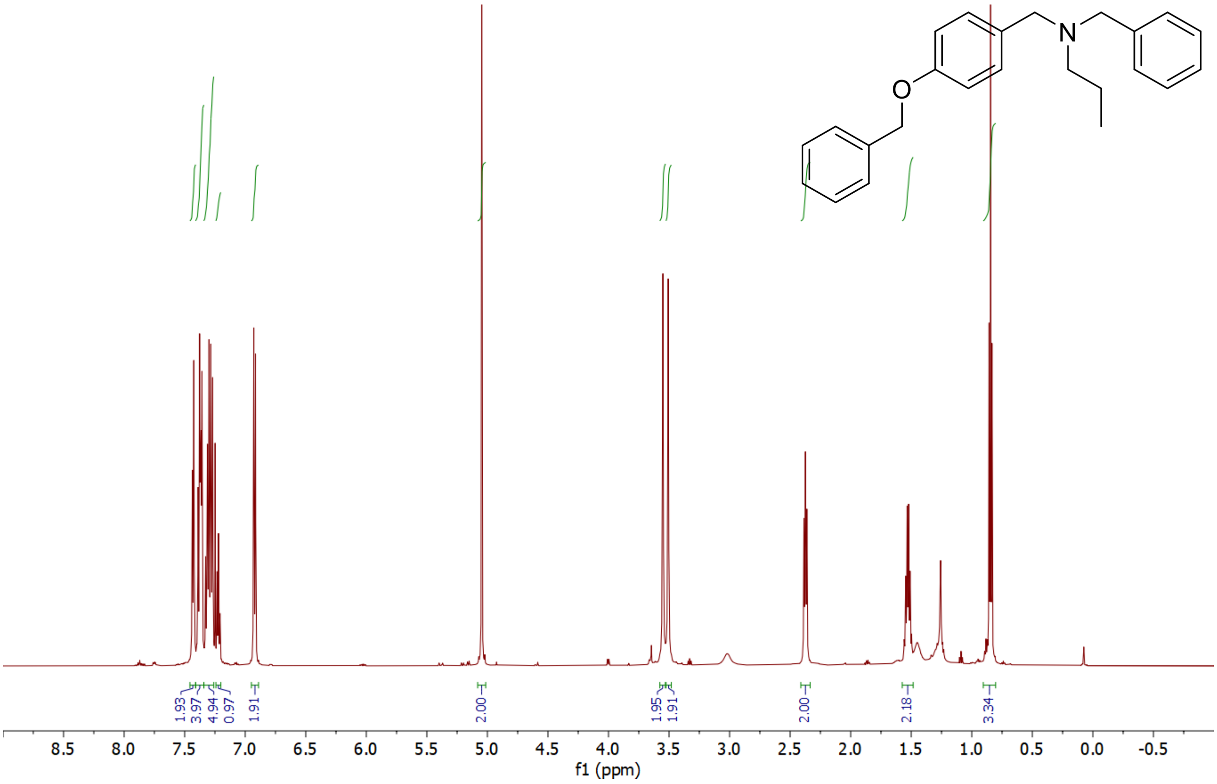

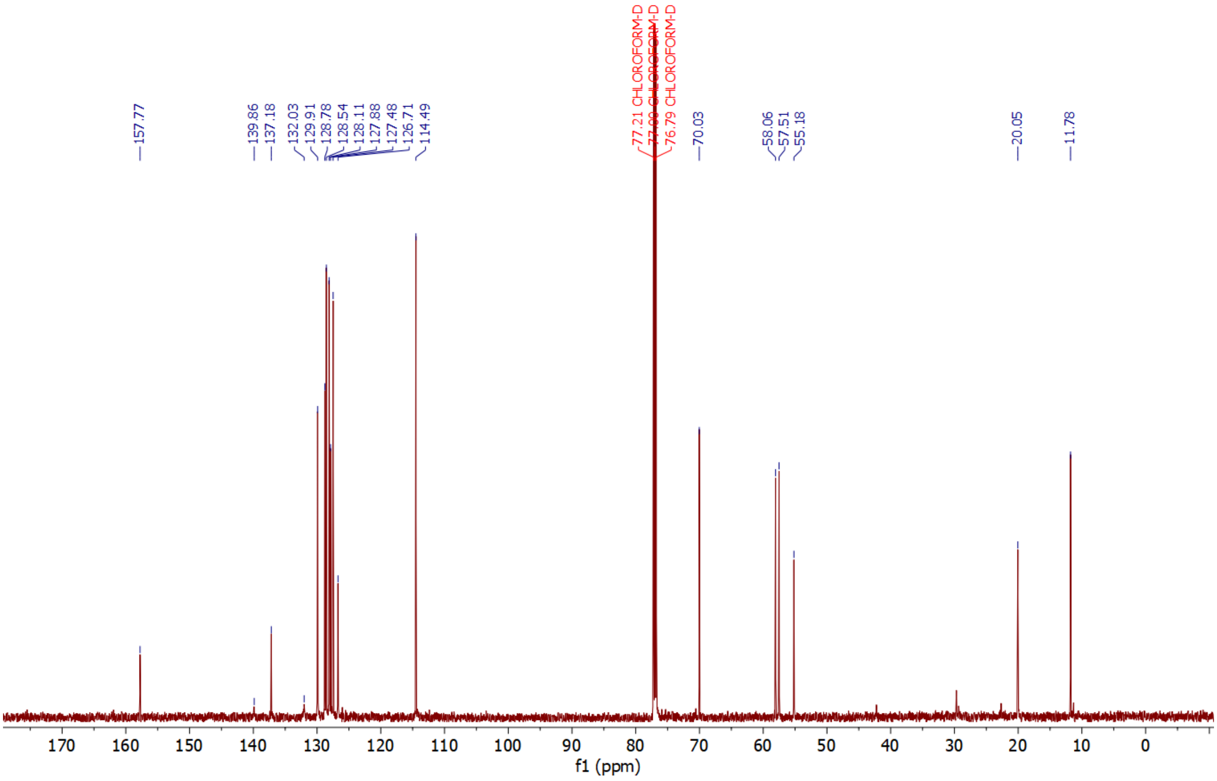

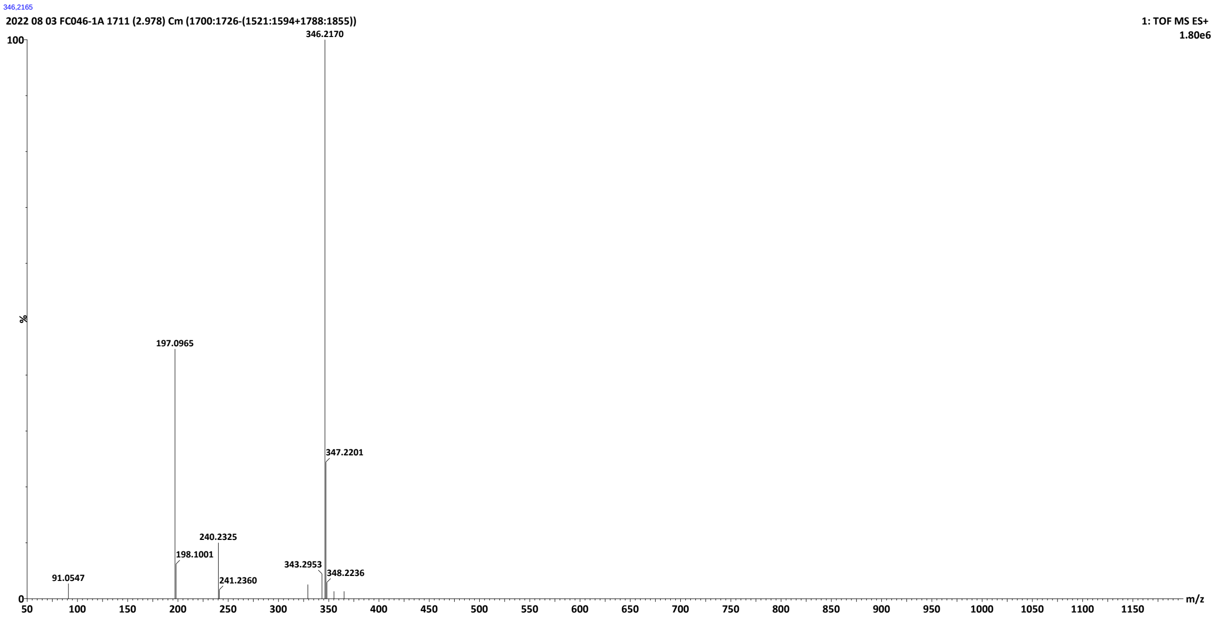
346.2165
2022 08 03 FC046-1A 1711 (2.978) Cm (1700:1726-(1521:1594+1788:1855))
1: TOF MS ES+
1.80e6
100
%
0
346.2170
197.0965
347.2201
240.2325
198.1001
343.2953
348.2236
91.0547
241.2360
50
100
150
200
250
300
350
400
450
500
550
600
650
700
750
800
850
900
950
1000
1050
1100
1150
m/z

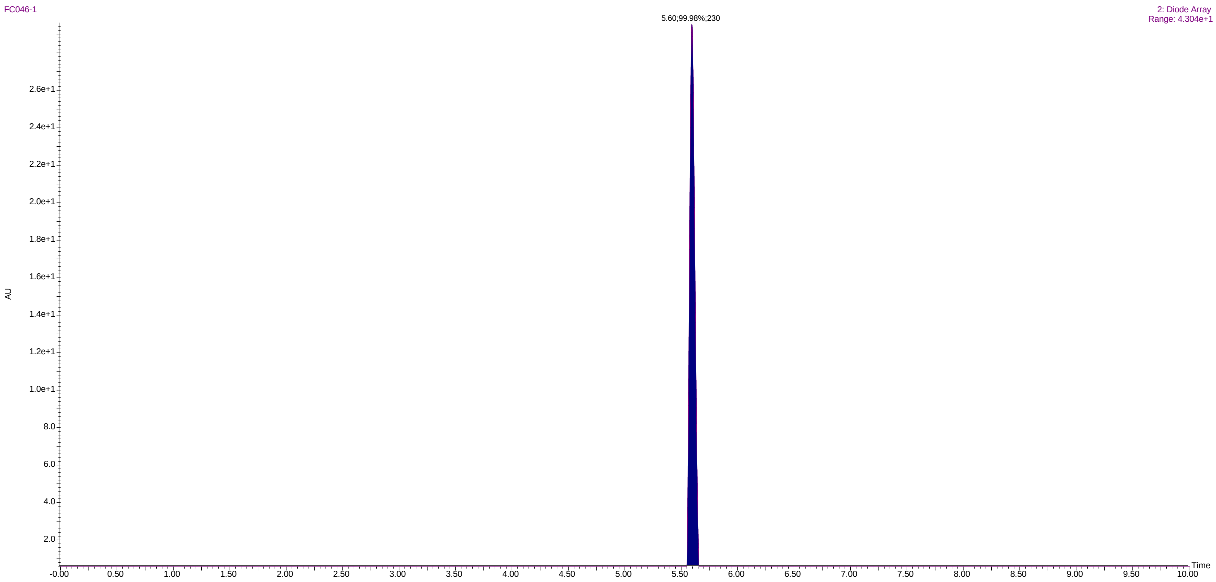
FC046-1
2: Diode Array
Range: 4.304e+1
5.60;99.98%;230
AU
2.6e+1
2.4e+1
2.2e+1
2.0e+1
1.8e+1
1.6e+1
1.4e+1
1.2e+1
1.0e+1
8.0
6.0
4.0
2.0
-0.00
0.50
1.00
1.50
2.00
2.50
3.00
3.50
4.00
4.50
5.00
5.50
6.00
6.50
7.00
7.50
8.00
8.50
9.00
9.50
10.00
Time

{[4-(Benzyloxy)phenyl]methyl}(3-phenylpropyl)propylamine (45):

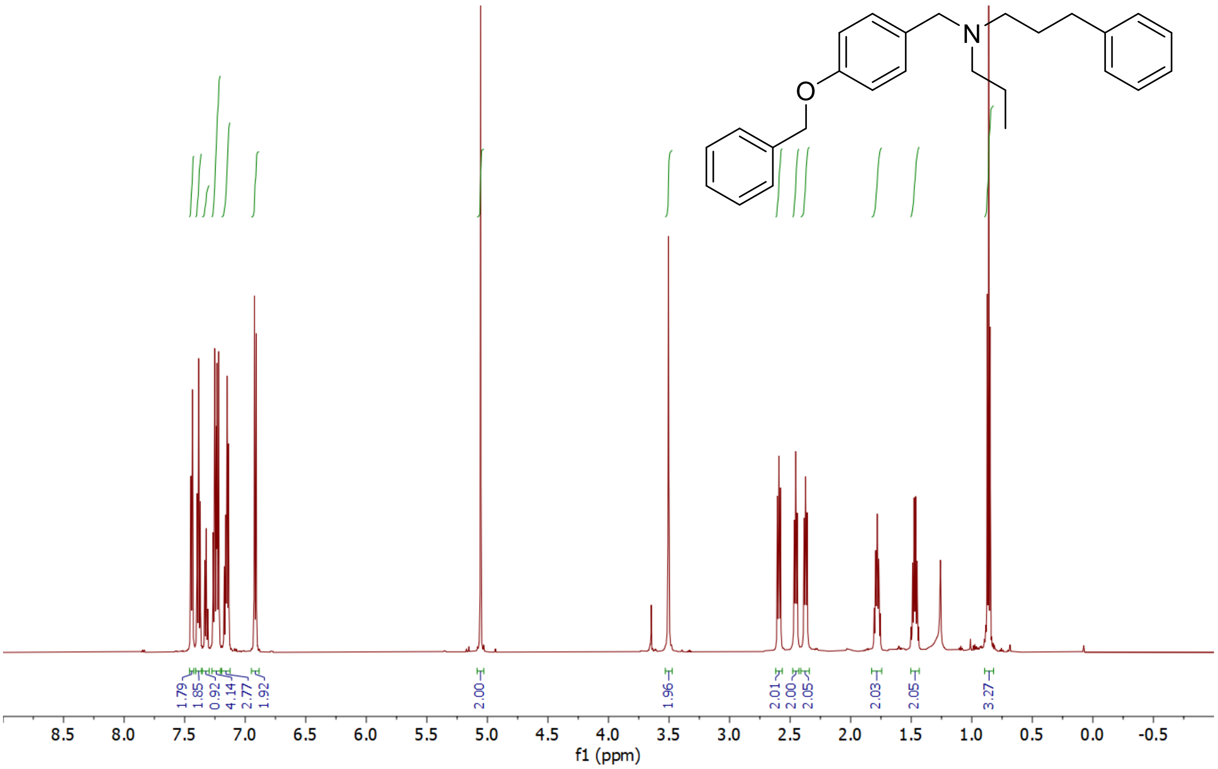

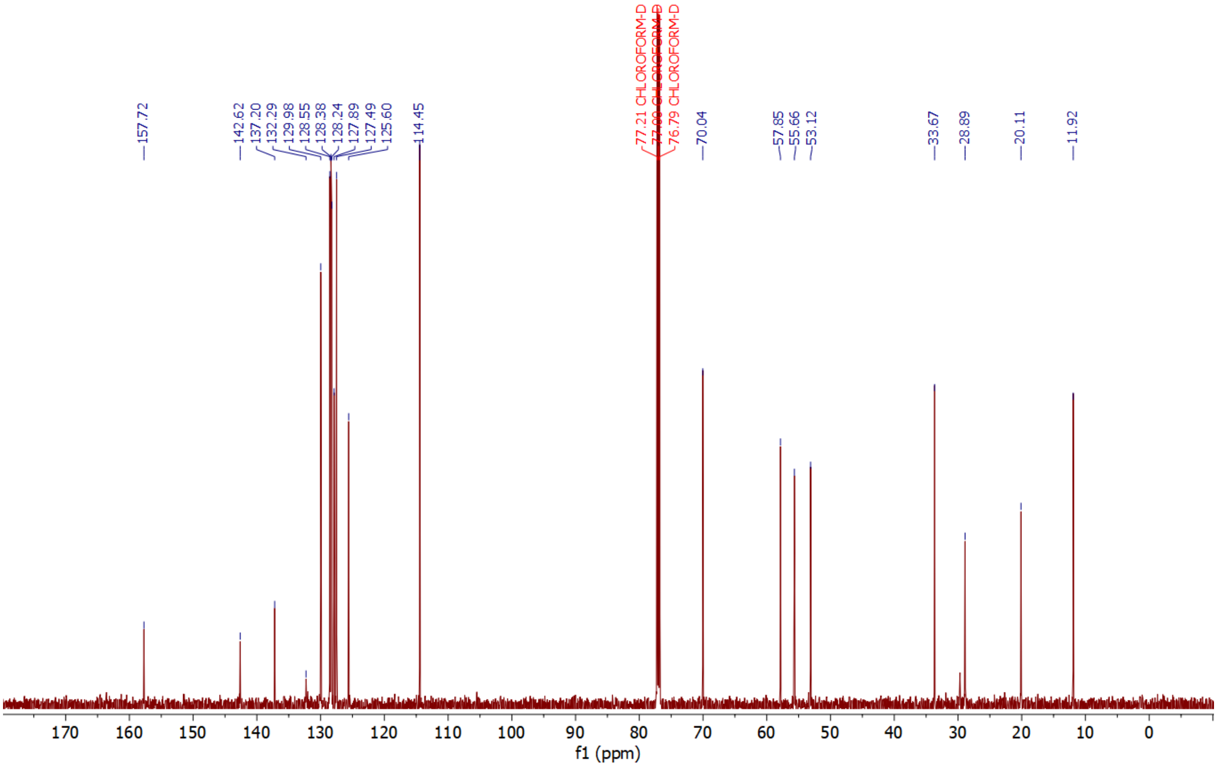

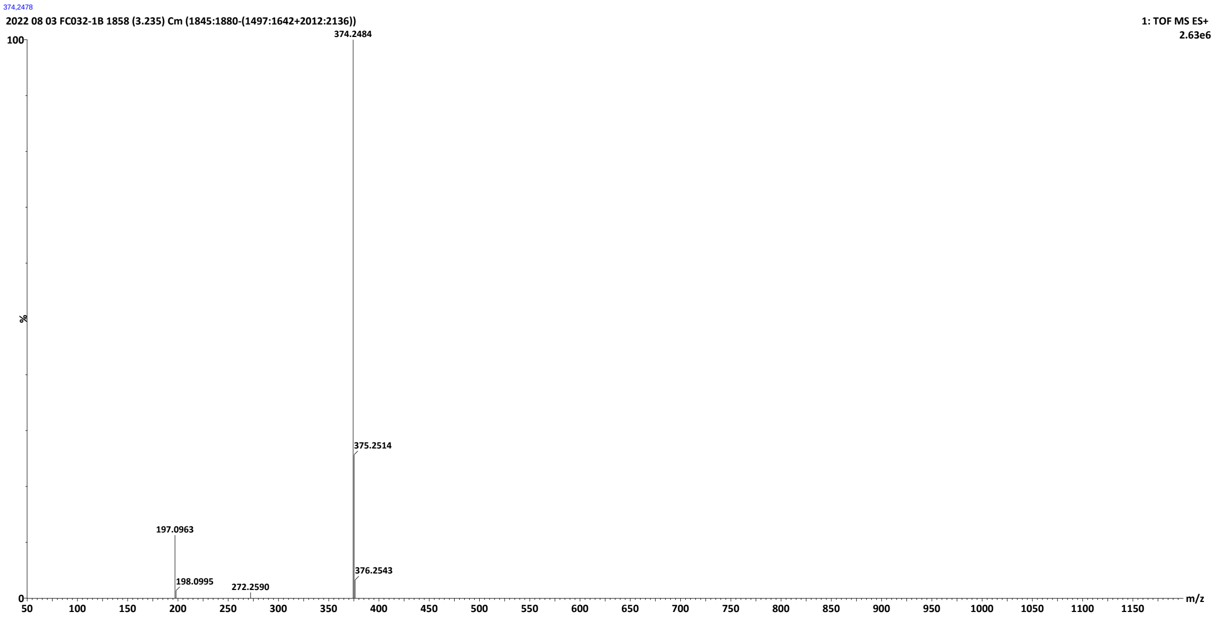

374.2478
2022 08 03 FC032-1B 1858 (3.235) Cm (1845:1880-(1497:1642+2012:2136))
1: TOF MS ES+
2.63e6
374.2484
375.2514
197.0963
198.0995
272.2590
376.2543
%
m/z

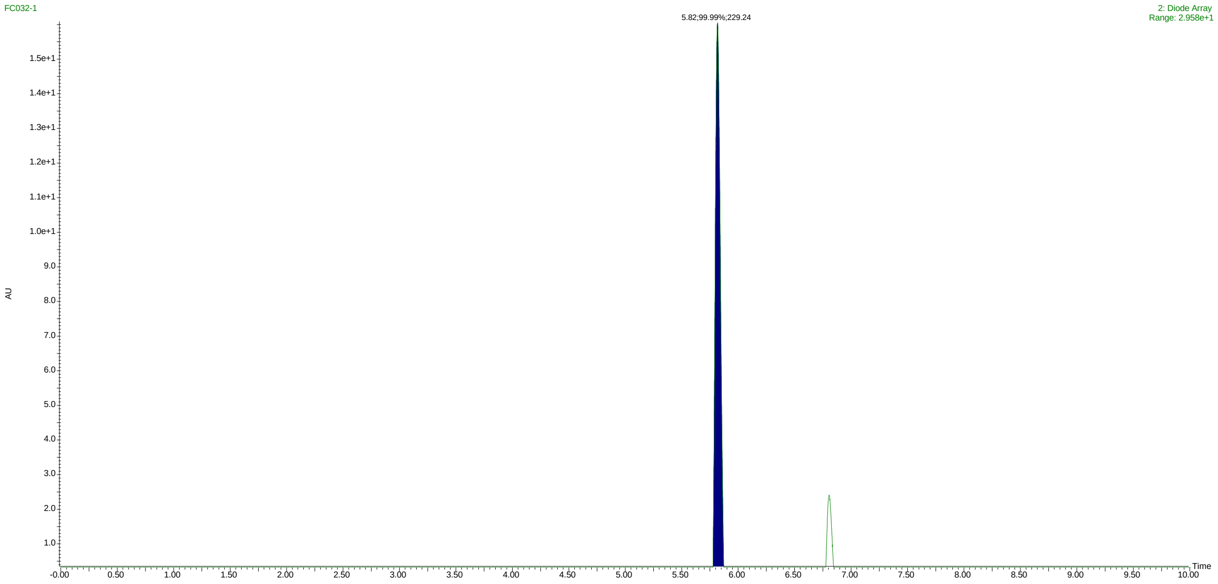

FC032-1
2: Diode Array
Range: 2.958e+1
5.82;99.99%;229.24
AU
Time

{[4-(Benzyloxy)phenyl]methyl}(ethyl)(4-phenylbutan-2-yl)amine (46):

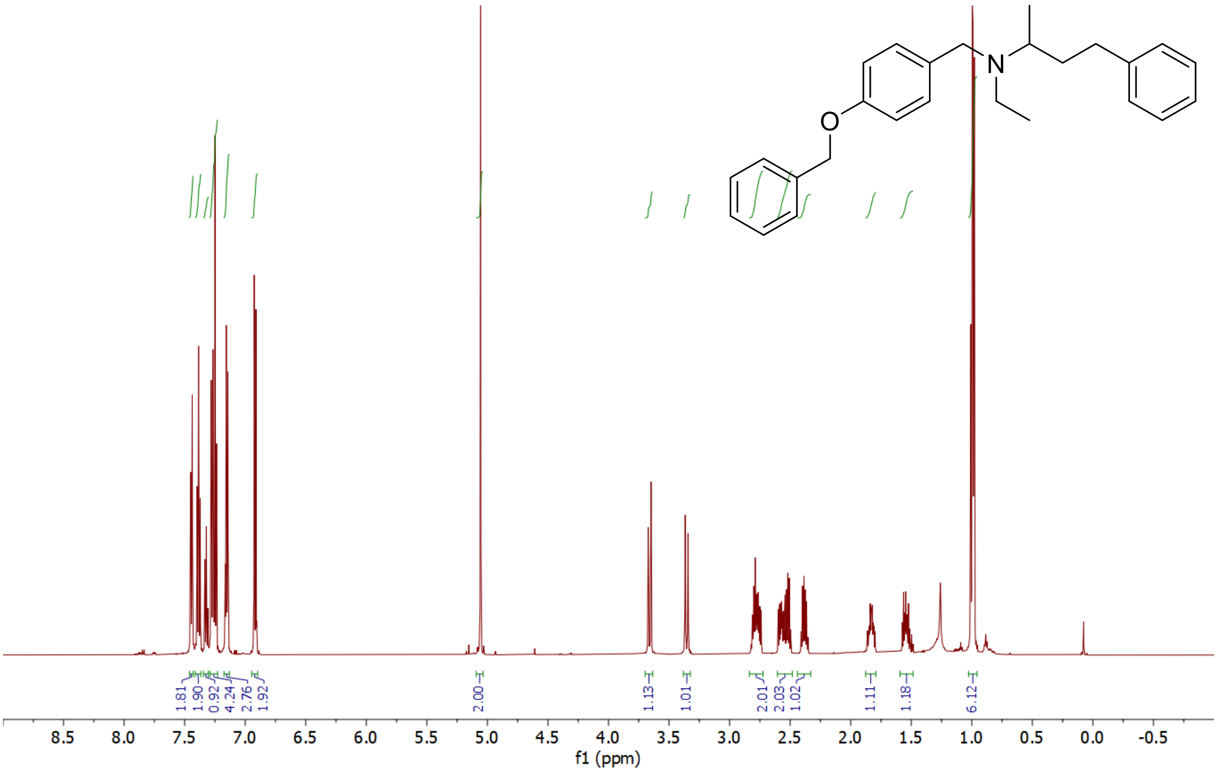

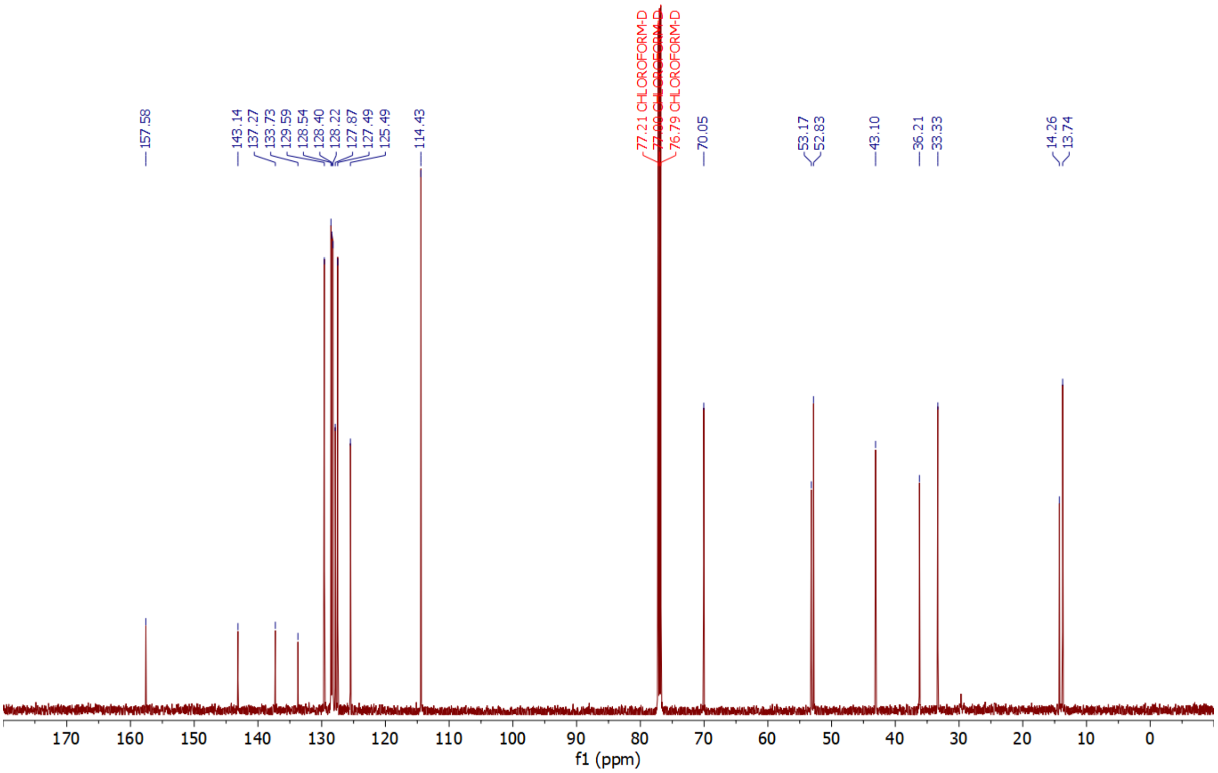

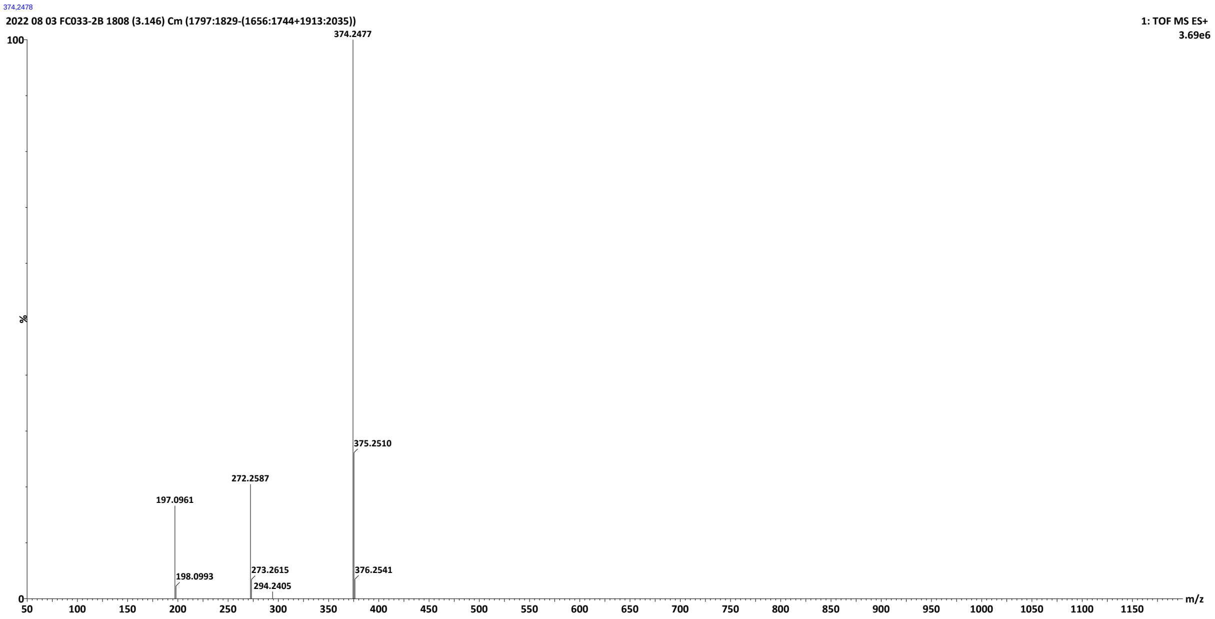
374.2478
2022 08 03 FC033-2B 1808 (3.146) Cm (1797:1829-(1656:1744+1913:2035))
1: TOF MS ES+
3.69e6
374.2477
375.2510
272.2587
197.0961
198.0993
273.2615
294.2405
376.2541
%
m/z

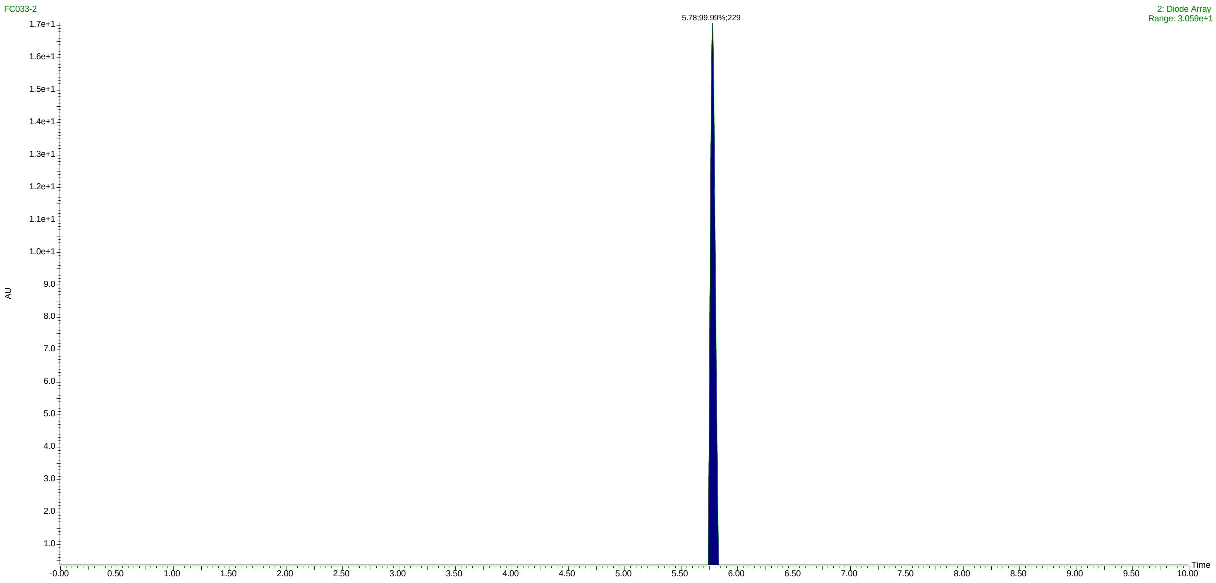
FC033-2
2: Diode Array
Range: 3.059e+1
5.78;99.99%;229
AU
Time

{[4-(Benzyloxy)phenyl]methyl}(4-phenylbutan-2-yl)propylamine (47):

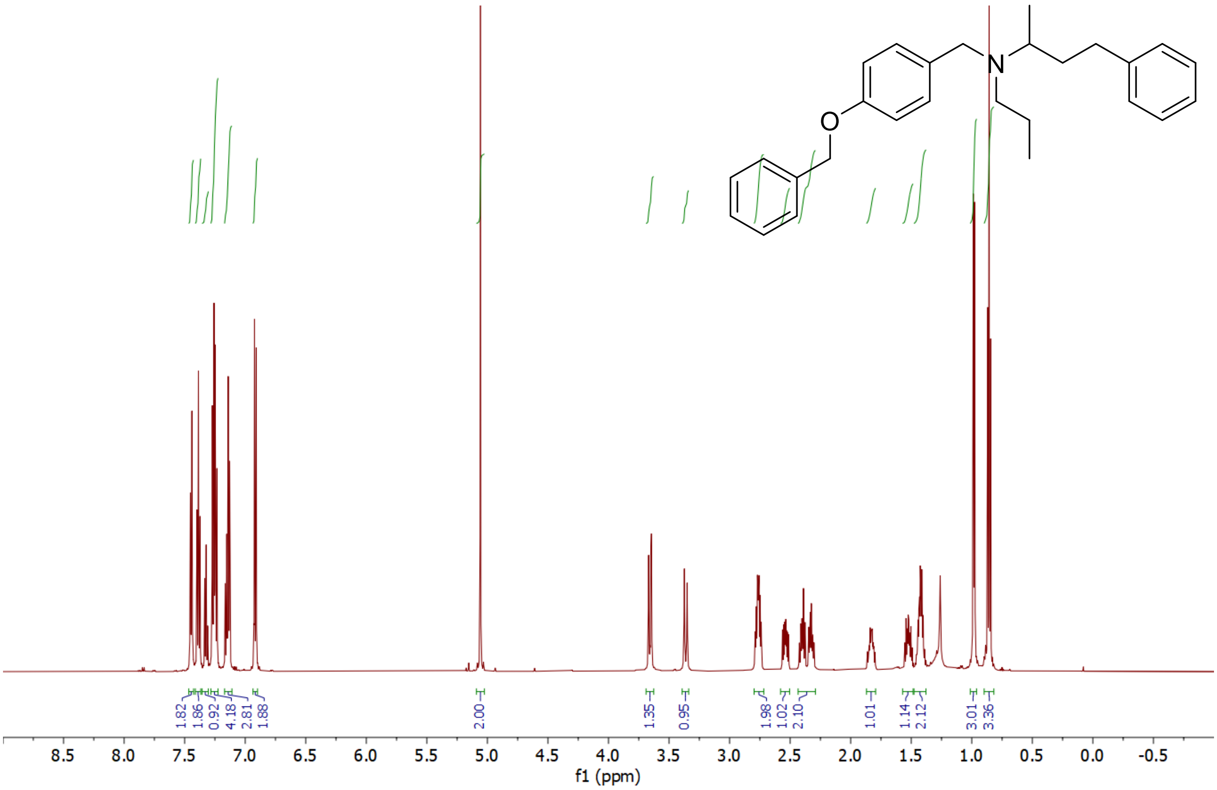

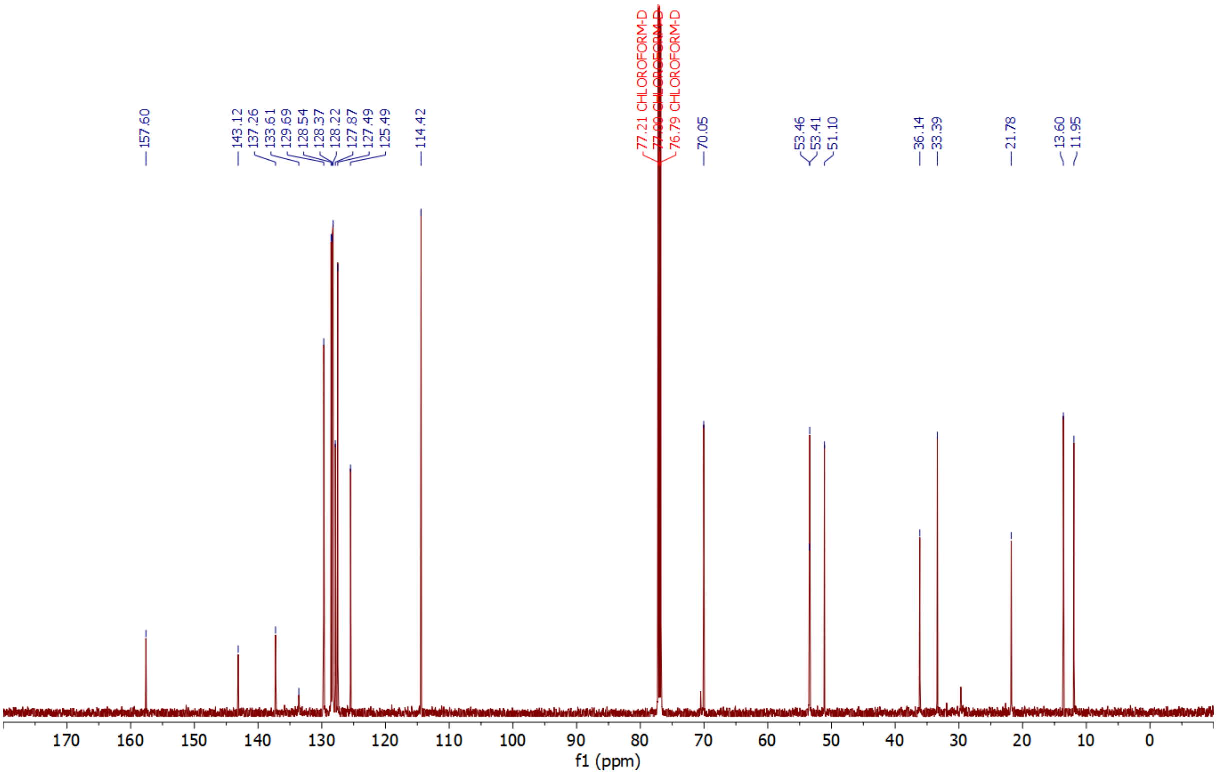

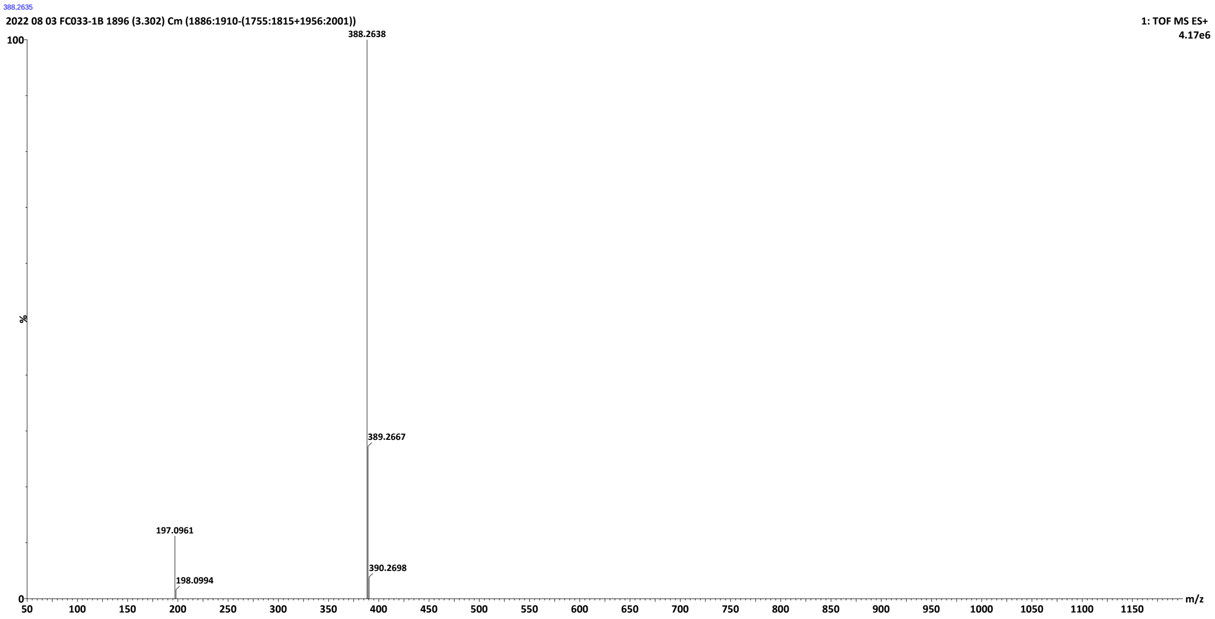
388.2635
2022 08 03 FC033-1B 1896 (3.302) Cm (1886:1910-(1755:1815+1956:2001))
1: TOF MS ES+
4.17e6
388.2638
389.2667
197.0961
198.0994
390.2698
%
m/z

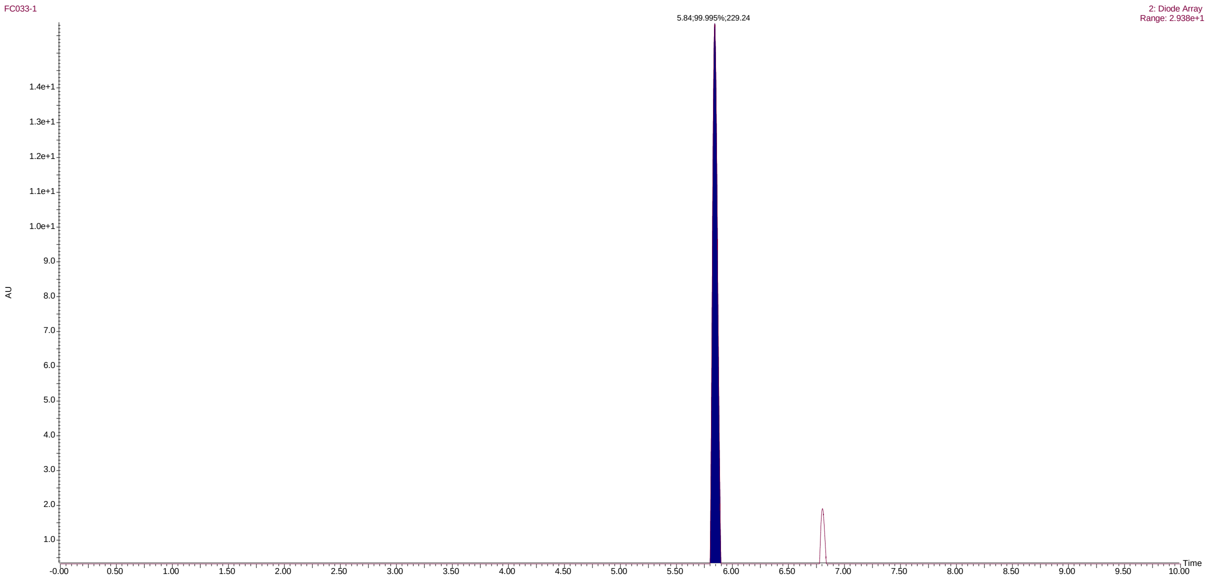
FC033-1
2: Diode Array
Range: 2.938e+1
5.84;99.995%;229.24
AU
Time

Dose–response curves for calculation IC$_{50}$ BChE values

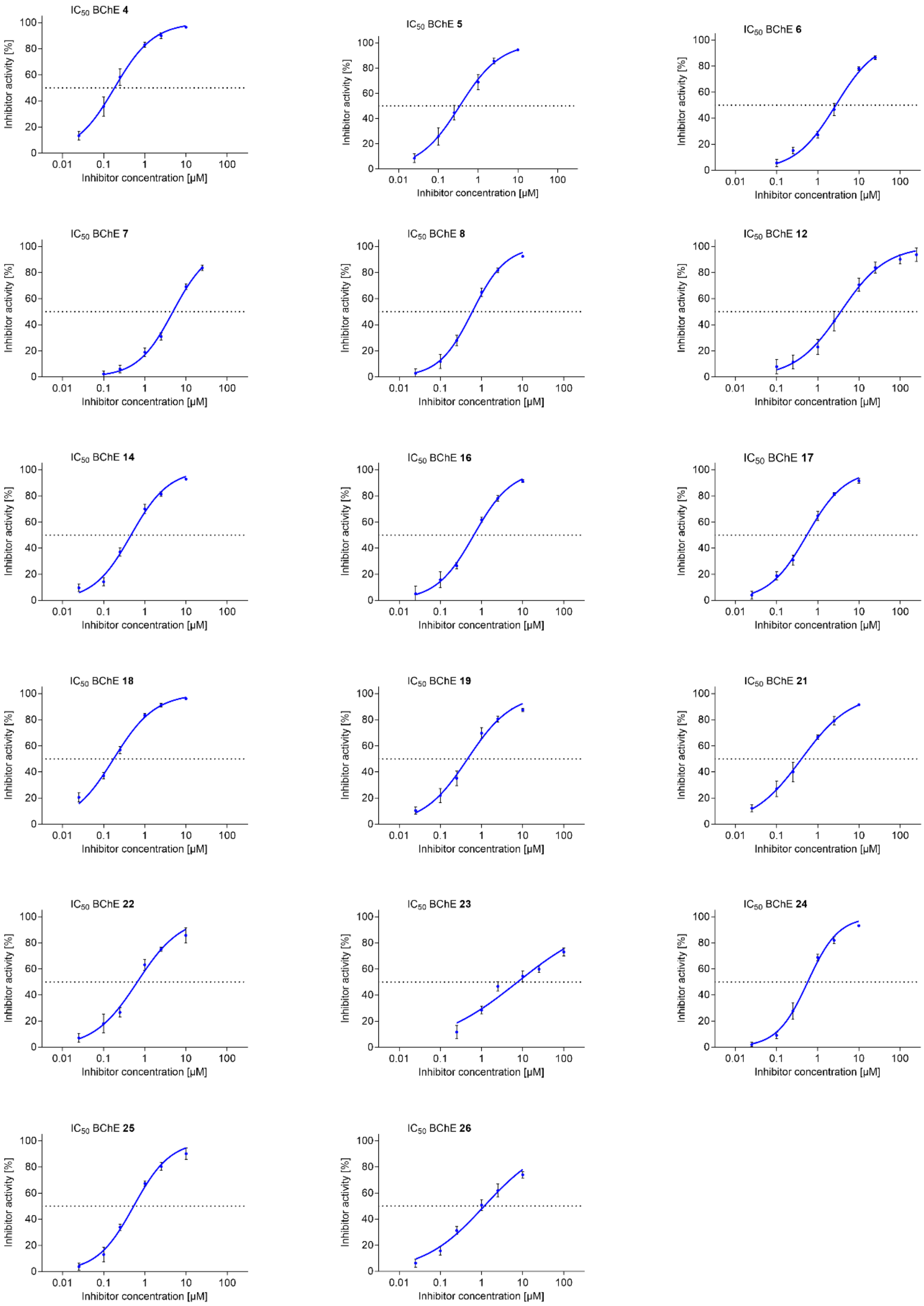

IC$_{50}$ BChE **28**

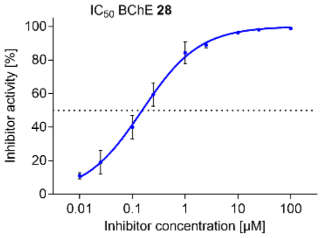

IC$_{50}$ BChE **29**

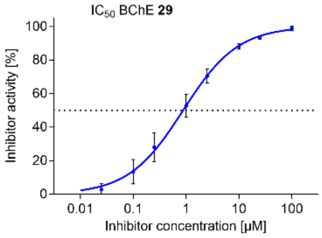

IC$_{50}$ BChE **30**

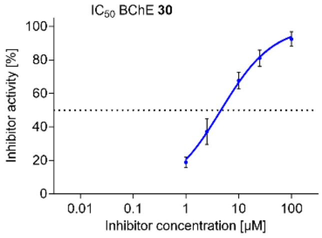

IC$_{50}$ BChE **31**

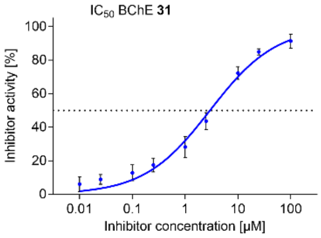

IC$_{50}$ BChE **32**

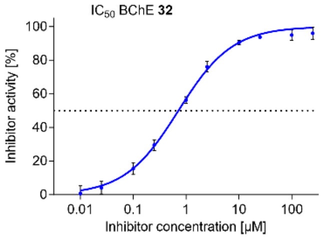

IC$_{50}$ BChE **33**

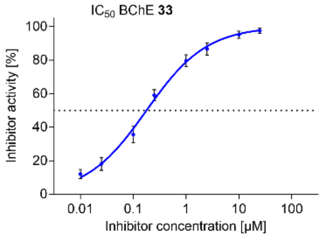

IC$_{50}$ BChE **35**

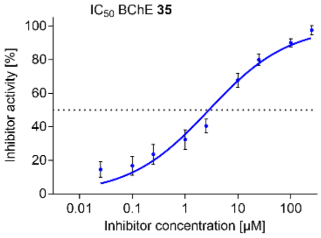

IC$_{50}$ BChE **37**

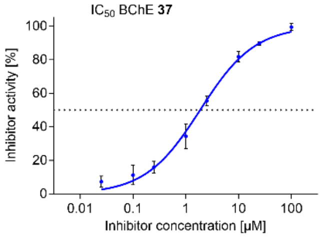

IC$_{50}$ BChE **39**

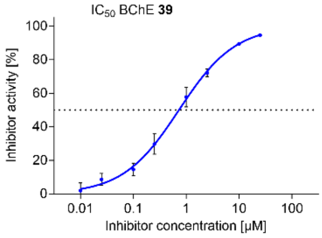

IC$_{50}$ BChE **40**

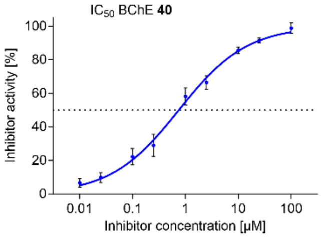

IC$_{50}$ BChE **41**

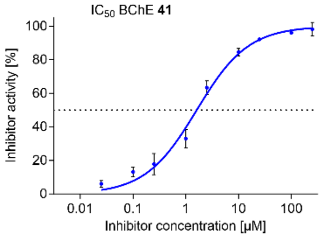

IC$_{50}$ BChE **42**

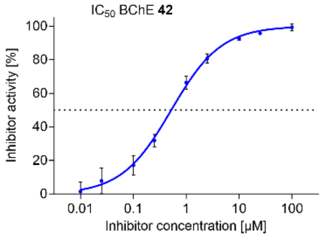

IC$_{50}$ BChE **45**

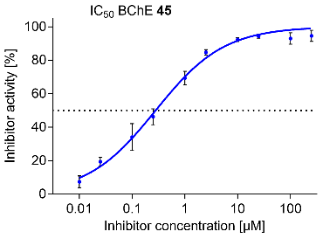

IC$_{50}$ BChE **46**

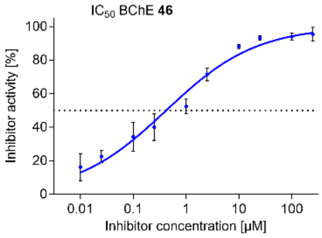

IC$_{50}$ BChE **47**

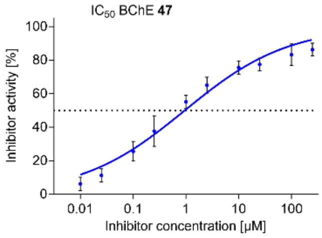

HLM stability

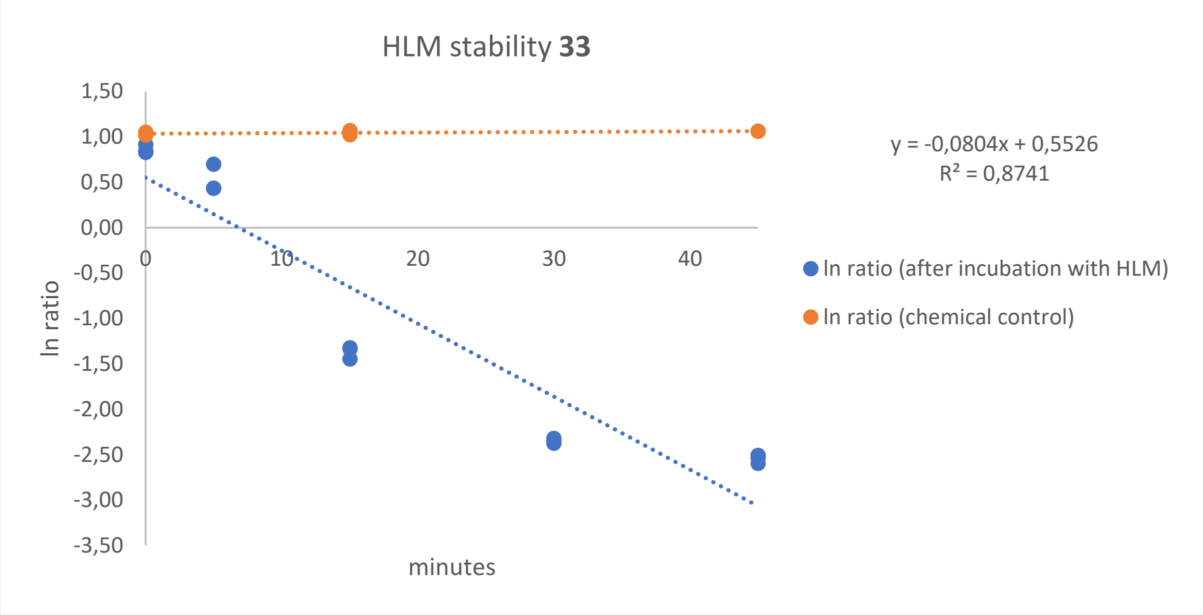

Human plasma stability

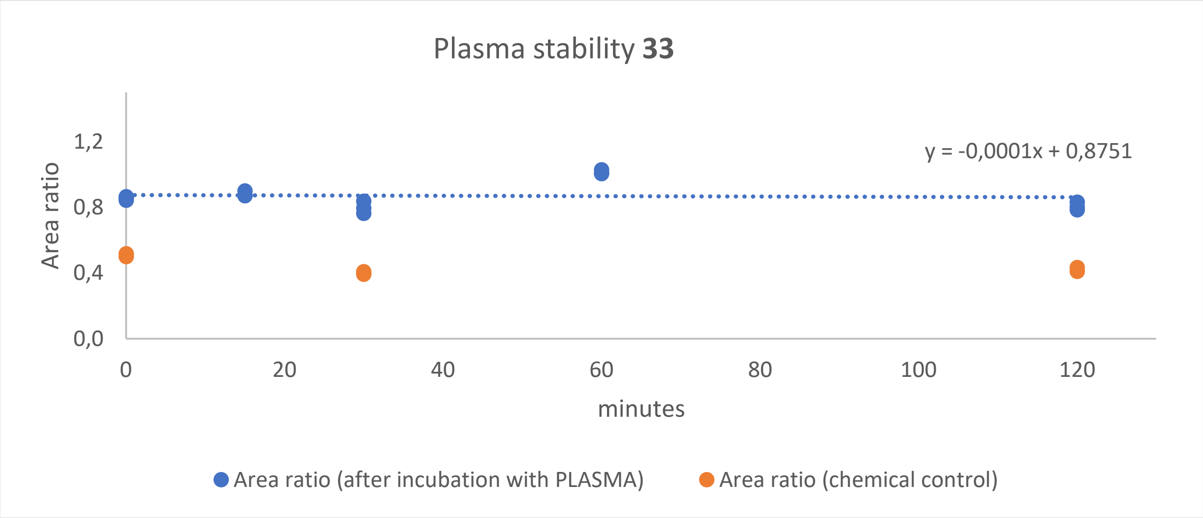

Supplement: MD-015-D4MD00060A-s001 [file MD-015-D4MD00060A-s001.pdf]
